# Transition transferases prime bacterial capsule polymerization

**DOI:** 10.1038/s41589-024-01664-8

**Published:** 2024-07-01

**Authors:** Christa Litschko, Valerio Di Domenico, Julia Schulze, Sizhe Li, Olga G. Ovchinnikova, Thijs Voskuilen, Andrea Bethe, Javier O. Cifuente, Alberto Marina, Insa Budde, Tim A. Mast, Małgorzata Sulewska, Monika Berger, Falk F. R. Buettner, Todd L. Lowary, Chris Whitfield, Jeroen D. C. Codée, Mario Schubert, Marcelo E. Guerin, Timm Fiebig

**Affiliations:** 1 Institute of Clinical Biochemistry, Hannover Medical School, Hannover, Germany; 2 German Center for Infection Research, Partner Site Hannover-Braunschweig, Hannover, Germany; 3 Structural Glycobiology Laboratory, Biocruces Bizkaia Health Research Institute, Cruces University Hospital, Barakaldo, Spain; 4 Structural Glycobiology Laboratory, Department of Structural and Molecular Biology; Molecular Biology Institute of Barcelona, Spanish National Research Council, Barcelona Science Park, Tower R, Barcelona, Spain; 5 Leiden Institute of Chemistry, Leiden University, Leiden, The Netherlands; 6 Department of Molecular and Cellular Biology, University of Guelph, Guelph, Ontario Canada; 7 Structural Glycobiology Laboratory, Center for Cooperative Research in Biosciences, Basque Research and Technology Alliance, Bizkaia Technology Park, Derio, Spain; 8 Proteomics, Institute of Theoretical Medicine, Faculty of Medicine, University of Augsburg, Augsburg, Germany; 9 Department of Chemistry, University of Alberta, Edmonton, Alberta Canada; 10 Institute of Biological Chemistry, Academia Sinica, Taipei, Taiwan; 11 Institute of Biochemical Sciences, National Taiwan University, Taipei, Taiwan; 12 Department of Biosciences and Medical Biology, University of Salzburg, Salzburg, Austria; 13 Department of Biology, Chemistry and Pharmacy, Free University of Berlin, Berlin, Germany; 14 Ikerbasque, Basque Foundation for Science, Bilbao, Spain

**Keywords:** Glycobiology, Infectious diseases, Enzyme mechanisms, X-ray crystallography, Bacteria

## Abstract

Capsules are long-chain carbohydrate polymers that envelop the surfaces of many bacteria, protecting them from host immune responses. Capsule biosynthesis enzymes are potential drug targets and valuable biotechnological tools for generating vaccine antigens. Despite their importance, it remains unknown how structurally variable capsule polymers of Gram-negative pathogens are linked to the conserved glycolipid anchoring these virulence factors to the bacterial membrane. Using *Actinobacillus pleuropneumoniae* as an example, we demonstrate that CpsA and CpsC generate a poly(glycerol-3-phosphate) linker to connect the glycolipid with capsules containing poly(galactosylglycerol-phosphate) backbones. We reconstruct the entire capsule biosynthesis pathway in *A. pleuropneumoniae* serotypes 3 and 7, solve the X-ray crystal structure of the capsule polymerase CpsD, identify its tetratricopeptide repeat domain as essential for elongating poly(glycerol-3-phosphate) and show that CpsA and CpsC stimulate CpsD to produce longer polymers. We identify the CpsA and CpsC product as a wall teichoic acid homolog, demonstrating similarity between the biosynthesis of Gram-positive wall teichoic acid and Gram-negative capsules.

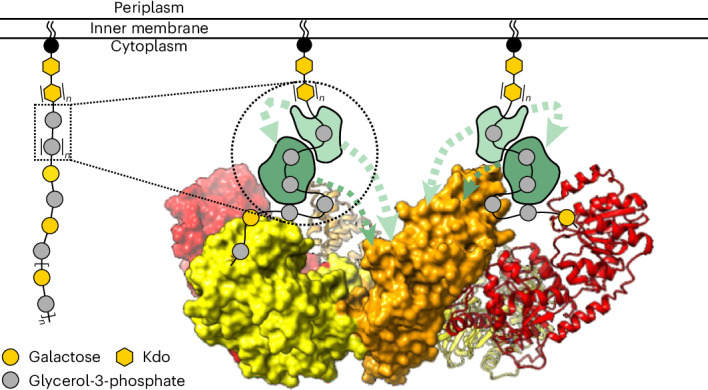

## Abstract (web summary)

The enzymes that link bacterial capsule polymers to the outermembrane glycolipids, termed transition transferases, are identified, enabling reconstruction of the entire capsule biosynthesis pathway.

## Main

Capsules, consisting of long-chain carbohydrate polymers (polysaccharides), are important virulence factors of bacterial pathogens^1^. In Gram-negative bacteria, capsules are generated according to three biosynthesis routes classified as Wzy-dependent, synthase-dependent or ABC transporter-dependent assembly systems^1^. The latter, often referred to as the group 2 system^2^, is used by human and animal mucosal pathogens that cause urinary tract infections, septicemia and meningitis, including extraintestinal pathogenic *Escherichia coli*, *Neisseria meningitidis* (Nm), *Haemophilus influenzae*, *Campylobacter jejuni*, *Pasteurella multocida* and *Actinobacillus pleuropneumoniae* (App)^1^.

At their reducing end terminus, group 2 capsule polymers are covalently attached to a glycolipid that anchors them to the outer membrane, allowing the formation of a dense and highly hydrated surface layer that confers protection against host defenses such as phagocytosis and complement-mediated killing^3^. The glycolipid anchor is highly conserved among all species and is assembled as part of an ABC transporter-dependent system. Its structure consists of phosphatidylglycerol attached to a polymer of β-linked 3-deoxy-d-*manno*-oct-2-ulosonic acid (poly(Kdo))^3^. In contrast, the capsule polymers attached to the glycolipid display high structural variability, giving rise to antigenic epitopes that are the foundation of capsule serotypes within a species^2^.

While the generation of the conserved glycolipid and the assembly of many serotype-specific capsule polymers are well understood^1^, the enzymatic repertoire required to link both components, and the chemical nature of the linking region, remain elusive. These knowledge gaps leave the question of how the dense capsule layer is attached to the cell surface unanswered. Here, we sought to identify and characterize the enzymes that assemble the linking region (hereafter referred to as transition transferases).

**Fig. 1:**
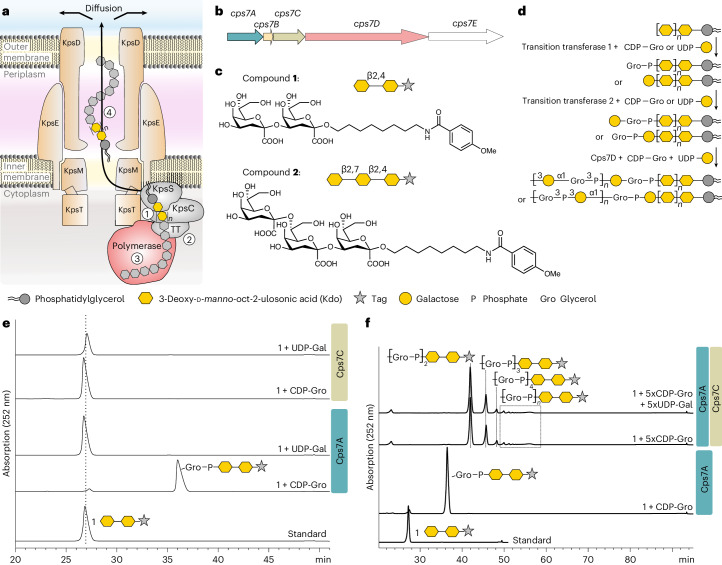
Identification of transition transferases. **a**, The current model for capsule assembly in ABC transporter-dependent systems comprises (1) the generation of a conserved glycolipid consisting of β-linked Kdo and phosphatidylglycerol, (2) the addition of a priming capsule polymer repeating unit by putative transition transferases (TT), (3) the elongation of said repeating unit by the capsule polymerase and (4) export of the polymer. **b**, Capsule gene cluster of App7, showing the capsule polymerase (Cps7D, pink), the enzyme providing CDP-Gro (Cps7B, yellow), putative transition transferases (Cps7A, turquoise; Cps7C, green) and a gene of unknown function (Cps7E, white). **c**, Chemical and symbolic structures of compounds **1** and **2**. **d**, Current working model for the in vivo synthesis of the capsule polymer in App7. The conserved glycolipid is extended by a putative transition transferase 1 that adds glycerol-phosphate or galactose. A second transition transferase 2 transfers galactose or glycerol-phosphate, completing the priming repeating unit, which serves as an acceptor for the capsule polymerase Cps7D, a two-domain enzyme that uses UDP-Gal and CDP-Gro as substrates for the generation of the capsule polymer. **e**,**f**, HPLC–AEC-based activity assay for the analysis of transition transferase activity of Cps7A (**e**) and Cps7C (**f**). Compound **1** was used as standard. Detection was performed at 252 nm. Nucleotide substrates and products are also detectable at this wavelength but elute early in the gradient (app. after 8–14 min; see Supplementary Fig. 3h for an example). **e**, Cps7A and Cps7C were incubated with compound **1** and either UDP-Gal or CDP-Gro. **f**, Cps7C utilizes CDP-Gro to generate a negatively charged polymer.

Capsule polymer production in ABC transporter-dependent assembly systems can be divided into four steps (Fig. 1a). In step I, the glycolipid anchor is generated, starting with the Kdo transferase KpsS that adds a single β-linked Kdo onto phosphatidylglycerol^4,5^. Subsequently, the two-domain Kdo-transferase KpsC^4,6,7^ generates poly(Kdo) with alternating β-(2→7)- and β-(2→4)-linkages. Step II is uncharacterized and comprises the synthesis of the region that connects glycolipid and capsule polymer. In step III, the serotype-specific capsule polymer is assembled by capsule polymerase(s), which are highly diverse, reflecting the structural variability of their products^1^. Step IV is the transport of the polymer from the cytoplasm to the outside of the cell via the ABC transporter coupled to a protein complex spanning the periplasm^1^ (Fig. 1a). The conserved glycolipid located at the reducing end of the polymer is believed to be the structural element recognized by the ABC transporter complex.

For *E. coli* K1 and K5 and for NmB, the capsule polymer is directly linked to the glycolipid^3,8^, leading to the hypothesis that transition transferases would transfer the first residue or repeating unit of the capsule polymer, thereby creating a primer for the capsule polymerase. The structural analysis of the linking region in K1 and K5 capsule polymers has been made possible by use of phage-borne depolymerases to remove the bulk of the capsular polymer, while leaving the poly(Kdo) and the first few capsule repeating units intact^3,8^. As such tools have not been identified for other group 2 capsules, the structural elucidation of the linking region in capsule polymers isolated from bacterial culture is infeasible using this approach.

To resolve this issue, we sought to identify transition transferases and reconstruct the entire biosynthesis of the capsular glycolipid in vitro, thereby generating the linker region in sufficient quantity and quality for structural analysis. Using App serotypes 3 and 7 as examples, we demonstrate that the linker between conserved glycolipid anchor and serotype-specific capsule polymer is assembled by two transition transferases. A tetratricopeptide repeat (TPR) domain is essential for the capsule polymerase to elongate said linker, and the transition transferases stimulate the capsule polymerase to produce long capsule polymers.

## Results

### Cps7A is a glycerol-3-phosphate transferase

**Extended Data Fig. 1:**
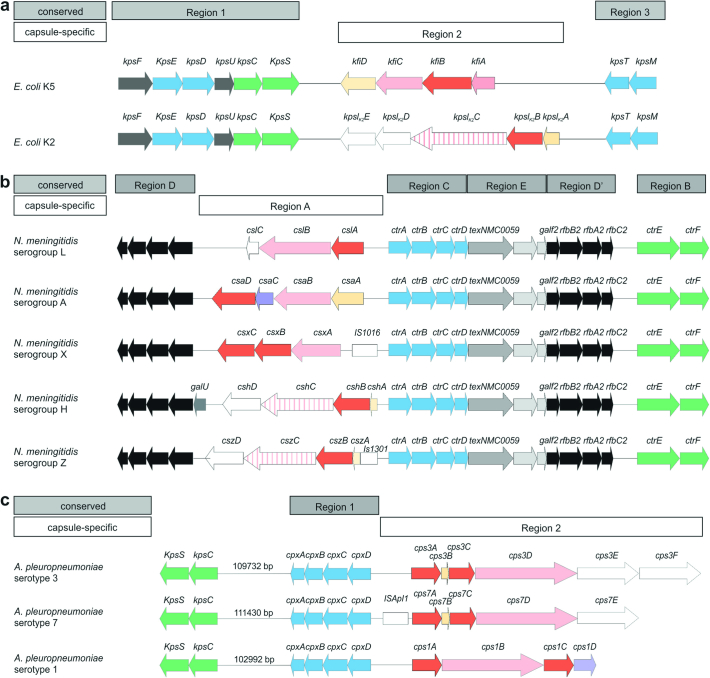
Capsule gene clusters containing transition transferases analyzed in this study. The organization of the cluster differs between **a**, *E. coli*^2^ **b**, *Neisseria meningitidis*^57^ and **c**, *Actinobacillus pleuropneumoniae*^58^. In all strains, the cluster is divided in conserved regions (grey boxes) and capsule-specific regions (white boxes), encoding enzymes involved in the generation of poly(Kdo) (green), proteins involved in polymer export (blue), and enzymes involved in capsule polymer synthesis^1^. Characterized (solid) or predicted (cross-hatched) capsule polymerases are shown in pink^9–11,13,14,17^. Enzymes involved in nucleotide substrate provision or polymer modification are shown in yellow^9,15,17,59^ or violet^9,13,60^, respectively. Putative transition transferases characterized herein (shown in orange) are CslA (CCP19790.1, NmL), CsaD (CAM07516.1, NmA), CsxB/CsxC (ATG32052.1, ATG32051.1, NmX), KfiB (TEZ99055.1, *E. coli* K5), Cps1A/Cps1C (AWG96007.1, AWG96005.1, App1), Cps7A/Cps7C (ACE62294.1, ACE62292.1, App7).

**Extended Data Fig. 2:**
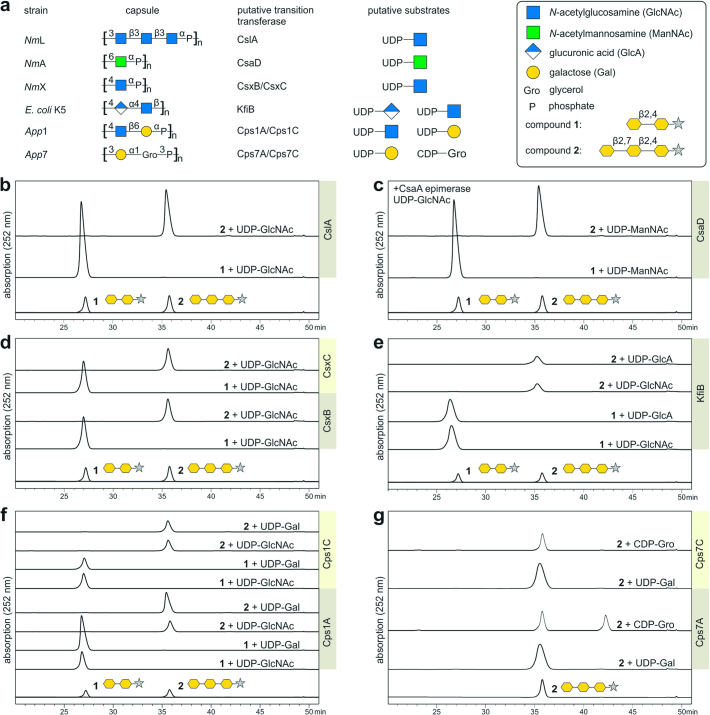
Putative transition transferases tested in this study. **a**, Overview of strain, capsule polymer repeating unit, putative transition transferase, and putative donor substrates for transition transferase candidates. *Nm*, *Neisseria meningitidis*; *App*, *Actinobacillus pleuropneumoniae*; *E*. *coli*, *Escherichia coli*. **b-g**, HPLC-AEC-based activity assays. Compounds **1** or **2** were incubated with the donor substrates and putative transition transferases as indicated. As UDP-ManNAc (panel **c**) is commercially unavailable, UDP-GlcNAc was supplied together with the epimerase CsaA, which has been used in a previous study to convert UDP-GlcNAc to UDP-ManNAc *in situ*^9^. Only Cps7A was able to utilize compound **1** (Fig. 1e) and compound **2** (panel g), leading to a species eluting at a later retention time. The inactivity of the other enzymes could be due to construct design, buffer conditions or choice of substrates. These enzymes could still be active transition transferases in the respective strains *in vivo*.

**Extended Data Fig. 3:**
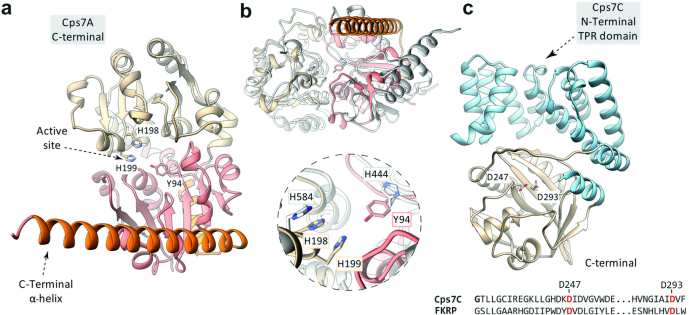
Characterization of Cps7A and Cps7C. **a**, AlphaFold model of Cps7A (Cps7A_AF_, B3GYR3; ACE62294.1; 380 residues) comprising two Rossmann-fold domains (beige, salmon), as observed in glycosyltransferase(GT)-B enzymes^23,61^, and a C-terminal α-helix (orange). Catalytically important amino acid residues identified in this study (Supplementary Fig. 3a,b) are located in a cleft between these two domains. **b**, Structural alignment of Cps7A_AF_ with the crystal structure of TagF^18^, the WTA type I polymerase from *Staphylococcus epidermidis*, an enzyme with Gro3P transferase activity (PDB code 3L7L; 15% sequence identity). In TagF, H444 likely functions as an active-site base, while H584 seems to play an essential role in catalysis by forming a complex with the pyrophosphate group of CDP-Gro^18^. H198/H199 of Cps7A_AF_ superimpose with H584 of TagF, while H444 of TagF superimposes with Y94 of Cps7A_AF_. Interestingly, H444 is part of the WHG motif in TagF and TagF-like Gro3P polymerases^14^. In GT-B fold hexose-phosphate transferases^14^ and other GT-B fold enzymes^62^, variations of the WHG motif comprise a QHG or a QYA motif, respectively. **c**, AlphaFold model of Cps7C (B3GYR1; ACE62292.1; 378 residues), comprising the N-terminal tetratricopeptide repeat (TPR) domain (blue, residues 1-190 and 368-378) and the C-terminal nucleotidyltransferase (NTase) domain^31^ (beige, residues 203-365). TPRs commonly mediate protein–protein interactions and the assembly of multi-protein complexes, but also the recognition of polymers^37^. Interestingly, the NTase domain of Cps7C displays homology with FKRP, an enzyme that modifies the phosphor-core M3 structure of the O-glycan of human α-Dystroglycan^63^. Using CDP-ribitol as donor, FKRP transfers the second ribitol-phosphate moiety of a tandem ribitol-phosphate and thus, like Cps7C, utilizes a polyol-phosphate as acceptor and a CDP-polyol as donor. FKRP (PDB code 6KAN, chain D) contains two aspartate residues important for catalytic activity^63^, which align with D247 and D293 of Cps7C according to PHYRE2^46^ homology modelling and are crucial for activity (Supplementary Fig. 3e).

Previous studies hypothesized that transition transferases would probably be serotype specific and, thus, located in the polymer biosynthesis region of the capsule gene cluster^4^, putatively encoded by genes currently lacking functional assignment (Extended Data Fig. 1). Capsule biosynthesis enzymes from Nm^9–12^, App^13–15^ and *E. coli* K5^16,17^ have been characterized previously. Candidates for transition transferases from these strains are CslA (NmL), CsaD (NmA), CsxB/CsxC (NmX), KfiB (*E. coli* K5), Cps1A/Cps1C (App1) and Cps7A/Cps7C (App7) (Fig. 1b and Extended Data Fig. 1). Recombinant proteins were expressed in *E. coli* and purified (Supplementary Table 1 and Supplementary Fig. 1). Two Kdo-based acceptor substrates (compounds **1** and **2**; Fig. 1c) mimicking the natural glycolipid were chemo-enzymatically synthesized as previously described^7^. Both compounds contain an ultraviolet (UV)-active *p*-methoxybenzamide tag, allowing the detection of transition transferase activity by high-performance liquid chomatography (HPLC)–anion exchange chromatography (AEC) (Fig. 1c). Compound **1** is a dimer of β-(2→4)-linked Kdo, and compound **2** contains an additional β-(2→7)-linked Kdo (Fig. 1c). Candidate enzymes were incubated with their respective donor substrates (nucleotide-activated carbohydrates/polyols; Fig. 1e and Extended Data Fig. 2) and compounds **1** or **2**, and analyzed after overnight incubation. None of the tested candidates from Nm, *E. coli* K5 and App1 was able to elongate compounds **1** or **2** (Extended Data Fig. 2b–f). However, a species eluting at later retention times (higher salt concentrations) was observed when compounds **1** and **2** were incubated with Cps7A (from App7) and CDP-Gro (cytidine diphosphate-activated glycerol, Fig. 1e and Extended Data Fig. 2g), indicating the transfer of a negatively charged moiety, presumably glycerol-phosphate. This transfer was specific for the Kdo present in compounds **1** and **2**, but not for sialic acid (Neu5Ac, 5-amino-3,5-dideoxy-d-glycero-d-galacto-non-2-ulosonic acid), which was used as a negative control (Supplementary Fig. 2). Bioinformatics analyses of Cps7A demonstrated sequence and structural similarity to the WTA type I polymerase TagF^18^ and TagF-like capsule polymerases^14^ with glycerol-3-phosphate transferase activity (Extended Data Fig. 3a,b and Supplementary Fig. 3a,b). Since compound **1** was consumed in <30 min by Cps7A, while compound **2** was still detectable after 360 min, the following experiments were performed with compound **1** (Supplementary Fig. 3c).

### Cps7C is a glycerol-3-phosphate polymerase

Because Cps7A transferred Gro3P onto poly(Kdo), we hypothesized that Cps7C was a galactosyltransferase required to complete the first repeating unit of the App7 polymer (Fig. 1d). A one-pot reaction containing Cps7A and Cps7C (Cps7A/C) together with compound **1** and UDP-Gal (uridine diphosphate-activated galactose)/CDP-Gro indeed yielded additional product, which eluted at higher salt concentrations (later retention times), again indicating the transfer of a negatively charged GroP instead of neutral Gal (Supplementary Fig. 3d). We compared Cps7C activity in the presence and absence of UDP-Gal and increased the concentration of CDP-Gro (and UDP-Gal) by fivefold to ensure sufficient supply of donor for the complete modification of compound **1**. Interestingly, new products appeared at later retention times and the product profile was independent of UDP-Gal (Fig. 1f). This finding together with bioinformatics analyses (Extended Data Fig. 3c, Supplementary Fig. 3e) indicate that Cps7C is a glycerol-phosphate polymerase. The Cps7C product can only be formed if CDP-3-Gro is provided as substrate (as opposed to the enantiomer CDP-1-Gro). Enantiopure CDP-3-Gro was generated in situ using Cps7B^15^ (Fig. 1b and Supplementary Fig. 3f,g) and is hereafter referred to as CDP-Gro.

### Structural characterization of Cps7A and Cps7C products

**Fig. 2:**
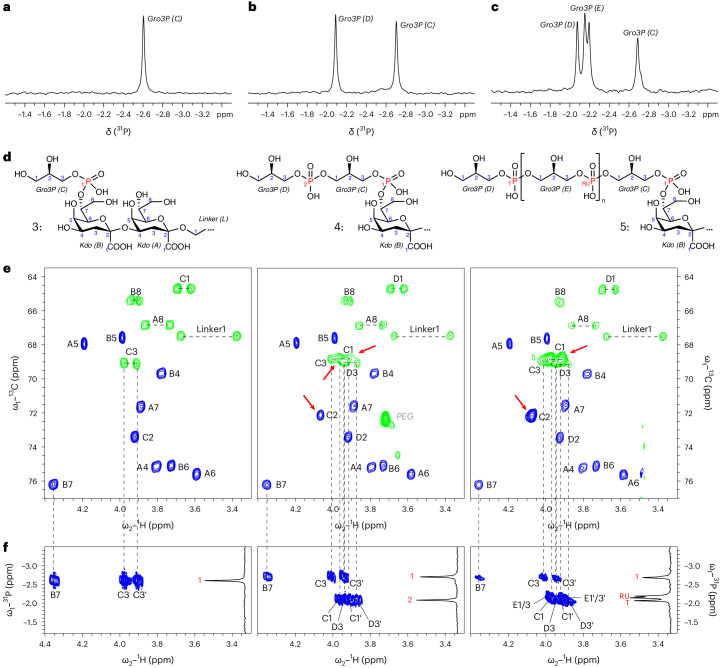
Comprehensive 1D and 2D NMR analysis of Cps7A and Cps7C reaction products. **a**–**c**, ^31^P NMR analysis of compound **3** (**a**), compound **4** (**b**) and compound **5** (**c**). **d**, Chemical structure of compound **3** (left), compound **4** (middle) and compound **5** (right). **e**,**f**, A combination of 2D ^1^H–^13^C HSQC (**e**) and ^1^H–^31^P HMBC (**f**) analysis was used to analyze the linkages between Kdo, phosphate and glycerol, demonstrating that Cps7A transfers Gro3P onto the 7-OH of the nonreducing end Kdo, thereby creating a new phosphodiester linkage. Cps7C adds additional Gro3P moieties onto 1-OH of the terminal glycerol phosphate (see Supplementary Table 3 for chemical shifts).

To characterize the products of Cps7A and Cps7A/C by nuclear magnetic resonance (NMR) spectroscopy and mass spectrometry (MS), products were purified from scaled-up reactions by preparative AEC, yielding compound **3** (**1** elongated by one Gro3P), compound **4** (**1** elongated by two Gro3P) and compound **5** (**1** elongated by several Gro3P) (Supplementary Fig. 3h–j). MS analysis confirmed that increased retention times detected by HPLC–AEC correlate with the addition of Gro3P moieties (Supplementary Fig. 4). The one-dimensional (1D) ^31^P NMR analyses of compounds **3**–**5** demonstrate that Cps7A and Cps7C are phosphotransferases, creating new phosphodiester linkages (Fig. 2a–c). A combination of two-dimensional (2D) ^1^H–^13^C HSQC (heteronuclear single quantum coherence) and ^1^H–^31^P HMBC (heteronuclear multiple bond correlation) experiments revealed that Cps7A catalyzes the transfer of Gro3P onto OH-7 of the nonreducing end Kdo of compound **1** (Fig. 2d–f, left). Cps7C then adds Gro3P onto OH-1 of the terminal Gro3P, generating a WTA^19^ type I-like Gro3P homopolymer (Fig. 2d–f, middle and right).

### The polymerase Cps7D elongates products of Cps7A and Cps7C

**Extended Data Fig. 4:**
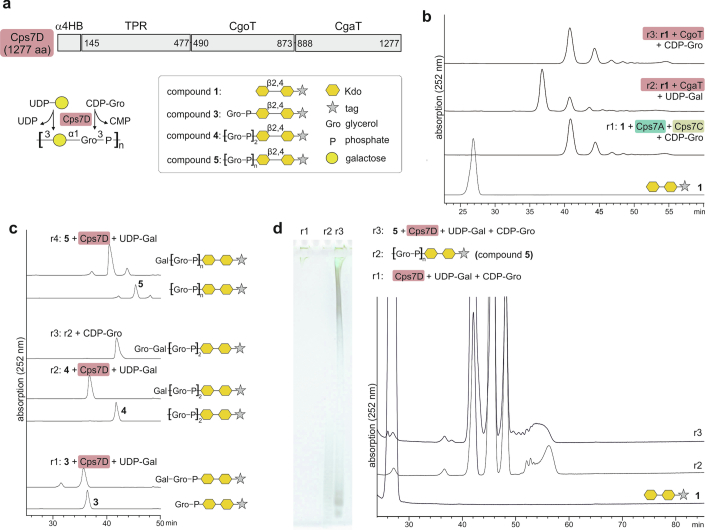
The reaction products of Cps7C are acceptor substrates for the α-1,1-galactosyltransferase (CgaT) domain of Cps7D. **a**, Cps7D consists of three regions from the N- to the C-terminus: (i) a domain rich in tetratricopeptide repeats (TPR), (ii) the Gro3P transferase CgoT (**C**apsule **g**lycer**o**l-3-phosphate **T**ransferase), (iii) a GT-B fold α-1,1-galactosyltransferase CgaT (**C**apsule α-1,1-**ga**lactosyl **T**ransferase), and an N-terminal α-helical bundle (α4HB). Cps7D requires UDP-Gal and CDP-Gro as substrates for polymerization. **b**, The reaction products of a Cps7A/C reaction were incubated with either UDP-Gal or CDP-Gro, and Cps7D-H743A or Cps7D-R1123A, in which either CgaT or CgoT remained active. Only CgaT could use poly(Gro3P) as acceptor, indicated by a shift of all species to earlier retention times due to the addition of a neutral galactose (r2). **c**, Compounds **3**, **4**, and **5** were incubated with Cps7D and UDP-Gal. While only 15% of compound **3** (the product of Cps7A) were used up by Cps7D over-night (r1), compounds **4–5** (products of Cps7C) were completely converted (r2, r4). The addition of one equivalent of CDP-Gro to reaction r2, or in other words, the addition of a complete repeating unit to compound **4** (r3), led to a species eluting at precisely the same time as compound **4**. **d**, Compound **5** was analyzed before (r2) and after (r3) incubation with Cps7D, CDP-Gro and UDP-Gal and products were visualized by PAGE (left) and HPLC-AEC (right), demonstrating that poly(Gro3P) can prime Cps7D. A reaction in the absence of compound **5** (r1) was performed as control for PAGE to document the extent of Cps7D’s previously published *de novo* activity^14,15^. A reaction in the presence of compound **1** was performed as standard for HPLC-AEC. Due to the complex composition of the polymer (tag, linker, Kdo, poly(Gro3P), (Gal-Gro3P)_n_), it is difficult to predict its electrophoretic mobility. Source data

Next, we investigated whether the products of Cps7A/C are substrates for the capsule polymerase of App7 (Cps7D^14,15^). Cps7D generates a polymer consisting of [→3)-α-Gal-(1→1)-Gro-(3-PO_4_^−^] and is a multi-modular enzyme comprising three regions (Extended Data Fig. 4a). Extended Data Fig. 4b demonstrates that the galactosyltransferase CgaT of Cps7D transfers Gal onto the products of Cps7A/C, while the Gro3P transferase CgoT did not add additional Gro3P residues (compare r2/r3 with r1). A suitable acceptor for CgaT comprises at least two Gro3P moieties, and its synthesis requires the combined activity of Cps7A and Cps7C (Extended Data Fig. 4c).

Interestingly, the addition of a complete repeating unit of the App7 polymer backbone to compound **4** led to a species eluting at precisely the same time as compound **4** (Extended Data Fig. 4c, compare r3 with **4**). Furthermore, the transfer of at least 5 RU to chemically synthesized and fluorescently labeled capsule polymer fragments (compounds **6** and **7**; Supplementary Fig. 5) was possible without considerably altering the retention time in the HPLC assay (Supplementary Fig. 6a–c). Importantly, for intermediate to long polymers, chain length does correlate with elution time and larger products could be separated and detected by HPLC–AEC (Supplementary Fig. 6b,d).

Due to the unusual elution of short-intermediate polymers in the HPLC–AEC, the elongation of compound **5** by Cps7D was analyzed by polyacrylamide gel electrophoresis (PAGE)^20^, which clearly demonstrated the addition of at least oligomers, if not polymers, even though the same products still eluted early in the HPLC–AEC assay (Extended Data Fig. 4d). In summary, the above presented results demonstrate that Cps7D utilizes poly(Gro3P) as an acceptor for oligo-/polymer assembly.

### Kdo is not required for poly(Gro3P) to be elongated by Cps7D

**Extended Data Fig. 5:**
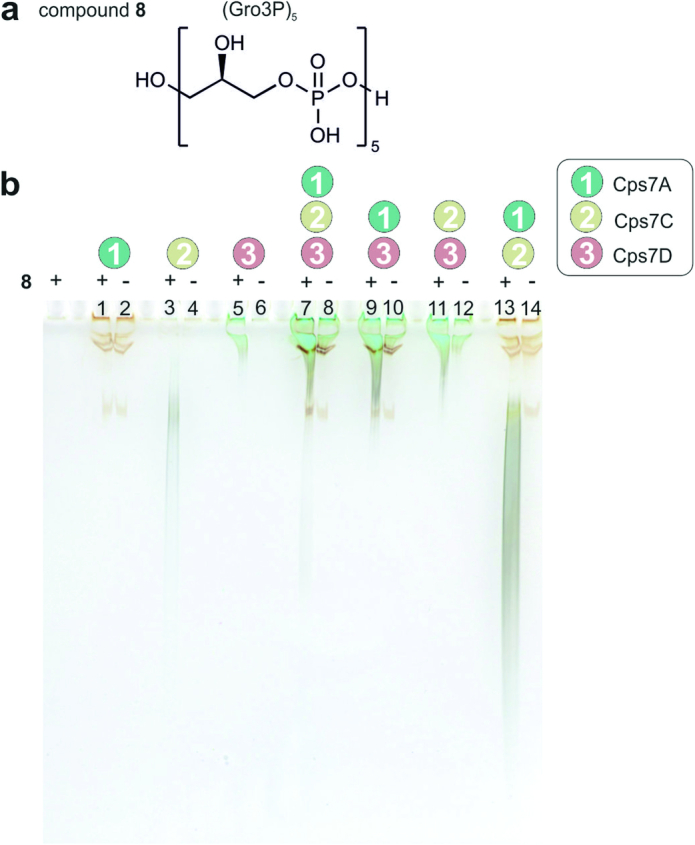
Kdo is not required for poly(Gro3P) to be recognized as acceptor by Cps7D. **a**, Chemical structure of the non-tagged Gro3P-pentamer (Gro3P)_5_ (compound **8**). **b**, Because compound **8** is not readily detected by UV, products were visualized by Alcian blue / silver staining after separation using PAGE^20^. Cps7D was used at a nanomolar concentration to avoid its previously shown *de novo* activity^14,15^ (production of long chains at high enzyme concentrations even without any acceptor present). Samples were incubated with different protein combinations as indicated and with or without compound **8** (+/−). Compound **8** is no suitable acceptor substrate for Cps7A (lane 1), but for the Gro3P polymerase Cps7C (lane 3) and the capsule polymerase Cps7D (lane 5). The presence of Cps7A stimulates Cps7D to produce polymer, both in the presence and absence of compound **8** (compare lanes 5 and 9, and lanes 6 and 10). Cps7A has a stimulating effect on Cps7C product formation (compare lanes 3 and 13). The presence of Cps7C has no detectable effect on product formation by Cps7A/D (compare lanes 7/8 with lanes 9/10). Cps7C alone also seems to increase polymer synthesis by Cps7D (lanes 11 and 12). Source data

Next, we asked if Kdo (the acceptor for Cps7A) was essential for Cps7D to initiate polymerization, or if poly(Gro3P), the product of Cps7A/C, would suffice. We generated a nontagged Gro3P-pentamer ((Gro3P)_5_, compound **8**; Extended Data Fig. 5a) according to established methods^21,22^ and tested its elongation. None of the enzymes generate detectable products de novo in the absence of compound **8** (Extended Data Fig. 5b, lanes 2, 4 and 6). As expected, compound **8** was not elongated by Cps7A (Extended Data Fig. 5b, lane 1) but was a suitable acceptor substrate for the Gro3P polymerase Cps7C and the capsule polymerase Cps7D (Extended Data Fig. 5b, lanes 3 and 5). Interestingly, the presence of Cps7A/C stimulated Cps7D to produce more polymer, both de novo as well as in the presence of acceptor compound **8** (Extended Data Fig. 5b, compare lanes 7–12 with lanes 5 and 6).

### Cps7A is required for the production of long polymer chains

**Fig. 3:**
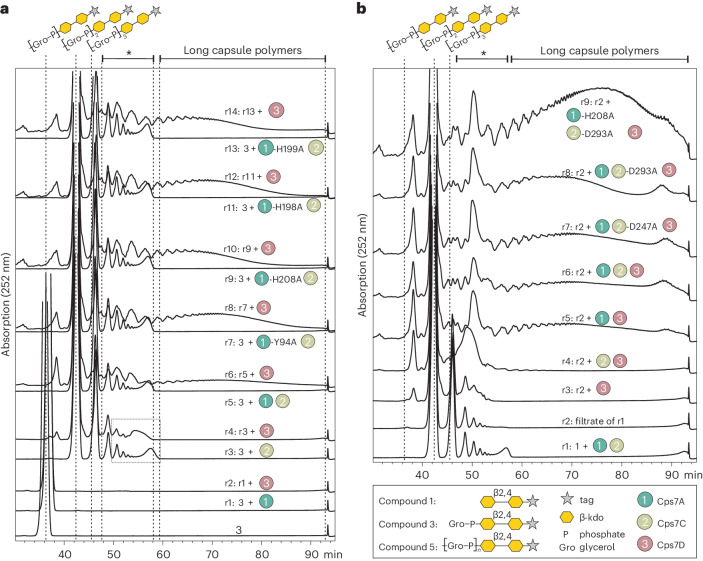
The presence of Cps7A promotes the generation of long chains by Cps7D. HPLC–AEC analysis of Cps7A/C/D reactions. Species eluting between 48 and 60 min (marked with asterisk) can comprise products of Cps7C and (short) products of Cps7D (see also Extended Data Fig. 4d). Long capsule polymers synthesized in Cps7A/(C)/D reactions have retention times between 60 and 90 min (see label at the top of **a** and **b**). **a**, Compound **3** was incubated with Cps7A or mutants of Cps7A, and Cps7C/D. The generation of long chains could only be observed in the presence of all three enzymes Cps7A/C/D. Interestingly, Cps7A could be substituted by inactive amino acid exchange mutants without losing the stimulating effect, demonstrating that the presence but not the activity of Cps7A is required once the product of Cps7A has been built. **b**, Compound **1** was elongated with Cps7A/C (r1), the enzymes were removed by filtration and the filtrate (r2) was used as substrate in subsequent reactions. Interestingly, long chains were produced in the presence of inactive Cps7C mutants and even in the absence of Cps7C, suggesting that, after the products of Cps7C have been assembled, the enzyme is not required for stimulating the assembly of long polymers.

To examine the possible influence of Cps7A and/or Cps7C on the length of polymers produced by Cps7D, the enzymes were incubated with compound **3**. As expected, Cps7A and Cps7A/D could not elongate **3** (Fig. 3a, r1 and r2), confirming that the products of Cps7C are required for elongation. Reactions containing Cps7C and Cps7A/C yielded very comparable product populations (Fig. 3a, r3 and r5). Cps7D was able to elongate the products generated by Cps7C, resulting in shorter retention times (Fig. 3a, box in r3/r4, and Extended Data Fig. 4d). Interestingly, only Cps7A/C/D together produced long chains (elution time 60–90 min; Fig. 3a, r6). Long chains were also produced in all reactions containing Cps7C/D in combination with a Cps7A catalytically inactive mutant (Fig. 3a, r8, r10, r12 and r14). These data indicate that, once the product of Cps7A (compound **3**) is generated, the presence of Cps7A is still required to stimulate the formation of long chains. Our data are consistent with an interaction between Cps7A and Cps7D that alters the catalytic properties of the polymerase.

We performed an analogous experiment with Cps7C and its catalytically inactive mutants. Because compounds **4** and **5** were not available in sufficient amounts for a large set of experiments, we first performed a Cps7A/C reaction starting from compound **1** (Fig. 3b, r1). Afterwards, Cps7A/C were removed by filtration, the product was recovered in the filtrate (Fig. 3b, r2) and used as substrate in subsequent reactions. In line with Fig. 3a, Cps7D alone could not elongate the substrates to produce long chains (Fig. 3b, r3) and only low amounts of larger chains were visible in a reaction containing Cps7C/D (Fig. 3b, r4). Surprisingly, long chains were only detected when Cps7D was combined with Cps7A or Cps7A-H208, irrespective of whether or not Cps7C or its inactive mutants Cps7C-D247A and Cps7C-D293A were present (Fig. 3b, compare r5–r9). In summary, these results indicate that Cps7A is required to generate long-chain App7 capsule polymer backbones.

### The structure of Cps3D

**Fig. 4:**
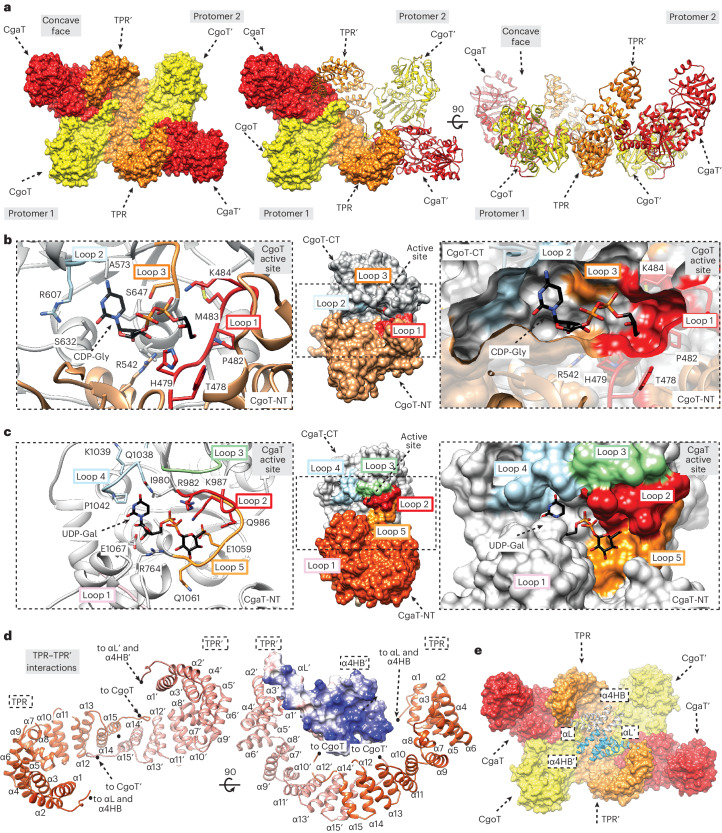
The overall structure of Cps3D. **a**, Each protomer of the homodimer is composed of a TPR domain (orange), the Gro3P-transferase CgoT (yellow) and the α-1,2-galactosyltransferase CgaT (red). The active sites of the two enzymes in each protomer face the concave surface of the homodimer. **b**, Middle: structure of CgoT. The N-terminal and C-terminal domains comprise residues 361–539 and 720–736 (CgoT-NT, orange) and residues 540–719 (CgoT-CT, gray), respectively. Left and right: CgoT active site showing the donor substrate CDP-Gro and selected loops and amino acid residues **c**, Middle: structure of CgaT. The N-terminal and C-terminal domains comprise residues 747–947 and 1,120–1,136 (CgaT-NT, salmon) and residues 948–1,119 (CgaT-CT, gray), respectively. Left and right: CgoT active site showing UDP-Gal and selected loops and amino acid residues. **d**, Structure of the TPR (dark orange) and TPR′ (salmon) domains. The predicted α4HB (α1_B_ (residues 10–24), α2_B_ (residues 27–41), α3_B_ (residues 45–59) and α4_B_ (residues 62–75)) and αL (residues 80–110) reveals a charge distribution with an intense positive character. **e**, Structure of Cps3D with possible location of the α4HB and αL structural elements shown as cartoon representation.

Because attempts to crystallize Cps7D failed, we solved the crystal structure of the homologous capsule polymerase Cps3D from App3 (38% sequence identity; A0A2S0ETM3; 1138 residues; Supplementary Fig. 7). The polymer backbones of App3 and App7 are composed of related poly(glycosylglycerol phosphate) structures ([→4)-α-Gal-(1→2)-Gro-(3-PO_4_^−^] and [→3)-α-Gal-(1→1)-Gro-(3-PO_4_^−^], respectively^15^) and both Cps3D and Cps7D require the alternating action of a CgoT and a CgaT domain for the transfer of Gro3P and Gal, respectively^14^. In addition, the capsule biosynthesis clusters of both serotypes are almost identical, containing CpsA and CpsC homologs with 93% and 96% sequence identity, respectively (Supplementary Fig. 7 and Extended Data Fig. 1). The crystal structure of Cps3D in its unliganded form was solved by molecular replacement methods to a maximum resolution of 3.0 Å (Fig. 4 and Supplementary Table 4). Cps3D (maltose-binding protein (MBP)-Cps3D_2–1138_-His_6_; Supplementary Figs. 1 and 8) crystallized in the *P* 3 2 1 space group with one molecule in the asymmetric unit (Supplementary Table 4), and the electron density maps allowed us to trace residues 100 to 1,145 (Supplementary Fig. 9).

Cps3D is a multi-modular enzyme composed of three regions from the N- to the C-terminus: (1) a TPR domain (residues 100–357; orange), (2) a CgoT domain (residue 358–736; yellow) and (3) a CgaT domain (residues 748–1,136; red) (Fig. 4a). The Cps3D protomers build into a physiological and functional dimer (Fig. 4a, overall size ca. 170 Å × 95 Å × 70 Å). Dimerization was confirmed by size exclusion chromatography (Supplementary Fig. 8d). The dimeric arrangement of Cps3D supports the presence of two reaction centers inside the concave side of Cps3D, comprising two dyads of enzymes—CgoT/CgaT and CgoT′/CgaT′ (Fig. 4a). The dimeric architecture is assembled primarily by an extensive interaction network between (1) the two TPR (α2–α15) and TPR′ domains and (2) the TPR domain and the N-terminal domain of CgaT′ (CgaT′-NT).

**Extended Data Fig. 6:**
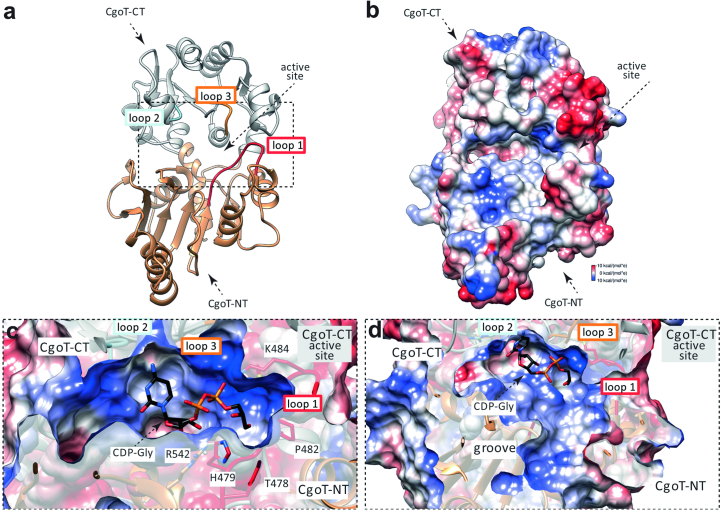
Structural basis of CgoT substrate specificity and catalysis in Cps3D. **a-b**, Ribbons representation (**a**) and electrostatic surface potential representation (**b**) of CgoT. The active site cleft is located between the two Rossmann domains (CgoT-NT; salmon, CgoT-CT; grey) and contains the essential residues for CgoT activity. The N-terminal and C-terminal domain cores are composed by seven-stranded and six-stranded parallel β-sheets, respectively, surrounded by α-helices (**a**). **c, d**, The structural comparison of CgoT with (i) the glycerol-phosphate polymerase TagF from *Staphylococcus epidermidis* in complex with CDP-glycerol^18^ (Supplementary Fig. 10a, PDB code 3L7K; root mean square deviation (r.m.s.d.) value between chains of 1.206 Å for 212 pruned atoms, and of 3.843 Å for all 355 pairs) and (ii) the capsule ribitol-5-phosphate transferase CroT from *H. influenzae* in complex with CMP^35^ (PDB code 8A0C; r.m.s.d. value between chains of 1.189 Å for 112 pruned atoms, and of 5.576 Å for all 292 pairs) revealed the active site architecture. The active site comprises α-helices 28-30, loop 1 (β5-α22, red), loop2 (α22-β10, light blue), and loop 3 (β11-α30, orange) (panels **a, c, d**). Exploiting the fact that TagF from *S. epidermidis* utilizes the same donor substrate as CgoT, we performed docking calculations revealing the location of CDP-glycerol in the active site (**c, d**). Key residues conserved between the two enzymes include F391, H479, G480, T481, P482, R542, A573, R607, S647, S648 and D652. Active site residues H479 and H609 of CgoT and their homologs in TagF^18,64^ and TagF-like capsule polymerases^14,35^ are crucial for enzyme activity (Supplementary Fig. 11). CgoT transfers Gro3P to the O4 position of the terminal galactose residue of the nascent chain, whereas TagF and Bcs3 transfer Gro3P and ribitol-5-phosphate to the O1 and O3 position of glycerol and ribose, respectively. By homology, we propose that CgoT follows a simple displacement mechanism similar to that observed in TagF^18^ and Bcs3^35^. We suggest that H479 acts as the putative base that contributes to the deprotonation of the O4 of the acceptor galactose residue, facilitating the nucleophilic attack of galactose to the β-PO_4_ of CDP-glycerol^18,35^.

**Extended Data Fig. 7:**
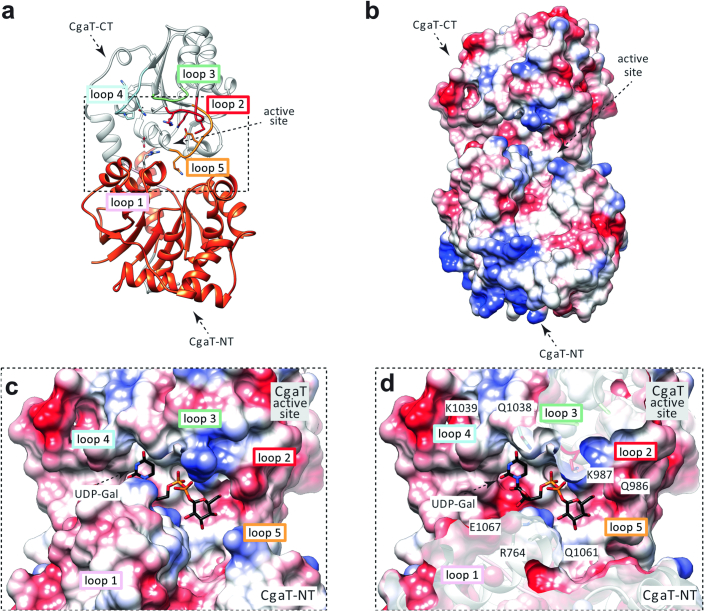
Structural basis for CgaT substrate specificity and catalysis in Cps3D. **a,b**, Ribbons representation (**a**) and electrostatic surface potential representation (**b**) of CgaT. The N-terminal (orange) and C-terminal (grey) domain cores are composed by seven-stranded and six-stranded parallel β-sheets, respectively, surrounded by α-helices (**a**). The active site is located in a deep fissure at the interface of the two Rossmann folds, suggesting an important inter-domain flexibility^23^. **c, d**, Structural comparison of CgaT with the glycosyltransferase BshA from *Staphylococcus aureus* in complex with UDP and N-acetylglucosamine^65^ (PDB code 6N1X; r.m.s.d. value between chains of 10.137 Å across all 339 pairs; Supplementary Fig. 10), support the location of the active site, which is defined by loop 1 (β20-α46; pink), loop 2 (β21-α48; red), loop 3 (β22-α49; light green), loop 4 (β23-α50; light blue), loop 5 (β24-α51; orange) and by helices α36, α51 and α52. Molecular docking calculations placed the donor UDP-Gal into the active site of CgaT. Residues R982 and K987 of the active site are highly conserved in TagF-like capsule polymerases^14^, crucial for Cps3D activity (Supplementary Fig. 11) and frequently involved in coordinating the phosphate moieties in retaining GT-B fold enzymes^66,67^. E1059 and E1067 are part of the EX_7_E motif found (among others) in the GT4 family of retaining glycosyltransferases^68^, which includes Cps3D homologs Cps2D and Cps7D (Cps3D is not yet listed in Carboyhdrate-active enzyme database CAZy^69^), as well as BshA^65^, PimA^67^ and TarM^66^. CgaT catalyzes the transfer of a galactose residue to the 2 OH position of the non-reducing end Gro3P of the nascent capsule polymer^14,15^. By homology, we suggest that CgaT is a retaining enzyme most likely following a S_N_i catalytic mechanism, involving an oxocarbenium ion transition state^23,70^.

CgoT adopts the typical GT-B fold of glycosyltransferases consisting of two Rossmann-fold domains^23^ (Fig. 4b, Extended Data Fig. 6 and Supplementary Fig. 10a). Similarly, CgaT displays a GT-B fold (Fig. 4c, Extended Data Fig. 7 and Supplementary Fig. 10b). The CgoT and CgaT active sites are located buried between the corresponding two Rossmann-fold domains, including the essential residues for catalytic activities (Fig. 4b,c). Using molecular docking calculations combined with site-directed mutagenesis, we deduced the catalytic mechanism of both enzymes (Extended Data Figs. 6 and 7, and Supplementary Fig. 11).

The TPR domain was modeled in the region spanning residues 100–357 and is displayed as 14 antiparallel α-helices and a small α-helix (α7) (residues 215–219) (Fig. 4a,d,e). The TPR and TPR′ domains form a continuous α-helical structure mainly mediated by the interaction of α14 (residues 320–332) and α15 (residues 336–346) from TPR with the equivalent α15′ and α14′ of TPR′, respectively, which seems important for the dimerization of Cps3D (Fig. 4d). Notably, we found no evidence for residues 2–100 in the electron density maps, suggesting structural flexibility. An atomic model for the N-terminal region of Cps3D was generated by AlphaFold^24^. This region probably comprises a bundle of four α-helices (α4HB) and a long α-helix (αL) that connects α4HB to the TPR domain (Fig. 4d,e). α4HB is a common structural motif characterized by a remarkable structural plasticity and known to mediate protein–protein interactions^25,26^.

### Comparison of the App3 and App7 biosynthesis systems

**Fig. 5:**
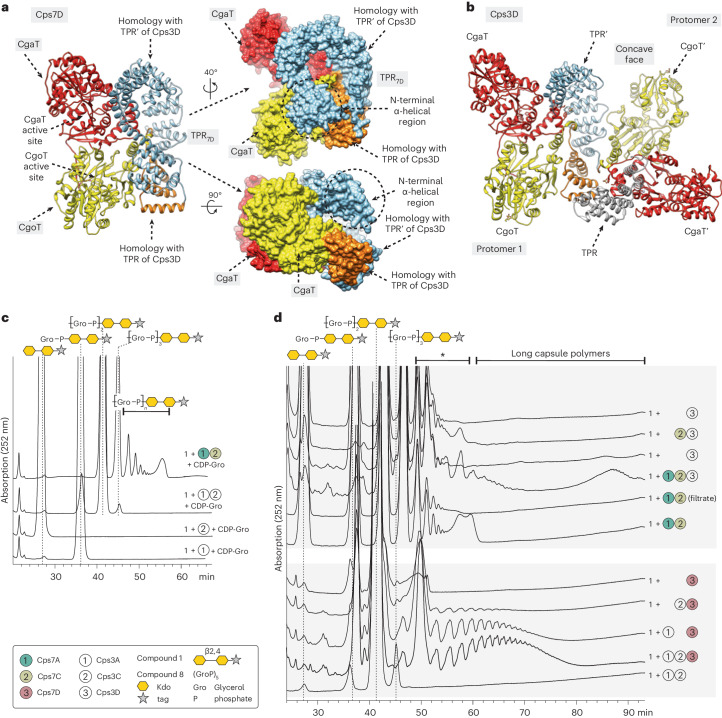
Differences and similarities between the App3 and App7 biosynthesis systems. **a**,**b**, AlphaFold model of Cps7D (**a**) as a cartoon representation (left) and surface representation (right) with rotations as indicated. Crystal structure of Cps3D as a cartoon representation (**b**). Color code: red, CgaT; yellow, CgoT; orange, homologous TPR repeats from monomeric Cps7D_AF_ (α16–α20) and protomer 1 of the Cps3D dimer (α11–α15); light blue, homologous TPR repeats from monomeric Cps7D_AF_ (α1–α15) and protomer 2 of the Cps3D dimer (α1′–α15′). **c**, Cps3A and Cps3C were incubated with compound **1** and CDP-Gro. Cps3A used compound **1** as acceptor and transferred one Gro3P moiety. Cps3C transferred Gro3P onto the product of Cps3A. A Cps7A/C reaction (from Supplementary Fig. 3f) is shown as a reference. **d**, The presence of Cps3A/C stimulates Cps7D to produce long polymers, and the presence of Cps7A/C stimulates Cps3D to produce larger products, indicating a high conservation of the stimulating effect and its components in both systems. Species eluting between app. 48 and 60 min (marked with asterisk) can comprise products of CpsC and (short) products of CpsD (see also Extended Data Fig. 4d). Long capsule polymers synthesized in CpsA/(C)/D reactions have retention times between 60 and 90 min (see label at the top of **d**).

Structural comparison between Cps3D with the Cps7D three-dimensional model generated by AlphaFold (Cps7D_AF_; ACE62291.1; 1,277 residues) revealed that the architecture of CgoT and CgaT in Cps3D is essentially preserved in the equivalent domains (CgoT_7D_ and CgaT_7D_) of Cps7D_AF_. Strikingly, Cps7D_AF_ predicts a longer TPR domain (TPR_7D_; residues 145–477) comprising 20 α-helices, which superimposes very well with (1) the α11–α15 TPR region of the equivalent Cps3D protomer 1 (Fig. 5a,b, orange) and (2) the α1′–α15′ TPR′ region of the neighbor protomer 2 observed in the dimeric Cps3D (Fig. 5a,b, blue). Judging from the Cps3D crystal structure, the interaction of α14/α15 of TPR with α14′/α15′ of TPR′ seems important for dimerization. In contrast to Cps3D, the TPR of Cps7D_AF_ additionally comprises α16–α20, and α14/α15 interact with α19/α20 of that same TPR domain. This suggests a monomeric architecture for Cps7D, which was confirmed by size exclusion chromatography (Supplementary Fig. 12a,b). Of note, the N-terminal region of Cps7D_AF_ (138 residues) is also predicted to contain several α-helices as observed in Cps3D_AF_.

To investigate the relationships between the App3 and App7 systems, we produced the putative transition transferases of App3. Like in the App7 biosynthesis system, Cps3A uses compound **1** as acceptor and transfers one Gro3P moiety, whereas Cps3C is not able to elongate compound **1** (Fig. 5c). Cps3C transfers Gro3P onto the product of Cps3A but adds fewer residues, creating a shorter linker in comparison with Cps7C (Fig. 5c and Supplementary Fig. 13a,b). Cps3D was able to elongate the Gro3P pentamer (compound **8**), indicating that Kdo is not required for the polymerase to recognize poly(Gro3P) as an acceptor (Supplementary Fig. 13c).

Similar to Cps7D (Extended Data Fig. 5), Cps3D produced more polymer in reactions containing Cps3A and/or Cps3C, both in the presence of compound **1** (Supplementary Fig. 13d, compare lanes 1–3 with lane 4) and de novo (Supplementary Fig. 13d, compare lanes 5 and 6). The detection of long chains in the HPLC assay could also be stimulated by Cps3A (to a lesser extent Cps3C) (Supplementary Fig. 13a,b). Importantly, long-chain polymers were detected even when CpsA/C from serotype 3 were combined with CpsD from serotype 7 and vice versa (Fig. 5d).

### Role of the TPR domain

**Fig. 6:**
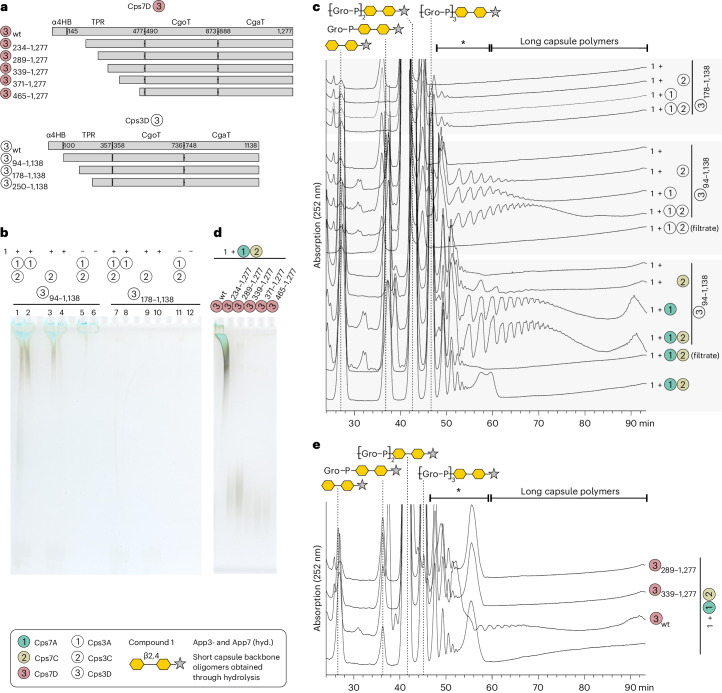
The role of the TPR domain in Cps3D and Cps7D. **a**, N-terminal truncations of Cps7D (top) and Cps3D (bottom) generated herein. See also Extended Data Fig. 8 and Supplementary Fig. 12. **b**,**c**, Compound **1** was elongated with CpsA and CpsC, the enzymes were removed by filtration and the filtrate was used as acceptor with proteins as indicated. None of the TPR truncations was able to elongate poly(Gro3P). However, Cps3D_94–1138_, in which only α4HB was truncated, retained this ability (**b**) and even produced long polymers when Cps3A/C or Cps7A/C were present (**c**). **d**,**e**, Elongation of the products of CpsA/C by the enzymes as indicated and subsequent analysis using PAGE (**d**) and HPLC-AEC (**e**). TPR truncations were unable to elongate poly(Gro3P). Species eluting between app. 48 and 60 min (marked with asterisk) can comprise products of CpsC and (short) products of CpsD (panel **c** and **e**; see also Extended Data Fig. 4d). wt, wildtype; hyd., hydrolyzed. Source data

**Extended Data Fig. 8:**
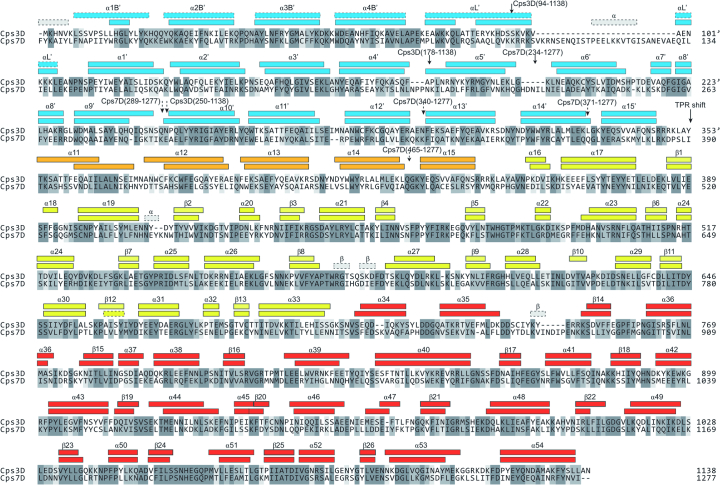
Comparison of secondary structural elements of Cps3D and Cps7D based on a superposition of the dimeric Cps3D crystal structure with the monomeric Alphafold model of Cps7D. Coloured secondary structural elements are labelled according to the crystal structure and the predicted N-terminus (α1B-α4B, αL) of Cps3D. Compared to Cps3D, Cps7D_AF_ has an extended TPR domain that comprises regions from both protomers of the Cps3D dimer, which is the molecular reason for the monomeric state of Cps7D. The first 15 α-helices of Cps7D_AF_’s TPR domain superimpose with the corresponding α-helices from the TPR domain of the neighbouring protomer 2 of the Cps3D dimer (α1’-α15’, light blue). In contrast, α16-α20 of Cps7D’s TPR domain superimpose with α11-α15 (orange) of the equivalent protomer 1 of the Cps3D dimer. An arrow labeled ‘TPR shift’ indicates that α1’-α15’ (light blue) and α11-α15 (orange) belong to two different protomers of the Cps3D structure. Surface representation of full-length Cps3D/Cps7D_AF_ and their truncations are presented in Fig. 5 and Supplementary Fig. 14. The TPR(‘) domain is required for the elongation of poly(Gro3P) (Fig. 6) and in close vicinity to CgaT(NT) (Fig. 5a, b), which transfers the first galactose onto poly(Gro3P). TPR(‘) and CgaT(NT) interact through many structural elements, that are more closely described in Supplementary Fig 14.

TPRs mediate protein–protein interactions and the majority of TagF-like capsule polymerases have a TPR domain at their N- or C-terminus^14^. To interrogate the role of the TPR domain, we constructed iterative N-terminal truncations of Cps3D and Cps7D (Fig. 6a, Extended Data Fig. 8 and Supplementary Figs. 1b and 14) and tested their activity (Supplementary Fig. 12c) using short capsule oligomers (Supplementary Fig. 15) as acceptor substrate. Truncating the N-terminal α-helical bundle (Cps3D_94–1138_) of Cps3D did not affect the enzyme’s ability to elongate the acceptors (Supplementary Fig. 12c). Neither did the removal of about 30% of the TPR domain of either enzyme (Cps3D_178–1138_ and Cps7D_234–1277_). Interestingly, construct Cps3D_250–1138_, lacking α1–α9 of the TPR domain, was inactive, while the corresponding Cps7D truncation Cps7D_289–1277_ could still produce polymer. However, Cps7D_339–1277_, missing α1–α12, was completely inactive (as were further truncated constructs Cps7D_371–1277_ and Cps7D_465–1277_), eluted in the void volume during size exclusion chromatography and appeared degraded in PAGE (Supplementary Figs. 1 and 12b,c). These observations might indicate aggregation, improper folding or instability as reasons for inactivity. Importantly, all Cps3D truncations were still able to assemble a dimer (Supplementary Fig. 12b). Interestingly, the TPR domain appears to be in contact with the N-terminal region of CgaT (CgaT-NT) in all active and stable constructs (Cps3D_94–1138_, Cps3D_178–1138_, Cps7D_234–1277_ and Cps7D_289–1277_; Supplementary Fig. 14).

Next, we analyzed the elongation mechanism of the active Cps3D and Cps7D truncations by varying the donor to acceptor (d/a) ratio (Supplementary Fig. 12d). Distributive enzymes would generate a product pool with low dispersity, corresponding in size to the adjusted d/a ratio, while processive enzymes would generate heterogeneous product pools with increased dispersity and skewed towards longer chains^27^. Wild-type Cps3D and Cps7D enzymes were processive, and long chains were already produced at a low d/a ratio. Removal of α4HB did not change the product profile (Cps3D_94–1138_). In contrast, all active TPR truncations (Cps3D_178–1138_, Cps7D_234–1277_, and Cps7D_289–1277_) produced shorter and less disperse products that correlated better with the d/a ratio, suggesting a more distributive mechanism (Supplementary Fig. 12d).

The AlphaFold model of the α4HB suggests a strong positive charge (Fig. 4d). Furthermore, in the Cps3D crystal structure, the N-terminal part of TPR(′) and the N-terminal domain of CgaT, which is the domain in GT-B fold enzymes that is typically involved in acceptor recognition^23^, are in close vicinity (Fig. 5a). To interrogate if the α4HB or the TPR domain might be required to use poly(Gro3P) as acceptor, we tested whether the truncated but still active constructs would elongate the products synthesized in CpsA/C reactions. Interestingly, Cps3D_94–1138_, lacking only the α4HB, could elongate poly(Gro3P), could be stimulated to produce more product in the presence of Cps3A and Cps3C (Fig. 6b, compare lane 1–3 with lane 4), and was able to generate long chains through stimulation by Cps3A/C and Cps7A/C, as detected by HPLC–AEC (Fig. 6c). In contrast, none of the active TPR truncations of Cps3D and Cps7D retained the ability to markedly elongate the products of CpsA/C (Fig. 6b–e), even though they were still able to elongate capsule fragments (Supplementary Fig. 12c,d).

## Discussion

**Extended Data Fig. 9:**
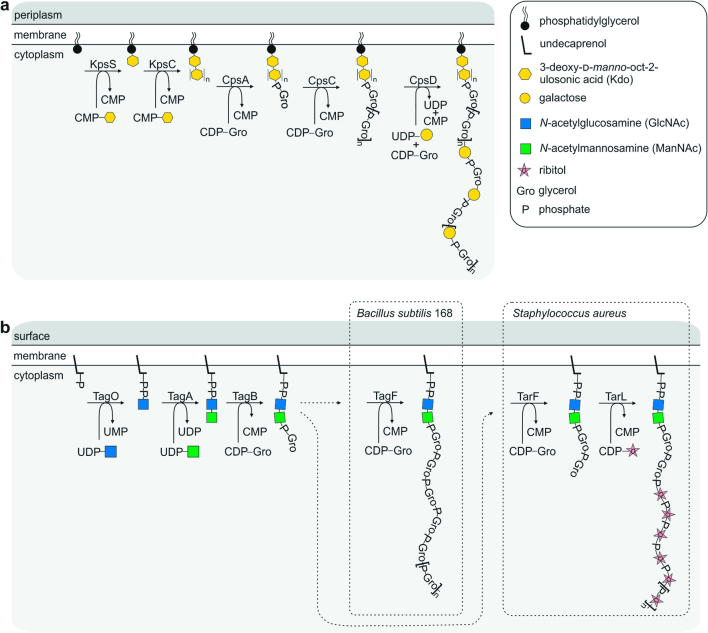
Comparison between Gram-negative group 2 capsule biosynthesis and Gram-positive WTA biosynthesis. **a**, Schematic showing the synthesis of Gram-negative capsule polymer at the cytoplasmic side of the inner membrane. KpsS/C generate the conserved glycolipid, which is linked to the capsule polymer (assembled by CpsD) via a poly(Gro3P)-linker assembled by CpsA/C. **b**, Schematic showing the biosynthesis of Gram-positive WTA of type I (reviewed in Brown et al.^19^ and Sewell et al.^71^, pathways for type II-IV are uncharacterized). In analogy to poly(Kdo), a conserved priming glycolipid is assembled on the cytoplasmic side of the bacterial cell membrane, but instead of (lyso)phosphatidylglycerol, membrane anchored undecaprenyl phosphate is used as lipid carrier^19^. The enzymes TagO/TarO and TagA/TarA create a ManNac(β1-4)GlcNAc discaccharide, which serves as acceptor for the priming glycerophosphotransferase TagB^72,73^. In analogy to Cps7A, TagB transfers the first GroP residue. The resulting product is conserved and used by all WTA I biosynthesis pathways investigated so far. Subsequently, the biosynthesis pathways diverge depending on the pathogen. In *B. subtilis* 168, the WTA type I polymerase TagF generates a chain of up to 40–60 Gro3P moieties. Unlike the product of CpsC (**a**), this structure represents the final WTA I polymer and is not further elongated by a polymerase like CpsD. In contrast, and in better analogy to the reactions catalyzed by CpsC and CpsD, the glycerophosphotransferase TarF from *S. aureus* only extends the product of TagB by one or a few Gro3P moieties, before the WTA I polymerase TarL adds the structurally distinct WTA I polymer (poly(ribitol-phosphate)), similar to CpsD in App (although CpsD generates a WTA type II structure).

In this study, we identified transition transferases that connect the conserved glycolipid anchor to the serotype-specific capsule polymer in Gram-negative group 2 capsule biosynthesis systems^1,2^ (Extended Data Fig. 9a). Rather than generating a structurally distinct polymer (such as CpsA/C), it has been hypothesized that transition transferases would add the first residue or repeating unit of the capsule polymer to poly(Kdo), which would then serve as priming acceptor for the capsule polymerase^4^. This hypothesis was in agreement with the activity of transition transferases from WTA^19^ and O-antigen^28^ biosynthesis systems and might still be correct for certain group 2 capsule expressing strains such as *E. coli* K1 and K5, for which the capsule polysaccharide was found to be connected directly to poly(Kdo), indicating the existence of a single transition transferase that adds one component of the polymer.

**Extended Data Fig. 10:**
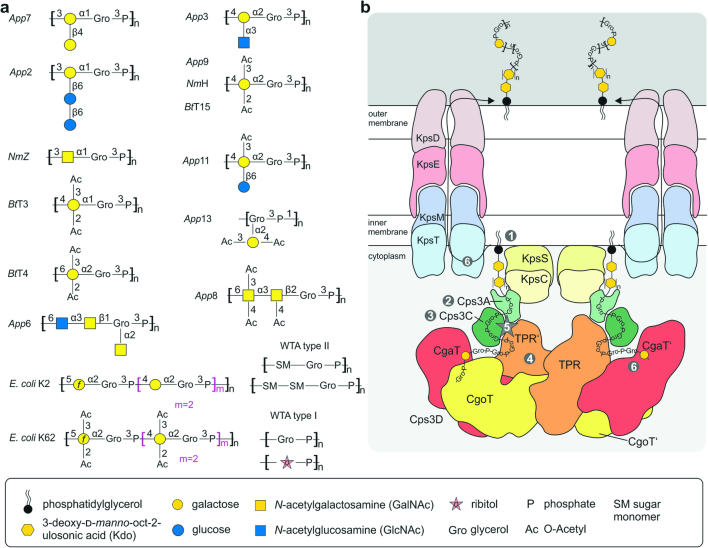
Revised working model for the biosynthesis of WTA-like group 2 capsule polymers. **a**, Characterized capsule structures of strains harbouring CpsA and CpsC homologs. **b**, Revised working model for group 2 capsule biosynthesis for WTA-like polymers: (1) KpsS and KpsC assemble the conserved glycolipid consisting of (lyso)phosphtidylglycerol and poly(Kdo), which is hypothesized to be an export signal^2^ and might already be associated with the ABC-transporter. (2) CpsA transfers the first Gro3P residue onto poly(Kdo), creating a primer for (3) the assembly of the WTA-type I-like poly(Gro3P) linker catalysed by CpsC. (4) The TPR domain enables CpsD to utilize poly(Gro3P) as primer for capsule polymerization and is required for processive elongation. (5) CpsA and CpsC stimulate CpsD to produce more and longer products. (6) The concave surface of CpsD and the predicted location of α4HB (see Fig. 4) above the concave surface suggest an orientation in which the active centres of the polymerase (CpsD) face the cytoplasmic membrane. Similar to the dimeric Hib capsule polymerase Bcs3^35^, the distance between the two active centres (app. 90 Å) might allow an enzymatic assembly in which each of the two chains generated by the Cps3D dimer is exported by one ABC transporter complex (diameter of 60–70 Å^74,75^). It is of note that interactions between components of the App biosynthesis system have not been experimentally proven whereas studies in *E. coli* present evidence for the formation of a multi-protein complex^76,77^. Moreover, the processive finalization of a polymer by a multi-enzyme system coupled to ATP-dependent translocation is hypothesized as efficient scenario for capsule expression.

Homologs of CpsA and CpsC could be identified in several important pathogens *(Actinobacillus* sp., *Bibersteinia* sp., *Pseudomonas* sp., *Avibacterium* sp., *E. coli*, Nm and *Shigella flexneri)*. Polymers generated by strains with characterized capsule polymer (Extended Data Fig. 10a) are reminiscent of Gram-positive WTA of type II^19^ and are assembled by polymerases with similarity to WTA biosynthesis enzymes^14^. In line with that, the transition transferases and their products also share similarity to WTA biosynthesis systems. The poly(Gro3P) generated by CpsA/C is identical to and reminiscent of two prominent WTA type I structures (compare Extended Data Fig. 9a,b): (1) the archetypal poly(Gro3P) WTA type I from, for example, *Bacillus subtilis* 168 and *Staphylococcus epidermidis* and (2) the oligo(Gro3P) linker connecting the poly(ribitol-phosphate) WTA type I from, for example, *Staphylococcus aureus* to the conserved priming glycolipid utilized in WTA I biosynthesis^19^. Bioinformatics data (BLAST^29^/HMMER^30^) revealed that the priming transition transferases CpsA (capsule biosynthesis) and TagB (WTA biosynthesis) share sequence identity and belong to the CDP-glycerol:poly(glycerolphosphate) glycerophosphotransferase family (Pfam family PF04464). The transition transferase TarF and the WTA polymerase TagF also belong to this family. However, unlike TarF and TagF, CpsC belongs to the LicD protein family (Pfam family PF04991), a subgroup of the nucleotidyltransferase (NTase) superfamily^31^ that use CDP conjugates as substrates^32^ and have, to the best of our knowledge, not been described as polyol-phosphate polymerases before.

Our structural and biochemical data support a mechanistic model for CpsD, in which CgaT transfers a galactose from UDP-Gal to a terminal glycerol (OH1 for App7 and OH2 for App3), and CgoT transfers a Gro3P from CDP-Gro to the 3 (App7) or 4 (App3) position of a terminal galactose. The dimeric structural arrangement of Cps3D supports the presence of two reaction centers inside the concave side, one in each protomer, allowing two chains to be elongated by two dyads of enzymes, CgaT/CgoT and CgaT′/CgoT′. It is unlikely that intermolecular reaction centers are formed in Cps3D (for example, CgaT′/CgoT and CgaT/CgoT′), as Cps7D is monomeric. Structural information is available for two monomeric^33,34^ and one dimeric^35^ group 2 capsule polymerase. Bcs3^35^, the dimeric capsule polymerase from *H. influenzae* type b, combines an SH3b-like polymer binding domain, a ribofuranosyltransferase (CriT), a phosphatase (CrpP) and a ribitol-phosphate transferase (CroT) homologous to CgoT, but lacks a TPR domain. In contrast to Cps3D, the two reaction centers comprise the SH3b-like domain and triads of enzymes originating from both protomers (CriT/CrpP′/CroT′ and CriT′/CrpP/CroT).

CpsD constructs with N-terminally truncated TPR(′) domain are unable to elongate poly(Gro3P), and although they can still elongate capsule polymer fragments, processive chain elongation is severely hampered. It is yet unclear how the N-terminal part of the TPR domain mediates processivity and elongation of poly(Gro3P), if it contains a binding site for poly(Gro3P) or capsule polymer, or if it modulates the active center in a way that the substrates are accepted in a more complex process, for example, facilitated or mediated by CpsA/C. It is interesting in this context that Cps3D and Cps7D show a considerable flexibility with regard to their acceptor substrate^14^ and that their domains can be combined in *trans* to assemble new polymers with non-App3/7 linkages^15^. Both TPR–polymer^36,37^ and TPR–protein^36–38^ interactions have been reported in synthase-dependent capsule biosynthesis systems, in which the TPR domain is sometimes expressed separately from the catalytic domain^1,39^. In contrast, TPR and catalytic modules are part of the same enzyme in all homologs of CpsD analyzed so far^14^.

We demonstrate that the transition transferase (CpsA, to a lesser extent CpsC) stimulates the polymerases acting downstream during biosynthesis (CpsC and CpsD) to produce more polymer and longer chains. It is unclear if this effect is mediated by the TPR domain of CpsD, as TPR truncations do not elongate poly(Gro3P). Interestingly, Cps7A_AF_ is predicted to contain a long electropositive C-terminal α-helix and Cps7C_AF_ is predicted to have an N-terminal TPR domain. How these structural elements are organized in space and time is intriguing and remains unknown. An activating effect has also been reported for a synthase-dependent system, in which the C-terminal TPR region of PgaA from *E. coli* interacts with the de-*N*-acetylase domain of PgaB, increasing its deacetylase and hydrolase activity^36^. How could the stimulating function be rationalized in the context of capsule biosynthesis? In the living cell, enzymes are often grouped in supramolecular assemblies, which promote higher performance of individual catalysts, enhance the effective concentration of substrates and products, and increase the overall reaction efficiency^40,41^. Capsule expression is an energy-intensive process for a bacterium. Thus, the biosynthesis system might have evolved in a way that, as soon as an initiating step (for example, by CpsA) is taken, all downstream enzymes commit to completing a polymer with high efficacy rather than producing many polymers that might be too short to fulfill their biological function (for example, protect the pathogen). The processivity observed for CpsD is in agreement with this hypothesis. Based on our findings, we propose a revised working model for group 2 capsule biosynthesis (Extended Data Fig. 10b).

The identification of transition transferases, the importance of the TPR domain for connecting poly(Kdo) and capsule polymer, the stimulating effect and the structure of a multi-enzyme polymerase that generates WTA type II-like polymers represent important milestones toward understanding and exploiting Gram-negative capsule and Gram-positive WTA biosynthesis.

## Methods

### General cloning

Primers and enzymes used in this study are presented in Supplementary Table 1. The full primer sequences are presented in Supplementary Table 2.

The genes *cps7A*/*cps7C* (ACE62294.1/ACE62292.1), *cps3A*/*cps3C* (ABU63689.1/UKH44265.1), *csxC* (ATG32051.1) and *csaD* (CAM07516.1) were cloned from genomic DNA of *Actinobacillus pleuropneumoniae* serotype 7 (App7) strain AP76 (GenBank accession number CP001091.1), lysate of App3 strain S1421 (GenBank® accession number EU090171.1 or CP031874.1), plasmid *pHC19* (ref. ^11^) and lysate of Nm serogroup A strain Z2491 (AL157959.1), respectively. The resulting PCR products were cloned into *pMBP-PreScission-S3N10-csxA-His*_*6*_ *(tac)*^35^ via BamHI/XhoI, replacing *csxA*. For CsaD, a thrombin (instead of a PreScission) cleavage site was introduced, resulting in MBP-S_3_N_10_-Thrombin-CsaD-His_6_.

To generate the crystallization construct MBP-PreScission-S_3_N_10_-cps3D-His_6_, a PreScission cleavage site was inserted into the modified p-Mal-c (New England BioLabs) vector *pMBP-S3N10-cps3D-His*_*6*_ *(tac)*^14^.

The DNA fragments encoding Cps1C (AWG96005.1), CsxB (ATG32052.1) and all Cps3D truncations were amplified from heat-inactivated lysate of App1 strain 4074 (GenBank accession number CP029003.1), plasmid *pHC19* (ref. ^11^) or *pMBP-PreScission-S3N10-cps3D-His*_*6*_, respectively. The resulting PCR products were cloned into *pMBP-PreScission-S3N10-cps3B-His*_6_ (ref. ^35^) via restriction-free cloning^42^, replacing *cps3B*.

To introduce the Cps7D truncations, the respective coding sequences were amplified by PCR using *pMBP-cps7D-His*_*6*_ (ref. ^14^) as template and the resulting PCR products were cloned into *pMBP-S*_*3*_*N*_*10*_*-csxA-His*_*6*_ *(tac)*^11^ by restriction-free cloning^42^, replacing *csxA*.

Single amino acid mutants of Cps7A, Cps7C and Cps3D were introduced according to Liu et al.^43^ or by using the Q5 Site-Directed Mutagenesis Kit (New England BioLabs) according to the manufacturer’s guidelines.

The *cps1A* gene was amplified from lysate of App1 strain 4074 (GenBank accession number CP029003.1) using primers CL86 and CL98. The resulting PCR product was cloned into *pMBP-csxA-His*_*6*_ (ref. ^11^) via NdeI/BamHI, thereby replacing *csxA*, but differed from the published sequence (AWG96007.1). Sequence alterations due to mismatched primers were corrected using CL100 and CL101 according to the Q5 Site-Directed Mutagenesis Kit (New England BioLabs). The sequence of Cps1A identified herein is shown in Supplementary Fig. 16.

*pΔN15-cslA-His*_*6*_ was obtained by using the Q5 Site-Directed Mutagenesis Kit (New England BioLabs) according to the manufacturer’s guidelines and *pMBP-S*_*3*_*N*_*10*_*-cslA-His*_*6*_ (ref. ^10^) as template.

### Expression and purification of recombinant proteins

The expression and purification of recombinant proteins was performed according to Cifuente et al.^35^ using a combination of affinity chromatography and size exclusion chromatography in 50 mM Tris pH 8.0, 300–500 mM NaCl, using a linear or step-wise gradient of 500 mM imidazole as indicated in Supplementary Fig. 8.

### Analytical size exclusion chromatography

The size exclusion chromatography column (Superdex 200 Increase 10/300 GL (Cytiva)) was equilibrated using a gel filtration marker kit for protein molecular weights of 29,000–700,000 Da (Sigma) according to the manufacturer’s guidelines. To determine the apparent molecular weight, 0.8 mg of each protein of interest was loaded onto the column.

### Polyacrylamide gel electrophoresis

For the analysis of capsule polymers, high-percentage (25% or, if indicated, 15%) PAGE and subsequent Alcian blue/silver staining was performed as previously described^20^. Alcian blue/silver-stained gels were scanned on an Amersham Imager 680. For the visualization of protein-containing samples, 2 µg of each indicated enzyme was analyzed by SDS–PAGE^11^.

### Synthesis and purification of compounds 1 and 2

Compounds 1 and 2 were generated according to Ovchinnikova et al.^7^. A 7-ml reaction mixture was prepared containing 50 mM HEPES, pH 8.0, 10 mM MgCl_2_, 2 mM Kdo, 5 mM CTP, 1.4 mM acceptor, 0.1 mM purified KpsC-N and 3.4 µM KdsB. The reaction was incubated at 30 °C for 40 min, and a 2 µl aliquot was analyzed by thin-layer chromatography (TLC). The TLC plate was developed in freshly prepared chloroform–methanol–water–acetic acid mixture (25:15:4:2, v/v), and the products were visualized using a hand-held UV lamp. Approximately 50% conversion was achieved. Precipitated proteins were removed by centrifugation, and the reaction mixture was loaded on two joined Sep-Pak C18 Plus cartridges equilibrated in water. After the sample was loaded, the cartridges were disconnected and treated separately. The product and unreacted acceptor were washed with water and eluted with a 50% can–water mixture. The sample was dried using a SpeedVac. To achieve full conversion of the unreacted acceptor, this mixture was used again as substrate in the same 7-ml reaction. After a 1 h incubation, nearly full conversion of acceptor was seen by TLC. The product was purified using two joint Sep-Pak C18 Plus cartridges followed by gel permeation chromatography on a Sephadex G-25 column (1.5 × 75 cm) in 100 mM ammonium acetate. The 1-ml fractions were concentrated in a SpeedVac to ~200 μl and analyzed by TLC. Fractions containing the product were pulled and lyophilized. The identity of the Kdo-(2,4)-Kdo-methoxibenzamide was confirmed by ^1^H and ^1^H,^13^C HSQC NMR.

### Activity assay for transition transferases

Activity assays for candidate transition transferases (and their mutants) were carried out with 2 µM enzyme at 37 °C in a total volume of 20–70 µl assay buffer (20 mM Tris pH 8.0, 10 mM MgCl_2_, 1 mM dithriothreitol (DTT)) containing 0.5–5 mM of each required donor substrate (racemic CDP-Gro (Sigma), UDP-Gal (Roche), UDP-GlcNAc (Roche) and UDP-GlcA (Sigma)) and 0.2–1.25 mM compound **1**/**2** or 5 µM alternative acceptor 4-methylumbelliferyl (4-MU)-Sia, 4-MU-Sia-Gal-Sia and 1,2-diamino-4,5-methylene-oxybenzene (DMB)-(Sia)_3_). After 3 h incubation or overnight incubation, samples containing 5–18 nmol compound **1** or **2** or 25 pmol fluorescence-labeled acceptor were analyzed by HPLC–AEC^20^ using a Prominence UFLC-XR (Shimadzu) at 50 °C and 0.6 ml min^−1^. Data were collected using LCSolution version 1.25 SP4 (Shimadzu). Elution was achieved using a linear gradient from (1) 0–50% mobile phase 2 (M_2_; 20 mM Tris pH 8.0 and 1 M NaCl) over 44 min (gradient 1) or (2) 0–100% M_2_ over 88 min (gradient 2). Elution profiles were monitored using a UV detector (SPD-20AV) (absorbance at 252 nm) or a fluorescence detector (RF-10A XL, 4-MU: excitation 315 nm/emission 375 nm; DMB: excitation 372 nm/emission 456 nm), as indicated. The AEC column (Dionex CarboPac PA100) was calibrated with compounds **1**, **2** and **3** at various points during the project, and if relevant, the respective chromatograms are either shown as standard or the elution time of the respective compound is indicated by a dotted line.

For the in situ synthesis of the donor substrate CDP-Gro (GCT reaction), 2–3 µM glycerol-3-phosphate cytidylyltransferase (GCT; Cps7B or Cps3B) was incubated at 37 °C in a volume of 60–100 µl of assay buffer containing 15 mM CTP [Sigma] and either *sn*-gylcerol-3-phosphate (Gro3P; Sigma), *sn*-glycerol-1-phosphate (Sigma) or racemic glycerol-phosphate (Sigma), as indicated. After 2 h incubation, GCT reaction was added to the reaction mixture for transition transferases, resulting in a concentration of approximately 1–5 mM CDP-Gro. For in situ synthesis of the donor substrate UDP-ManNAc, 1 µM UDP-*N*-acetyl-d-glucosamine-2-epimerase^9^ (CsaA) was added to the reaction mixture containing UDP-GlcNAc and CsaD.

For time course experiments, enzymatic reactions were carried out in a total volume of 100 µl. Aliquots were taken at defined time points (30, 60, 120, 180 and 360 min), frozen in liquid nitrogen and thawed before analysis by HPLC–AEC coupled to UV detection using gradient 1.

### Synthesis and purification of compounds 3–5

For the synthesis of compound **3**, 2 µM Cps7A was incubated overnight at 37 °C in a total volume of 50 µl assay buffer containing 4 µM GCT, 1 mM compound **1** and 2 mM CTP and Gro3P. A sample containing 4.5 nmol acceptor was analyzed by HPLC–AEC (UV detection, gradient 1). The reaction was scaled up to yield 1 mg of compound **3**, resulting in a total reaction volume of 1.4 ml (28× volume of test reaction). The reaction product was purified by AEC using a Mono Q 5/50 GL column (GE Healthcare) and a combination of linear gradients (0–150 mM NaCl over three column volumes (CV), 150–240 mM NaCl over 3 CV, 240–290 mM NaCl over 3 CV and 290–500 mM NaCl over 30 CV). Product-containing fractions were pooled, dialyzed against water (ZelluTrans, 1,000 molecular weight cutoff; Roth) and freeze-dried.

For the synthesis of compounds **4** and **5**, Cps7A and Cps7C were incubated overnight at 37 °C in a total volume of 20 µl assay buffer containing 2.8 µM GCT, 1 mM compound **1** and 2 or 3 mM CTP and Gro3P, resulting in d/a ratios of 2:1 or 3:1 as indicated. Samples containing 4.5 nmol of product were analyzed by HPLC–AEC (UV detection, gradient 2). The reaction was upscaled with a d/a ratio of 3:1 to yield a theoretical 1.5 mg of compound **4**, resulting in a total volume of 2.1 ml. The products were then purified by AEC as described for compound **3**. Acceptor-containing fractions were analyzed by HPLC–AEC, pooled as shown in Supplementary Fig. 3h–j and dialyzed against water.

### NMR analysis

Spectra were measured on a 600-MHz Bruker Avance III HD equipped with a ^1^H/^13^C/^15^N/^31^P QXI probe at 298 K according to Cifuente et al.^35^. Typically, samples were dissolved in 500 μl D_2_O (100.0 atom%; Armar Chemicals) and measured in a 5-mm NMR standard tube. ^1^H 1D spectra were recorded with eight transients and a recycle delay of 10 s. ^31^P 1D spectra were acquired with a recycle delay of 3 s and 352 transients. Standard ^1^H–^13^C HSQC experiments from the Bruker library (hsqcedetgpsisp2.2) were measured with 32 scans, 2,048 × 230 points and a recycle delay of 1.5 s. ^1^H–^1^H TOCSY spectra were recorded with four scans, 2,048 × 360 points, a recycle delay of 1.5 s and a mixing time of either 80 ms or 12 ms. ^1^H–^13^C HMBC spectra (Bruker pulse sequence: hmbclpndqf) were acquired with either 64 or 96 scans, 4,096 × 380 points and a recycle delay of 1.5 s. ^1^H–^31^P HMBC spectra (Bruker pulse sequence: hmbcgpndqf) were recorded with 32 scans, 4,096 × 120 points and a recycle delay of 1.5 s. All spectra were processed using Topspin 3.6.1 (Bruker Biospin) and analyzed using Sparky 3.111 and 3.115 (T. D. Goddard and D. G. Kneller, SPARKY 3, University of California, San Francisco, CA, United States). Proton chemical shifts were calibrated to 2,2-dimethyl-2-silapentane-5-sulfonic acid using an external sample of 2 mM sucrose and 0.5 mM 2,2-dimethyl-2-silapentane-5-sulfonic acid in H_2_O/D_2_O (Bruker). Indirect chemical shift referencing was applied to ^13^C and ^31^P according to the International Union for Pure and Applied Biophysics (IUPAB) using the chemical shift referencing ratios of 0.251449530 and 0.404808636 (ref. ^44^).

### Mass spectrometry

Cps7A and Cps7A/C products were dissolved in 5 µl water and diluted 1:10 with water, and 1 µl thereof was mixed with 1 µl DHB (2,5-dihydroxybenzoic acid solution, 5 mg ml^−1^ in 70% methanol) and spotted on a metal target plate. MS was performed by matrix-assisted laser desorption/ionization (MALDI) time-of-flight (TOF) analysis in negative-ion reflector mode using a 5800 MALDI TOF/TOF (AB Sciex). Spectra were processed with Data Explorer Software V4.8 applying ‘Advanced Baseline Correction’.

### Synthesis and purification of compounds 6, 7 and 8

Detailed information about the synthesis of compounds **6** and **7** is presented in a separate file (Supplementary Note at the end of Supplementary Information), and a synthesis scheme is given in Supplementary Fig. 5. Compound **8** was synthesized according to established methods^21,22^.

### App3 and App7 polymer backbones and oligomers

For the synthesis of 50 mg App3 and App7 polymer backbone, 2 µM Cps7D or Cps3D were incubated in assay buffer containing 2–3 µM GCT and 6 mM donor substrate (CTP, Gro3P, UDP-Gal) at 37 °C overnight. The reaction progress was monitored by HPLC–AEC^15,20^, and polymer was purified by preparative AEC^13^. Elution fractions were analyzed by high-percentage (15%) PAGE^20^, and polymer-containing fractions were pooled, dialyzed against water (10,000 molecular weight cutoff, Zellutrans; Roth) and freeze-dried.

For the generation of oligomers, acidic hydrolysis of the App7 polymer backbone (2.5 mg ml^−1^) was tested in 25 mM trifluoroacetic acid (Sigma) at 70 °C. Samples of 10 µl were collected at time points as indicated, mixed with 10 µl of 2 M sucrose and analyzed by high-percentage (25%) PAGE^20^. The reaction was scaled up to hydrolyze 30 mg of App7 polymer in a total volume of 12 ml. After 33 min of hydrolysis, the reaction was neutralized with NaOH. Oligomers were purified by AEC^13^ and visualized by Alcian blue/silver staining after high-percentage (25%) PAGE^20^. Oligomer-containing fractions were divided into four pools, dialyzed against water (Zellutrans, 1,000 molecular weight cutoff; Roth) and then freeze-dried. To remove terminal phosphomonoesters, each pool was dissolved in 37,5 ml buffer (50 mM Tris pH 7.0, 100 mM NaCl and 10 mM MgCl_2_) containing 375 U alkaline phosphatase (Quick CIP; New England BioLabs). After 1 h at 37 °C, another 100 U of alkaline phosphatase was added and incubation was continued for 1 h at 37 °C. Removal of alkaline phosphatase was achieved using Amicon centrifugal devices (50,000 molecular weight cutoff; Merck). Each oligomer pool was dialyzed against water (Zellutrans, 1,000 molecular weight cutoff; Roth) and freeze-dried. App3 oligomers were obtained using a similar protocol with the following change: App3 polymer backbone (2.5 mg ml^−1^) was hydrolyzed at 50 °C for 8 h in 25 mM trifluoroacetic acid.

### Elongation of various acceptors by CpsD constructs

Compounds **3**–**5**, compound **8**, or the products of a CpsA/C reaction (as described above, if indicated including removal of CpsA/C by filtration, 30 kDa molecular weight cutoff), were used as acceptor substrates for CpsD constructs at a concentration of 0.1–0.5 mM in a total volume of 20–70 µl assay buffer (20 mM Tris pH 8.0, 10 mM MgCl_2_ and 1 mM DTT). Donor substrate (CDP-Gro from GCT reaction, UDP-Gal) was added at a 5–50× excess, depending on whether single or multiple transfers should be achieved. Then, 10–100 nM of CpsD construct were added for polymer production with minimized de novo activity, and 2 µM single action mutants of CpsD were used, for example, for the stepwise elongation of an acceptor. CpsA/C or its mutants were added at a concentration of 2 µM. After incubation for 3–24 h at 37 °C, samples containing 2–5 nmol product were analyzed by HPLC–AEC (UV detection, gradient 2) or PAGE followed by Alcian-blue/silver staining^20^.

Elongation of compounds **6** and **7** was carried out using 25–50 nM Cps7D at 37 °C in a total volume of 20 µl assay buffer containing 1 µM GCT, 5 mM CTP, 5 mM Gro3P and 5 mM UDP-Gal, and 50 or 250 µM acceptor resulting in the d/a ratios of 100:1 and 20:1. After overnight incubation, a sample was analyzed by PAGE (25%) and HPLC–AEC coupled to fluorescence detection (4,4-difluoro-4-bora-3*a*,4*a*-diaza-*s*-indacene (BODIPY): excitation 503 nm/emission 515 nm) using a −2 curved gradient from 0% to 37% M_2_ (20 mM Tris pH 8.0, 1 M NaCl) over 4 min, followed by a linear gradient from 37% to 65% M_2_ over 33 min (gradient 3).

For the step-wise elongation of compound **7**, 2 µM Cps7D-R1123A was incubated at 37 °C in a total volume of 1.5 ml assay buffer containing 2 µM GCT, 5 µM acceptor (**7**) and 5 mM of each substrate (CTP, Gro3P). Complete modification of compound **7** was monitored by HPLC–AEC coupled to fluorescence detection using a −2 curved gradient from 0% to 40% M_2_ (20 mM Tris pH 8.0 and 1 M NaCl) over 4 min followed by a linear gradient from 40% to 60% M_2_ over 33 min (gradient 4). After 2 h incubation, Cps7D-R1123A was removed from the reaction using Amicon centrifugal devices (30,000 molecular weight cutoff). The enzyme-free filtrate was supplemented with 1.7 µM Cps7D-H743A, 1 mM UDP-Gal and 1 mM DTT, incubated at 37 °C and analyzed again by HPLC–AEC to check for complete modification, before removing Cps7D-H743A and starting a new cycle. After each extension, aliquots were taken (10 µl) to test the suitability of the generated molecules as acceptor substrate for full-length Cps7D (25 nM Cps7D, 37 °C, 20 µl assay buffer containing 1 µM GCT and 5 mM of each donor substrate (CTP, Gro3P and UDP-Gal)). After overnight incubation, samples containing 25 pmol acceptor were analyzed by HPLC–AEC.

To analyze the elongation mechanism of CpsD constructs, 5 µM, 50 µM and 500 µM hydrolyzed App3 or App7 polymer backbone was incubated with 25–100 nM CpsD, 1 µM GCT and 5 mM substrate (CTP, Gro3P and UDP-Gal). After overnight incubation, samples were analyzed by high-percentage (25%) PAGE.

### Bioinformatics

Alignments were performed using Clustal Omega^45^, homology modeling and structure prediction was performed using Phyre2^46^ and AlphaFold^24^.

### Cps3D crystallization and data collection

For our studies, we used a Cps3D construct comprising from the N- to the C-terminus: (1) an MBP, (2) a PreScission protease cleavage site (LEVLFQ/GP), (3) the full-length version of Cps3D (residues 2–1,138) and (4) a 6× histidine tag (MBP-Cps3D_2-1138_-His_6_; Supplementary Figs. 1 and 8). The unliganded form of Cps3D was crystallized by mixing 0.25 μl of a MBP-Cps3D_11-1138_-His_6_ solution (12.0 mg ml^−1^ in 10 mM Tris pH 8.0, 300 mM NaCl and 1 mM DTT) with 0.25 μl 1.6 M magnesium sulfate heptahydrate, 0.1 M MES pH 6.5 (structure I and II commercial screening, Molecular Dimensions). Crystals were plunge frozen under liquid nitrogen. A complete X-ray diffraction dataset was collected at the beamline i24 using a beam transmission equal to 100% (Diamond Light Source Synchrotron). Cps3D crystallized in the *P* 3 2 1 space group with one molecule in the asymmetric unit. The structure was solved at a maximum resolution of 3.0 Å (Supplementary Table 4). The dataset was integrated and scaled with XDS following standard procedures^47^.

### Cps3D structure determination and refinement

The structure determination of Cps3D was performed by molecular replacement methods implemented in Phaser 2.8^48^ and the PHENIX suite^49^, using a Cps3D initial model generated by AlphaFold^24^. Model building was performed with the CCP4 8.0^50^ suite and Coot^51^, and refinement with phenix.refine^52^. The structure was validated with MolProbity 4.5.2^53^. Data collection and refinement statistics are presented in Supplementary Table 4. Molecular graphics and structural analysis were performed using the UCSF Chimera package^54^.

### Structural analysis and sequence alignment

Homologs for the two active domains in Cps3D were identified using DALI^55^. Structure-based sequence alignment analysis was performed using the UCSF Chimera package^54^.

### Molecular substrate docking

The substrate binding analysis and identification of the active sites have been done for CgoT and for CgaT on the basis of (1) homolog identification and placement of the substrate by homology and (2) the minimization of the substrate docked ligand using UCSF Chimera^54^. For CgoT, the modeling of CDP-glycerol in the active site was obtained structurally comparing it with the Wall Teichoic Acid polymerases TagF (mutant H444N from *S. epidermidis* RP62A, PDB entry: 3L7K). Then, a docking was performed using a tight search box to minimize the position of CDP-glycerol using AutoDock Vina. For CgaT, a similar procedure was employed. The structural comparison and superposition with BshA from *S. aureus* complexed with UDP and *N*-acetylglucosamine allowed the identification of the active site. UDP-galactose was extracted from PDB code 1A9Z and docked using a tight search box to minimize the ligand position, using AutoDock Vina^56^. All docking calculations were thoroughly inspected and confronted with the protein surfaces to verify absences of clashes.

### Statistics and reproducibility

No statistical method was used to predetermine sample size. Sample sizes were chosen according to common practices in enzyme research. The chosen sample sizes are standard for investigations of this kind and were chosen as sufficient to represent any variance present in the samples but also to be within the technically practical limits for performing the experiment. There are no quantitative data that would require additional data points for statistical analysis. Recombinant proteins were purified at least once, each purification was documented by SDS–PAGE and separate Coomassie-stained gels displaying all constructs were included in the manuscript (Supplementary Fig. 1a–c). To analyze the elongation of compound **6** and **7**, at least three reactions were performed with highly consistent results (Supplementary Fig. 6a). The crystallization construct was purified three times with similar results using the protocol shown in Supplementary Fig. 8. Activity of Cps3D constructs was tested at least two times with very similar results (Supplementary Fig. 11c). Elongation of capsule polymer fragments with inactive truncations of Cps7D and Cps3D was performed at least two times with very similar results (Supplementary Fig. 12c). To analyze the elongation mechanism of CpsD constructs, at least six reactions were performed at different d/a ratios using the active truncations of CpsD with highly consistent results (Extended Data Fig. 8). The elongation of poly(Gro3P) followed by PAGE analysis to document the activating effect was repeated five times with very similar results for the App3 biosynthesis system (Supplementary Fig. 13d) and two times with very similar results for the App7 biosynthesis system (Extended Data Fig. 5b). Scaled-up purification of App3 and App7 polymer was performed at least three times, and purified polymer was hydrolyzed and fractionated at least two times with highly similar results (Supplementary Fig. 15c,d,f). The elongation of compound **8** was performed at least three times with very similar results (Supplementary Fig. 13c). The elongation of CpsAC products with CpsD and active CpsD truncations was analyzed at least three times (Fig. 6b,d and Extended Data Fig. 4d). No data were excluded from the analyses. The experiments were not randomized, because no allocation of samples into experimental groups was required. In our experimental setup, defined enzyme variants were compared under well-controlled conditions. Accordingly, the assays performed in this study did not depend on statistical analyses of an unknown relationship but required a rational approach for activity comparison. The Investigators were not blinded to allocation during experiments and outcome assessment, because results did not require subjective judgment or interpretation.

### Reporting summary

Further information on research design is available in the Nature Portfolio Reporting Summary linked to this article.

## Online content

Any methods, additional references, Nature Portfolio reporting summaries, source data, extended data, supplementary information, acknowledgements, peer review information; details of author contributions and competing interests; and statements of data and code availability are available at 10.1038/s41589-024-01664-8.

## Supplementary information

Supplementary Information Supplementary Tables 1–5, Figs. 1–22 (Supplementary Figs. 17–22 contain source data for Supplementary Figs. 1, 6, 8, 12, 13 and 15) and note (including Supplementary Figs. 23–41).

Reporting Summary

## Source data

Source Data Fig. 6 Unprocessed gels.

Source Data Extended Data Fig. 4 Unprocessed gel.

Source Data Extended Data Fig. 5 Unprocessed gel.

## Data Availability

The atomic coordinates and structure factors have been deposited in the Protein Data Bank, accession codes 8QOY for Cps3D. Data collection and refinement statistics are presented in Supplementary Table 4. PDB IDs used in the analysis of this work include 3L7K, 3L7L,1A9Z, 4WYI, 8A0C, 3OT5, 6N1X, 3OKA, 6TVP, 1A9Z and 6KAN. Accession codes for sequences used in this study are available in Supplementary Table 1. NMR chemical shifts are presented in Supplementary Tables 3 and 5. Source data are provided with this paper.
