# Supplementary material for: Transition transferases prime bacterial capsule polymerization
Source: Nat Chem Biol. 2024 Jul 1;21(1):120–30. doi: 10.1038/s41589-024-01664-8 (PMC11666461; doi:10.1038/s41589-024-01664-8)
Supplement: Supplementary file 1 — Supplementary Tables 1–5, Figs. 1–22 (Supplementary Figs. 17–22 contain source data for Supplementary Figs. 1, 6, 8, 12, 13 and 15) and note (including Supplementary Figs. 23–41). [file 41589_2024_1664_MOESM1_ESM.pdf]

nature chemical biology

Supplementary information https://doi.org/10.1038/s41589-024-01664-8

Transition transferases prime bacterial capsule polymerization

In the format provided by the authors and unedited

TABLE OF CONTENTS

Supplementary Tables 1 - 5

Supplementary Table 1 | Enzymes and primers used in this study……………………………………………….3

Supplementary Table 2 | Primers used in this study……………………………………………………………….5

Supplementary Table 3 | Chemical shifts of the compounds 3, 4 and 5 measured at 298 K and referenced to 2,2-Dimethyl-2-silapentane-5-sulfonate sodium salt (DSS)………………………………………..7

Supplementary Table 4 | Data collection and refinement statistics……………………………………………….9

Supplementary Table 5 | ^{1}H and ^{13}C chemical shifts of the hydrolyzed App3 and App7 polymer backbones at 298 K referenced to 2,2-Dimethyl-2-silapentane-5-sulfonate sodium salt (DSS)…………10

Supplementary Figures 1 - 22

Supplementary Fig. 1 | Coomassie-stained polyacrylamide gels and schematic representations of all constructs (final pools) used in this study……………………………………………………………………………...11

Supplementary Fig. 2 | Alternative acceptors for the transition transferase Cps7A………………………..12

Supplementary Fig. 3 | Characterization of Cps7A and Cps7C and purification of their products…….13

Supplementary Fig. 4 | Mass spectrometry analysis of products synthesized by Cps7A and Cps7C….15

Supplementary Fig. 5 | Synthesis of BODIPY-labeled App7 capsule polymer fragments (compounds 6 and 7)………16

Supplementary Fig. 6 | Acceptor preference and product profile of Cps7D……………………………………17

Supplementary Fig. 7 | Comparison of App3 and App7 proteins……………………………………………………18

Supplementary Fig. 8 | Purification of the crystallization construct MBP-Cps3D$_{2-1138}$-His$_{6}$………………19

Supplementary Fig. 9 | Stereo view of Cps3D.

Supplementary Fig. 10 | Structural homologues of Cps3D…………………………………………………………...22

Supplementary Fig. 11 | Analysis of the active site of CgoT and CgaT of Cps3D and site-directed mutagenesis of crucial amino acid residues…………………………………………………………………………………23

Supplementary Fig. 12 | Biochemical characterization of wildtype and N-terminally truncated Cps3D and Cps7D constructs……24

Supplementary Fig. 13 | Transition transferases stimulate the capsule polymerase to produce more polymer and longer chains………………………………………………………………………………………………………26

Supplementary Fig. 14 | Three dimensional models of the Cps7D and Cps3D truncations generated in this study………27

Supplementary Fig. 15 | Generation of App7 capsule polymer fragments……………………………………..29

Supplementary Fig. 16 | Sequence of Cps1A as determined in this study……………………………………..31

Supplementary Fig. 17 | Source Data for Supplementary Figure 1……………………………………………….32

Supplementary Fig. 18 | Source Data for Supplementary Figure 6……33
Supplementary Fig. 19 | Source Data for Supplementary Figure 8……34
Supplementary Fig. 20 | Source Data for Supplementary Figure 12……35
Supplementary Fig. 21 | Source Data for Supplementary Figure 13……36
Supplementary Fig. 22 | Source Data for Supplementary Figure 15……37

Supplementary Note | Chemical Synthesis of compounds 6 and 7

General information……38
Synthesis of Glycerol building blocks……39
Supplementary Fig. 23 | Synthesis of glycerol building block 13……39
Synthesis of App7 monomer and dimer……40
Supplementary Fig. 24 | Synthesis of App*7* monomer 20 and dimer 25……40
Coupling of App7 with BODIPY……45
Supplementary Fig. 25 | Synthesis of App7-BODIPY……45
Supplementary Fig. 26 | Chemical structure of compound 6……45
Supplementary Fig. 27 | Chemical structure of compound 7……45
Supplementary Fig. 28 - 41 | NMR Spectra of compounds 13 – 25, 6 and 7……47

Supplementary References……87

SUPPLEMENTARY TABLES

Supplementary Table 1. Enzymes and primers used in this study. CsaA was cloned into a pET22b-based plasmid, all other constructs were cloned into a pMal-c based plasmid (see methods section).

Protein	Accession number	Identifier	Recombinant construct	MW [g/mol]	Primer
Cps1A	See Suppl. Fig. 16	4969	Cps1A-His_6	43,929	CL86/CL98 CL100/CL101
Cps1C	AWG96005.1	5545	MBP-S_3N_{10}-Prescission-Cps1C-His_6	90,130	IB87/IB88
Cps3A	ABU63689.1	5641	MBP-S_3N_{10}-Prescission-Cps3A-His_6	88,080	AB182/AB183
Cps3B	ABY70165.1	5113	Cps3B-His_6	17,544	Litschko et al. 2021[1]
Cps3C	UKH44265.1	5642	MBP-S_3N_{10}-Prescission-Cps3C-His_6	87,964	AB184/Ab185
Cps3D	KY807157	5360	MBP-S_3N_{10}-Cps3D-His_6	175,638	Litschko et al. 2018[2]
		5833	MBP-S_3N_{10}-Prescission-Cps3D-His_6	176,522	CL253/CL254
		5390	MBP-S_3N_{10}-Cps3D-H479A-His_6	175,572	CL213/CL214
		5391	MBP-S_3N_{10}-Cps3D-H609A-His_6	175,572	CL215/CL216
		5393	MBP-S_3N_{10}-Cps3D-R982A-His_6	175,553	Litschko et al. 2021[1]
		5394	MBP-S_3N_{10}-Cps3D-K987A-His_6	175,581	CL221/CL222
		6412	MBP-S_3N_{10}-Prescission-Cps3$D_{94\text{-}1138}$-His_6	165,505	JS62/CL254
		6413	MBP-S_3N_{10}-Prescission-Cps3$D_{178\text{-}1138}$-His_6	155,663	JS63/CL254
		6308	MBP-S_3N_{10}-Prescission-Cps3$D_{250\text{-}1138}$-His_6	147,408	JS61/CL254
Cps7A	ACE62294.1	5162	MBP-S_3N_{10}-Prescission-Cps7A-His_6	88,234	AB124/AB125
		5543	MBP-S3N10-Prescission-Cps7A-Y94A-His_6	88,142	AB157/AB158
		5567	MBP-S_3N_{10}-Prescission-Cps7A-H198A-His_6	88,168	AB163/AB164
		5568	MBP-S_3N_{10}-Prescission-Cps7A-H199A-His_6	88,168	AB165/AB164
		5544	MBP-S_3N_{10}-Prescission-Cps7A-H208A-His_6	88,168	AB159/AB160
Cps7B	ACE62293.1	5152	Cps7B-His_6	17,500	Litschko et al. 2021[1]
Cps7C	ACE62292.1	5154	MBP-S_3N_{10}-Prescission-Cps7C-His_6	87,781	AB112/AB113
		5750	MBP-S_3N_{10}-Prescission-Cps7C-D247A-His_6	87,737	AB213/AB214
		5751	MBP-S_3N_{10}-Prescission-Cps7C-D293A-His_6	87,737	AB215/AB216
Cps7D	ACE62291.1	4887	MBP-S_3N_{10}-Cps7D-His_6	191,732	Litschko et al. 2018[2]
		5055	MBP-S_3N_{10}-Cps7D-H743A-His_6	191,665	Litschko et al. 2018[2]
		5058	MBP-S_3N_{10}-Cps7D-R1123A-His_6	191,646	Litschko et al. 2018[2]
		6064	MBP-S_3N_{10}-Cps7$D_{234\text{-}1277}$-His_6	164,451	AB247/AB248
		6209	MBP-S_3N_{10}-Cps7$D_{289\text{-}1277}$-His_6	158,107	AB260/AB248
		6268	MBP-S_3N_{10}-Cps7$D_{339\text{-}1277}$-His_6	151,896	AB263/AB248
		6180	MBP-S_3N_{10}-Cps7$D_{371\text{-}1277}$-His_6	147,908	AB259/AB248
		6065	MBP-S_3N_{10}-Cps7$D_{465\text{-}1277}$-His_6	137,004	AB249/AB248
CsaA	CAM07513.1	3528	Strep-CsaA-His_6	44,732	Fiebig et al. 2014[3]
CsaD	CAM07516.1	4847	MBP-S_3N_{10}-Thrombin-CsaD-His_6	77,711	AB86/AB85
CslA	CCP19790.1	5027	ΔN15-CslA-His_6	42,645	CL145/CL146
CsxB	ATG32052.1	5546	MBP-S_3N_{10}-Prescission-CsxB-His_6	83,535	TF170/TF171
CsxC	ATG32051.1	5547	MBP-S_3N_{10}-Prescission-CsxC-His_6	72,810	TF172/TF173
KfiB	TEZ99055.1	5668	MBP-S_3N_{10}-Prescission-KfiB-His_6	109,220	Sulewska et al. 2023[4]

CslB	Q9RGQ9		Only used for alignment (Supplementary Fig. 3a)		Litschko et al. 2018[2]
Cps1B	E0EA77		Only used for alignment (Supplementary Fig. 3a)		Litschko et al. 2018[2]
Cps12B	Q69AA8		Only used for alignment (Supplementary Fig. 3a)		Litschko et al. 2018[2]
Ccs2	AEC50903.1		Only used for alignment (Supplementary Fig. 3a)		Litschko et al. 2018[2]
Fcs2	AAQ12660.1		Only used for alignment (Supplementary Fig. 3a)		Litschko et al. 2018[2]
Cps4B	F4YBG0		Only used for alignment (Supplementary Fig. 3a)		Litschko et al. 2018[2]
Bt Y31	OAQ14264.1		Only used for alignment (Supplementary Fig. 3a)		Litschko et al. 2018[2]

Supplementary Table 2: Primers used in this study

Primer	Sequence (5' to 3')
AB85	aagttcctcgagattttttagaaattttcgatcaaaatc
AB86	gcatctggatcccgtaagattacttttattatcc
AB112	gcatctggatcctttcaaatcctacaaaagcatttacc
AB113	gcatctctcgagttttctgaatatatatattttcatttcagc
AB124	gcatctggatccttgatgaaaatagcatttatcgc
AB125	gcatctctcgagtttttaattaatcttttagaaatctgtc
AB157	ttgcattgcttcaagcgggatatgctaaggaaccg
AB158	ttttggtgcggtgaaacaaataaag
AB159	cacgcttttattagcaaacaaggcgcagaattatgaaaaattgtatcc
AB160	ttatggtgtaattttactaacacgttg
AB163	aacgtgttagtaaaattagcgcataacacgcttttattagc
AB164	gtaaaataaagataatttcgtaatttc
AB165	aacgtgttagtaaaattacacgcgaacacgcttttattagcaaac
AB182	gcatctggatccttgatgaaaatagcatttatcgc
AB183	gcatctctcgagtttttaattaactttttagaaatctg
AB184	gcatctggatcctttcaaatcctacaaaagcatttacc
AB185	gcatctctcgagttctcgttgtttattaacataaacc
AB213	tagggcatgataaagctattgatgtcggcgtttgggatgagtactcctacgaag
AB214	aaacgccgacatcaatagctttatcatgccctaataattttccttctcgaatacaacc
AB215	aatggtatcgcaattgctgtgtttattcattatcgcgaacctaatgactattggc
AB216	ataatgaataaacacagcaattgcgataccattaacgtgtcttaacatgactaaatg
AB247	ataacaataacaacaacaataacaataacggatccatactgaatattgaactggcagatg
AB248	ggtggtggtggtggtgctcgagaataacattataaaatctattaattgctt
AB249	ataacaataacaacaacaataacaataacggatccggtaagtatttgcaagcatgcg
AB259	ataacaataacaacaacaataacaataacggatccgggctatatgaaagagcctcaa
AB260	ataacaataacaacaacaataacaataacggatccgctgagttattttatcgtataggtt
AB263	ataacaataacaacaacaataacaataacggatccattcaagctacaaagaattatgaaa
CL86	gcatctcatatgaatagaaaattttctaagttac
CL98	ggtgctcgagactgtaataggagtttaaaaaag
CL145	catatgctatggtccttg
CL100	gaaaacaattgattttgatag
CL101	ttttctcgagcaccac
CL146	cgtgactatttcaacaaaaaaatatc
CL213	ttatttaaatacttgggcgggtacgccaatg
CL214	acctgaccttcttttctaataaaataataaggg
CL215	ttagaggagcgcacttagtagaac
CL216	aaatcagattatatttatttgatttaagttttcttaagtcg
CL221	cgcacgaggcggatcaattaaaattaattg
CL222	acattctgccaatattaataaacttctggcc
CL253	aggtgctgtttcaaggtccgggatccaaacataatgtgaaactatcatctgtgccaagc
CL254	tggtggtggtggtggtgctcgagatttgctagtagtgagtaaaacttagccatgg
IB87	cctggaggtgctgtttcaaggtccgctaaaagagcaaacatataaagcaagtaatgg
IB88	ggtggtggtggtggtgctcgagtttttcaaaataaacaatgttatttactacatgtaatg
JS61	ggtgctgtttcaaggtccgggatccccccagctatattatcgcattggcatag
JS62	ggtgctgtttcaaggtccgggatcctcaaaagttaaagttgccgaaaataagaagaaattagaag
JS63	ggtgctgtttcaaggtccgggatccctaaaccgtaactataaatatcgtatggggtataacctag

TF170	ggtgctgtttcaaggtccgggatcctcaccctcacatcacagcctagtg
TF171	ggtggtggtggtgctcgagtctgttcagggacggagtgagc
TF172	gcatctggatccaaaaaatttttttctatcctctcgatatttatc
TF173	gcatctcgagtcgagagatatattttacgcc

Supplementary Table 3: Chemical shifts of compounds 3, 4 and 5 measured at 298 K and referenced to 2,2-Dimethyl-2-silapentane-5-sulfonate sodium salt (DSS).

Compound	3	4	5
**α-Kdo (A)**			
C1	176.6	176.6	176.6
C2	103.9	103.8	103.8
C3	36.3	36.3	36.3
C4	75.1	75.2	75.3
C5	67.9	67.9	67.9
C6	75.6	75.6	75.6
C7	71.6	71.7	71.5
C8	66.8	66.8	66.9
H3ax	1.820	1.816	1.819
H3eq	2.333	2.329	2.331
H4	3.803	3.801	3.808
H5	4.189	4.191	4.193
H6	3.588	3.579	3.586
H7	3.886	3.887	3.890
H8	3.865	3.866	3.853
H8'	3.734	3.734	3.741
**α-Kdo (B)**			
C1	176.4	176.3	176.4
C2	105.0	105.1	105.1
C3	37.9	37.9	37.9
C4	69.7	69.6	69.7
C5	67.6	67.7	67.6
C6	75.1	75.1	75.1
C7	76.2	76.2	76.2
C8	65.4	65.4	65.5
H3ax	1.874	1.869	1.871
H3eq	2.468	2.461	2.465
H4	3.774	3.777	3.775
H5	3.986	3.990	3.993
H6	3.723	3.725	3.732
H7	4.351	4.350	4.354
H8	3.936	3.936	3.937
H8'	3.903	3.901	3.923
**Gro3P (C)**			
C1	64.7	68.8	68.8
C2	73.4	72.1	72.2
C3	69.1	68.9	68.9
H1	3.691	3.963	3.953
H1'	3.622	3.904	3.894

H2	3.918	4.062	4.073
H3	3.975	4.009	4.009
H3'	3.909	3.942	3.942
P	-2.61	-2.71	-2.72
**Gro3P (E)**			
C1	—	—	68.8
C2	—	—	72.2
C3	—	—	68.9
H1	—	—	3.953
H1'	—	—	3.894
H2	—	—	4.073
H3	—	—	3.999
H3'	—	—	3.930
P	—	—	-2.15
**Gro3P (D)**			
C1	—	64.7	64.8
C2	—	73.4	73.5
C3	—	69.0	69.1
H1	—	3.690	3.692
H1'	—	3.622	3.629
H2	—	3.916	3.925
H3	—	3.929	3.922
H3'	—	3.874	3.875
P	—	-2.10	-2.06
**Linker**			
C1	67.5	67.5	67.5
C2	31.6	31.6	31.6
C3	27.8	27.7	27.8
C4	31.0	31.0	31.0
C5	31.1	31.1	31.1
C6	28.7	28.7	28.8
C7	31.0	31.0	31.0
C8	42.7	42.7	42.7
H1	3.672	3.671	3.677
H1'	3.375	3.372	3.374
H2	1.524	1.526	1.528
H3	1.308	1.307	1.308
H4	1.314	1.311	1.311
H5	1.342	1.346	1.346
H6	1.373	1.374	1.376
H7	1.612	1.613	1.617
H8	3.374	3.373	3.372

Supplementary Table 4. Data collection and refinement statistics. Statistics for the highest-resolution shell are shown in parentheses.

	Cps3D
PDB code	8QOY
Beamline	I24 – Diamond Light Source
Wavelength (Å)	0.6702
Resolution range (Å)	41.55 - 3.0 (3.1 - 3.0)
Space group	P 3 2 1
Unit cell	207.911 207.911 93.6591<br>90 90 120
Total reflections	2925443 (298976)
Unique reflections	46791 (4630)
Multiplicity	62.5 (64.5)
Completeness (%)	99.86 (99.94)
Mean I/sigma(I)	9.23 (1.66)
Wilson B-factor	54.33
R-merge	0.5283 (3.253)
R-meas	0.5325 (3.278)
CC1/2	0.998 (0.936)
CC*	0.999 (0.983)
Reflections used in refinement	46749 (4630)
Reflections used for R-free	2297 (255)
R-work	0.1833 (0.2814)
R-free	0.2199 (0.3203)
CC(work)	0.379 (0.262)
CC(free)	0.422 (0.288)
Number of non-hydrogen atoms	8529
macromolecules	8498
ligands	11
solvent	20
Protein residues	1036
RMS(bonds)	0.002
RMS(angles)	0.50
Ramachandran favored (%)	95.45
Ramachandran allowed (%)	4.36
Ramachandran outliers (%)	0.19
Rotamer outliers (%)	0
Clashscore	5.60
Average B-factor	59.89
macromolecules	59.90
ligands	81.53
solvent	43.27

Supplementary Table 5: ^{1}H and ^{13}C chemical shifts of hydrolyzed App3 and App7 polymer backbones at 298 K referenced to 2,2-Dimethyl-2-silapentane-5-sulfonate sodium salt (DSS).

App3									
	Gal						Gro		
	C1/H1	C2/H2	C3/H3	C4/H4	C5/H5	C6/H6(+H6')	C1/H1(+H1')	C2/H2	C3/H3+H3'
repeating unit	100.9/ 5.215	71.3/ 3.896	71.6/ 3.986	77.4/ 4.542	73.5/ 4.177	63.6/ 3.752	64.0/ 3.790	79.9/ 3.971	67.2/ 4.116+4.054
Gal at non-red. end	101.0/ 5.203	71.2/ 3.816	72.1 3.911	72.1/ 3.998	73.5/ 4.094	63.9/ 3.749			
Gal next to red. end	100.8/ 5.180	71.2/ 3.917	71.5/ 3.987	77.4/ 4.554	73.5/ 4.187	63.6/ 3.752			
Gro at red. end							63.1/ 3.774+3.736	81.6/ 3.835	(63.1/ 3.774+3.736)
App7									
	Gal						Gro		
	C1/H1	C2/H2	C3/H3	C4/H4	C5/H5	C6/H6(+H6')	C1/H1(+H1')	C2/H2	C3/H3+H3'
repeating unit	101.1/ 5.014	70.1/ 3.4969	77.6/ 4.380	71.2/ 4.213	73.5/ 3.980	63.9/ 3.758	71.1/ 3.824+3.638	71.9/ 4.129	69.2/ 4.042+3.989
Gal at non-red. end	101.1/ 4.967	72.0/ 3.821	72.2/ 3.913	72.1/ 3.998	73.6/ 3.953	63.9/ 3.742			
Gro at red. end							71.3/ 3.774+3.589	73.2/ 3.971	65.2/ 3.691+3.660

SUPPLEMENTARY FIGURES

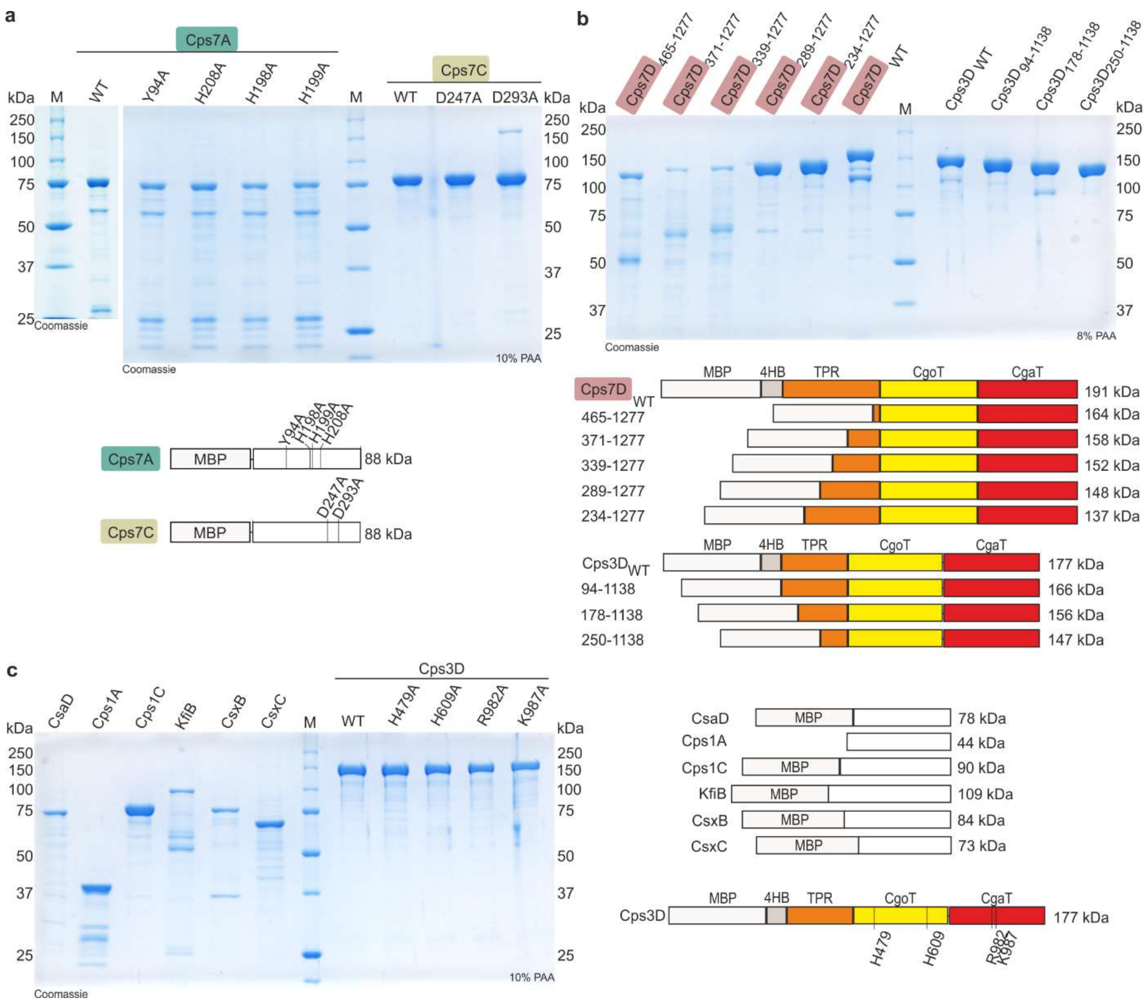

Supplementary Fig. 1 | Coomassie-stained polyacrylamide gels and schematic representations of all constructs (final pools) used in this study. a, Cps7A and Cps7C wildtype and amino acid exchange mutants. **b,** Cps7D and Cps3D truncation constructs and **c,** putative transitions transferases and Cps3D amino acid exchange mutants. Cps3D wildtype was used for crystallization studies. Abbreviations are: α4HB, region comprising a bundle of four α-helices; CgaT, capsule α-1,1-galactosyl transferase; CgoT, capsule glycerol-3-phosphate transferase; MBP, maltose-binding protein; TPR, tetratricopeptide repeat.

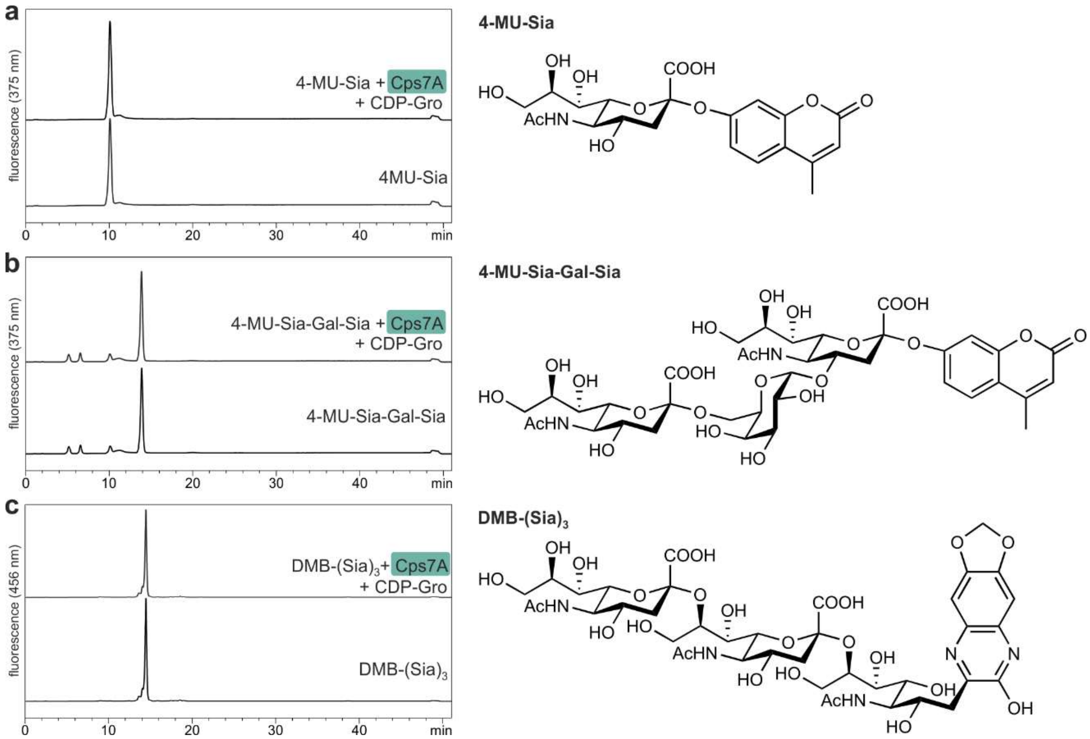

Supplementary Fig. 2 | Alternative acceptors for the transition transferase Cps7A. To investigate if the reaction catalysed by Cps7A was specific for the terminal ulosonic acid Kdo, several compounds ending with 5-amino-3,5-dideoxy-D-glycero-D-galacto-non-2-ulosonic acid (Neu5Ac, Sia) were tested as acceptor. These compounds were **a**, 2'-(4-methylumbelliferyl)-labelled (4-MU) sialic acid and **b**, a 4-MU-labelled trimer of the *Neisseria meningitidis* serogroup W capsule polymer repeating unit, both obtained from Romanow et al[5], as well as **c**, a 1,2-diamino-4,5-methylenedioxybenzene (DMB)-labeled trimer of the *Neisseria meningitidis* serogroup B capsule polymer repeating unit, obtained from Keys et al[6]. None of the above described compounds were suitable acceptor substrates for Cps7A.

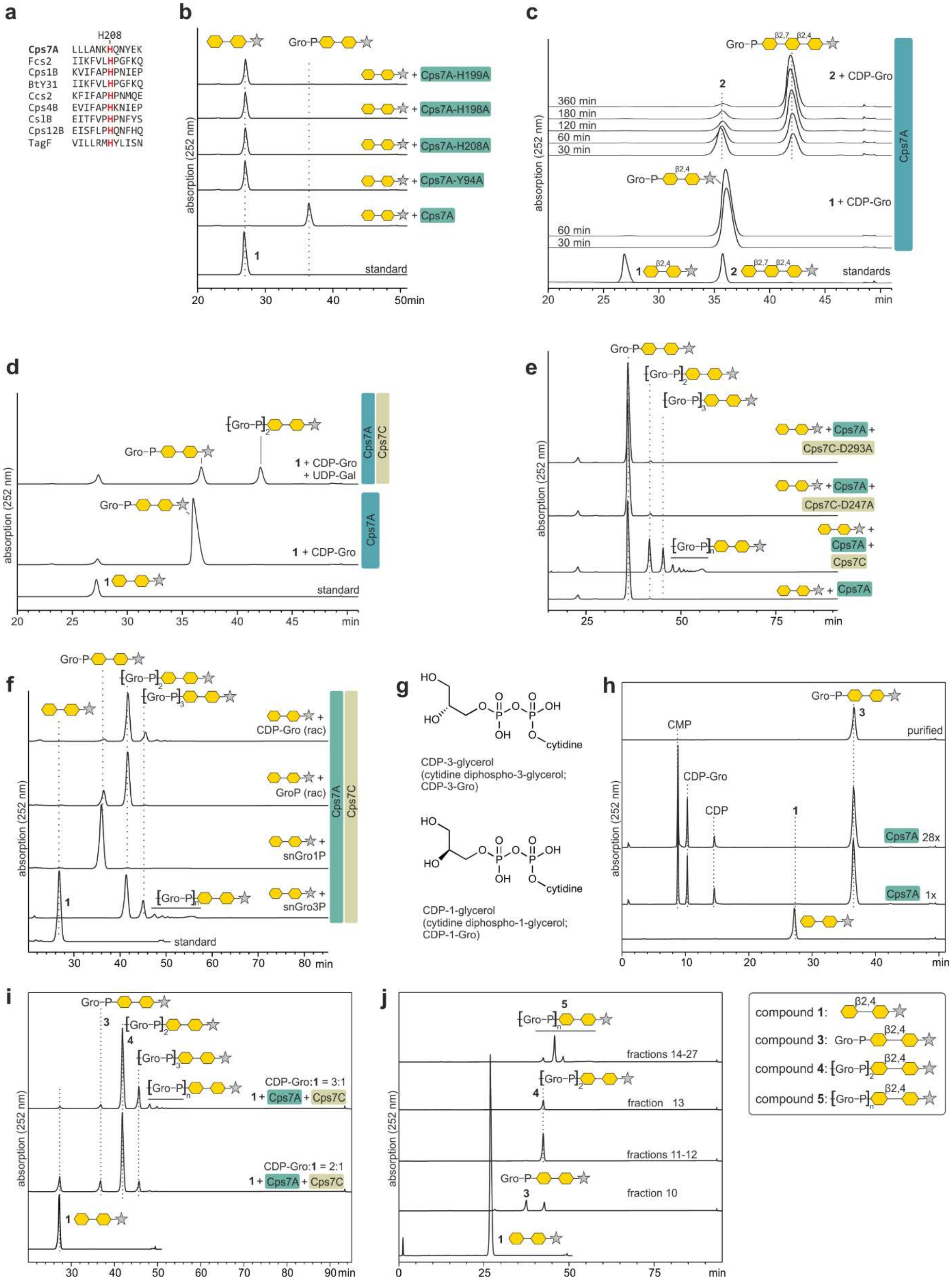

Supplementary Fig. 3 | Characterization of Cps7A and Cps7C and purification of their products. a, In an alignment (see Supplementary Table 1 for accession codes) between Cps7A, TagF and TagF-like hexose-phosphate transferases using Clustal Omega[7], H584 of TagF (see Extended Data Fig. 3a,b) aligned with H208 of Cps7A. **b,** Cps7A-Y94A, Cps7A-H198A, Cps7A-H199A (see Extended Data Fig. 3b) and Cps7A-H208A (see panel **a**) were cloned and purified (Supplementary Fig. 1). None of the mutants could utilize compound **1** as an acceptor, supporting a role of the targeted amino acids in the enzyme's

catalytic mechanism. **c**, Time-course of Cps7A incubated with CDP-Gro and either compounds **1** or **2**. **d**, Cps7C generates a product that elutes with increased retention time in the presence of UDP-Gal / CDP-Gro, indicating that the enzyme transfers glycerol-3-phosphate instead of galactose. **e,** Cps7C-D247A and Cps7C-D293A (Extended Data Fig. 3c) were generated and purified (Supplementary Fig. 1), but could not elongate the reaction product of Cps7A (**1** + GroP), supporting the proposed enzymatic activity for Cps7C. **f,** Donor substrate preference of Cps7A and Cps7C. We recently showed that the CTP:glycerol-phosphate cytidylyltransferase (GCT) Cps7B of App7 can utilize GroP and CTP to generate CDP-Gro, which can then be used by the polymerase Cps7D as donor substrate[1]. While both GCT (Cps7B) and polymerase (Cps7D) display a clear preference for Gro3P, Gro1P could also be used[1]. Cps7A and Cps7C were incubated with racemic CDP-Gro (top chromatogram). Alternatively, racemic CDP-Gro, or enantiopure CDP-1-Gro or CDP-3-Gro, were produced *in situ* using CTP:glycerol-phosphate cytidylyltransferase (GCT), CTP and the glycerol-phosphate enantiomers as indicated (racemic GroP (rac), Gro1P, Gro3P). While Cps7A was able to transfer Gro1P from CDP-1-Gro onto **1**, Cps7C could not further extend the resulting product (compare bottom and third chromatogram). In line with the substrate preference of GCT and Cps7D, most product was produced in the presence of enantiopure Gro3P, indicating that also Cps7C prefers this enantiomer. Products were shorter in reactions containing a racemic mix of CDP-Gro, presumably because only CDP-3-Gro was utilized. **g**, Chemical structures of CDP-3-glycerol and CDP-1-glycerol. **h,** A Cps7A reaction (1x, 50 uL) and an up-scaled reaction (28x, 1.4 ml) were compared by HPLC-AEC, showing that up-scaling did not affect the reaction yield and that complete conversion of compound **1** was achieved. Preparative AEC followed by dialysis was used to purify the product of the Cps7A reaction (compound **3**) from other constituents. **i,** To maximize consumption and minimize co-purification of compounds **1** and **3,** Cps7A/C were incubated with CDP-Gro and compound **1** at ratios of 2:1 and 3:1. At a 3:1 ratio, compound **1** and compound **3** were almost completely consumed. **j,** The reaction was scaled up, purified by AEC and fractions were analyzed by HPLC-AEC. Fractions 11-13 and 14-27 were pooled, yielding compounds **4** and **5**, respectively.

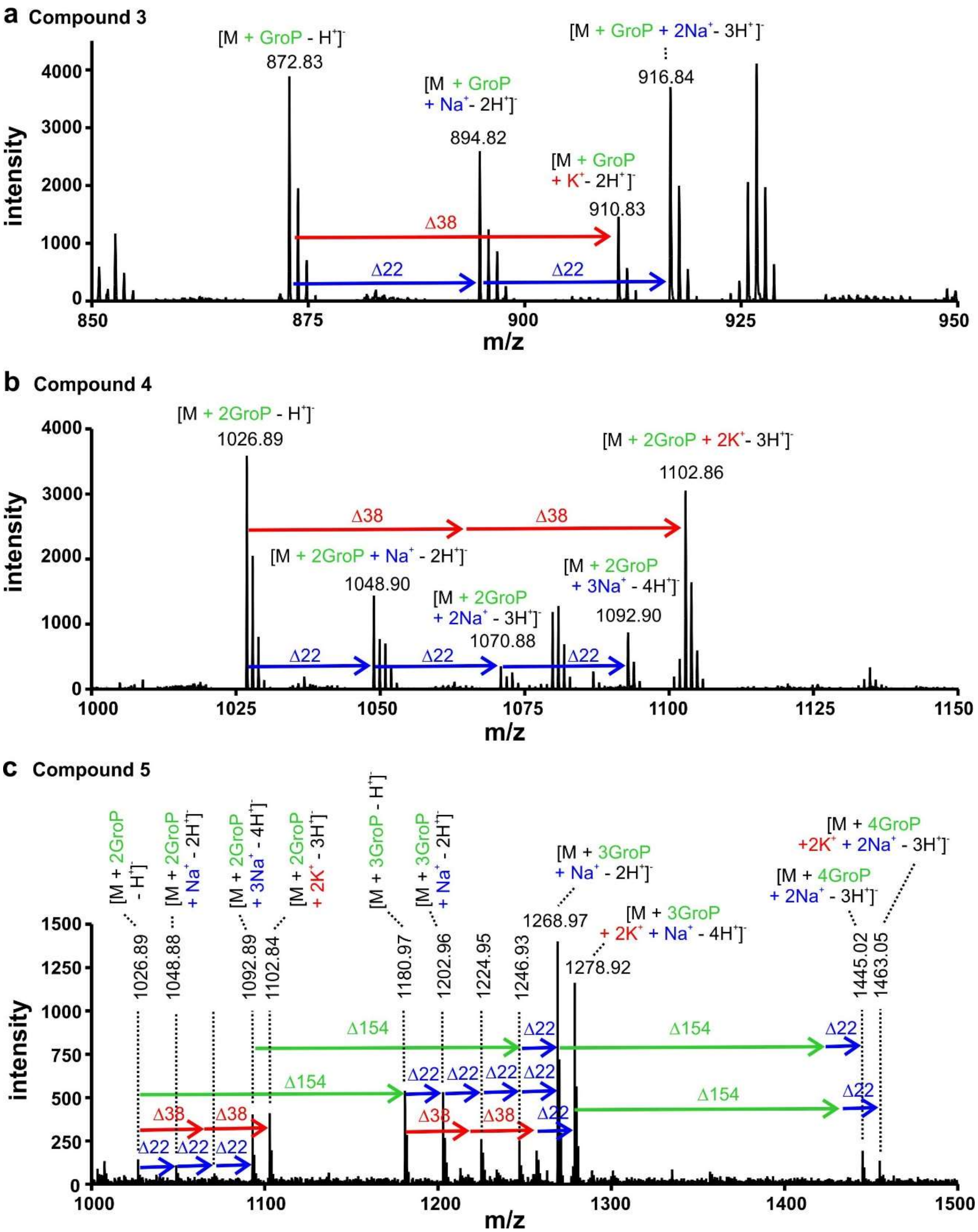

Supplementary Fig. 4 | Mass spectrometry analysis of products synthesized by Cps7A and Cps7C. a, Compound **3** (**1** elongated by one Gro3P) **b,** compound **4** (**1** elongated by two Gro3P) **c,** compound **5** (**1** elongated by several Gro3P). Mass shifts caused by the covalent attachment of GroP (+154 Da) or by the formation of Na^+- (+22 Da) or K^+-ion adducts (+38 Da) are highlighted by green, blue or red arrows, respectively.

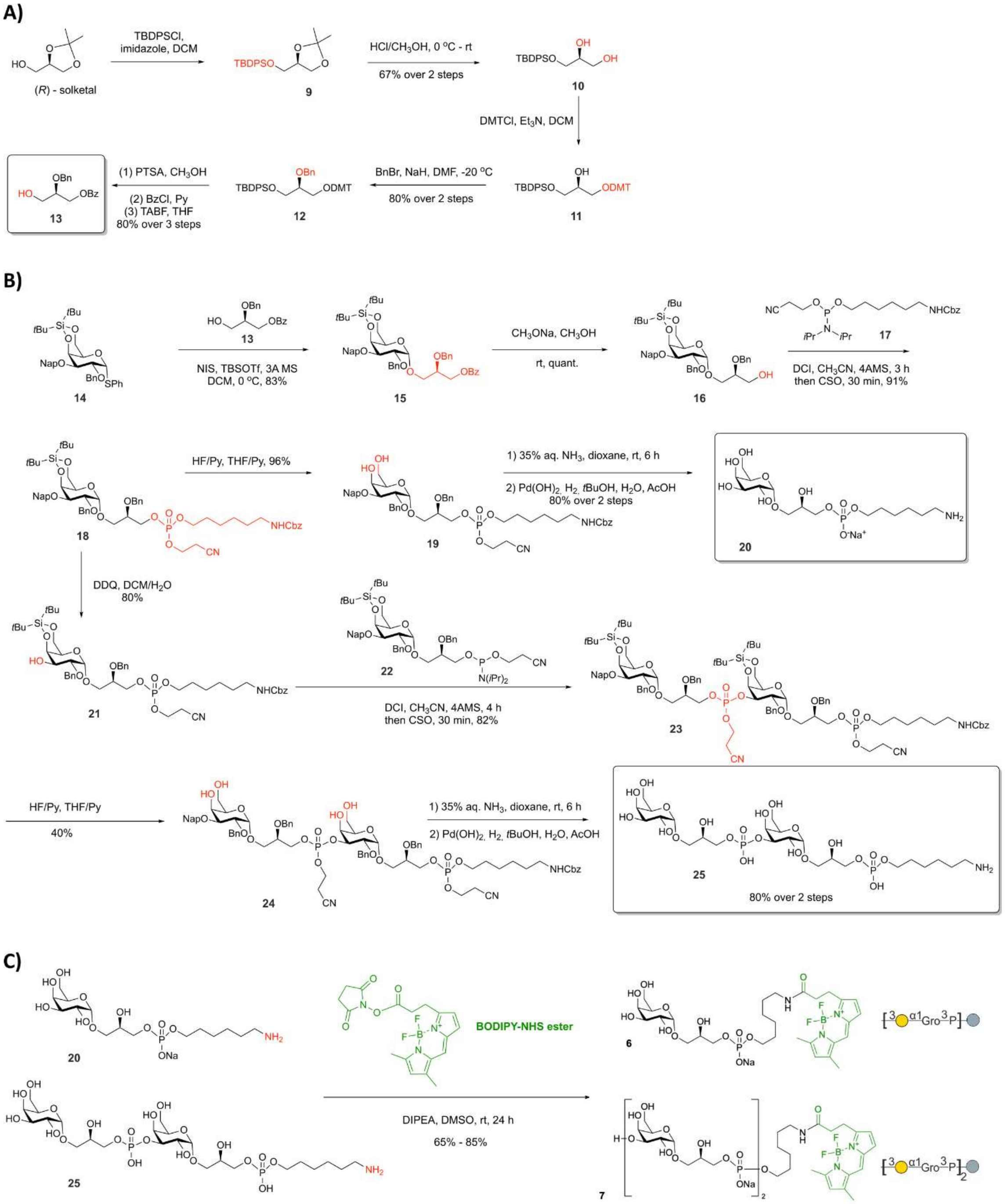

Supplementary Fig. 5 | Synthesis of BODIPY-labeled App7 capsule polymer fragments (compounds 6 and 7). Detailed information is presented as separate file (see Supplementary Note) a, Synthesis of glycerol building blocks. **b,** Synthesis of App7 monomer and dimer. **c,** Coupling of App7 monomer and dimer with BODIPY.

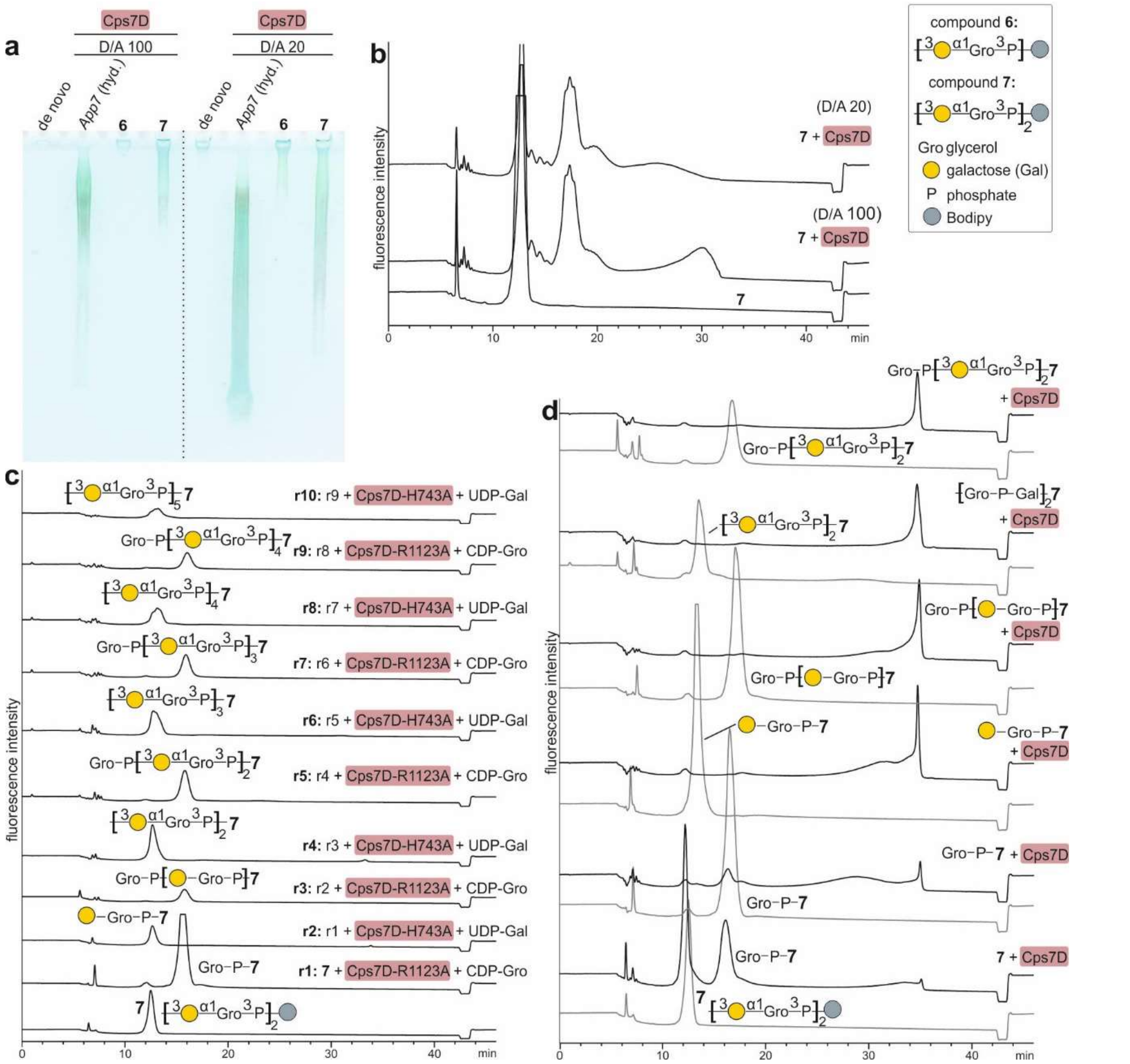

Supplementary Fig. 6 | Acceptor preference and product profile of Cps7D. a, b To test if compounds **6** and **7** were suitable to prime elongation by Cps7D, assays were performed at donor (CDP-Gro/UDP-Gal) to acceptor (compounds **6** or **7**) substrate ratios (d/a) of 100 and 20. Non-tagged hydrolyzed fragments of the App7 polymer backbone (App7 hyd.) were used as positive control. Since TagF-like polymerases display *de novo* activity even in the absence of acceptor[2], Cps7D was used in a concentration in which *de novo* activity was reduced to a minimum (see control *de novo*). The resulting products were compared on an Alcian blue / silver-stained gel (**a**) and by HPLC-AEC with fluorescence detection (**b**). The Alcian blue silver stained PA gel clearly shows that the amount of product in the presence of compound **7** is above *de novo* activity. The fact that the product size decreases with decreasing d/a confirms that the elongation is specific. In contrast, activity in the presence of compound **6** is hardly above *de novo* activity, and elongation could barely be observed by HPLC-AEC (not shown), indicating that a single RU as present in compound **6** is not a suitable acceptor. **c,** To investigate the elution behavior of small to intermediate sized chains in the HPLC-AEC assay in more detail, compound **7** was elongated enzymatically in a stepwise manner to obtain larger oligomers. The previously published single action transferase Cps7D-R1123A[2], in which only CgoT remained active, was utilized to add a Gro3P to the acceptor (**c,** second chromatogram from the bottom, GroP-7). After

removing the enzyme from the reaction by filtration, the flow-through was supplemented with Cps7D-H743A[2], in which only CgaT activity remains (**c**, third chromatogram from the bottom, Gal-GroP-7). This procedure was repeated until a heptamer of the App7 repeating unit was obtained. The addition of a complete repeating unit always led to product eluting with a retention time identical to the acceptor itself (see also Extended Data Fig. 4c, r3). **d,** It was tested if the products assembled in (**c**) can be utilized by wildtype Cps7D to prime the assembly of larger polymers. The donor to acceptor ratio was high (2.5 µM acceptor, 5 mM donor) to allow the generation of long chains. The bottom (grey) and top (black, bold) chromatogram of each pair show the acceptor as a control, and the products after elongation by Cps7D, respectively. Indeed, long products eluting at later retention times could be produced in each reaction. However, considerable amounts of compound **7** and GroP-**7** were neglected by Cps7D, indicating that acceptors larger than these are preferred by the enzyme.

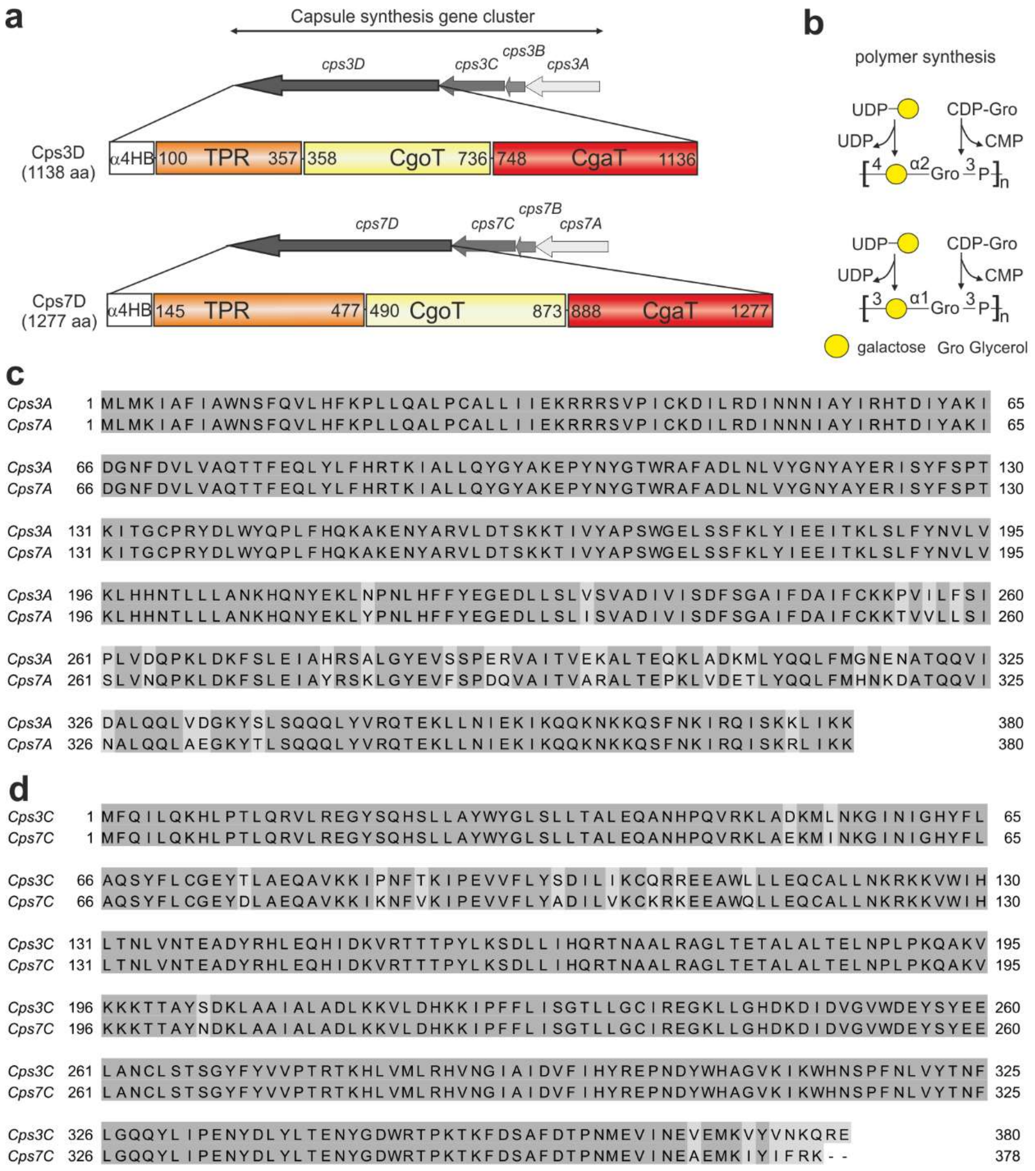

Supplementary Fig. 7 | Comparison of App3 and App7 proteins. a, Schematic showing the genetic organization of region 2 of the capsule synthesis gene clusters of App3 and App7 and the predicted domain organization[2] of the capsule polymerases Cps3D and Cps7D. Both enzymes contain a N-terminal bundle of four α-helices (α4HB), a domain rich in tetratricopeptide repeats (TPR), the CgoT (**C**apsule **g**lycer**o**l-3-phosphate **T**ransferase) domain and the CgaT (**C**apsule α-**ga**lactosyl **T**ransferase) domain. The length of each polypeptide in amino acids (aa; indicated in parentheses after each name) is indicated. The information presented for the Cps3D domain organization is based on the crystal structure solved in this study, whereas details for Cps7D result from Phyre2[8]/Alphafold[9] predictions. **b,** Reactions catalyzed by Cps3D and Cps7D. **c, d,** Sequence alignments of **c,** Cps3A (GenBank accession number: ABU63689.1) and Cps7A (GenBank accession number: ACE62294.1) and **d,** Cps3C (GenBank accession number: UKH44265.1) and Cps7C (GenBank accession number: ACE62292.1) from App3 strain S1421 and App7 strain AP76.

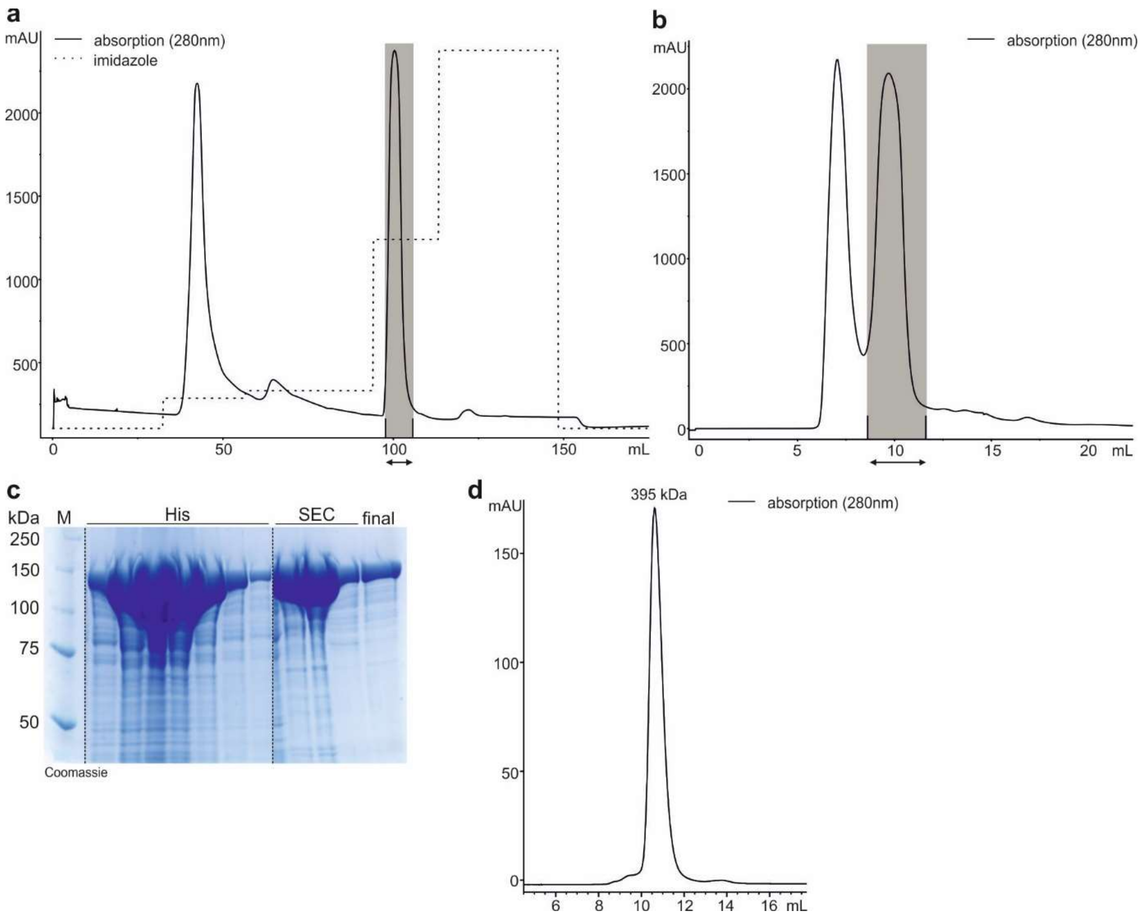

Supplementary Fig. 8 | Purification of the crystallization construct MBP-Cps3D$_{2\text{-}1138}$-His$_6$. a, Affinity chromatography (IMAC, HisTrap™). Fractions highlighted by a grey box were subjected to **b,** size exclusion chromatography (SEC). Roughly half of the construct eluted in the void volume (first peak). Grey boxed fractions were pooled and used for crystallization studies. **c,** Coomassie staining after SDS-PAGE of protein samples taken during the purification procedure and the final protein used for crystallization. A dashed line indicates that samples not relevant for data presentation were excised. **d,** Size exclusion chromatography, extracted from Supplementary Fig. 12b. MBP-Cps3D-His$_6$ (Mw = 176 kDa) elutes with an apparent molecular weight (Mw_{obs}) of 395 kDa, confirming the formation of dimers in solution (Mw_{obs}:M_w = 2,25).

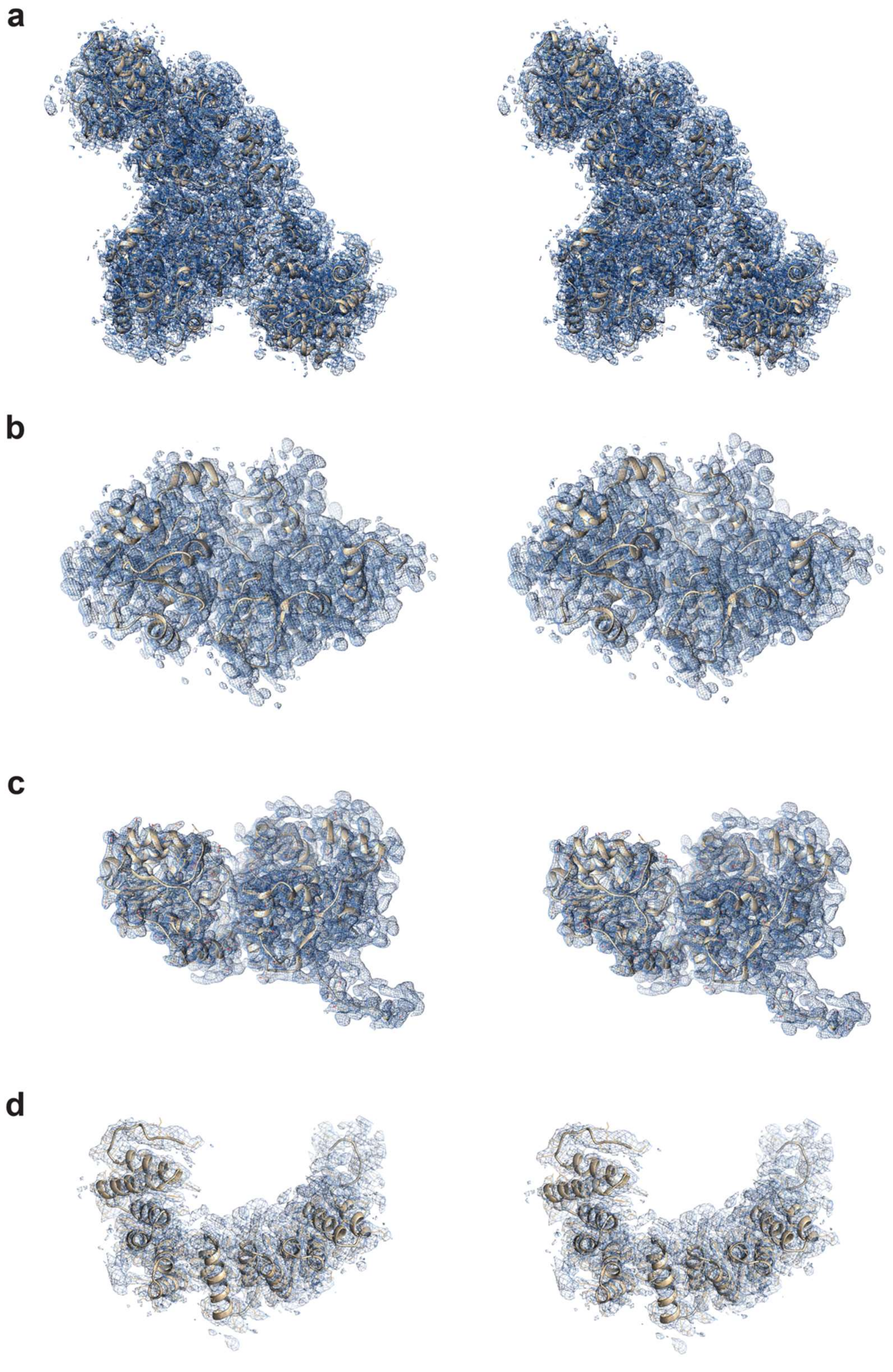

Supplementary Fig. 9 | Stereo view of Cps3D. a, Electron density of Cps3D shown at 1.0 σ r.m.s deviation. **b,** Electron density of the CgoT domain shown at 1.0 σ r.m.s deviation. **c** Electron density of CgaT domain shown at 1.0 σ r.m.s deviation. **d** Electron density of the TPR domain shown at 1.0 σ r.m.s deviation.

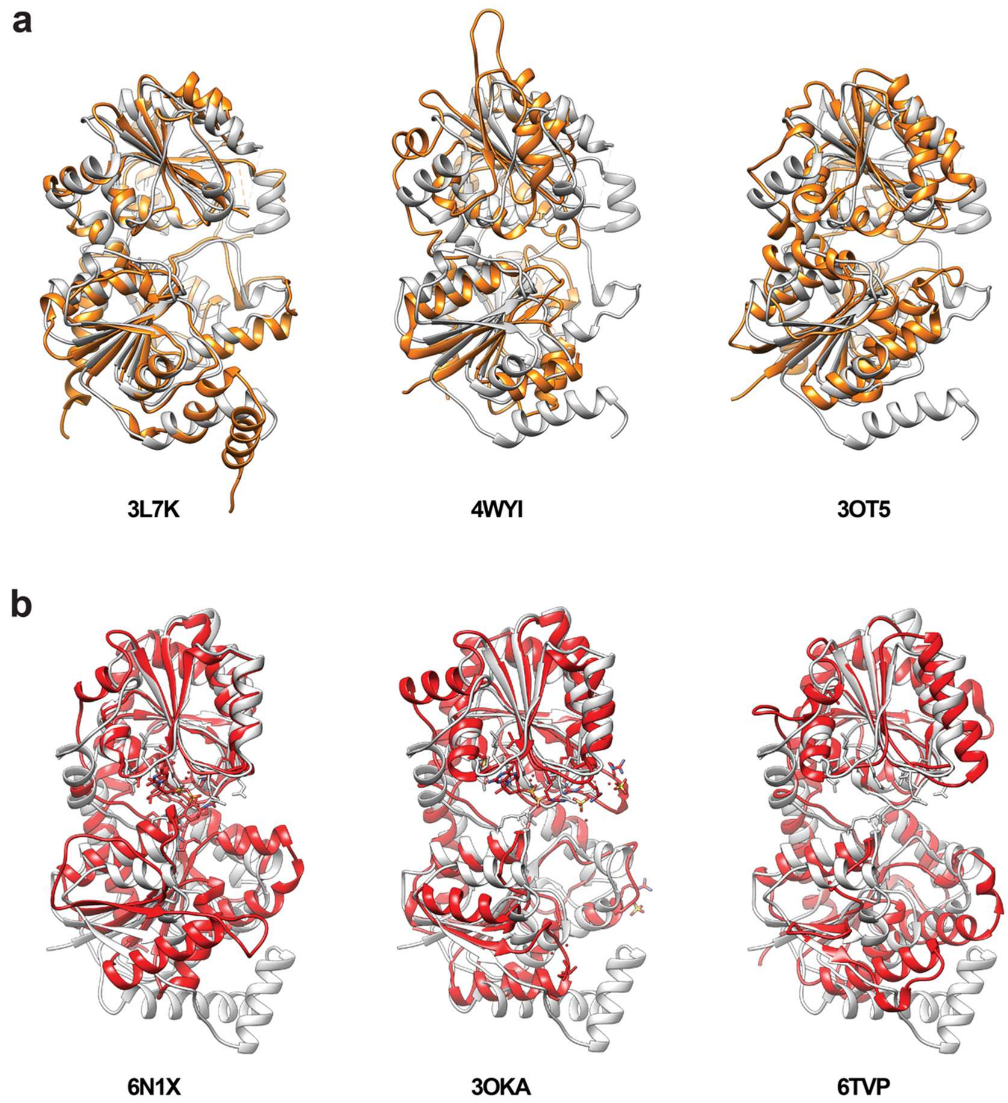

Supplementary Fig. 10 | Structural homologues of Cps3D. a Structural superposition of the X-ray crystal structure of CgoT (yellow) and the structural homologues (grey) TagF (Wall Teichoic Acid polymerase from *S. epidermis*; PDB code 3L7K), MGD1 (Galactolipid synthase from *Arabidopsis thaliana*; PDB code 4WYI), and LMO2537 (Putative UDP-*N*-acetylglucosamine 2-epimerase from *Listeria monocytogenes*; PDB code 3OT5). **b** Structural superposition of the X-ray crystal structure of CgaT (red) and the structural homologue (grey) BshA (Glycosyltransferase from *S. aureus*; PDB code 6N1X), PimB' (mannosyltransferase from *Corynebacterium glutamicum*; PDB code 3OKA), and GlgM (alpha-maltose-1-phosphate synthase from *Mycobacterium smegmatis*); PDB code 6TVP).

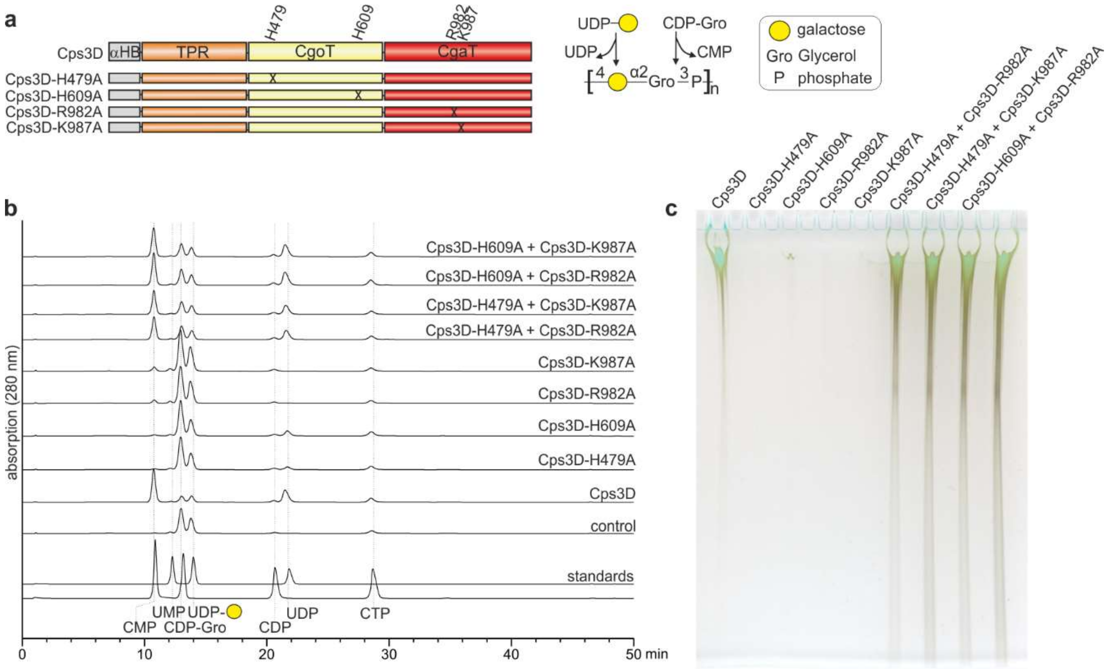

Supplementary Fig. 11 | Analysis of the active site of CgoT and CgaT of Cps3D and site-directed mutagenesis of crucial amino acid residues. a, Schematic representation of Cps3D including an overview of Cps3D mutations analyzed in this study and the reaction scheme of Cps3D. Homologs of active site residues H479 and H609 of CgoT in TagF[10] and TagF-like capsule polymerases[2,11,12] have been described as crucial for enzyme activity. Residues R982 and K987 are highly conserved in TagF-like capsule polymerases[2], and frequently involved in coordinating the phosphate moieties in retaining GT-B fold enzymes[13,14]. **b,** HPLC-AEC analyses and **c,** Alcian blue/silver-stained PA gel showing that all single-domain mutants were unable to (i) consume the donor substrates CDP-Gro/UDP-Gal (shown in panel **b**) and (ii) produce polymer (shown in panel **c**), whereas the combination of two single-domain mutants in *trans* restores donor substrate uptake (shown in panel **b**) and polymer production (shown in panel **c**)**.**

When analysing the interaction between active site residues of CgoT and CgaT with CDP-Gro and UDP-Gal (both placed by molecular docking, see Fig. 4 and Extended Data Fig. 6 and 7), respectively, the following interaction were observed. **CgoT/CDP-Gro**: (i) the backbones of G608 and P574 together with the side chain or R607 interact with cytosine N4, (ii) A573 interacts with C4 and C5 and S632 makes hydrogen bonding with O2 of the cytosine skeleton, (iii) R542 makes hydrogen bonds with the O2' and the O3' of the ribosyl ring, (iv) H479 makes a hydrogen bond with O2 of the glycerol moiety. **CgaT/UDP-Gal**: (i) the side chain of Q1038 makes electrostatic interactions with O4 of the uridine skeleton, (ii) E1067 hydrogen bonds with O2 and with the O3 hydroxyl groups, (iii) the main chain of R982 interacts with the O1 of the β-PO_4 (iv) the side chain of K987 interacts with O1 of the α-PO_4 and the O3 and the O1 of the β-PO_4, (v) K987 also hydrogen bonds with the O5' of the galactose, (vi) R764 hydrogen bonds the O2' of the galactose residue while the Q1061 side chain interacts with the O3', (vii) E1059 makes hydrogen bonds with the O4' and with the O6'.

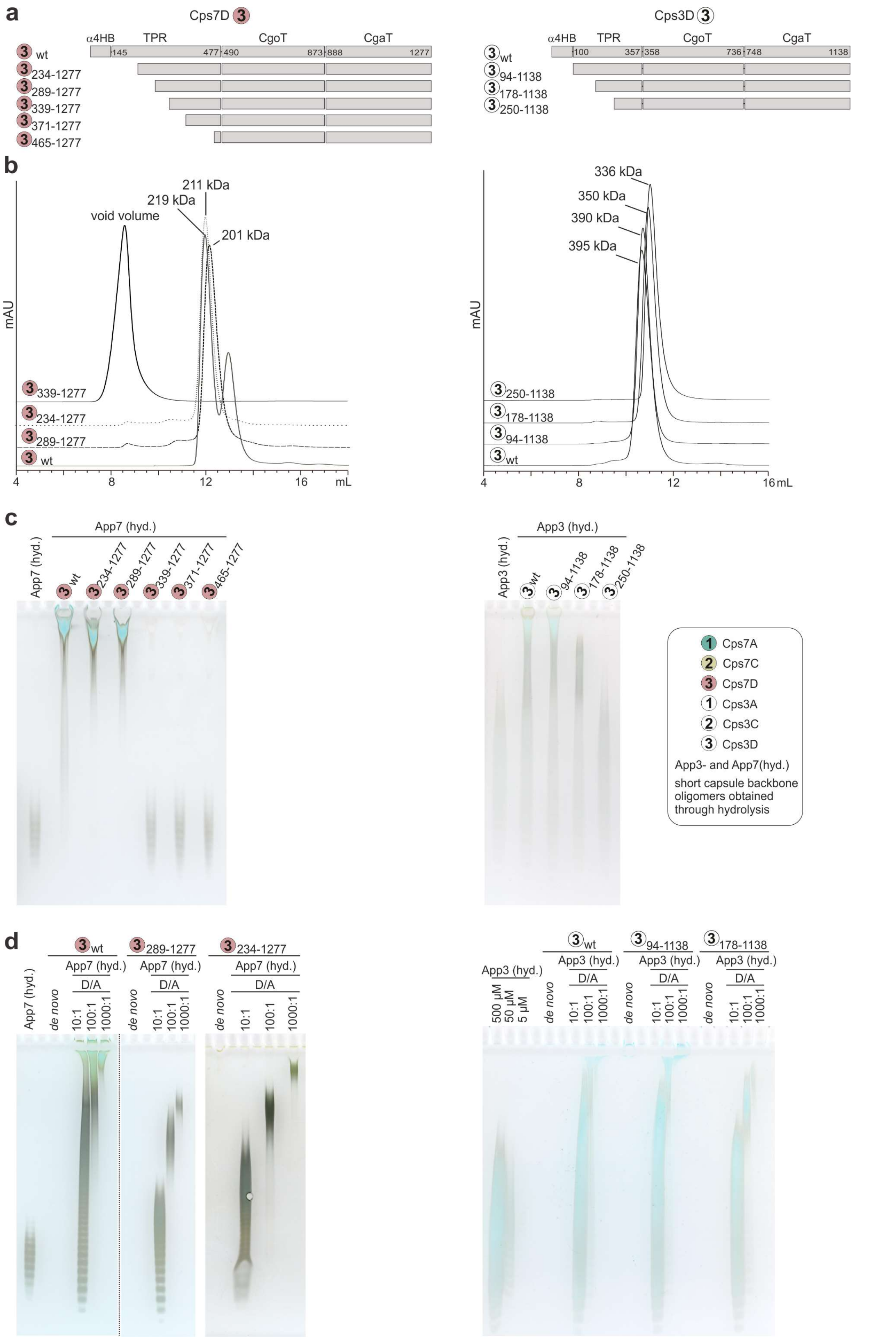

Supplementary Fig. 12 | Biochemical characterization of wildtype and N-terminally truncated Cps3D and Cps7D constructs. a, N-terminally truncated Cps7D (left) and Cps3D (right) constructs generated in this study. All analyzed constructs (full-length and truncations) are N-terminally fused with maltose-binding protein (MBP) and C-terminally fused to a hexa-histidine tag not shown in the schematic

(compare Supplementary Fig. 1). Abbreviations are: α4HB, region comprising a bundle of four α-helices; MBP, maltose-binding protein; CgaT, capsule α-1,1-galactosyl transferase; CgoT, capsule glycerol-3-phosphate transferase; TPR, tetratricopeptide repeat. **b,** Size exclusion chromatography of constructs as indicated. Introduced truncations do not affect the dimeric and monomeric state of Cps3D and Cps7D, respectively. $Cps7D_{339\text{-}1277}$ elutes in the void volume, most likely due to aggregation. **c,** PAGE analysis of reactions containing different N-terminally truncated Cps3D and Cps7D constructs. Hydrolyzed capsule polymer (App7 (hyd.), App3 (hyd.), see Supplementary Fig. 15) was used as acceptor substrate. Due to pronounced degradation and lower concentration of the constructs of interest in preparations of $Cps7D_{339\text{-}1277}$, $Cps7D_{371\text{-}1277}$ and $Cps7D_{465\text{-}1277}$ (Supplementary Fig. 1), these constructs were used at a fivefold concentration. **d,** Product profiles generated by the active N-terminal truncations of Cps7D ($Cps7D_{289\text{-}1277}$, $Cps7D_{234\text{-}1277}$) (left) and Cps3D ($Cps3D_{94\text{-}1138}$, $Cps3D_{178\text{-}1138}$) (right) were analyzed in comparison to the product profiles generated by Cps7D and Cps3D wildtype in the presence of variable amounts of acceptor substrates. The wildtype enzymes appear to be processive, generating large products even at low donor to acceptor ratios (d/a), whereas the size of the products generated by the constructs with a truncated TPR domain can be influenced by the d/a ratio, indicating a more distributive elongation mechanism. Truncating the α4HB from Cps3D does not influence the elongation mode.

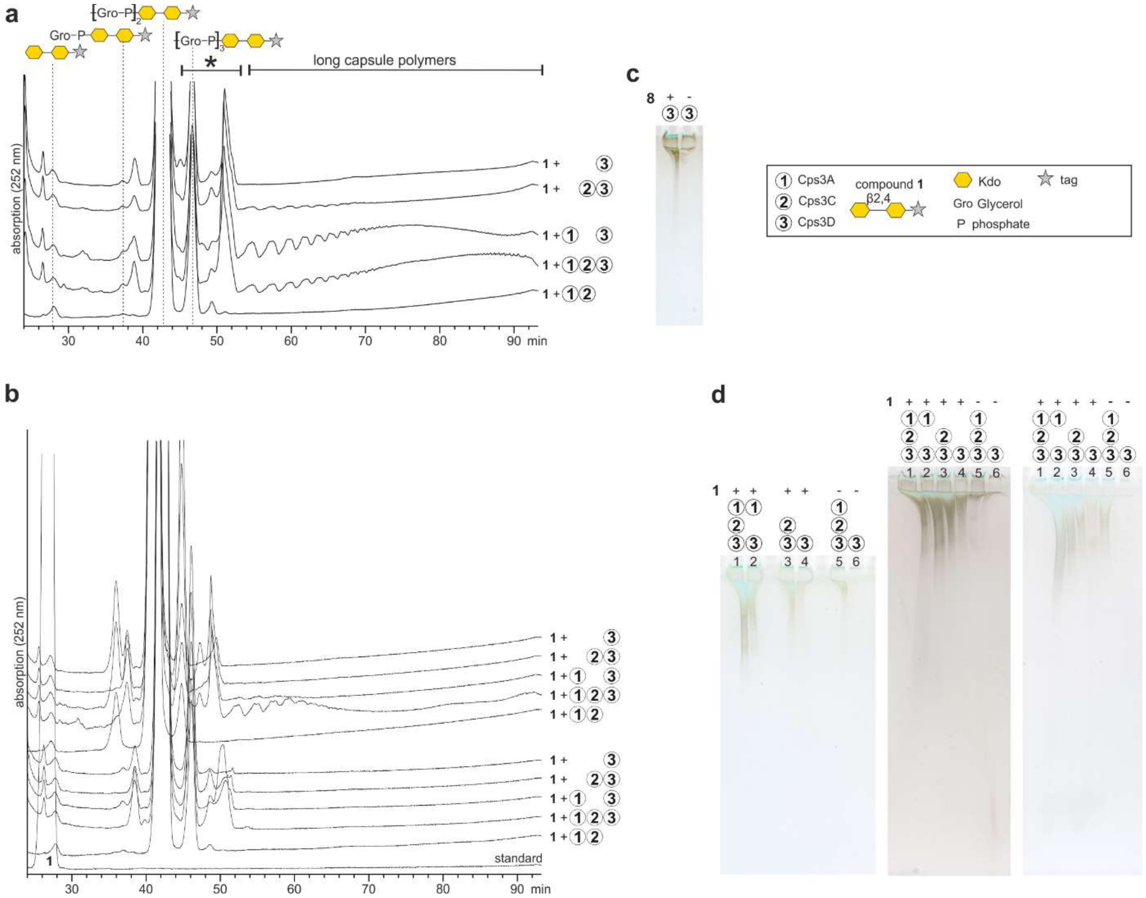

Supplementary Fig. 13 | Transition transferases stimulate the capsule polymerase to produce more polymer and longer chains. PAGE and HPLC-AEC analysis of reaction products from App3 biosynthesis enzymes. In the reactions analyzed in panels **a**, **b** and **d**, compound **1** was elongated with Cps3A and Cps3C, the enzymes were removed by filtration and the filtrate was used as acceptor with substrate and proteins as indicated. In panel **c**, $(Gro3P)_5$ (compound **8**) was elongated with Cps3D, indicating that Kdo is not required to prime elongation.

In analogy to the App7 biosynthesis system, more product was observed after PAGE (**d**), when Cps3A/C were present, both with compound **1** (compare lanes 1-3 to lane 4 in each gel) and *de novo* (compare lanes 5 and 6 in each gel). The production of long chains detectable by HPLC-AEC (**a**,**b**) was also stimulated by Cps3A (and to a lesser extent by Cps3C). However, while the stimulating effect was always visible by PAGE, long chains were not reproducibly detectable by HPLC-AEC (three out of five experimental repeats, of which three are shown here to document the extent of variation). The samples analyzed on the middle and right gel in panel (**d**) correspond to the bottom and top set of chromatograms shown in panel (**b**). The comparison between PAGE and HPLC-AEC shows that (i) oligo-/polymers of considerable length after separation by PAGE still elute early in the HPLC-AEC assay (see also Extended Data Fig. 4d) and (ii) that polymers eluting at later retention times from the AEC column are too large to migrate into the gel to a detectable extent. Interestingly, $Cps3D_{94-1138}$ (lacking α4HB) produced long chains reproducibly (three out of three experimental repeats, one repeat shown in Fig. 6c), suggesting that the presence of α4HB might have a negative impact on the stimulating function in the Cps3D dimer. In agreement with that, previous reports demonstrated that the removal of domains required for *in vivo* function (incl. membrane association) improved the *in vitro* performance of capsule polymerases[15–17].

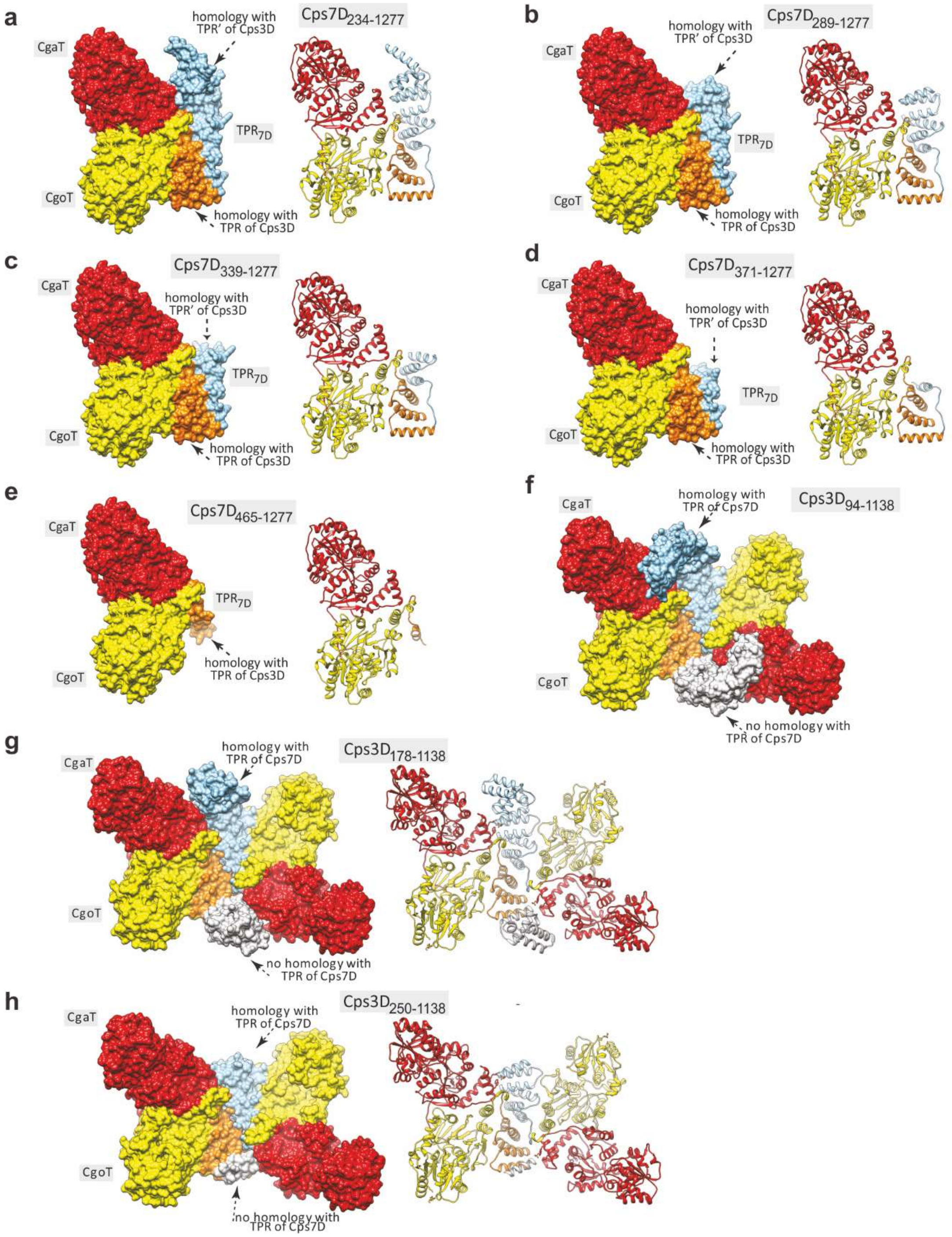

Supplementary Fig. 14 | Three dimensional models of the Cps7D and Cps3D truncations generated in this study. Color code: red, CgaT; yellow, CgoT; orange, homologous TPR repeats from monomeric Cps7D (α16-α20) and protomer 1 of the Cps3D dimer (α11-α15); light blue, homologous TPR repeats from monomeric Cps7D (α1-α15) and protomer 2 of the Cps3D dimer (α1'-α15'). See also Fig. 6 and Extended Data Fig. 8. **a-e,** Truncations of Cps7D as indicated based on Cps7D$_{AF}$. **f-h**, Truncations of Cps3D as indicated and based on the dimeric crystal structure of Cps3D. The TPR(') domain is required for the elongation of poly(Gro3P) (Fig. 6) and, according to the crystal structure of Cps3D, appears to be in close vicinity to CgaT(NT) (Fig. 5), which transfers the first galactose onto poly(Gro3P). TPR(') and CgaT(NT) interact through the following structural elements: (i) the TPR α2 (residues 128-141) interacts with the α42-α43 loop (residues 900 and 901) of CgaT', (ii) the TPR α3-α4 loop (residues 159-

161) interacts with α42 (residues 893-899) and α43 (residues 902-913) of CgaT’, (iii) the end of TPR α5 (residues 180-193) interacts with the α39-α40 loop (residues 832-836) of CgaT’, (iv) the end of TPR α8 (residues 221-228) interacts with α39 (residues 819-831) of CgaT’, (v) the beginning of TPR α9 (residues 231-245) together with the TPR α10-α11 loop (residues 263-265) interact with the α39-α40 loop (residues 832-836), (vi) the α10-α11 loop also interacts with the beginning of α37 (residues 786-790) and α39 (residues 819-831) of CgaT’, (vii) the TPR α12 (residues 285-297) interacts with α39 of CgaT' and with the α15-α16 loop (residues 347-356) of the opposite TPR’ domain, (ix) TPR α14 (residues 320-332) interacts with α15 (residues 336-346) and the α15-α16 loop (residues 279-284) of TPR’, (x) TPR α15 interacts with α14 of TPR’, and (xi) the TPR α15-α16 loop interacts with α10, α14 and α15 of TPR’.

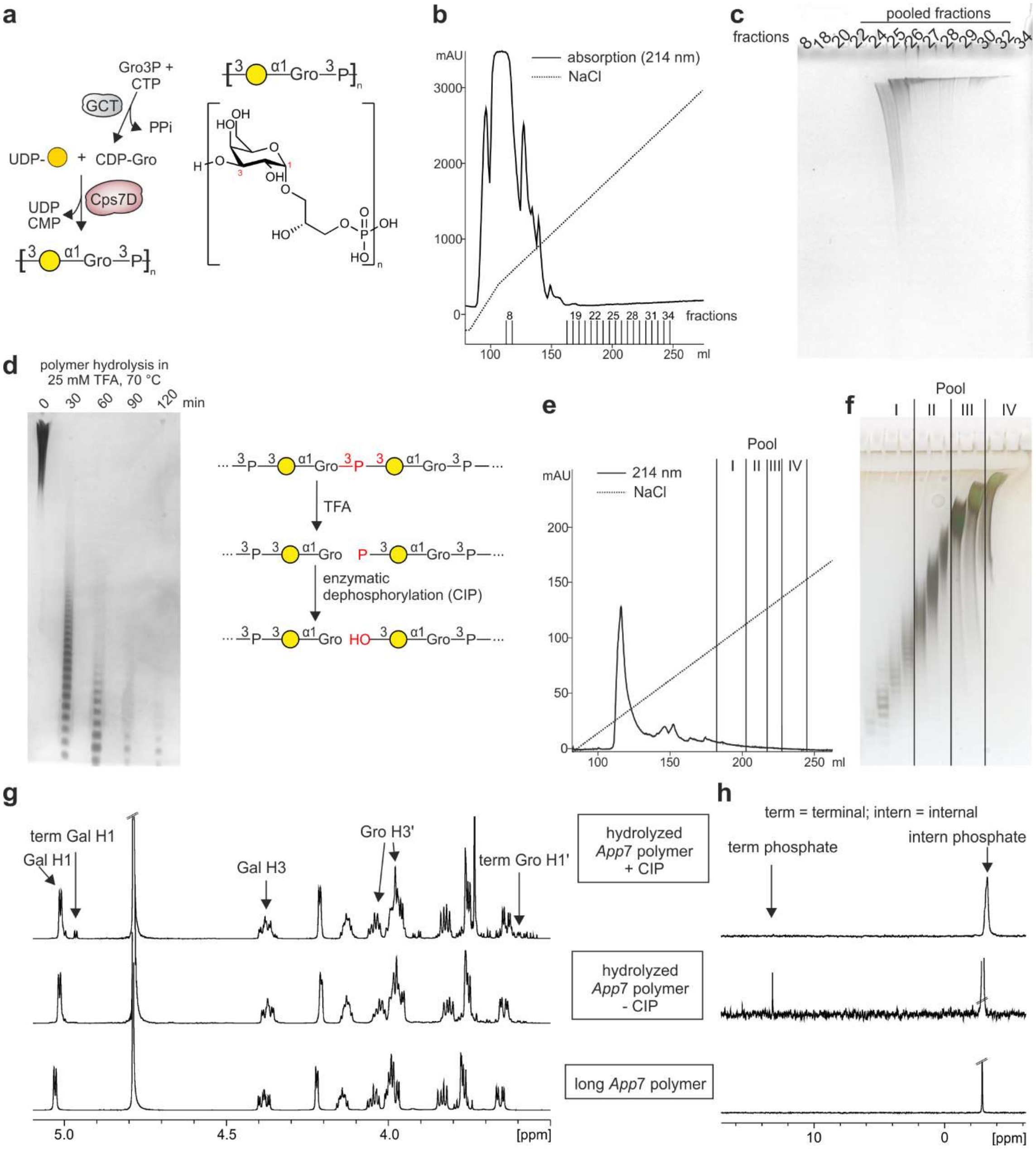

Supplementary Fig. 15 | Generation of App7 capsule polymer fragments. a, Scheme of the App7 capsule polymer backbone synthesis catalysed by the polymerase Cps7D and GCT from the substrates UDP-Gal, CTP and Gro3P as previously published[1]. **b,** Purification of App7 polymer backbone via anion-exchange chromatography. **c**, Alcian blue/silver-stained polyacrylamide gel of collected fractions to visualize the UV-inactive polymer. **d,** Alcian blue/silver-stained polyacrylamide gel of samples taken during hydrolysis of the App7 polymer backbone (TFA and at 70°C). Schematic overview of the acidic polymer hydrolysis followed by calf intestinal phosphatase (CIP) treatment to remove terminal phosphate groups. **e,** Purification of the hydrolyzed and CIP-treated App7 polymer via anion-exchange chromatography. **f**, Visualization of the UV-inactive App7 polymer fragments using PAGE followed by Alcian blue/silver-staining. **g,** ^{1}H NMR and **h,** ^{31}P NMR analyses demonstrated that the integrity of internal repeating units was not altered during acidic hydrolysis and that phosphomonoesters present at the termini of the hydrolyzed fragments could by successfully removed by CIP treatment. A comprehensive 2D NMR analysis revealed a galactose and a glycerol at the non-reducing and reducing

ends of the final products, respectively (see also panel g, top spectrum). The App3 backbone was hydrolyzed accordingly with similar results. Chemical shift values for both polymers are shown in Supplementary Table 5.

Supplementary Fig. 16 | Sequence of Cps1A as determined in this study.

DNA sequence

ATGAATAGAAAATTTTCTAAGTTACTAAAAAATCCACATATTTTTTTTAGGGATTTTCTAAATAAAAAGTACCCT
ATAAAAAATACGGAACTTCCCTTCTCAGAATCTGAAGAAGCTAACTTAATAGAAGCAAACCAAAAATTAGATA
AGATTATCCAAAAGAATACGTTGCAACAAACTAATATTGATGTGGTATTTACTTGGGTAGATGGTTCTGATCCT
TCATGGCAAGCTAAATATTCCCAATATGCACCAAATTATCAAGCGAAATCCGCTCTATATGCAACGGATATCGC
CCGATTTGAAGATCATAATGAATTATATTATTCAGTACATGCTGTACTTAAATATATGCCTTGGGTTAGGCATAT
ATTTATTATAACAGATAATCAAAAGCCAAAGTGGCTGGATGAGACGAGACAAGAAAAAATTACACTAATCGAT
CATCAAGATATTATAGATAAAGAATATCTTCCAACGTTTAATTCCCATGTTATTGAAGCATTTTTACATAAAATT
CCTAATTTAAGCGAGAATTTTATCTATTTTAATGATGATGTTTTTATTGCACGAGAACTACAAGCTGAACACTTT
TTCCAAGCAAATGGTATTGCCTCTATATTTATGTCGGAAAAAAGCCTCACTCAAATGCGTAACAGAGGAACTAT
TACACCGACTCTTTCTGCTTCGGAATATAGTATTCGCTTACTAAACAAATATTACAATACAAATATTGACTCACC
ACTTGTACACACTTATATCCCATTGAAAAAAAGTATGTATGAATTGGCATGGCGGCGTTATGAGAAAGAAATTC
TTGGATTTTTACCCAATAAATTtAGAACAAATAACGATTTAAATTTTGCAAACTTTCTTATTCCTTGGTTAATGTA
TTTCGAAGGGAAAGCAATGCCTAAAATAGATATTTGTTATTATTTTAATATTAGATCTCCAAATGCACTTACACA
ATATAAAAAACTTTTAAATAAAAAAAACATAGGCGAACAGCCTAATTCATTTTGCGCAAATGATTTTAATAGTC
AAAAAAGTATTAACAACTATCAAAATCAATTGTTTTCTTTT

Amino acid sequence

MNRKFSKLLKNPHIFFRDFLNKKYPIKNTELPFSESEEANLIEANQKLDKIIQKNTLQQTNIDVVFTWVDGSDPSWQA
KYSQYAPNYQAKSALYATDIARFEDHNELYYSVHAVLKYMPWVRHIFIITDNQKPKWLDETRQEKITLIDHQDIIDKE
YLPTFNSHVIEAFLHKIPNLSENFIYFNDDVFIARELQAEHFFQANGIASIFMSEKSLTQMRNRGTITPTLSASEYSIRLL
NKYYNTNIDSPLVHTYIPLKKSMYELAWRRYEKEILGFLPNKFRTNNDLNFANFLIPWLMYFEGKAMPKIDICYYFNI
RSPNALTQYKKLLNKKNIGEQPNSFCANDFNSQKSINNYQNQLFSF

Supplementary Figure 1a (left panel)

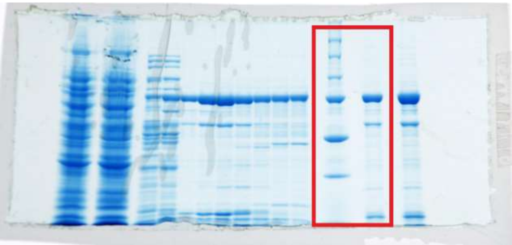

Supplementary Figure 1a (right panel)

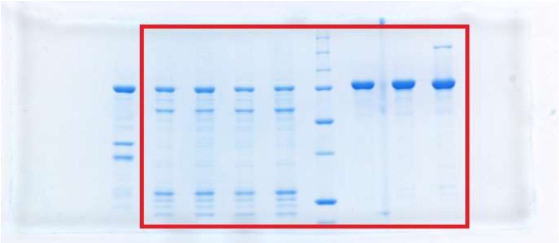

Supplementary Figure 1b

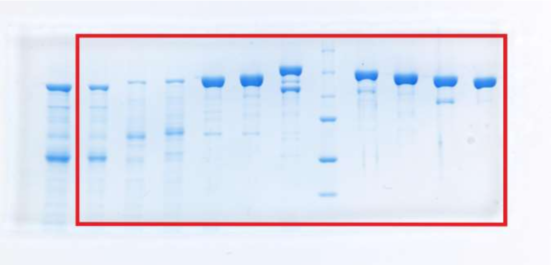

Supplementary Figure 1c

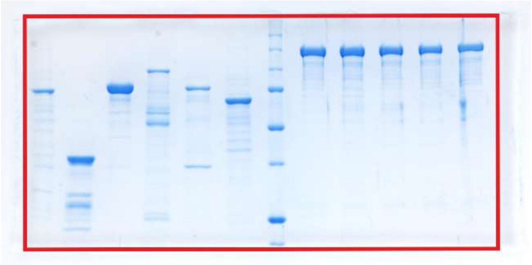

Supplementary Fig. 17 | Source Data for Supplementary Figure 1. Unprocessed gels.

Supplementary Figure 6a

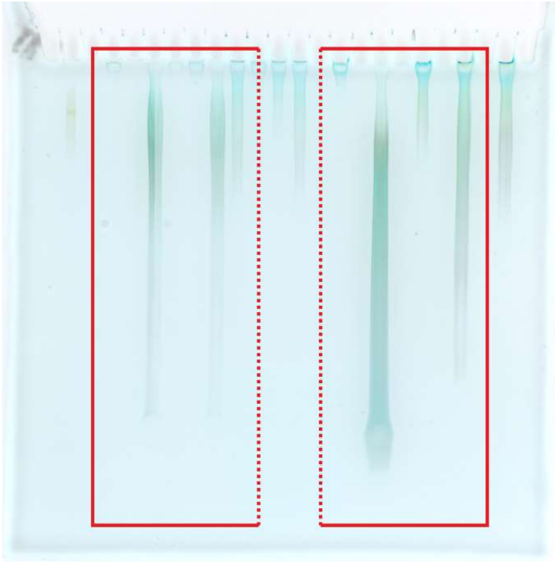

Supplementary Fig. 18 | Source Data for Supplementary Figure 6. Unprocessed gel.

Supplementary Figure 8c

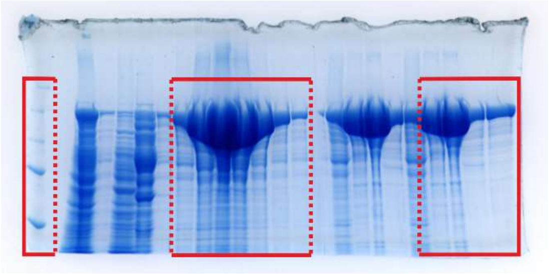

Supplementary Fig. 19 | Source Data for Supplementary Figure 8. Unprocessed gel.

Supplementary Figure 12c (left panel)

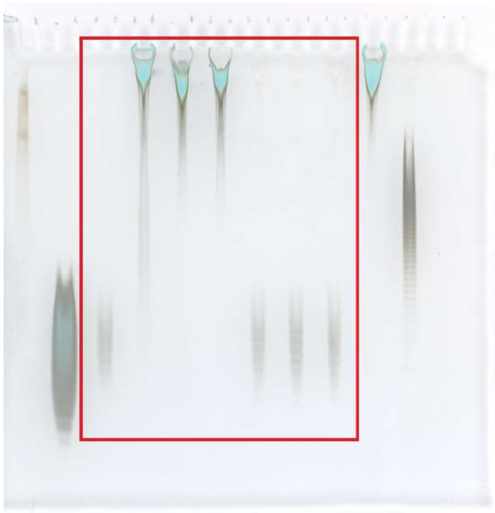

Supplementary Figure 12c (right panel)

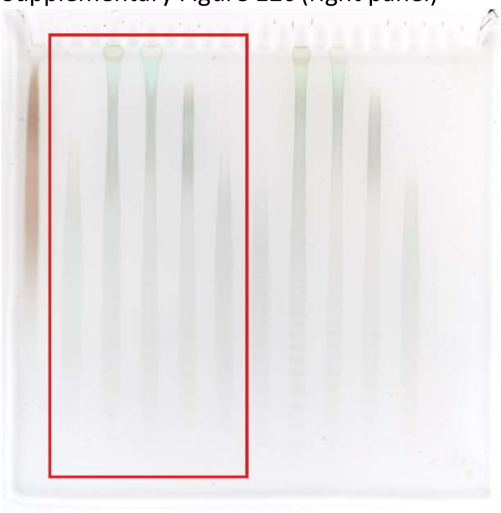

Supplementary Figure 12d (left panel)

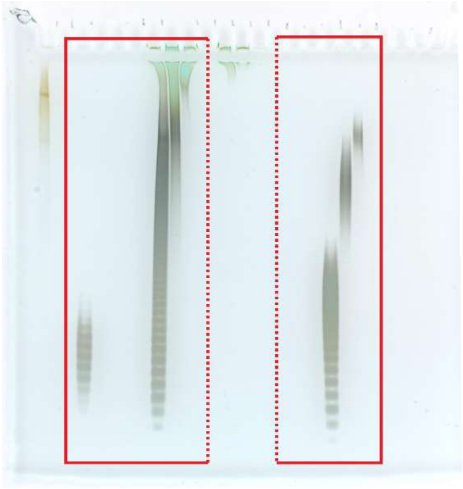

Supplementary Figure 12d (middle panel)

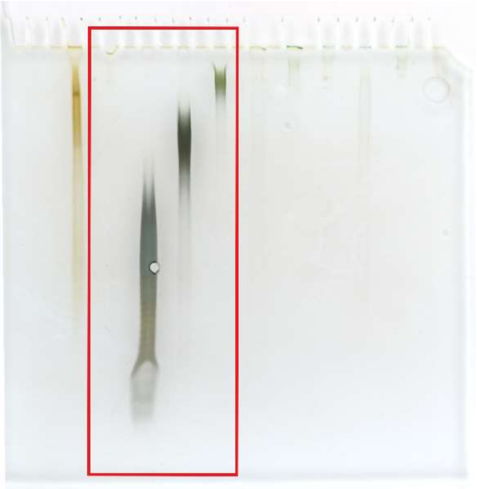

Supplementary Figure 12d (right panel)

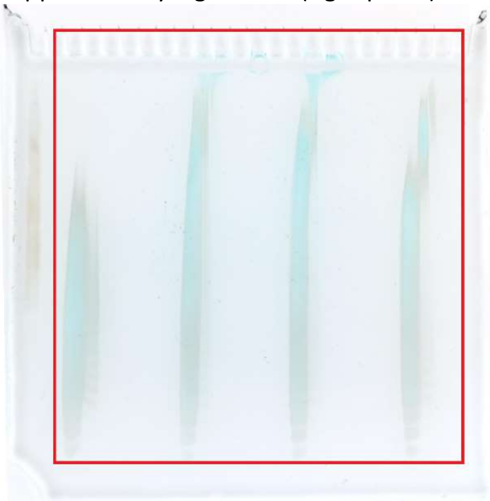

Supplementary Fig. 20 | Source Data for Supplementary Figure 12. Unprocessed gels.

Supplementary Figure 13c

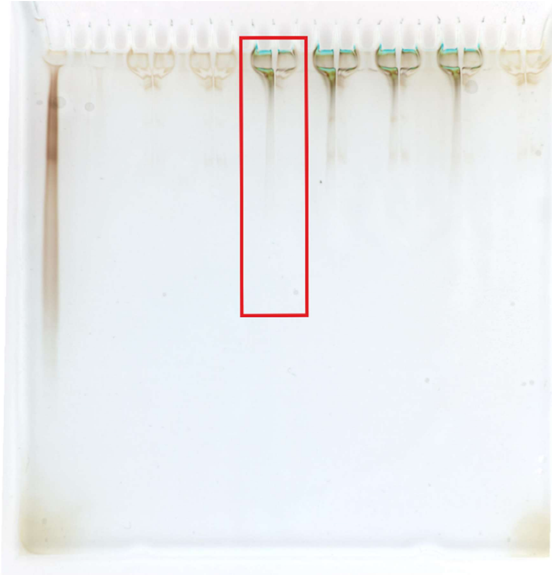

Supplementary Figure 13d (left panel)

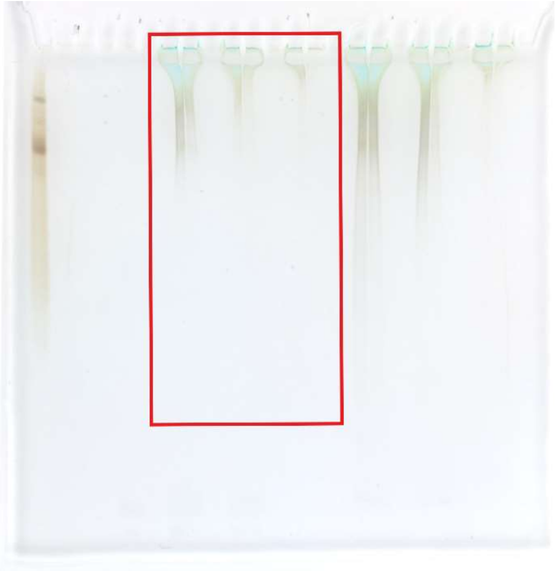

Supplementary Figure 13d (middle panel)

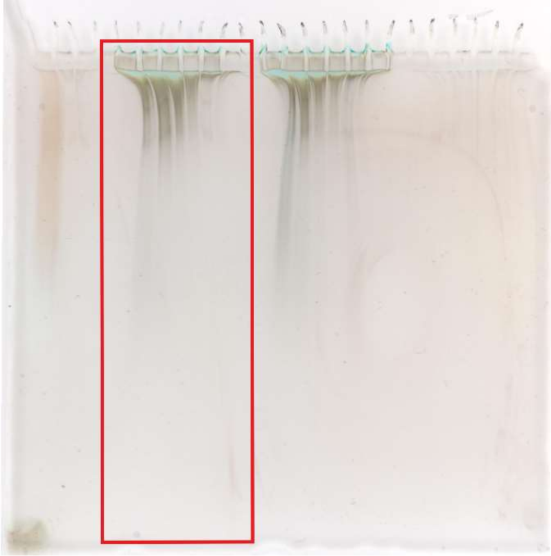

Supplementary Figure 13d (right panel)

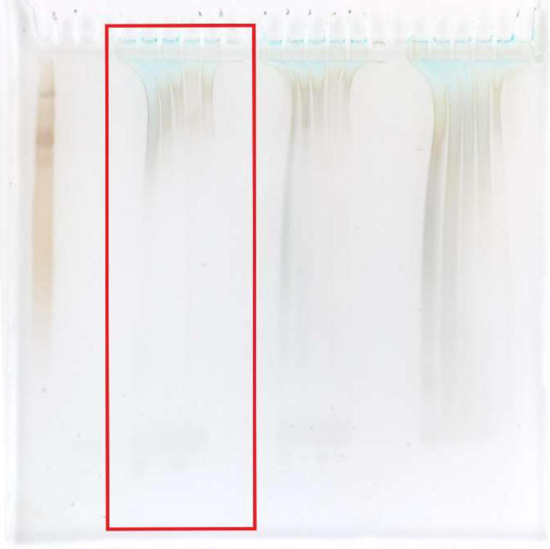

Supplementary Fig. 21 | Source Data for Supplementary Figure 13. Unprocessed gels.

Supplementary Figure 15c
Colors were adjusted equally across the entire image to improve the visualization of the Alcian blue/silver staining

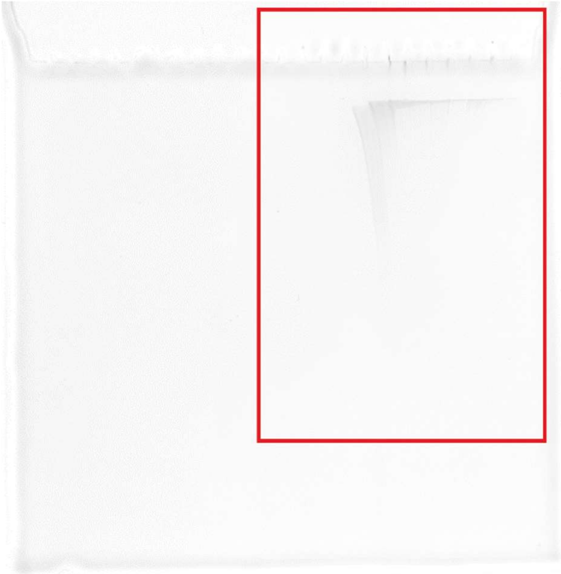

Supplementary Figure 15d

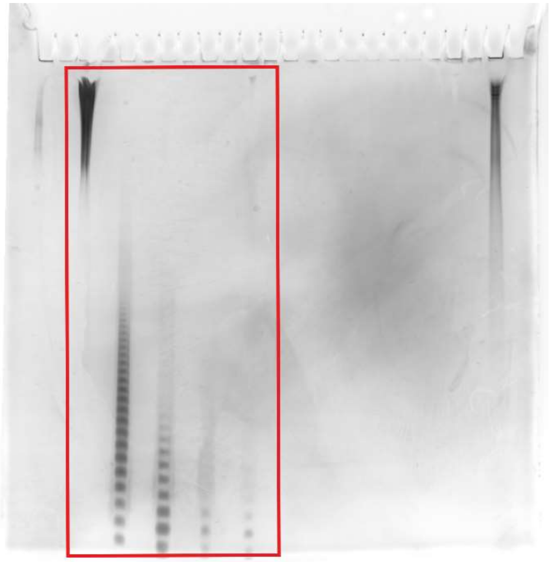

Supplementary Figure 15f

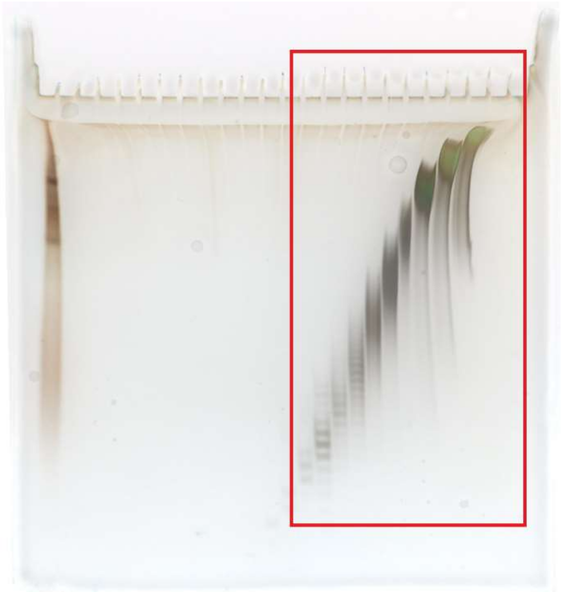

Supplementary Fig. 22 | Source Data for Supplementary Figure 15. Unprocessed gels.

Supplementary Note

Chemical Synthesis of compounds 6 and 7

General information

All reagents were of commercial grade and used as received. All moisture sensitive reactions were performed under an argon atmosphere. DCM used in the glycosylation reactions was dried with flame dried 4Å molecular sieves before being used. Reactions were monitored by TLC analysis with detection by UV (254 nm) and where applicable by spraying with 20% sulfuric acid in EtOH or with a solution of $(NH_4)_6Mo_7O_{24}\cdot 4H_2O$ (25 g/L) and $(NH_4)_4Ce(SO_4)_4\cdot 2H_2O$ (10 g/L) in 10% sulfuric acid (aq.) followed by charring at ~150 °C. Flash column chromatography was performed on silica gel (40-63µm). ^1H and ^{13}C spectra were recorded on a Bruker AV 400 and Bruker AV 500 in $CDCl_3$ or D_2O. Chemical shifts (δ) are given in ppm relative to tetramethylsilane as internal standard (^1H NMR in $CDCl_3$) or the residual signal of the deuterated solvent. Coupling constants (*J*) are given in Hz. NMR spectra are combined in Supplementary Fig. 28 at the end of this Supplementary note. All ^{13}C spectra are proton decoupled. NMR peak assignments were made using COSY and HSQC experiments. Where applicable Clean TOCSY, HMBC and GATED experiments were used to further elucidate the structure. The anomeric product ratios were analyzed through integration of proton NMR signals. Column chromatography was carried out using silica gel (0.040-0.063 mm). Size-exclusion chromatography was carried out using Sephadex LH-20.

Synthesis of glycerol building block

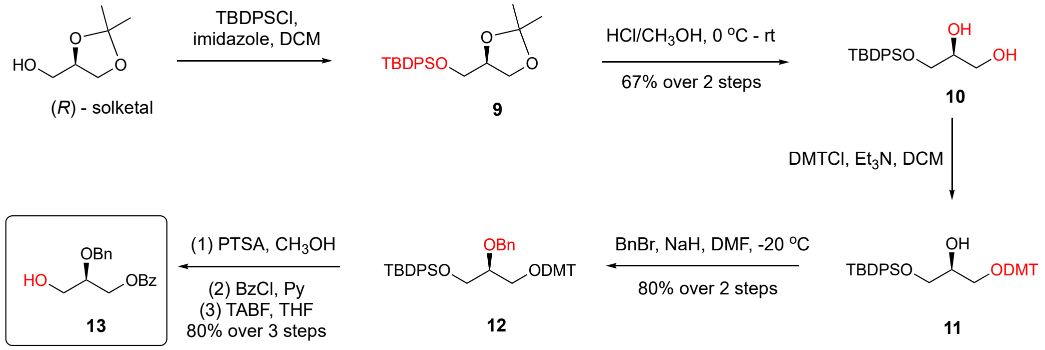

Supplementary Fig. 23 | Synthesis of glycerol building block 13.

To a solution of (*R*)-solketal (5 g, 37.88 mmol) in 50 mL DCM was added imidazole (2.5 eq) at room temperature, followed by the addition of TBDPSCl (1.2 eq) at rt. The resulting mixture was stirred at rt overnight until TLC indicated the complete conversion of starting material (pentane/ethyl acetate = 4/1, R_f = 0.5). The reaction was washed with 1 M HCl solution (50 mL), and the organic phase was concentrated *in vacuo*. The obtained residue **9** was dissolved in 50 mL methanol followed by addition of 2 drops of concentrated HCl at 0 °C, the reaction was allowed to warm to room temperature and stirred at this temperature for 30 min until TLC indicated the complete conversion of starting material (pentane/acetone = 4/1, R_f = 0.3). The solvent was evaporated, the given residue **10** was then dissolved in 50 mL DCM, followed by the addition of Et_3N (1.5 eq) and DMTCl (1 eq) at 0 °C, the reaction was stirred at room temperature for 3 h until TLC indicated the complete conversion of starting material (pentane/ethyl acetate = 4/1, R_f = 0.25). Then the reaction mixture was washed with sat. $NaHCO_3$ (50 mL) and brine (50 mL), the organic layer was dried over anhydrous Na_2SO_4, concentrated *in vacuo*. The afforded crude compound **11** was dissolved in 50 mL DMF, BnBr (1.5 eq) and NaH (1.0 eq) were added at -20 °C, the benzylation was completed in 2 h (pentane/ethyl acetate = 10/1, R_f = 0.6), then the reaction was quenched by methanol, diluted in ethyl acetate (50 mL) and washed with brine (50 mL). The compound **12** was purified using chromatography column (pentane/ethyl acetate = 15/1). Then compound **12** was dissolved in 20 mL methanol and catalytic amount of *p*-Toluenesulfonic acid was added, the reaction was stirred at room temperature for 20 min, then methanol was removed under reduced pressure, the given residue was dissolved in 20 mL pyridine and BzCl (1.0 eq) was added, the reaction was stirred at room temperature for 2 h until completion (pentane/ethyl acetate = 10/1, R_f = 0.6), then pyridine was removed, the residue was dissolved in ethyl acetate (50 mL) and washed with 1 M HCl solution (50 mL), sat. $NaHCO_3$ solution (50 mL) and brine (50 mL), the organic layer was dried over anhydrous Na_2SO_4, concentrated *in vacuo* and dissolved in 50 mL THF, followed by the addition of TBAF (1.0 eq, 1. 0 M solution in THF). The reaction was stirred at room temperature for 1 h until TLC indicated the complete conversion of starting material (pentane/ethyl acetate = 4/1, R_f = 0.4). The reaction was diluted in ethyl acetate (50 mL) and washed with sat. NH_4Cl solution (50 mL) and brine (50 mL), the organic layer was dried over anhydrous Na_2SO_4, concentrated *in vacuo* and purified using chromatography column (pentane/ethyl acetate = 4/1) to give compound **13** (4.6 g) as a colorless oil. ^1H NMR (500 MHz, $CDCl_3$) δ 8.09 – 8.04 (m, 2H, ArH), 7.59 (s, 1H, ArH), 7.47 (dd, *J* = 8.1, 7.4 Hz, 2H, ArH), 7.41 – 7.28 (m, 5H, ArH), 4.78 (d, *J* = 11.7 Hz, 1H, CH Bn), 4.69 (d, *J* = 11.8 Hz, 1H, CH Bn), 4.56 – 4.45 (m, 2H, CH_2OBz), 3.88 (dd, *J* = 5.4, 4.2 Hz, 1H, CH), 3.84 – 3.79 (m, 1H, CH_2OH), 3.75 (dd, *J* = 11.8, 5.6 Hz, 1H, CH_2OH), 2.61 (br, 1H, OH). ^{13}C NMR (126 MHz, $CDCl_3$) δ 166.55, 137.90, 133.21, 129.83, 129.67, 128.55, 128.46, 127.97, 127.92, 77.34 (CH), 72.24 (CH_2), 63.59 (CH_2), 62.03 (CH_2) (Supplementary Fig. 28).

Synthesis of App7 monomer and dimer

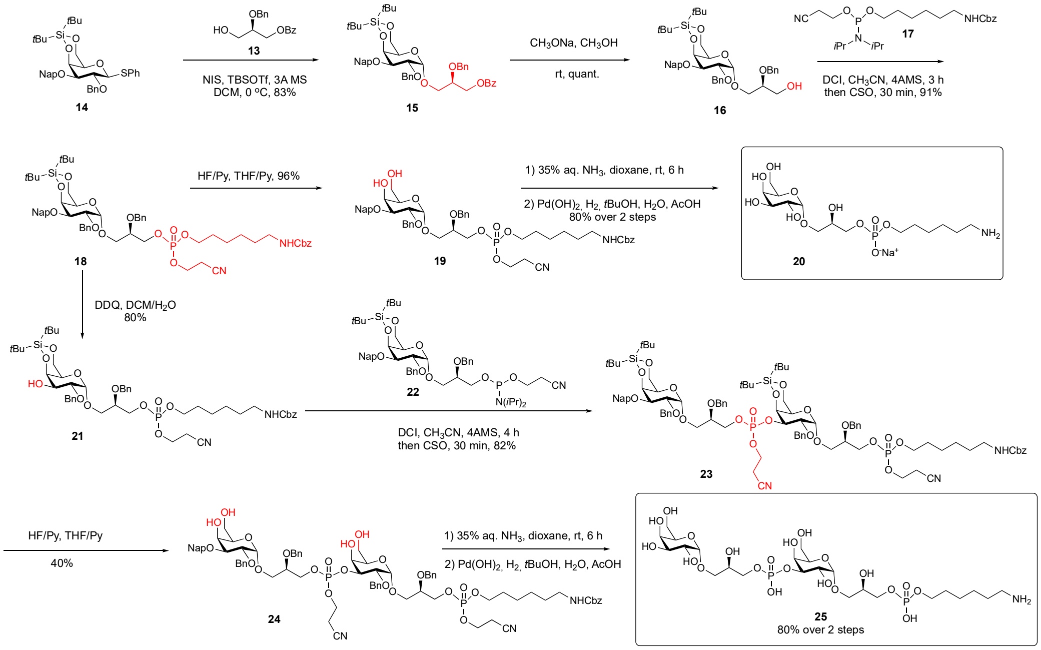

Supplementary Fig. 24 | Synthesis of App*7* monomer 20 and dimer 25.

Synthesis of compound **15**

Donor **14** (1.6 g, 2.5 mmol) (Supplementary Fig. 29) and acceptor **13** (650 mg, 2.273 mmol) were co-evaporated with toluene 3 times, then diluted in 15 mL anhydrous DCM, followed by addition of 4A molecule sieves, NIS (1.02 g, 4.546 mmol) and TBSOTf (120 mg, 0.455 mmol) at 0 °C. The resulting dark purple mixture was stirred at 0 °C for 2 h until TLC indicated the complete conversion of starting material (pentane/ethyl acetate = 10/1, R_f = 0.5). The reaction was quenched by Na_2S_2O3, diluted in DCM (30 mL), washed with sat. $NaHCO_3$ (50 mL) and brine (50 mL). The organic layer was dried over anhydrous Na_2SO_4, concentrated *in vacuo* and purified by silica gel column chromatography (pentane/ethyl acetate = 12/1, 1.54 g, 83%). ^{1}H NMR (400 MHz, $CDCl_3$) δ 8.01 (dd, *J* = 8.4, 1.4 Hz, 2H, ArH), 7.88 – 7.74 (m, 4H, ArH), 7.61 – 7.53 (m, 2H, ArH), 7.47 – 7.34 (m, 6H, ArH), 7.30 – 7.14 (m, 8H, ArH), 4.92 – 4.85 (m, 3H, $ArCH_2$, OCH_2), 4.77 (d, *J* = 3.5 Hz, 1H, H1), 4.70 (dd, *J* = 11.7, 6.6 Hz, 2H, OCH_2), 4.57 – 4.47 (m, 2H, CH_2OBz), 4.41 (dd, *J* = 3.1, 1.1 Hz, 1H, H4), 4.36 (dd, *J* = 11.7, 5.6 Hz, 1H, CH_2OBz), 4.05 – 3.92 (m, 4H, H2, H5, CH, $ArCH_2$), 3.85 (dd, *J* = 10.0, 3.0 Hz, 1H, H3), 3.77 (dd, *J* = 10.6, 6.6 Hz, 1H, H6), 3.64 (dd, *J* = 10.6, 4.6 Hz, 1H, H6), 3.58 (q, *J* = 1.6 Hz, 1H, $ArCH_2$), 1.05 (s, 9H, *t*Bu), 1.00 (s, 9H, *t*Bu). ^{13}C NMR (101 MHz, $CDCl_3$) δ 166.37, 138.68, 138.20, 136.61, 133.40, 133.22, 133.09, 130.01, 129.76, 128.53, 128.46, 128.43, 128.36, 128.20, 127.98, 127.91, 127.78, 126.33, 126.15, 125.96, 125.88, 98.32, 77.43, 76.21, 74.44, 73.74, 72.64, 71.35, 71.27, 67.95, 67.41, 67.12, 64.05, 27.79, 27.42, 23.52, 20.76. ESI HRMS (m/z): $[M + Na]^+$ calcd for $C_{49}H_{58}O_9SiNa$, 841.3748; found 841.3746 (Supplementary Fig. 30).

Synthesis of compound **16**

Compound **15** (1.4 g, 1.71 mmol) was dissolved in 10 ml methanol, 0.5 ml CH_3ONa (4.5 M solution in methanol) was added. The reaction was stirred at room temperature for 1 h until TLC indicated the complete conversion of starting material (pentane/ethyl acetate = 4/1, R_f = 0.4). The reaction was neutralized by AcOH, solvent was removed under reduced pressure, purification by chromatography column to afford compound **16** as a white solid (1.0 g, quant.). ^{1}H NMR (500 MHz, $CDCl_3$) δ 7.95 – 7.92 (m, 1H), 7.90 – 7.81 (m, 3H), 7.65 (dd, *J* = 8.4, 1.7 Hz, 1H), 7.56 – 7.43 (m, 4H), 7.41 – 7.24 (m, 8H), 5.01

– 4.92 (m, 3H), 4.86 (d, *J* = 3.7 Hz, 1H), 4.80 (d, *J* = 11.8 Hz, 1H), 4.68 (d, *J* = 11.7 Hz, 1H), 4.61 (d, *J* = 11.7 Hz, 1H), 4.49 (dd, *J* = 3.1, 1.1 Hz, 1H), 4.14 – 4.05 (m, 3H), 3.90 (dd, *J* = 10.0, 3.0 Hz, 1H), 3.87 – 3.70 (m, 4H), 3.65 – 3.54 (m, 2H), 2.08 (s, 1H), 1.13 (s, 9H), 1.09 (s, 9H). ^{13}C NMR (126 MHz, $CDCl_3$) δ 174.45, 138.31, 138.19, 136.36, 133.26, 132.97, 128.36, 128.10, 127.86, 127.76, 127.70, 127.68, 126.23, 126.05, 125.81, 125.79, 77.87, 77.30, 74.17, 73.67, 72.10, 71.18, 71.07, 68.68, 67.29, 67.02, 62.57, 27.67, 27.31, 23.40, 20.66. ESI HRMS (m/z): $[M + Na]^+$ calcd for $C_{42}H_{54}O_8SiNa$, 737.3486; found 737.3485 (Supplementary Fig. 31).

Synthesis of compound **17** refers to the previously reported[18].

Synthesis of compound **18**

Compound **16** (237 mg, 0.333 mmol) and compound **17** (300 mg, 0.665 mmol) were co-evaporated with toluene for 3 times followed by adding 4A molecule sieves, the mixture was dissolved in 2 mL anhydrous acetonitrile and stirred at room temperature for 15 min. DCI (2.67 mL, 0.25 M solution in ACN) was added to the mixture, the reaction was stirred at room temperature for 3 h until TLC indicated the complete conversion of starting material (pentane/ethyl acetate = 4/1, R_f = 0.5). Then the reaction was cooled to 0 °C, and CSO (1.33 mL, 0.5 M solution in ACN) was added, the mixture was stirred at 0 °C for 30 min until TLC indicated the complete conversion of P(III) to P(V) (pentane/acetone = 3/1, R_f = 0.3). The precipitate was filtered off, the filtrate was diluted in 30 mL ethyl acetate and washed with brine (30 mL). The organic layer was dried over anhydrous Na_2SO_4, concentrated *in vacuo* and purified by silica gel column chromatography (pentane/ethyl acetate = 1/1, 326 mg, 91%). ^{1}H NMR (500 MHz, $CDCl_3$) δ 7.88 – 7.79 (m, 4H), 7.78 – 7.73 (m, 1H), 7.57 (dd, *J* = 8.5, 1.7 Hz, 2H), 7.49 – 7.42 (m, 2H), 7.39 – 7.17 (m, 14H), 5.08 (s, 2H), 4.93 – 4.82 (m, 4H), 4.80 (d, *J* = 3.6 Hz, 1H), 4.70 (d, *J* = 11.8 Hz, 1H), 4.63 (dd, *J* = 11.4, 1.4 Hz, 1H), 4.53 (dd, *J* = 11.4, 3.9 Hz, 1H), 4.47 – 4.42 (m, 1H), 4.24 (m, 1H), 4.15 – 3.97 (m, 8H), 3.86 – 3.79 (m, 2H), 3.70 (ddd, *J* = 10.8, 6.1, 1.6 Hz, 1H), 3.62 – 3.53 (m, 2H), 3.15 (q, *J* = 6.7 Hz, 2H), 2.59 – 2.47 (m, 2H), 1.63 (p, *J* = 6.7 Hz, 2H), 1.47 (p, *J* = 7.3 Hz, 2H), 1.39 – 1.19 (m, 4H), 1.10 – 0.95 (m, 18H). ^{13}C NMR (126 MHz, $CDCl_3$) δ 156.50, 138.65, 138.62, 137.98, 137.92, 136.74, 136.52, 133.36, 133.05, 128.60, 128.47, 128.45, 128.28, 128.25, 128.17, 127.95, 127.92, 127.87, 127.78, 126.27, 126.16, 125.90, 98.48, 77.35, 76.79, 76.77, 76.74, 76.71, 74.42, 73.69, 73.67, 72.58, 71.19, 71.17, 71.05, 71.03, 68.43, 68.38, 68.33, 67.42, 67.11, 67.07, 67.02, 66.96, 66.94, 66.92, 66.89, 66.64, 61.84, 61.80, 61.76, 40.92, 30.11, 30.06, 29.84, 27.76, 27.39, 26.17, 25.05, 23.50, 20.74, 19.60, 19.58, 19.55, 19.52. ^{31}P NMR (202 MHz, $CDCl_3$) δ 86.29, -1.29, -1.44. ESI HRMS (m/z): $[M + Na]^+$ calcd for $C_{59}H_{77}O_{13}N_2PSiNa$, 1103.4830; found 1103.4828 (Supplementary Fig. 32).

Synthesis of compound **19**

Compound **18** (110 mg, 0.1 mmol) was dissolved in 3 ml THF/Pyridine (1/1, v/v), followed by the addition of HF/Pyr (0.407 mmol, 11 uL) at 0 °C, the reaction mixture was allowed to stir at rt for 3 h until TLC indicated the complete conversion of starting material (DCM/ACE = 4/1, R_f = 0.15). The reaction was quenched by sat. $NaHCO_3$ and diluted in ethyl acetate (20 mL), the organic phase was washed with sat. $NaHCO_3$, brine, and dried over anhydrous Na_2SO_4, concentrated *in vacuo* and purified by flash column (DCM/methanol = 20/1, 84 mg, 96%). ^{1}H NMR (400 MHz, $CDCl_3$) δ 7.79 (ddt, *J* = 15.0, 9.4, 3.4 Hz, 4H), 7.47 (td, *J* = 5.7, 2.5 Hz, 3H), 7.37 – 7.21 (m, 15H), 5.07 (s, 2H), 4.93 – 4.73 (m, 5H), 4.68 – 4.56 (m, 3H), 4.30 (m, 1H), 4.16 – 3.97 (m, 6H), 3.93 – 3.81 (m, 3H), 3.80 – 3.67 (m, 4H), 3.63 (d, *J* = 1.1 Hz, 1H), 3.13 (d, *J* = 6.6 Hz, 2H), 2.56 – 2.47 (m, 2H), 1.62 (t, *J* = 7.1 Hz, 2H), 1.49 – 1.37 (m, 2H), 1.37 – 1.25 (m, 4H). ^{13}C NMR (101 MHz, $CDCl_3$) δ 156.51, 138.48, 138.45, 137.91, 137.86, 136.71, 135.59, 135.57, 133.27, 133.07, 128.57, 128.50, 128.37, 128.14, 127.93, 127.90, 127.87, 127.78, 126.60, 126.31, 126.13, 125.80, 125.78, 116.67, 116.59, 98.28, 98.19, 77.48, 77.21, 77.16, 76.84, 76.52, 76.48,

76.45, 76.42, 75.83, 73.30, 73.28, 72.69, 72.37, 69.67, 68.70, 68.67, 68.51, 68.45, 68.38, 67.20, 67.13, 66.96, 66.87, 66.61, 62.71, 61.90, 61.86, 61.81, 40.88, 30.06, 29.99, 29.78, 29.32, 26.11, 25.00, 19.58, 19.54, 19.50, 19.47. ^{31}P NMR (162 MHz, $CDCl_3$) δ -0.08, -0.24. ESI HRMS (m/z): $[M + Na]^+$ calcd for $C_{51}H_{61}O_{13}N_2PSiNa$, 963.3809; found 963.3806 (Supplementary Fig. 33).

Synthesis of compound **20**

To a solution of substrate **19** (84 mg, 0.09 mmol) in 0.5 mL dioxane was added conc. ammonia (0.5 mL). The resulting mixture was sealed and stirred at rt for 4 h until TLC indicated the complete conversion of starting material (DCM/methanol = 10/1, R_f = 0.12). The solvent and ammonia were evaporated, and the resulting residue was dissolved in *t*BuOH and water (6 mL, 1/1, v/v), followed by the addition of 1 drop acetic acid and $Pd(OH)_2/C$ (100 mg). The reaction was purged with a hydrogen balloon and stirred at rt overnight until completion. Then palladium was filtered, the given filtrate was evaporated. The crude product was purified using size-exclusion column (LH-20) giving 25 mg of the product. Ion exchange with Dowex-Na provided the sodium salt (23 mg). ^{1}H NMR (500 MHz, D_2O) δ 4.91 (d, *J* = 3.8 Hz, 1H), 4.03 (m, 1H), 3.97 – 3.74 (m, 9H), 3.73 – 3.63 (m, 2H), 3.55 (dd, *J* = 10.6, 3.7 Hz, 1H), 3.00 – 2.92 (m, 2H), 1.68 – 1.57 (m, 4H), 1.38 (q, *J* = 3.7 Hz, 4H). ^{13}C NMR (126 MHz, D_2O) δ 98.37, 71.01, 69.48, 69.22, 69.11, 69.04, 68.49, 67.97, 66.14, 66.10, 65.94, 65.90, 61.17, 39.39, 29.47, 29.42, 26.57, 25.11, 24.42. ^{31}P NMR (202 MHz, D_2O) δ 0.72. ESI HRMS (m/z): $[M + H]^+$ calcd for $C_{15}H_{33}NO_{11}P$, 434.1791; found 434.1792 (Supplementary Fig. 34).

Synthesis of compound **21**

To a solution of substrate **20** (147 mg, 0.136 mmol) in 5 mL DCM was added 0.5 mL water, followed by the addition of DDQ (46 mg, 0.203 mmol). The resulting mixture was stirred at rt for 2 h until TLC indicated the complete conversion of starting material (pentane/acetone = 7/3, R_f = 0.5). The reaction was quenched by sat. $Na_2S_2O_3$ and the mixture was washed with sat. $NaHCO_3$, and brine. The organic layer was dried over anhydrous Na_2SO_4, concentrated *in vacuo* and purified by flash column (pentane/acetone = 2/1, 93 mg, 73%). ^{1}H NMR (500 MHz, $CDCl_3$) δ 7.39 – 7.23 (m, 15H), 5.08 (s, 2H), 4.95 – 4.88 (m, 1H), 4.83 – 4.74 (m, 2H), 4.72 – 4.65 (m, 2H), 4.60 (dd, *J* = 11.5, 3.8 Hz, 1H), 4.35 – 4.30 (m, 1H), 4.30 – 4.20 (m, 1H), 4.19 – 3.99 (m, 7H), 3.94 – 3.79 (m, 2H), 3.75 – 3.62 (m, 3H), 3.54 (ddd, *J* = 10.6, 4.7, 2.7 Hz, 1H), 3.16 (d, *J* = 6.6 Hz, 2H), 2.65 – 2.50 (m, 3H), 1.70 – 1.60 (m, 2H), 1.53 – 1.41 (m, 2H), 1.39 – 1.22 (m, 4H), 1.02 (s, 9H), 0.95 (s, 9H). ^{13}C NMR (126 MHz, $CDCl_3$) δ 156.52, 138.21, 137.98, 137.93, 136.77, 128.61, 128.57, 128.54, 128.33, 128.20, 128.17, 128.03, 128.01, 127.99, 127.93, 116.63, 116.61, 97.96, 97.94, 76.84, 76.80, 76.74, 75.69, 73.58, 73.01, 72.68, 69.82, 68.47, 68.41, 68.37, 67.12, 67.03, 66.98, 66.93, 66.85, 66.64, 61.88, 61.84, 61.80, 40.93, 30.13, 30.08, 29.84, 27.63, 27.27, 26.19, 25.08, 23.41, 20.70, 19.68, 19.65, 19.62, 19.59. ^{31}P NMR (202 MHz, $CDCl_3$) δ -1.30, -1.45. ESI HRMS (m/z): $[M + Na]^+$ calcd for $C_{48}H_{69}N_2O_{13}PSiNa$, 963.4204; found 963.4205 (Supplementary Fig. 35).

Synthesis of compound **22**

Compound **16** (195 mg, 0.273 mmol) and 3-((bis(diisopropylamino)phosphaneyl)oxy)propanenitrile (165 mg, 0.546 mmol) were co-evaporated with toluene for 3 times followed by adding 4A molecule sieves, the mixture was dissolved in 2 mL anhydrous DCM and stirred at room temperature for 15 min. Tetrazolide salt (52 mg, 0.3 mmol) was added to the mixture, the reaction was stirred at room temperature for 2 h until TLC indicated the complete conversion of starting material (pentane/ethyl acetate = 3/1, R_f = 0.7). Then the mixture was washed with brine (30 mL). The organic layer was dried over anhydrous Na_2SO_4, concentrated *in vacuo* and purified by flash column (pentane/ethyl acetate = 5/1, eluted with 0.5% Et_3N, 257 mg, 91%). ^{1}H NMR (500 MHz, Acetone) δ 7.95 (s, 1H), 7.88 (dd, *J* = 8.9, 5.1 Hz, 2H), 7.82 – 7.77 (m, 1H), 7.61 (dd, *J* = 8.4, 1.7 Hz, 1H), 7.50 – 7.46 (m, 2H), 7.44 – 7.40 (m, 2H), 7.34 – 7.20 (m, 8H), 5.03 (t, *J* = 3.2 Hz, 1H), 4.99 – 4.92 (m, 1H), 4.86 (d, *J* = 12.3 Hz, 1H), 4.83 – 4.69 (m,

4H), 4.18 (d, J = 2.2 Hz, 1H), 4.08 – 4.00 (m, 2H), 3.97 – 3.73 (m, 11H), 3.72 – 3.58 (m, 5H), 2.84 (s, 1H), 2.79 (m, 3H), 2.73 – 2.65 (m, 2H), 1.24 – 1.11 (m, 15H), 1.07 (s, 9H), 1.01 (s, 9H). ^{13}C NMR (126 MHz, Acetone) δ 140.17, 140.07, 138.06, 134.32, 133.90, 129.02, 128.98, 128.69, 128.67, 128.63, 128.51, 128.47, 128.44, 128.13, 128.10, 126.87, 126.65, 126.62, 126.53, 98.81, 98.75, 78.96, 78.91, 78.89, 78.85, 78.18, 78.16, 75.61, 75.59, 73.25, 73.22, 72.67, 72.64, 71.79, 71.76, 71.00, 70.97, 68.21, 68.13, 68.03, 67.93, 63.97, 63.85, 63.77, 63.65, 59.69, 59.57, 59.54, 59.52, 59.42, 59.37, 43.90, 43.83, 43.80, 43.78, 43.73, 43.68, 29.84, 28.07, 27.88, 25.00, 24.95, 24.88, 24.82, 20.78, 20.72, 20.66. ^{31}P NMR (202 MHz, Acetone) δ 148.34, 148.27, 148.15. ESI HRMS (m/z): [M + Na]$^+$ calcd for $C_{51}H_{71}N_2O_9PSiNa$, 937.4564; found 937.4565 (Supplementary Fig. 36).

Synthesis of compound **23**

Compound **23** was synthetized by following the same procedure as for compound **18**. Substrate **21** (130 mg, 0.14 mmol), phosphoramidite **22** (291 mg, 0.24 mmol), DCI (1.28 mL, 0.25 M solution in ACN), CSO (0.64 mL, 0.5 M solution in ACN) and 2 mL anhydrous ACN were used for the reaction. The crude product was purified by silica gel column chromatography (pentane/acetone = 2/1, 201 mg, 82%). ^{1}H NMR (500 MHz, $CDCl_3$) δ 7.87 – 7.72 (m, 4H), 7.56 (m, 1H), 7.45 (m, 2H), 7.40 – 7.22 (m, 24H), 7.18 (m, 5H), 5.07 (s, 2H), 4.98 – 4.80 (m, 5H), 4.78 – 4.34 (m, 12H), 4.30 – 3.91 (m, 17H), 3.87 – 3.73 (m, 4H), 3.68 (s, 1H), 3.64 – 3.43 (m, 5H), 3.14 (q, J = 6.7 Hz, 2H), 2.64 – 2.52 (m, 3H), 2.46 (dt, J = 17.0, 6.2 Hz, 1H), 2.34 – 2.21 (m, 1H), 1.64 (t, J = 7.2 Hz, 2H), 1.46 (t, J = 7.2 Hz, 2H), 1.36 – 1.23 (m, 5H), 1.08 – 0.93 (m, 40H). ^{13}C NMR (126 MHz, $CDCl_3$) δ 156.44, 138.57, 138.10, 138.06, 137.78, 137.74, 137.72, 137.70, 137.67, 137.63, 136.71, 136.51, 136.49, 133.29, 132.98, 128.58, 128.53, 128.39, 128.32, 128.25, 128.23, 128.09, 128.00, 127.98, 127.94, 127.91, 127.88, 127.81, 127.71, 126.16, 126.08, 125.83, 125.81, 116.73, 116.71, 116.68, 116.60, 116.49, 98.27, 98.19, 97.68, 97.46, 77.31, 77.29, 76.84, 76.78, 76.59, 76.54, 76.49, 76.44, 74.29, 73.56, 73.54, 73.48, 73.42, 73.22, 73.19, 72.90, 72.88, 72.61, 72.58, 72.47, 72.43, 72.35, 72.24, 71.11, 70.98, 70.96, 68.38, 68.31, 68.26, 67.29, 67.23, 67.09, 67.02, 66.94, 66.89, 66.80, 66.67, 66.52, 66.32, 61.98, 61.93, 61.83, 61.81, 61.79, 40.84, 30.05, 29.99, 29.75, 29.30, 27.69, 27.61, 27.33, 27.29, 27.25, 26.09, 24.98, 23.41, 23.29, 20.67, 20.64, 19.61, 19.59, 19.56, 19.53, 19.50, 19.33, 19.27, 19.12, 19.06. ^{31}P NMR (202 MHz, $CDCl_3$) δ -1.17, -1.39, -2.25, -2.36 ESI HRMS (m/z): [M + Na]$^+$ calcd for $C_{93}H_{125}N_3O_{23}P_2Si_2Na$, 1792.7615; found 1792.7614 (Supplementary Fig. 37).

Synthesis of compound **24**

Compound **24** was synthetized by following the same procedure as for compound **19**. Substrate **23** (170 mg, 0.096 mmol), HF/Py (20 uL), THF/Py (4 mL, 1/1, v/v) were used. The compound was purified using silica gel column chromatography (DCM/Methanol = 30/1, 44 mg, 40%). ^{1}H NMR (400 MHz, $CDCl_3$) δ 7.83 – 7.72 (m, 4H), 7.54 – 7.42 (m, 3H), 7.39 – 7.11 (m, 26H), 5.07 (s, 2H), 5.01 – 4.71 (m, 6H), 4.70 – 4.48 (m, 7H), 4.44 – 3.95 (m, 13H), 3.94 – 3.45 (m, 16H), 3.13 (d, J = 7.3 Hz, 2H), 2.88 (d, J = 8.5 Hz, 1H), 2.68 – 2.44 (m, 3H), 2.31 – 2.13 (m, 1H), 1.62 (s, 2H), 1.45 (s, 2H), 1.28 (d, J = 19.4 Hz, 5H). ^{13}C NMR (126 MHz, $CDCl_3$) δ 156.53, 138.45, 138.41, 138.01, 137.92, 137.84, 137.78, 137.72, 137.56, 137.48, 137.33, 137.31, 136.71, 135.68, 135.61, 135.57, 135.55, 133.28, 133.07, 128.59, 128.57, 128.52, 128.37, 128.16, 127.96, 127.89, 127.79, 126.63, 126.32, 126.13, 125.83, 125.81, 116.85, 116.82, 116.74, 116.71, 98.07, 97.93, 97.90, 97.78, 77.26, 76.21, 75.83, 75.79, 73.41, 73.37, 73.32, 73.29, 72.69, 72.66, 72.35, 72.32, 72.22, 70.04, 69.88, 69.55, 68.56, 68.49, 68.38, 68.29, 66.93, 66.62, 62.67, 62.62, 62.59, 62.52, 62.44, 62.40, 62.08, 62.04, 61.98, 61.94, 61.89, 61.86, 59.95, 59.87, 40.90, 30.06, 30.00, 29.78, 26.13, 25.01, 19.65, 19.59, 19.56, 19.50. ^{31}P NMR (202 MHz, $CDCl_3$) δ -1.34, -1.40, -1.62, -2.21. ESI HRMS (m/z): [M + Na]$^+$ calcd for $C_{77}H_{93}N_3O_{23}P_2Na$, 1512.5573; found 1512.5574 (Supplementary Fig. 38).

Synthesis of compound **25**

Compound **25** was synthetized by following the same procedure as for compound **20**. Substrate **24** (40 mg, 0.028 mmol), conc. ammonia (3 mL), dioxane (3 mL), *t*BuOH and water (3 mL, 1/1, v/v), 1 drop of acetic acid and $Pd(OH)_2/C$ (50 mg) were used and the hydrogenation took 24 h. The compound was purified using size-exclusion column chromatography (LH-20) giving 9 mg product as a white solid. ^{1}H NMR (500 MHz, D_2O) δ 4.99 (dd, *J* = 3.8, 3.8 Hz, 2H), 4.55 (dd, *J* = 3.0, 3.0 Hz, 1H), 4.15 – 3.88 (m, 14H), 3.88 – 3.71 (m, 7H), 3.63 (ddd, *J* = 10.4, 6.8, 3.7 Hz, 2H), 3.08 – 2.97 (m, 2H), 1.69 (dt, *J* = 13.0, 6.9 Hz, 4H), 1.49 – 1.40 (m, 4H). ^{13}C NMR (126 MHz, D_2O) δ 98.50, 98.47, 74.78, 74.74, 71.11, 70.85, 69.51, 69.32, 69.23, 69.16, 69.12, 68.56, 68.28, 68.09, 66.21, 66.16, 66.09, 66.04, 61.26, 60.95, 39.46, 29.54, 29.48, 26.62, 25.17, 24.49. ^{31}P NMR (202 MHz, D_2O) δ 0.81, 0.78. ESI HRMS (m/z): $[M + H]^+$ calcd for $C_{24}H_{50}NO_{21}P_2$, 750.2351; found 750.2350 (Supplementary Fig. 39).

Coupling of App7 with BODIPY

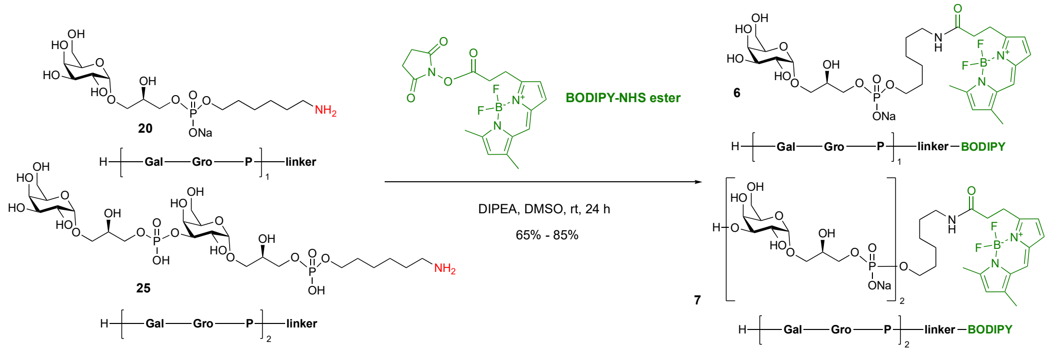

Supplementary Fig. 25 | Synthesis of App7-BODIPY.

General procedure

To a solution of App7 substrates (1 mg – 6.9 mg) in 0.5 mL DMSO was added BODIPY-NHS ester (1.5 eq) and DIPEA (2.0 eq). The resulting mixture was stirred at rt for 24 h until completion. The mixture was lyophilized, and the residue was purified by LH-20 gel filtration and prep-HPLC (C18).

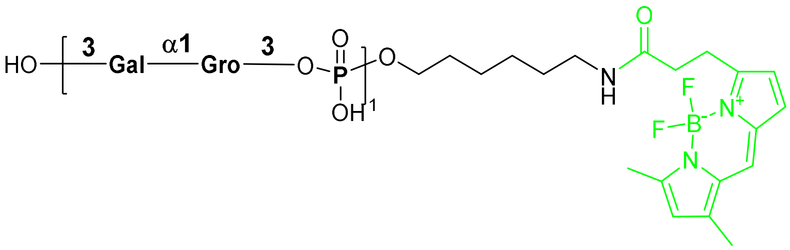

Supplementary Fig. 26 | Chemical structure of compound 6.

^{1}H NMR (850 MHz, D_2O) δ 7.45 (s, 1H), 7.04 (s, 1H), 6.34 (s, 1H), 6.27 (s, 1H), 4.89 (d, *J* = 3.9 Hz, 1H), 4.02 – 3.98 (m, 1H), 3.92 – 3.81 (m, 6H), 3.79 – 3.72 (m, 4H), 3.69 – 3.65 (m, 2H), 3.52 (dd, *J* = 10.6, 3.6 Hz, 1H), 3.15 (s, 2H), 3.07 (t, *J* = 6.5 Hz, 2H), 2.62 (t, *J* = 7.1 Hz, 2H), 2.50 – 2.44 (m, 3H), 2.25 – 2.20 (m, 3H), 1.47 – 1.41 (m, 2H), 1.33 – 1.29 (m, 2H), 1.21 – 1.14 (m, 2H), 1.04 (s, 2H). ^{13}C NMR (214 MHz, D_2O) δ 175.58, 162.49, 156.47, 147.50, 136.29, 134.08, 129.71, 125.86, 121.98, 117.81, 99.27, 71.89, 70.40, 70.13, 70.03, 69.99, 69.43, 68.86, 67.18, 67.15, 66.75, 66.73, 62.04, 40.09, 35.81, 30.71, 30.68, 29.12, 26.50, 25.69, 25.39, 15.15, 11.47. ESI HRMS (m/z): $[M + H]^+$ calcd for $C_{29}H_{46}BF_2N_3O_{12}P$, 708.2880; found 708.2882 (Supplementary Fig. 40).

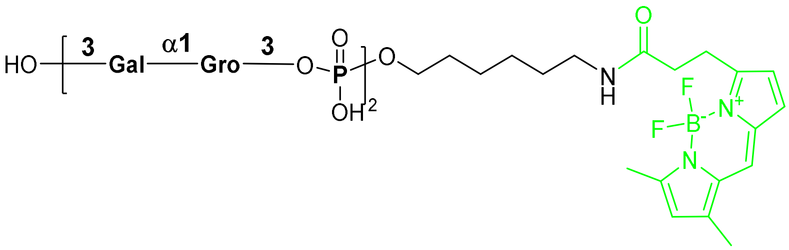

Supplementary Fig. 27 | Chemical structure of compound 7.

^{1}H NMR (850 MHz, D_2O) δ 7.50 (d, *J* = 5.7 Hz, 1H), 7.08 (d, *J* = 3.7 Hz, 1H), 6.38 – 6.32 (m, 1H), 6.30 (s, 1H), 4.91 (dd, *J* = 3.9, 3.9 Hz, 2H), 4.48 (dd, *J* = 9.1, 3.1 Hz, 1H), 4.07 – 3.63 (m, 21H), 3.58 – 3.52 (m,

2H), 3.16 (d, J = 7.0 Hz, 2H), 3.10 – 3.03 (m, 2H), 2.63 (s, J = 7.0 Hz, 3H), 2.49 (s, 3H), 2.26 (s, 3H), 1.44 (t, J = 7.5 Hz, 2H), 1.34 – 1.29 (m, 2H), 1.21 – 1.15 (m, 2H), 1.03 (t, J = 7.9 Hz, 2H). ^{13}C NMR (214 MHz, D_2O) δ 175.59, 162.56, 156.50, 147.58, 136.32, 134.08, 129.75, 125.94, 122.02, 117.88, 99.35, 99.26, 75.62, 75.59, 71.95, 71.93, 71.66, 70.35, 70.33, 70.16, 70.06, 70.03, 69.94, 69.43, 69.18, 68.94, 68.89, 67.45, 67.32, 67.19, 67.17, 66.89, 66.86, 62.11, 61.98, 61.77, 40.09, 35.87, 30.71, 30.68, 29.12, 26.50, 25.70, 25.42, 15.17, 11.49. ESI HRMS (m/z): $[M + H]^+$ calcd for $C_{38}H_{63}BF_2N_3O_{22}P_2$, 1024.3440; found 1024.3441 (Supplementary Fig. 41).

Supplementary Fig. 28 | NMR Spectra of compound 13.

a) ^{1}H NMR (500 MHz, $CDCl_3$)

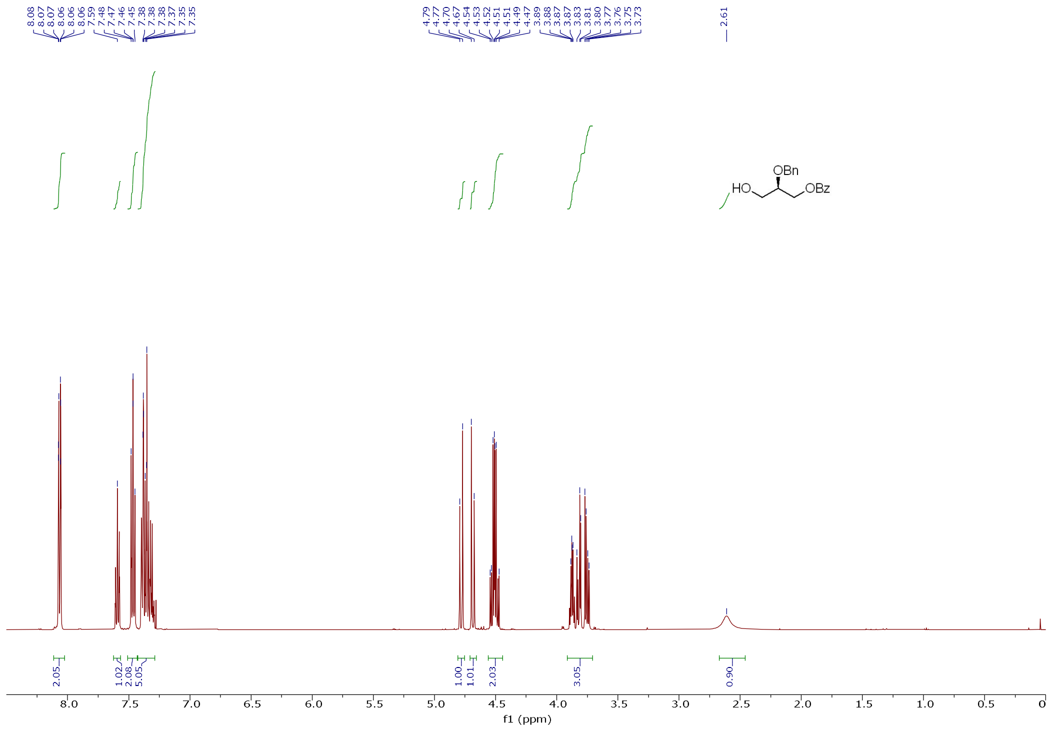

b) H-H COSY NMR

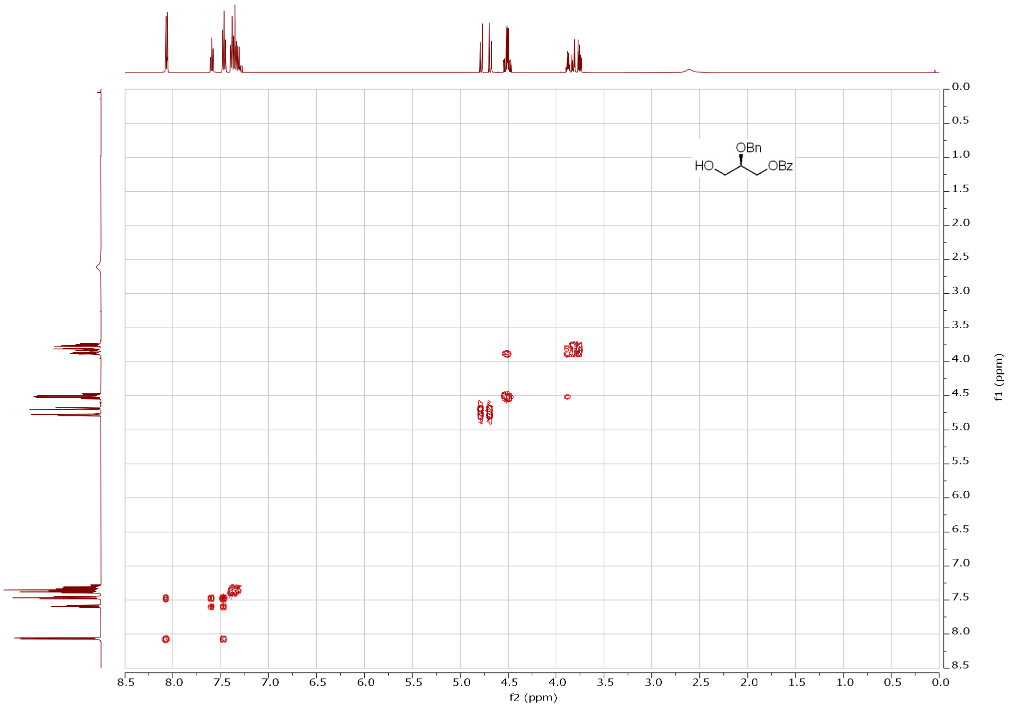

c) HSQC

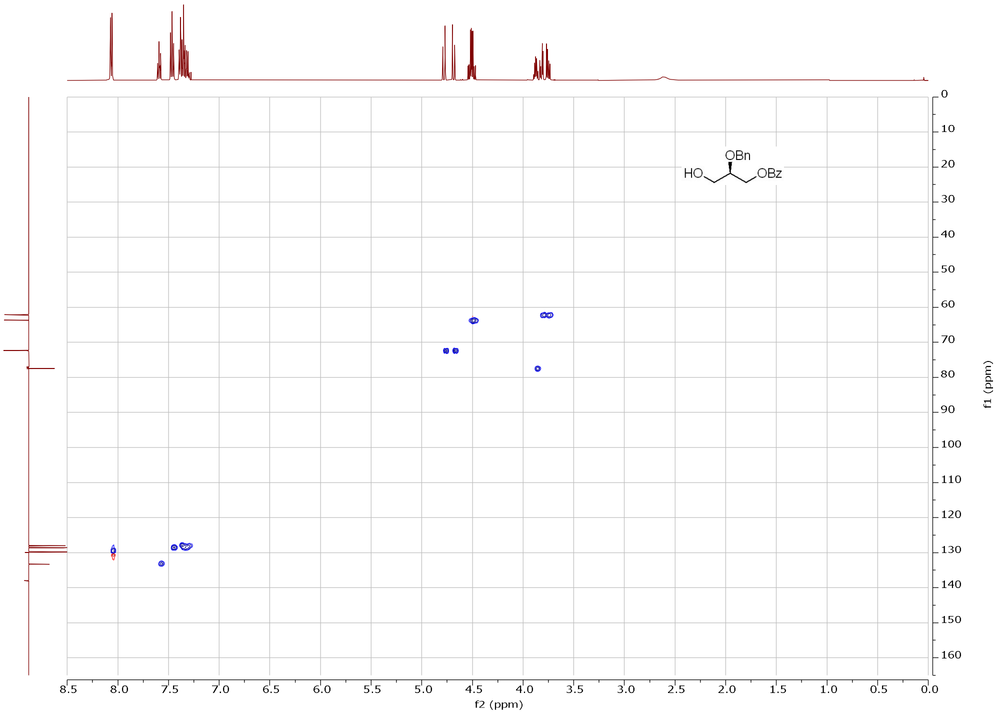

d) ^{13}C NMR (126 MHz, $CDCl_3$)

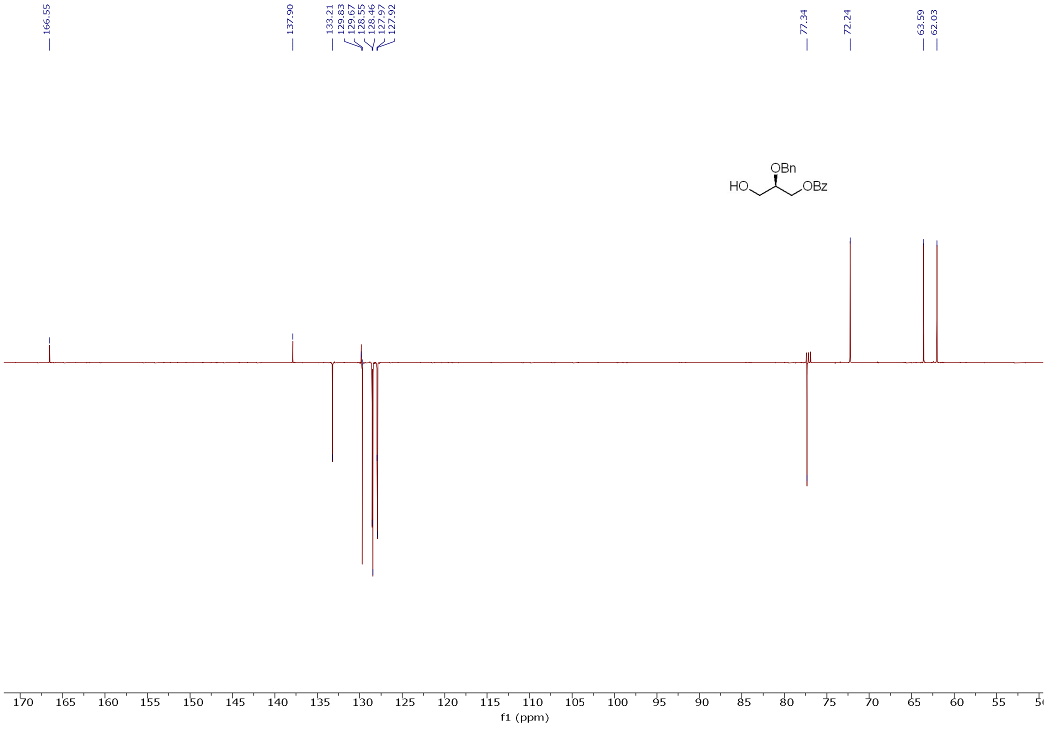

Supplementary Fig. 29 | NMR Spectra of compound 14.

a) ^{1}H NMR (500 MHz, $CDCl_3$)

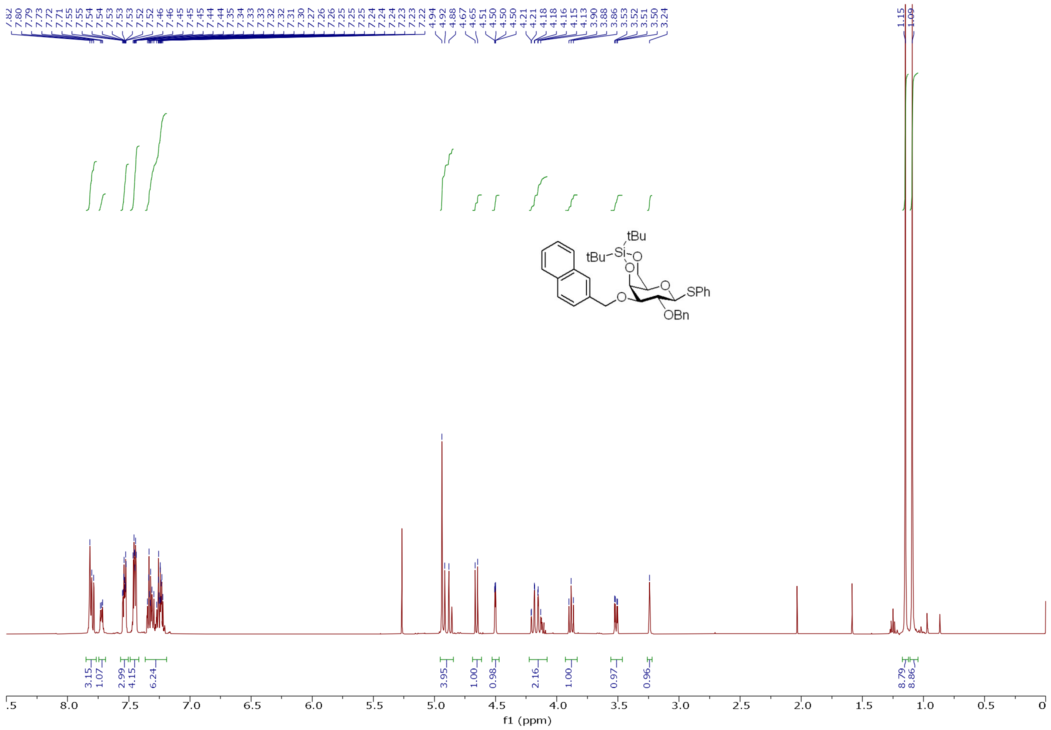

b) H-H COSY

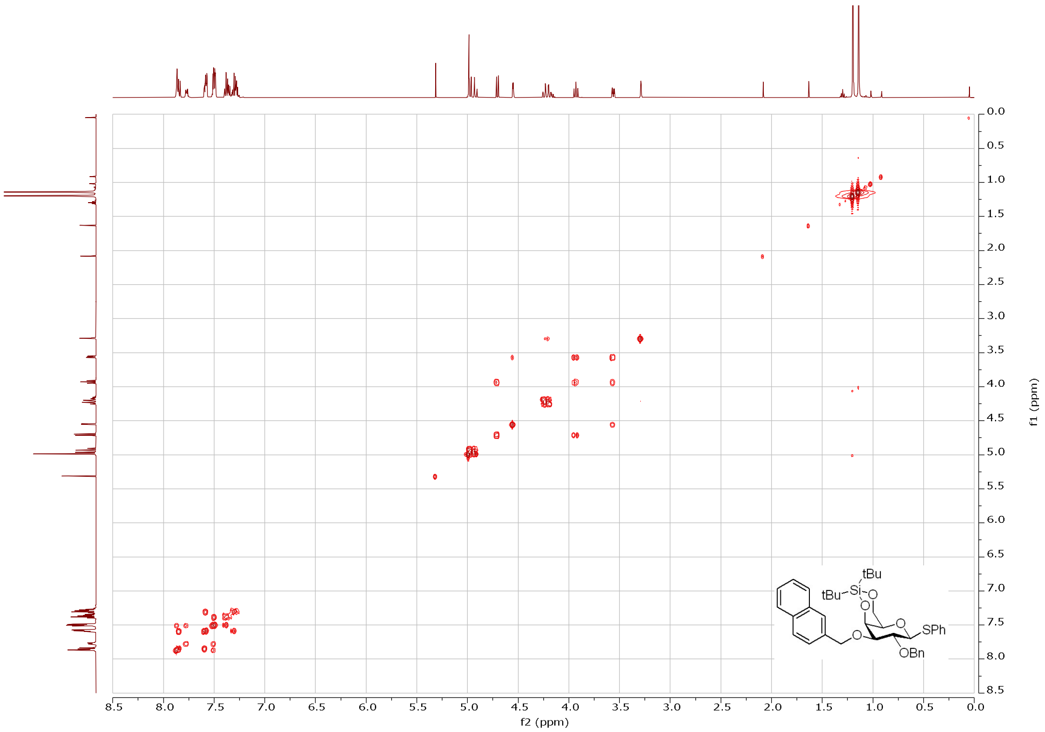

c) HSQC

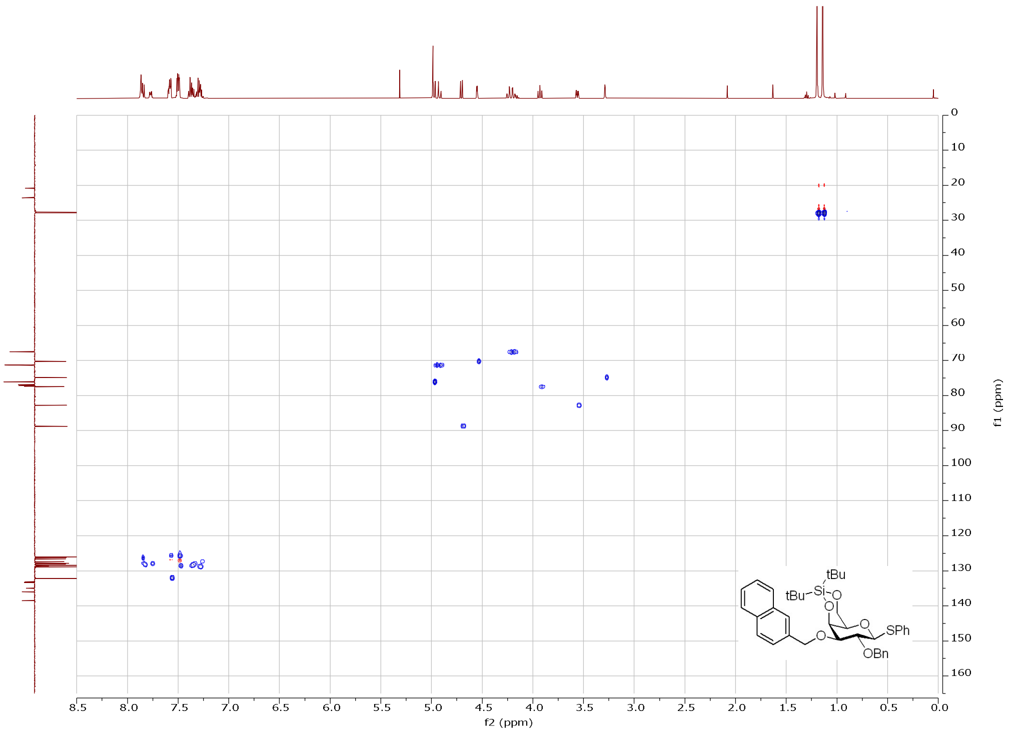

d) ^{13}C NMR (126 MHz, $CDCl_3$)

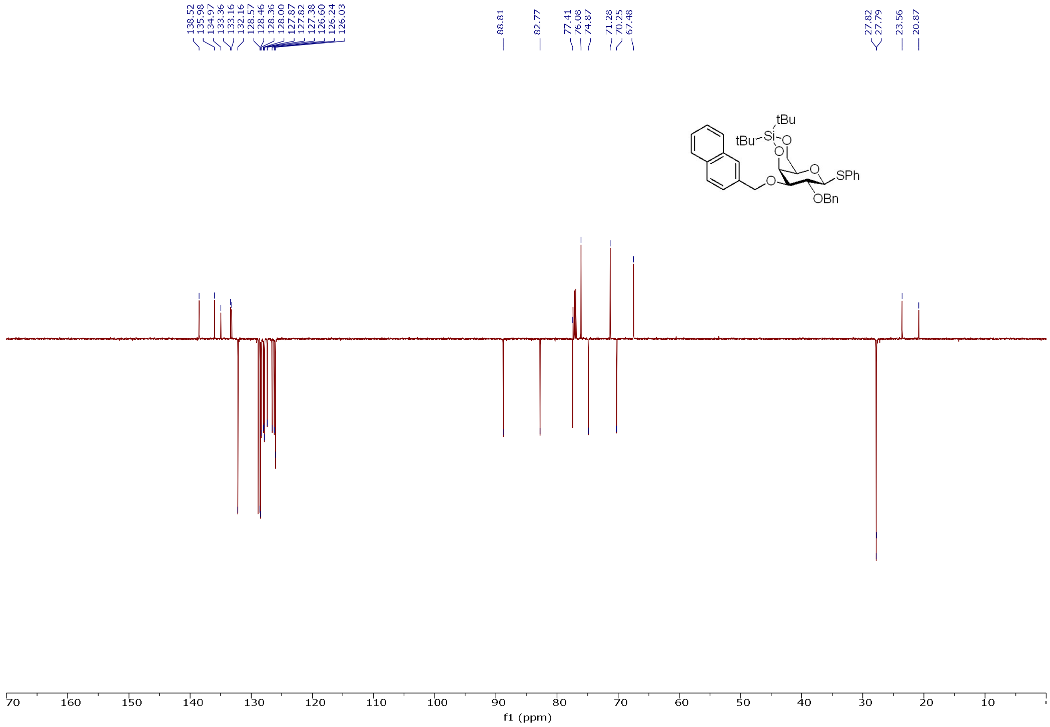

e) HMBC

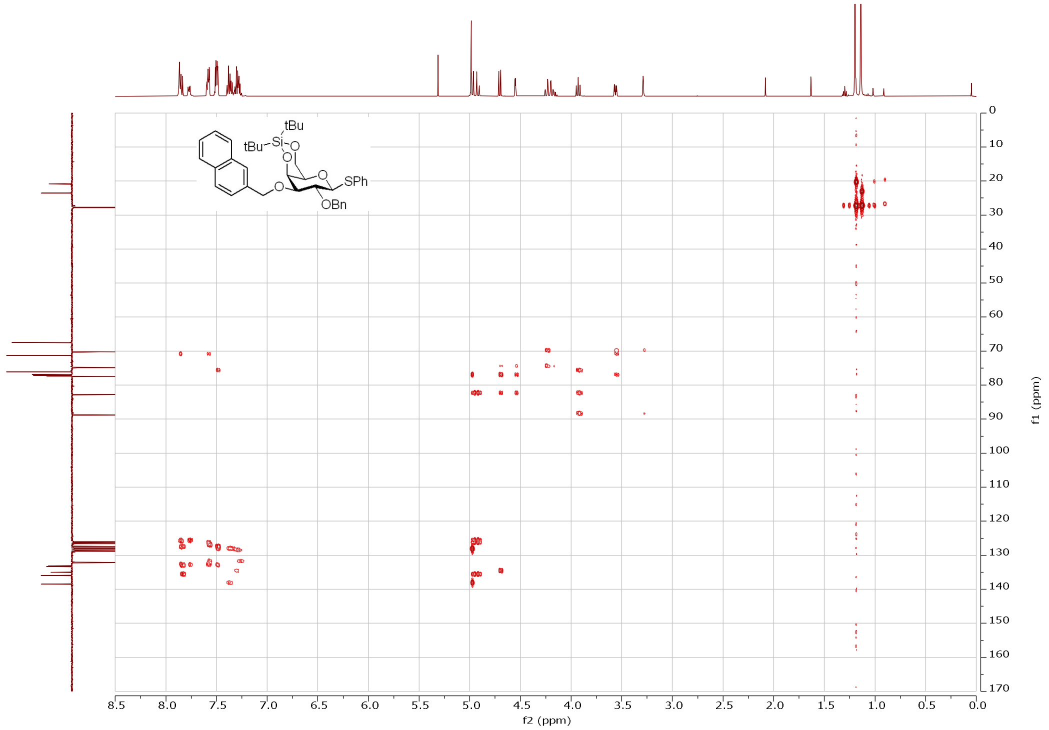
tBu
tBu
Si
O
O
O
O
SPh
OBn
f1 (ppm)
f2 (ppm)
0
10
20
30
40
50
60
70
80
90
100
110
120
130
140
150
160
170
8.5
8.0
7.5
7.0
6.5
6.0
5.5
5.0
4.5
4.0
3.5
3.0
2.5
2.0
1.5
1.0
0.5
0.0

Supplementary Fig. 30 | NMR Spectra of compound 15.

a) ^{1}H NMR (400 MHz, $CDCl_3$)

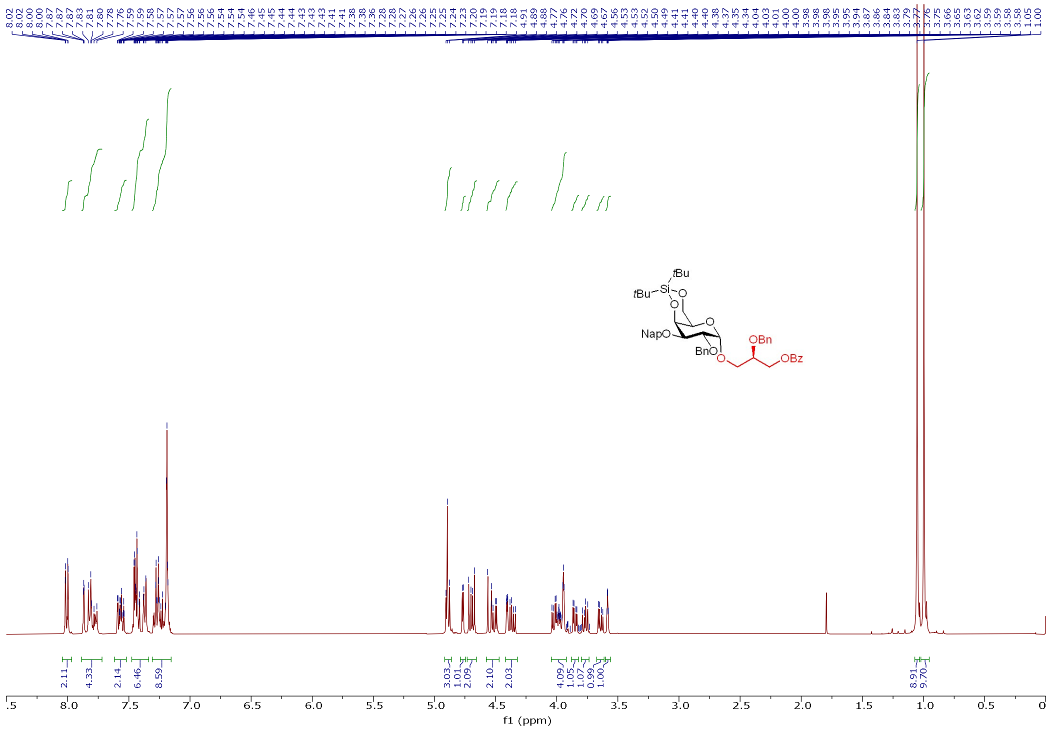

b) H-H COSY

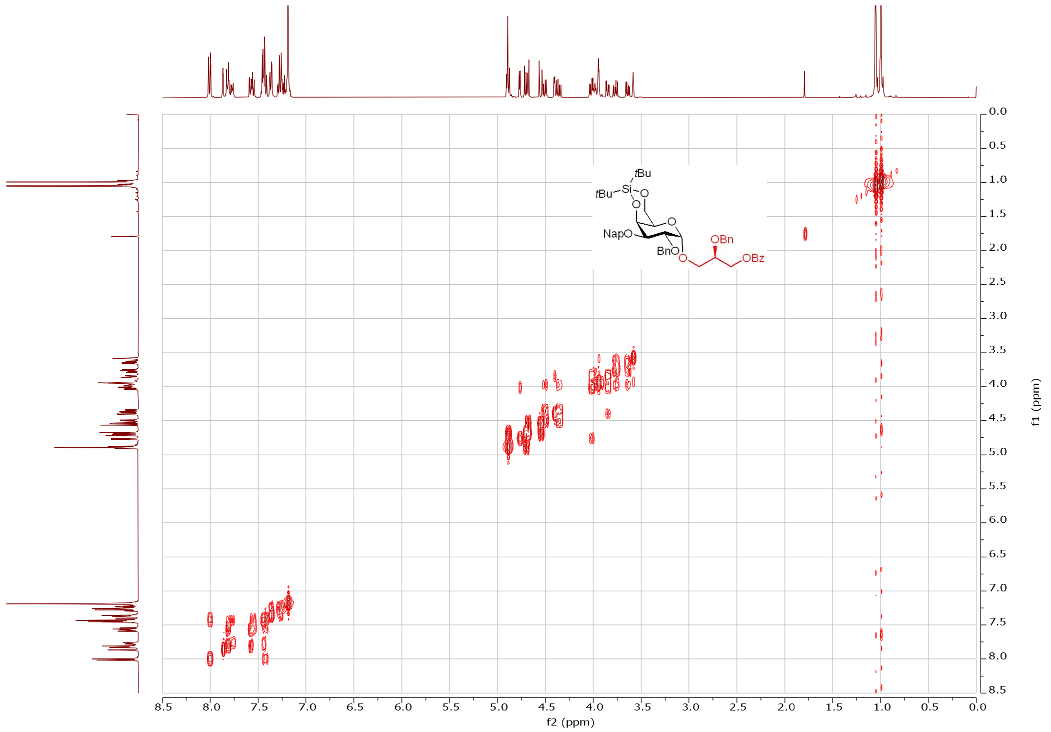

c) HSQC

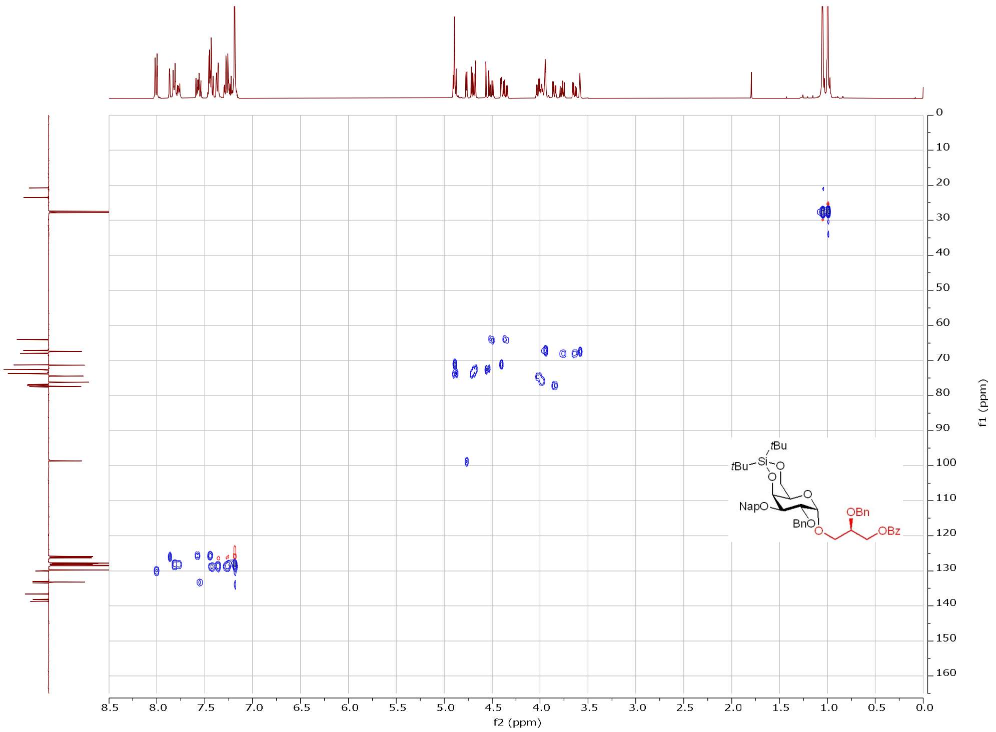

d) ^{13}C NMR (101 MHz, $CDCl_3$)

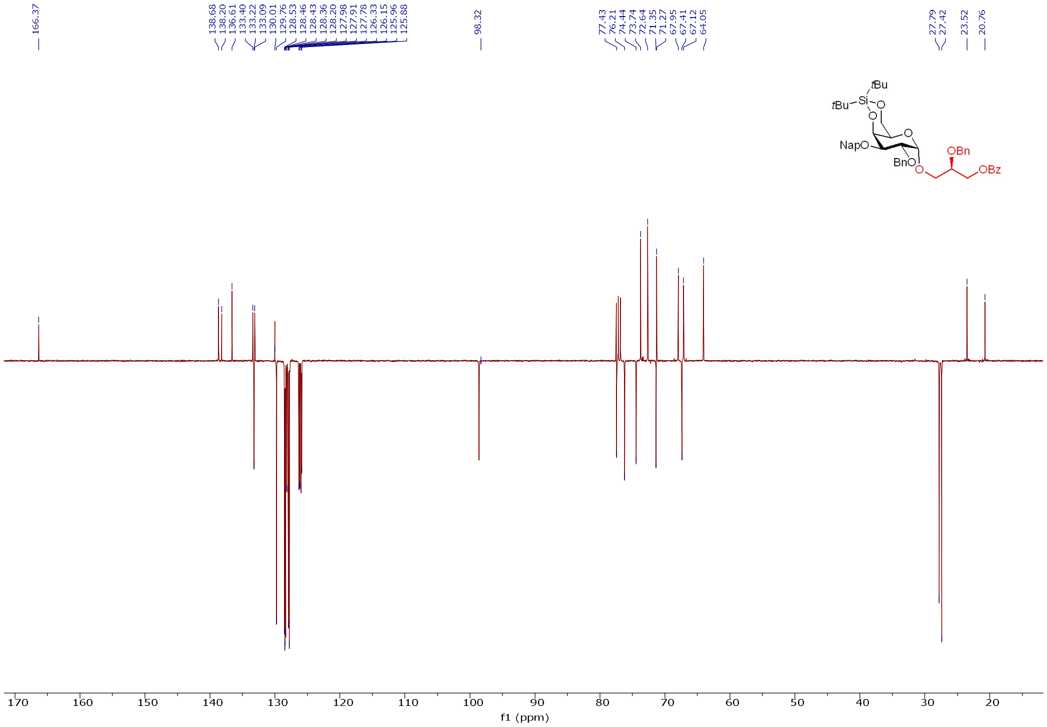

e) HMBC

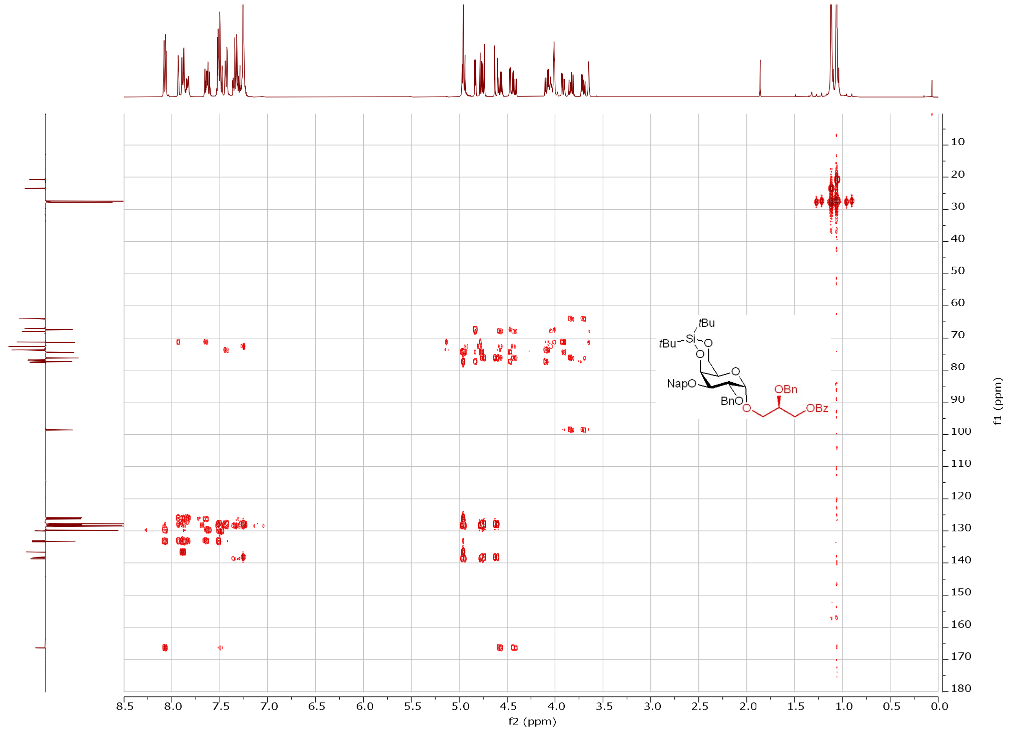

f) ^{13}C-HMBCipv GATED

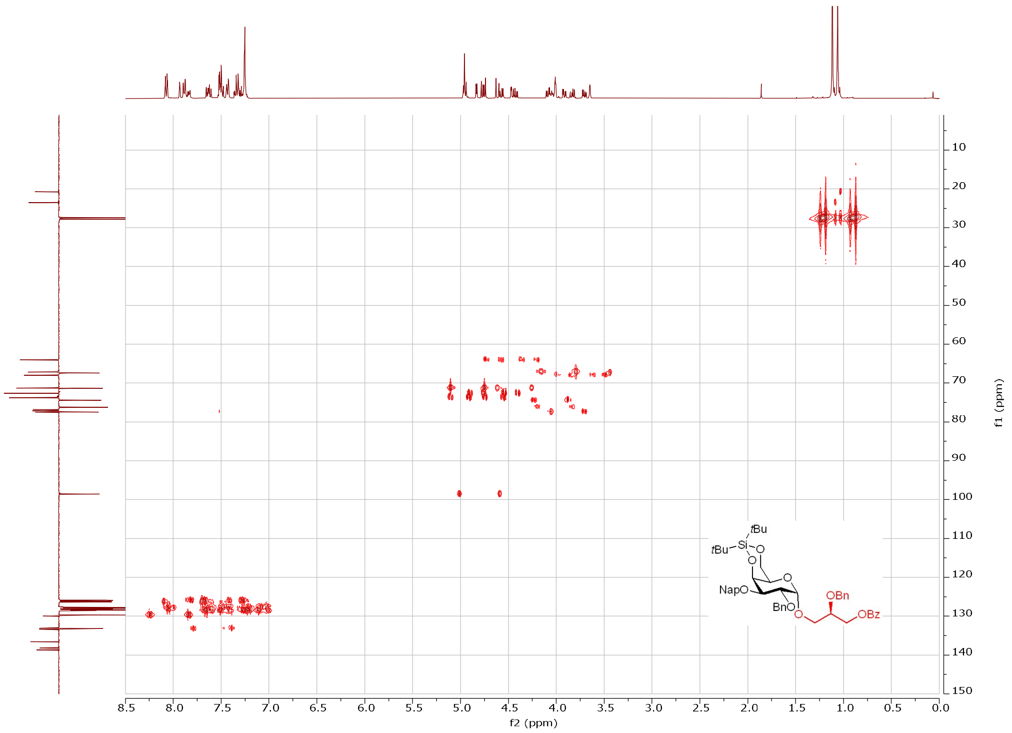

Supplementary Fig. 31 | NMR Spectra of compound 16.

a) ^{1}H NMR (500 MHz, $CDCl_3$)

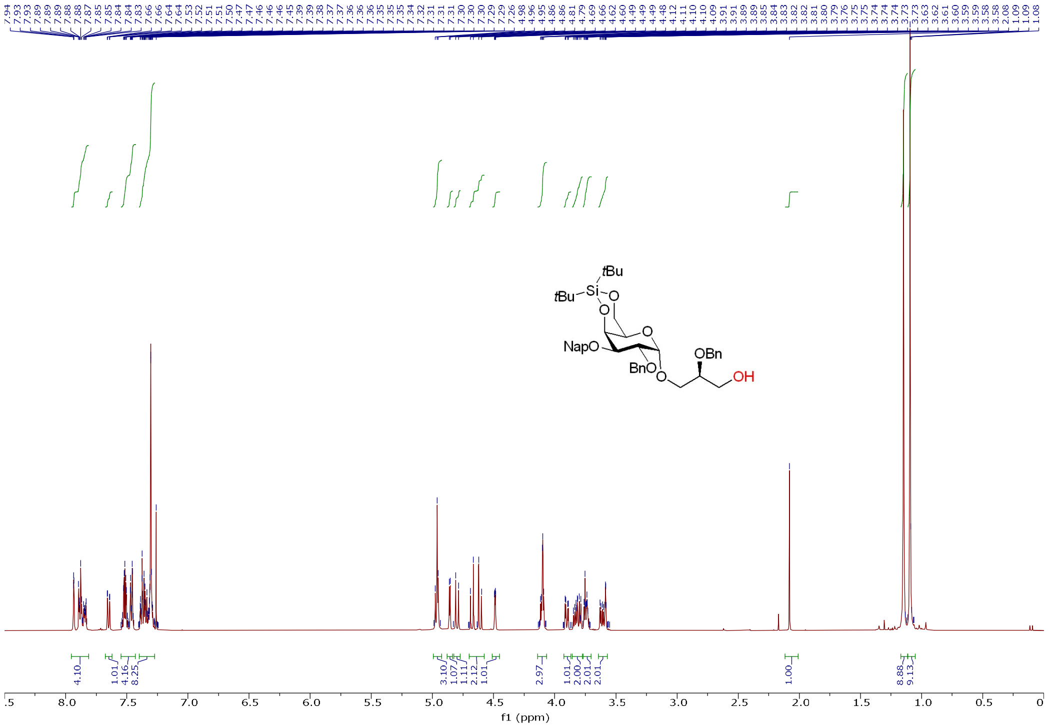

b) H-H COSY

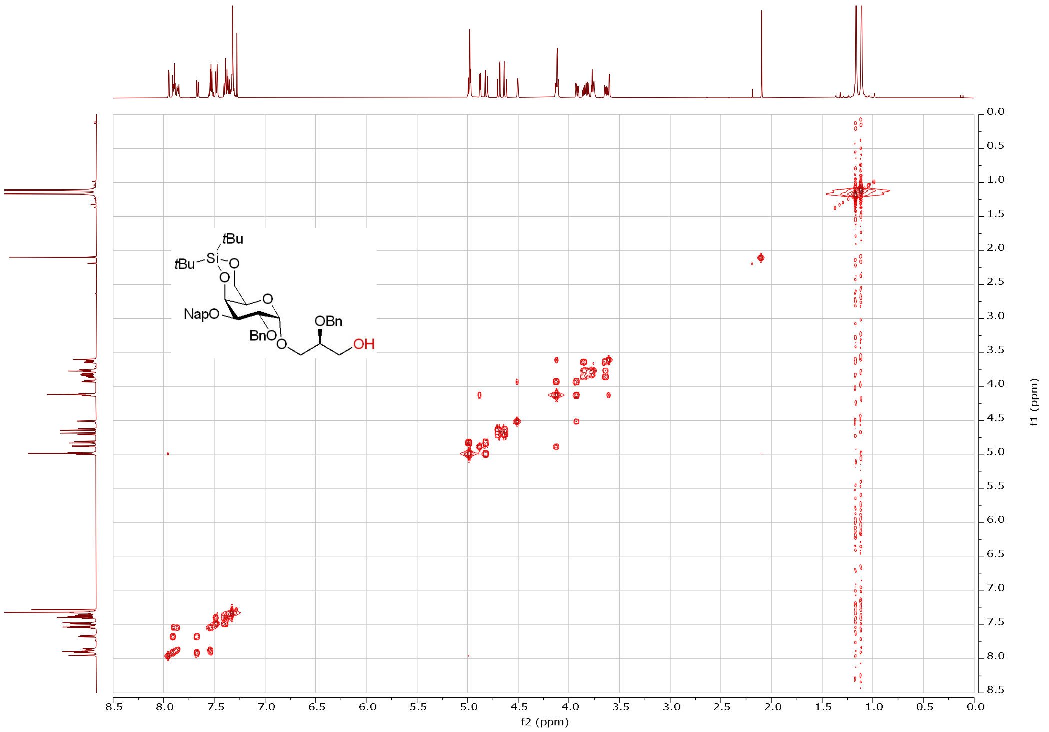

c) HSQC

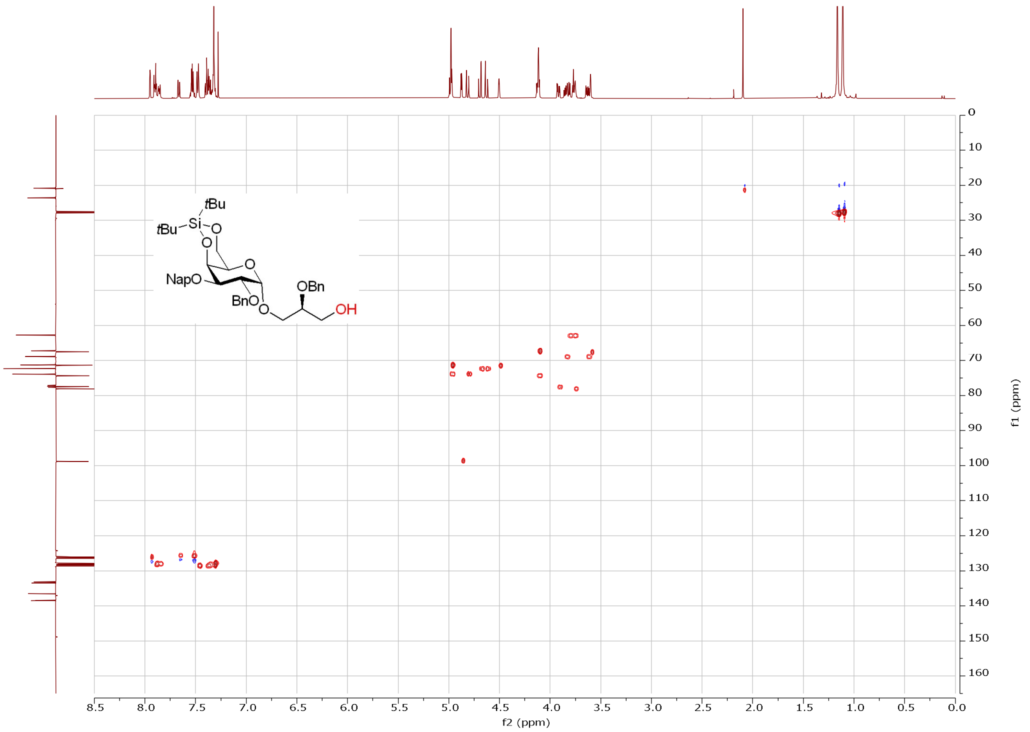

d) ^{13}C NMR (126 MHz, $CDCl_3$)

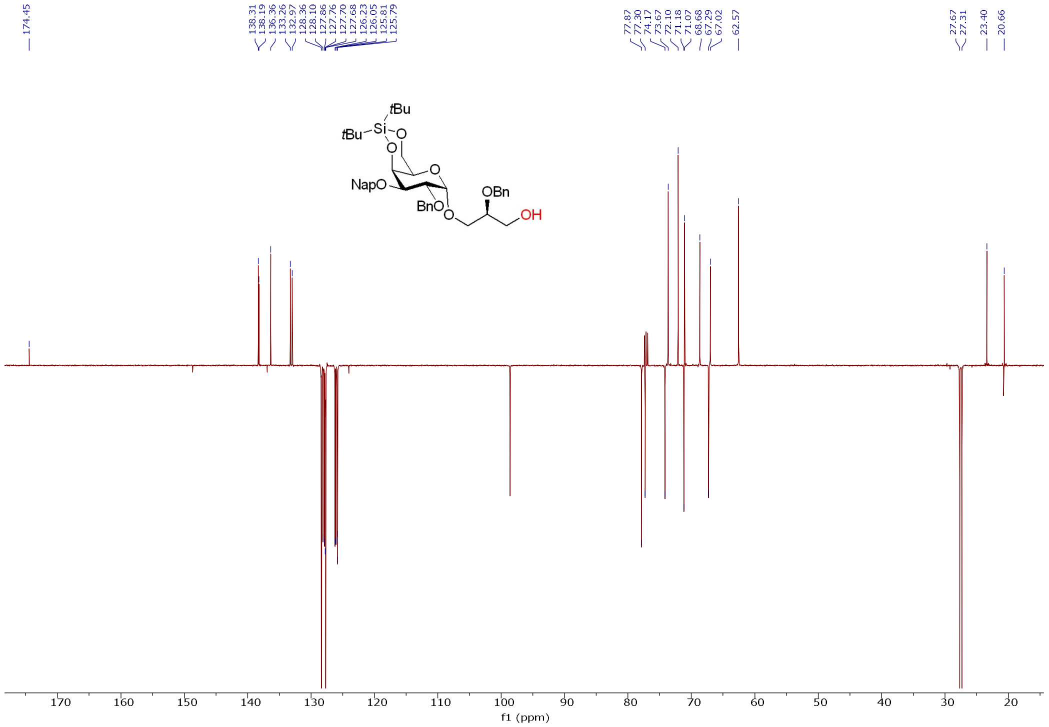

e) HMBC

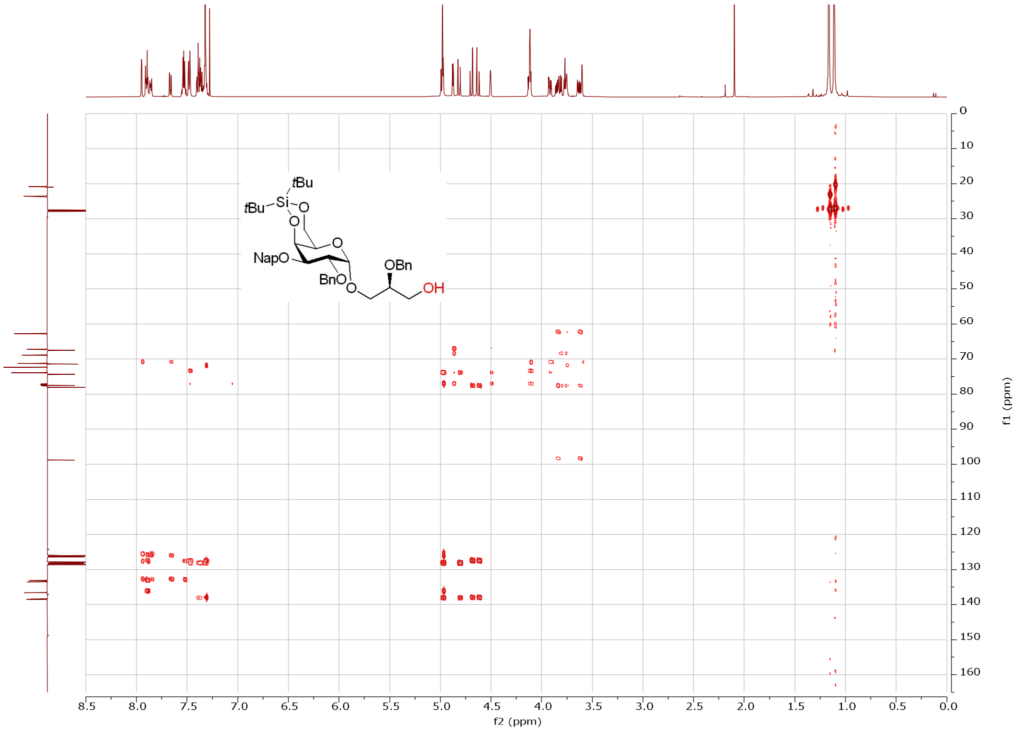
tBu
tBu
Si
O
O
O
NapO
BnO
O
OBn
OH
f1 (ppm)
f2 (ppm)
8.5
8.0
7.5
7.0
6.5
6.0
5.5
5.0
4.5
4.0
3.5
3.0
2.5
2.0
1.5
1.0
0.5
0.0
0
10
20
30
40
50
60
70
80
90
100
110
120
130
140
150
160

Supplementary Fig. 32 | NMR Spectra of compound 18.

a) ^{1}H NMR (500 MHz, CDCl$_3$)

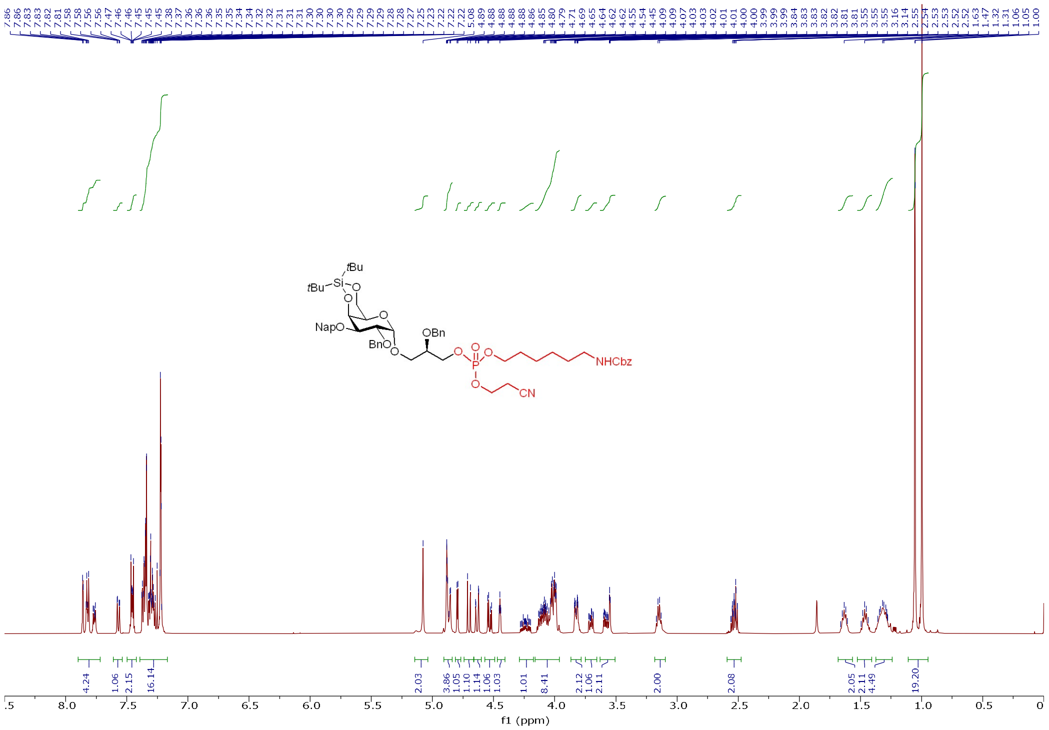

b) H-H COSY

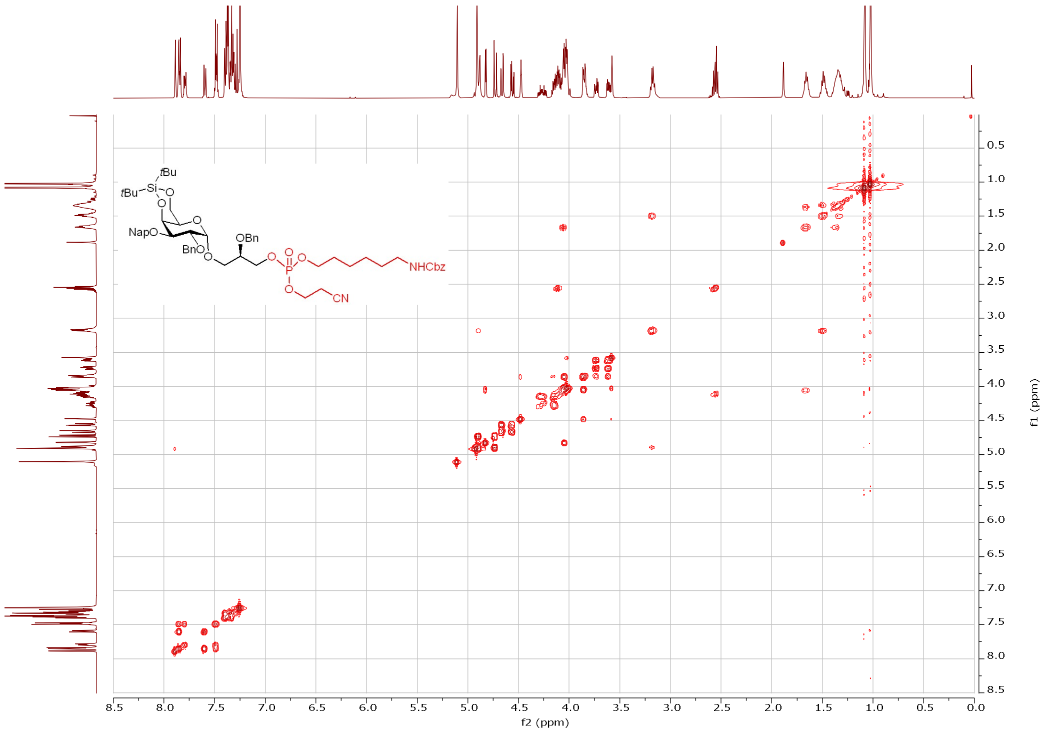

c) HSQC

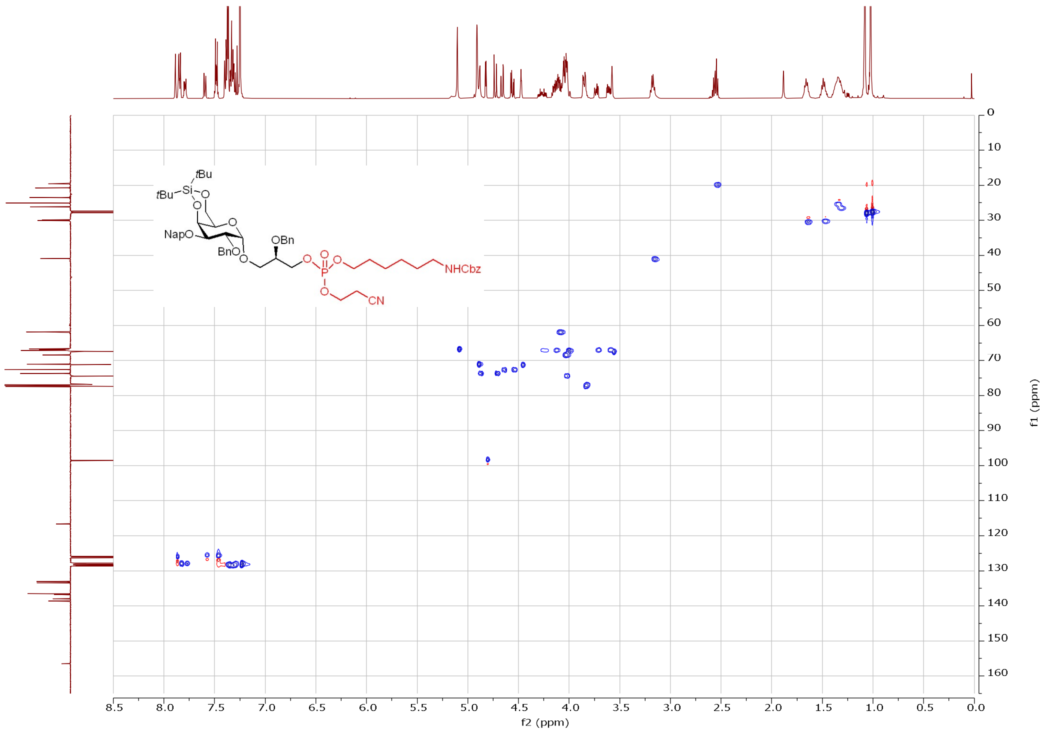

d) ^{13}C NMR (126 MHz, $CDCl_3$)

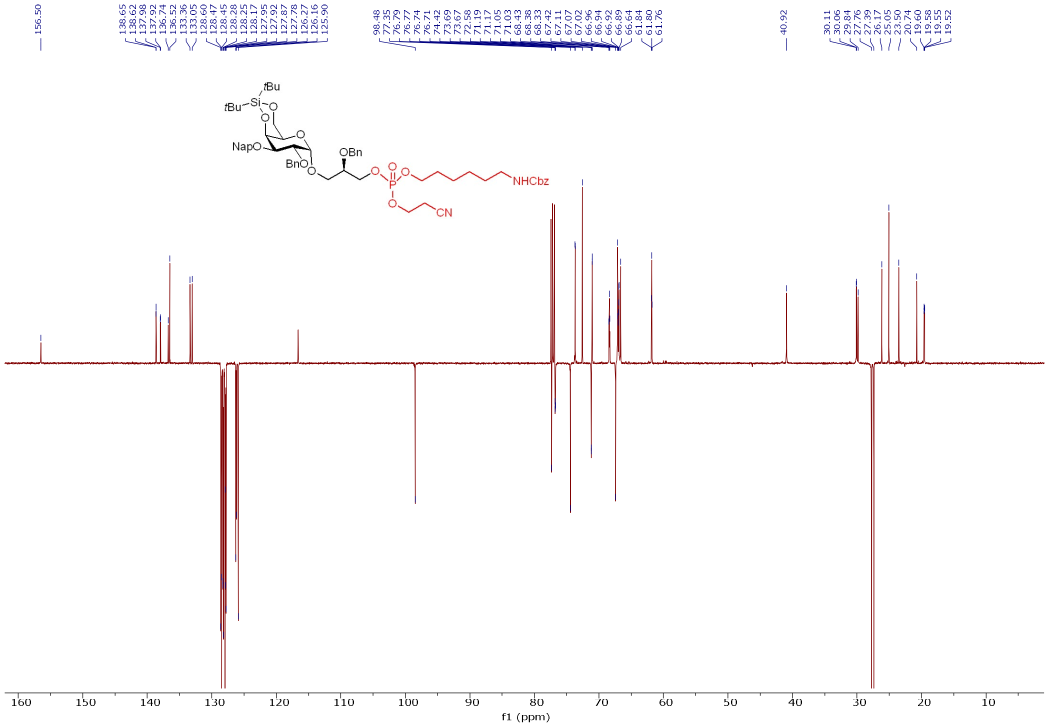

d) ^{31}P NMR (202 MHz, $CDCl_3$)

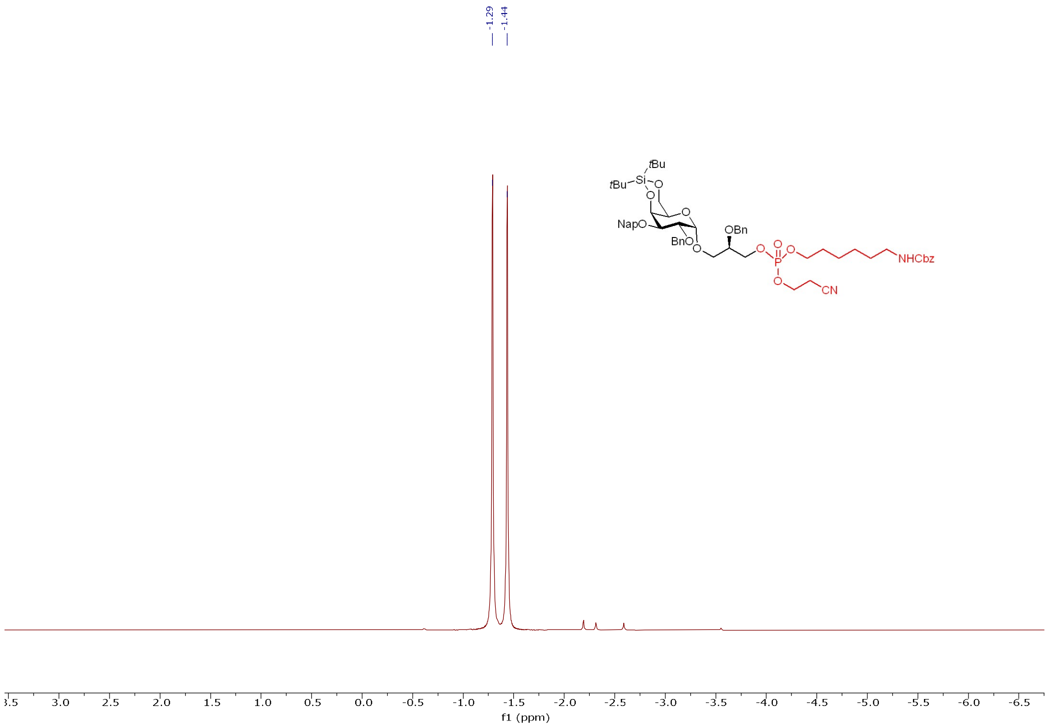

Supplementary Fig. 33 | NMR Spectra of compound 19.

a) ^{1}H NMR (400 MHz, $CDCl_3$)

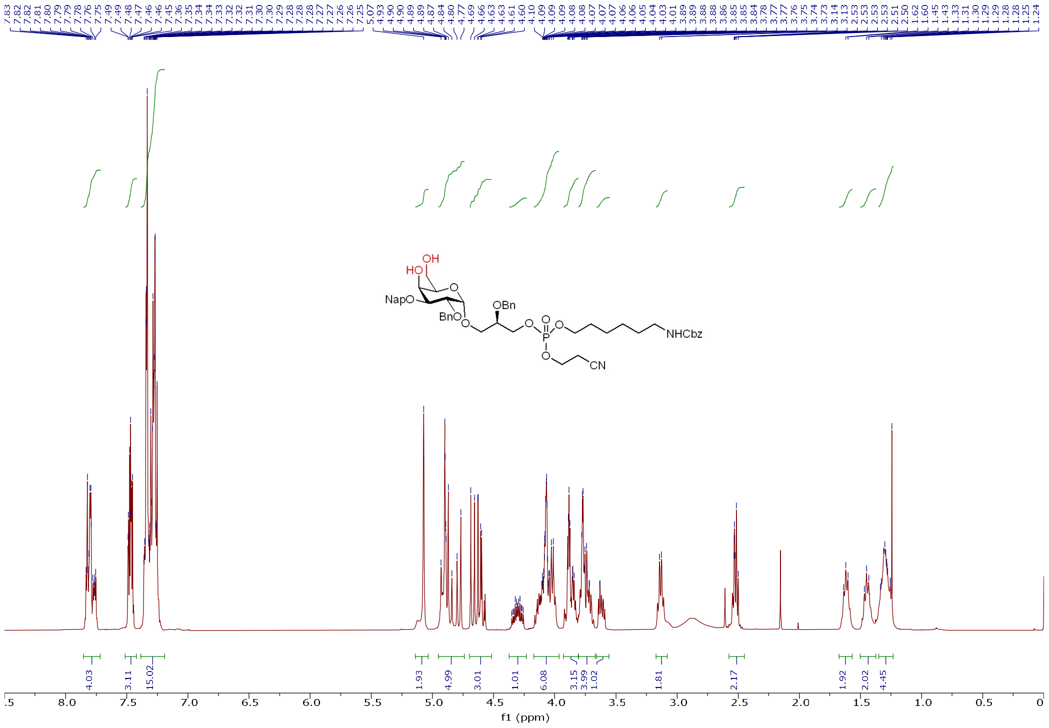

b) H-H COSY

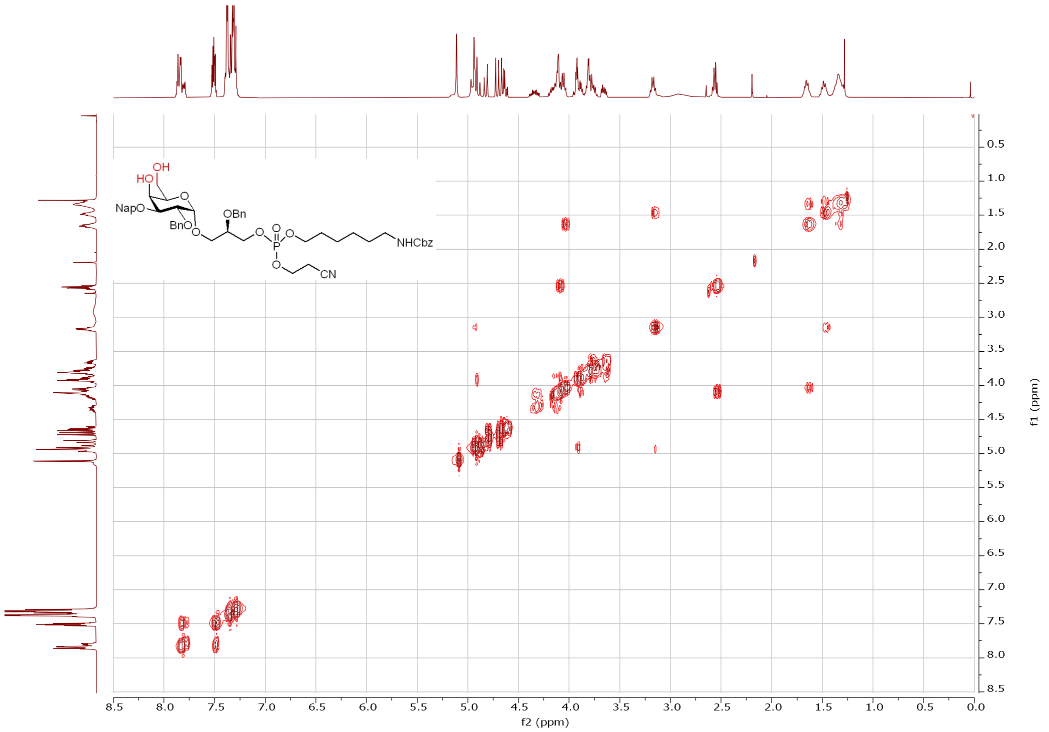

c) HSQC

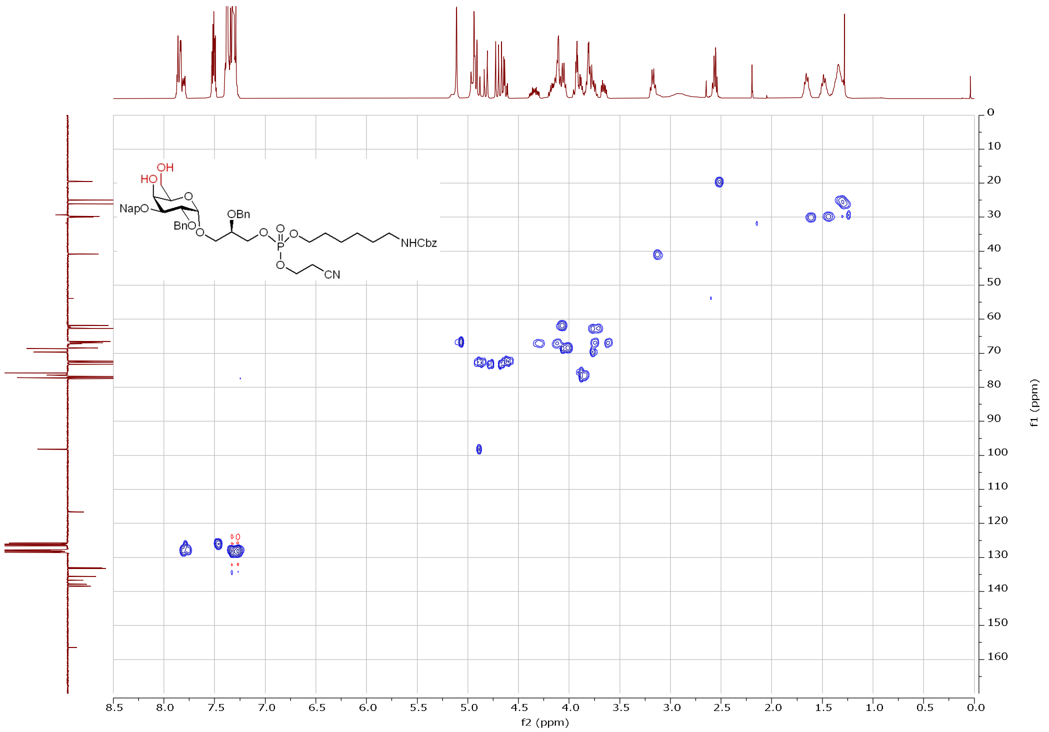

d) ^{13}C NMR (101 MHz, $CDCl_3$)

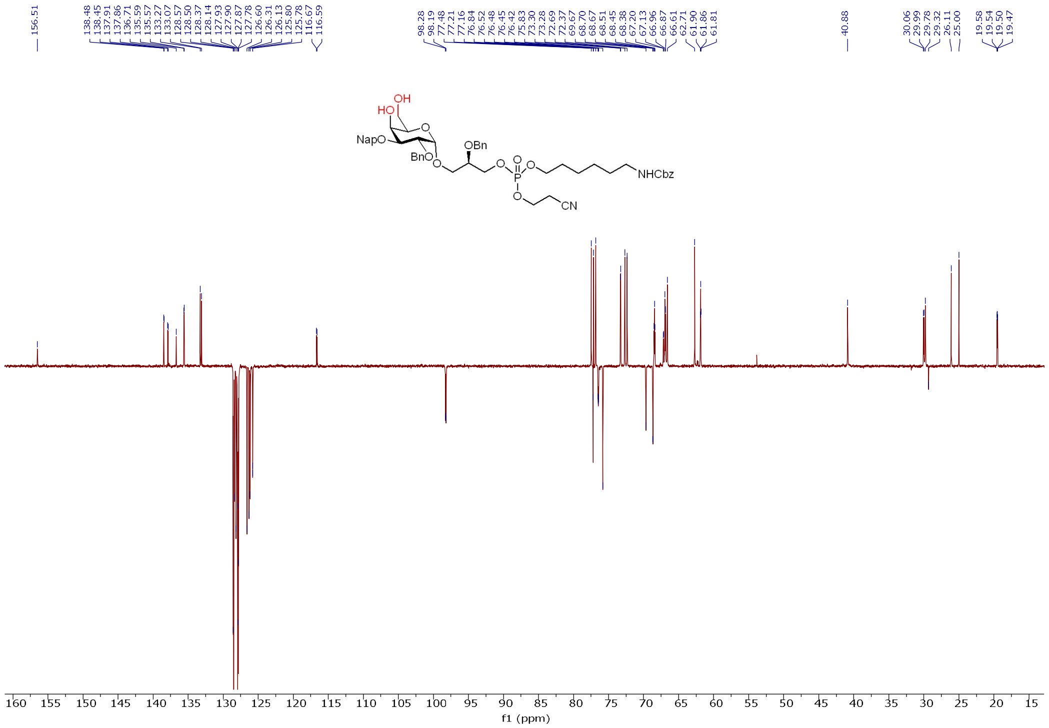

d) HMBC

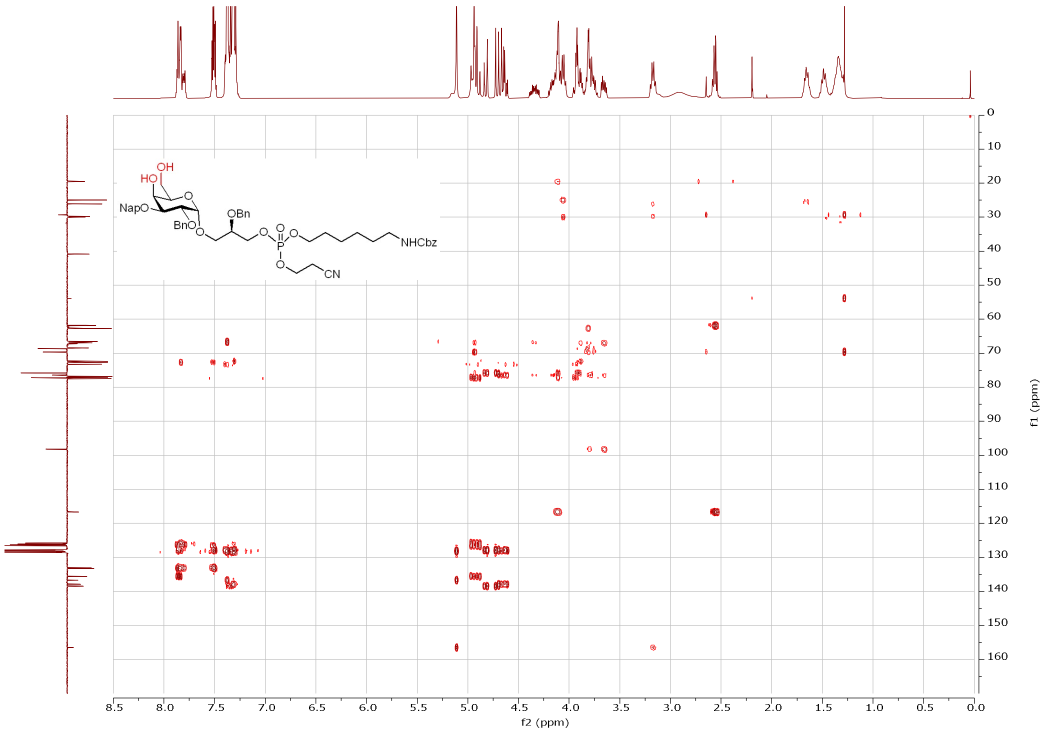

e) ^{31}P NMR (162 MHz, $CDCl_3$)

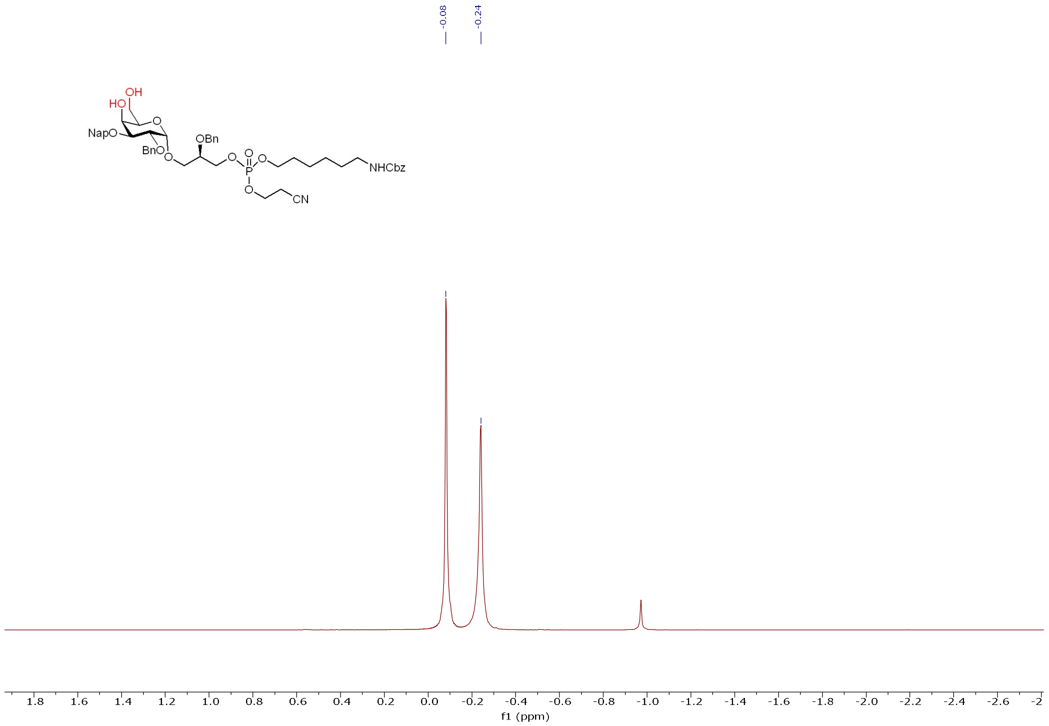

Supplementary Fig. 34 | NMR Spectra of compound 20.

a) ^1H NMR (500 MHz, D_2O)

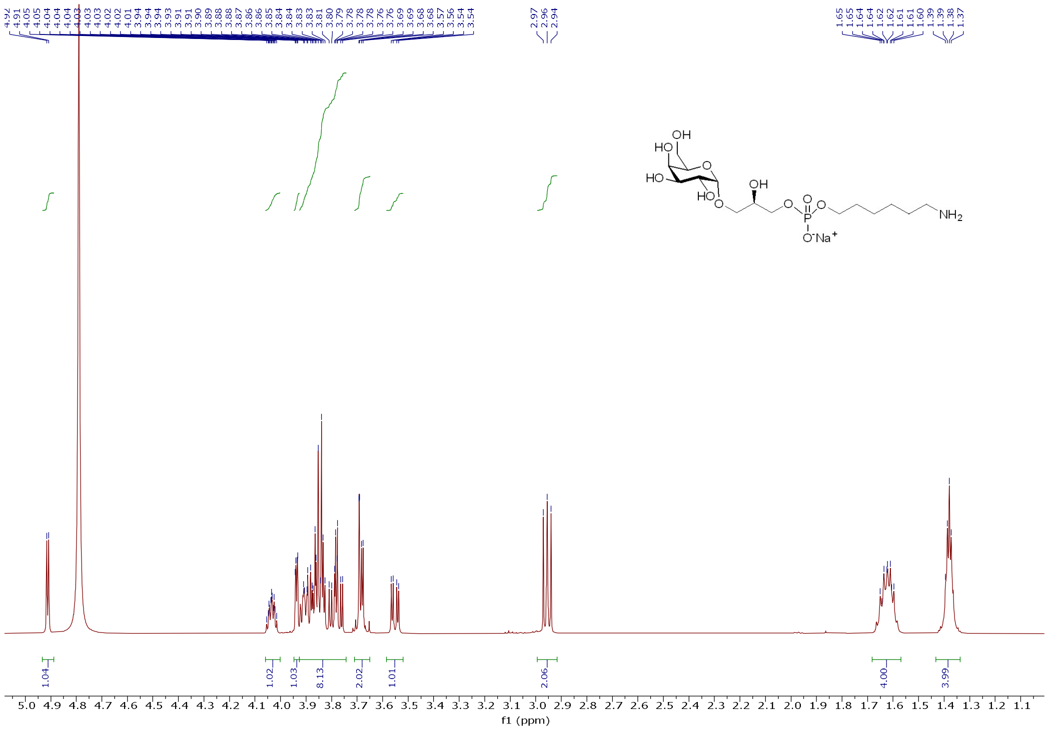

b) H-H COSY

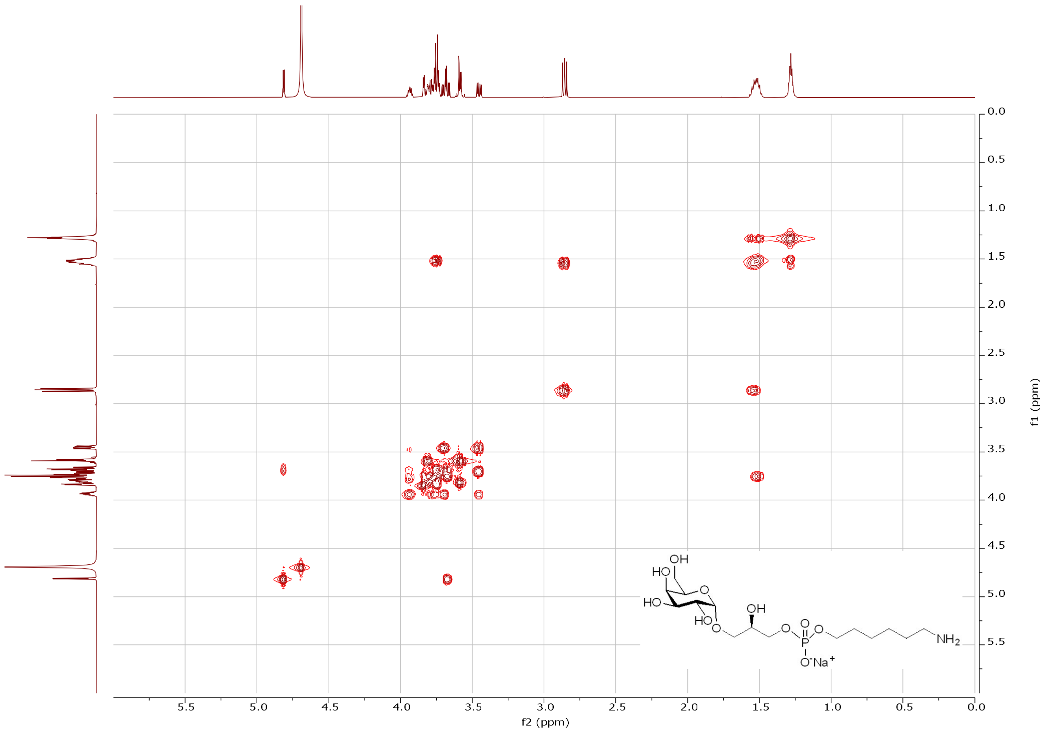

c) HSQC

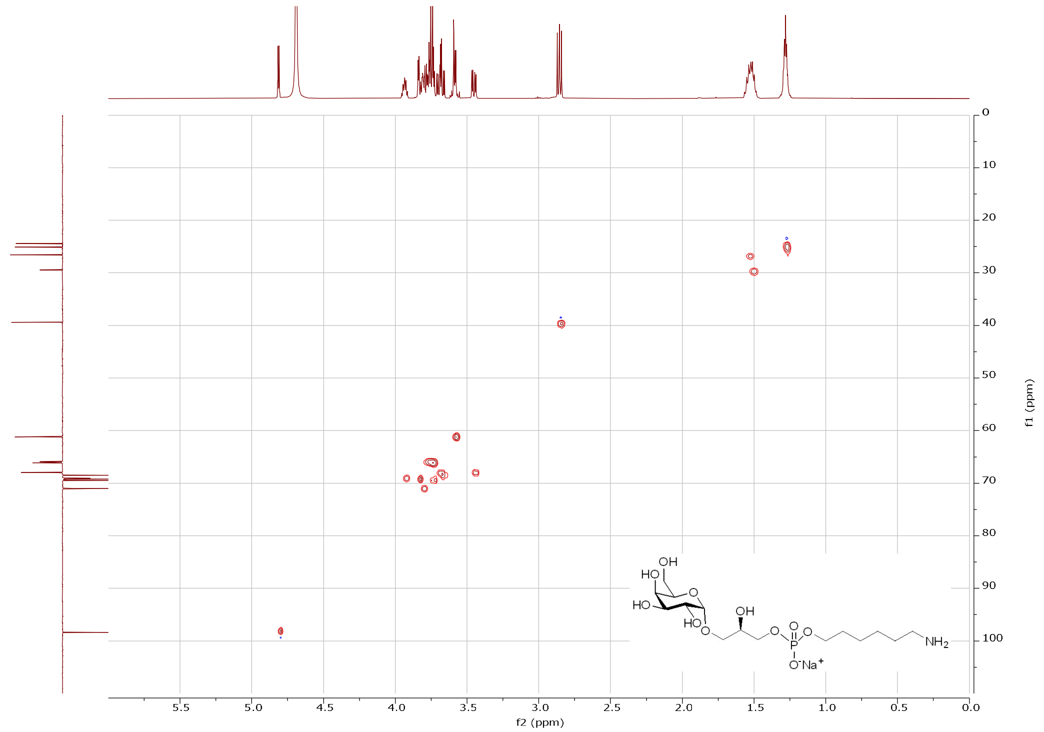

d) ^{13}C NMR (126 MHz, D_2O)

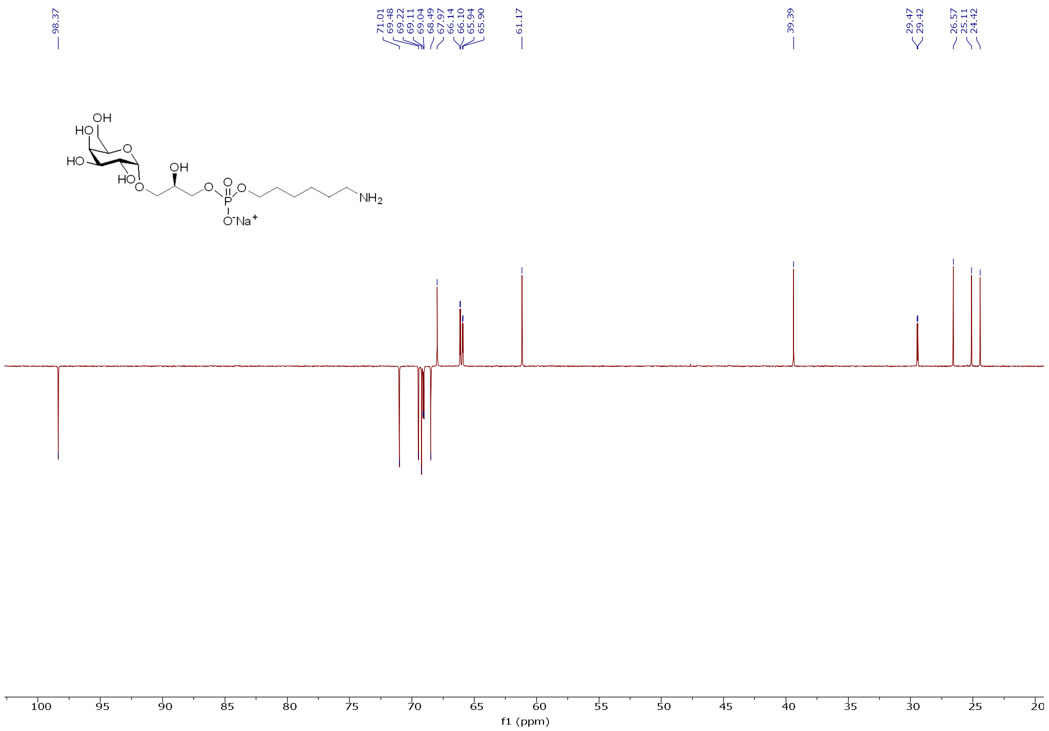

e) HMBC

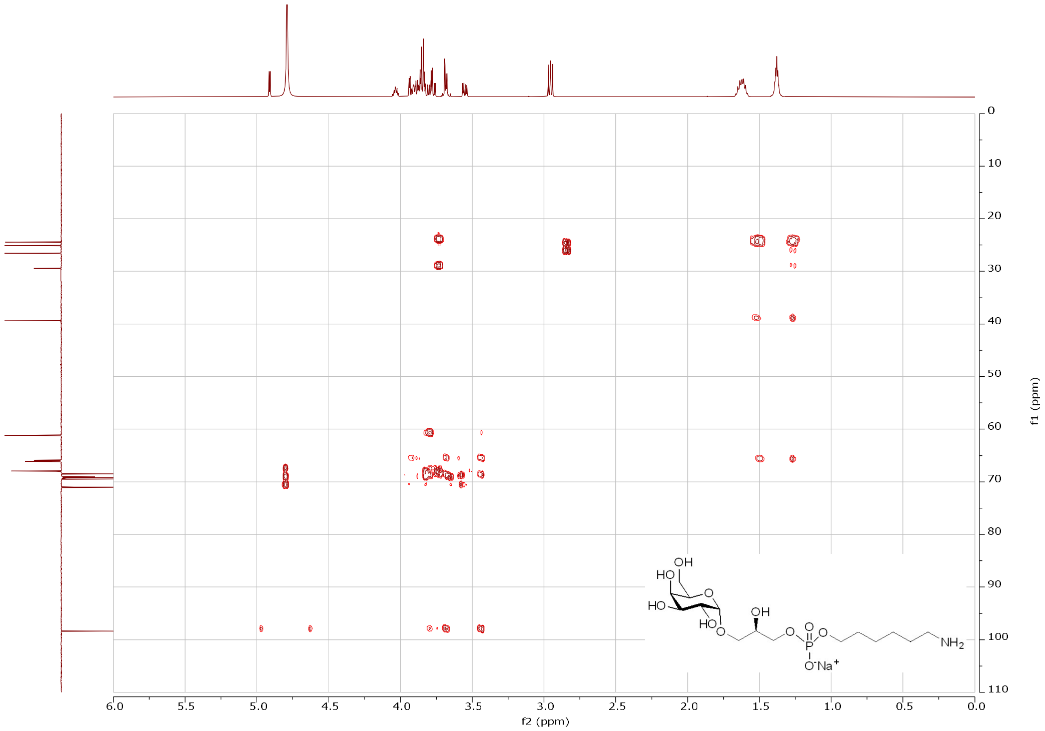

f) 13C-HMBC-ipvGATED

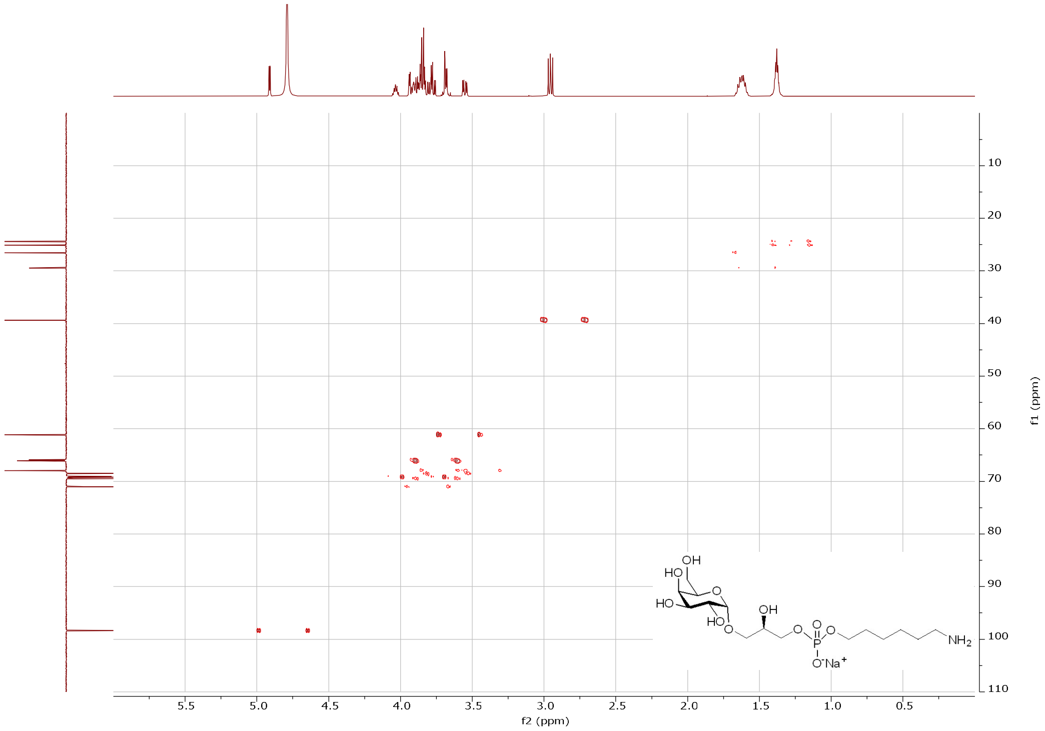

g) ^{31}P NMR (202 MHz, D_2O)

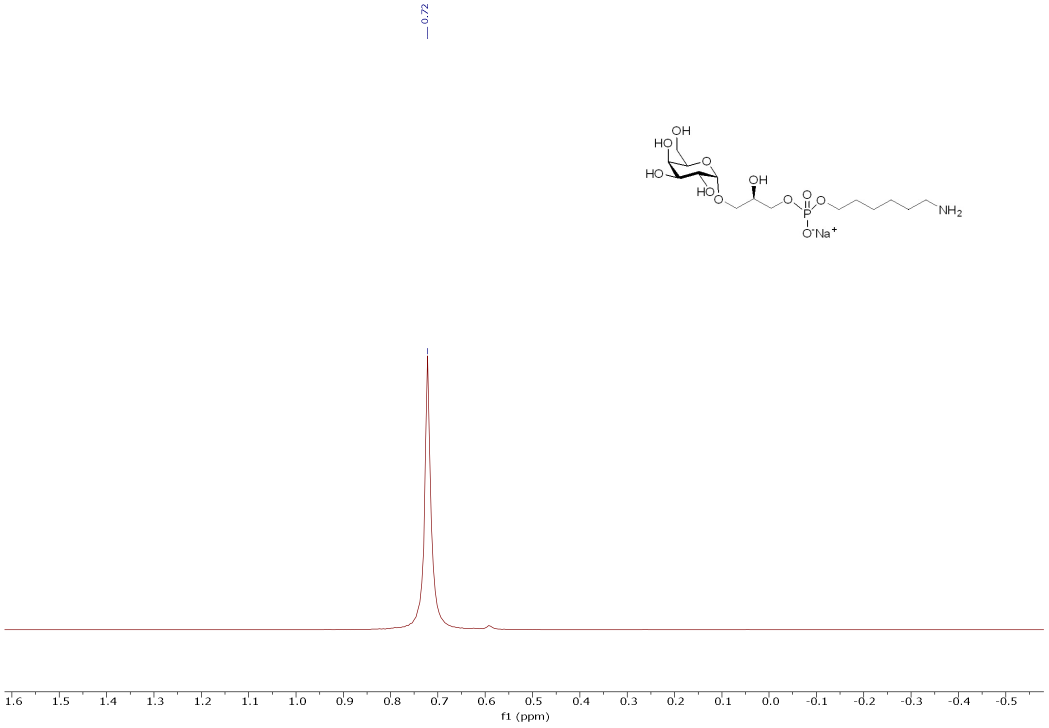

Supplementary Fig. 35 | NMR Spectra of compound 21.

a) ^1H NMR (500 MHz, $CDCl_3$)

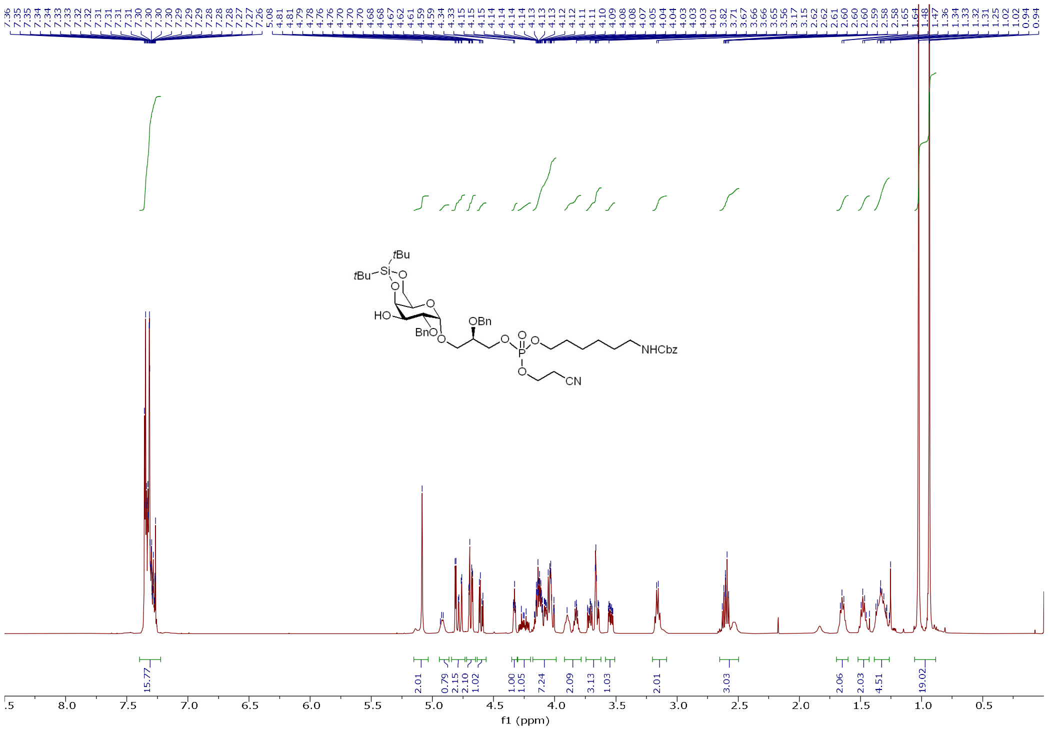

b) H-H COSY

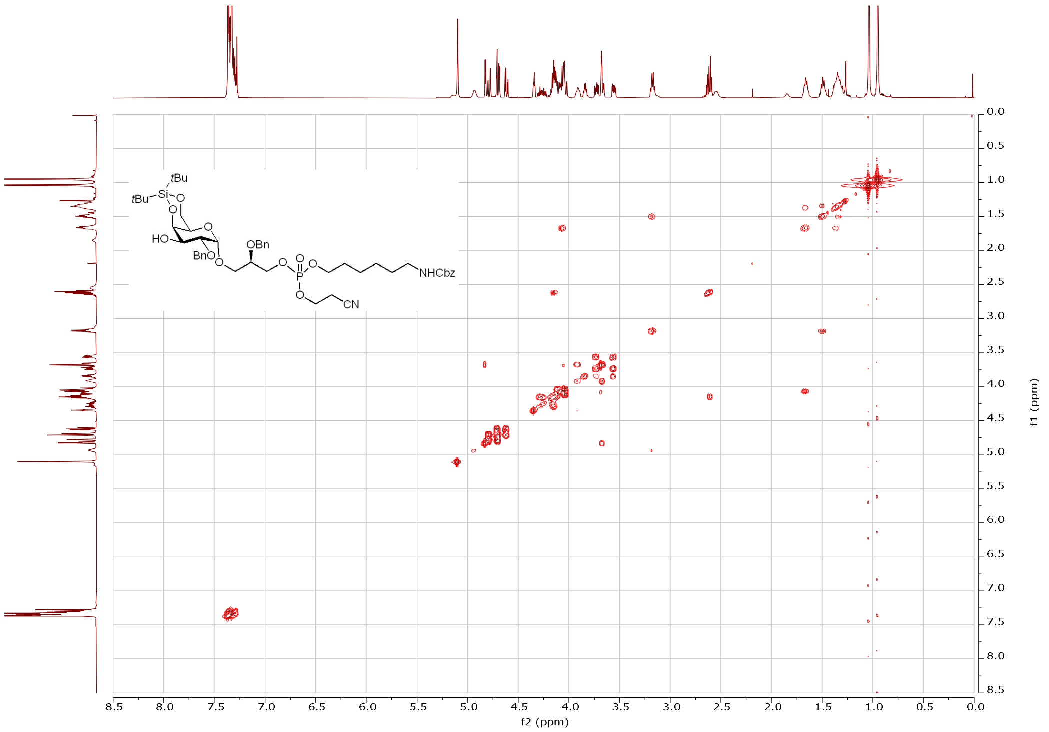

c) HSQC

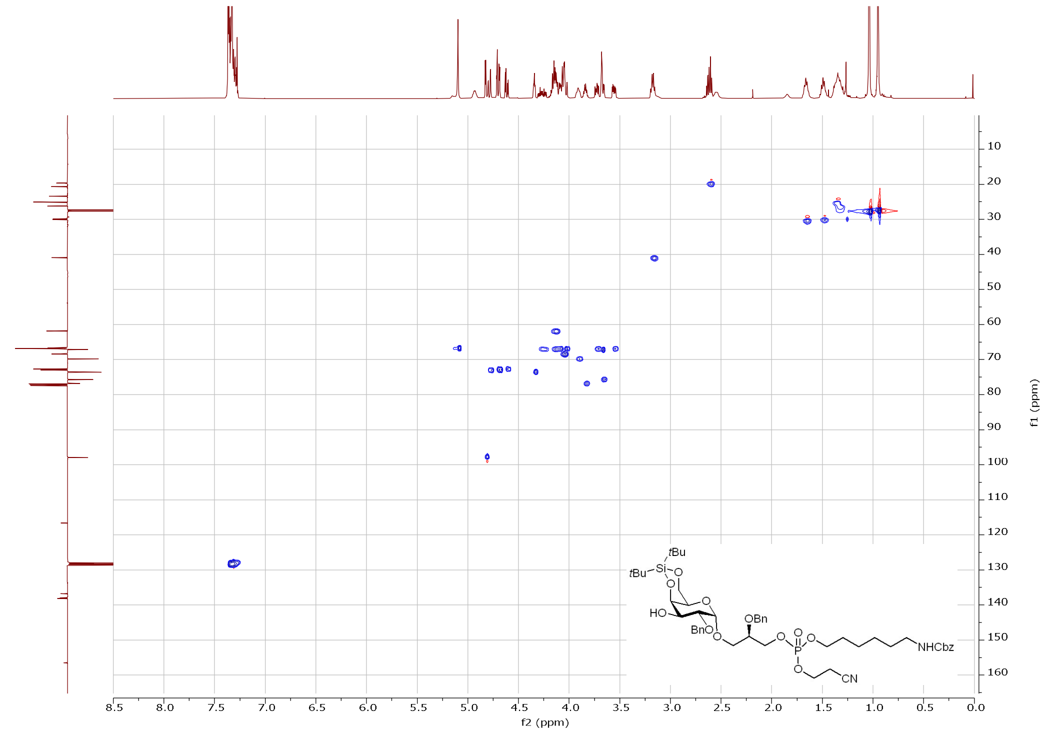

d) ^{13}C NMR (126 MHz, $CDCl_3$)

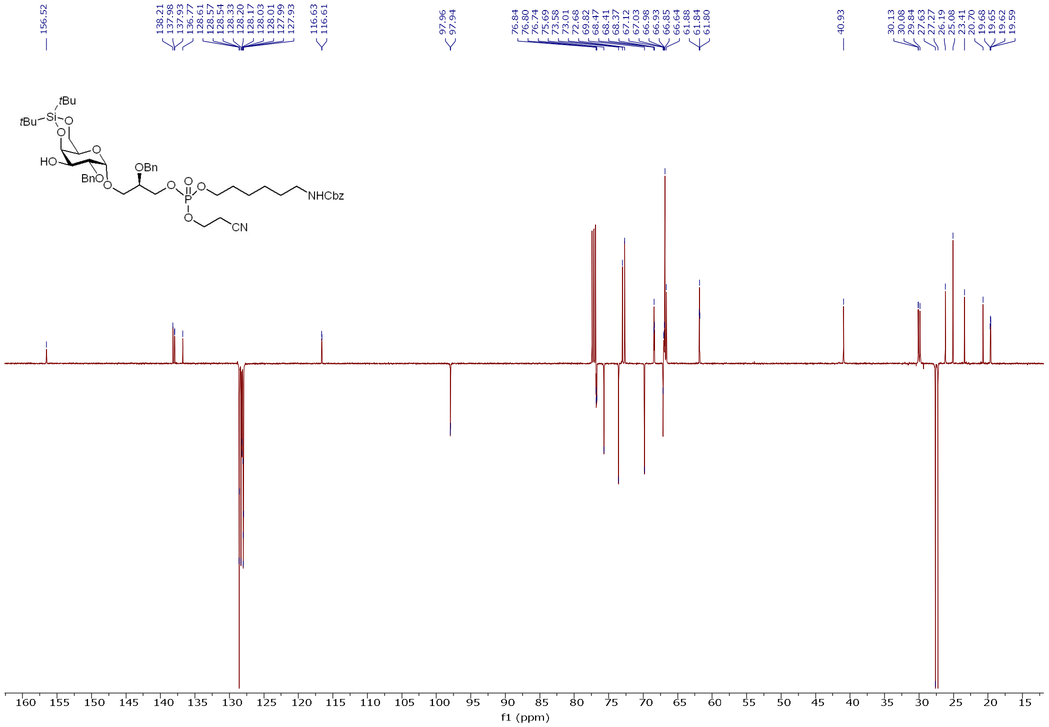

e) HMBC

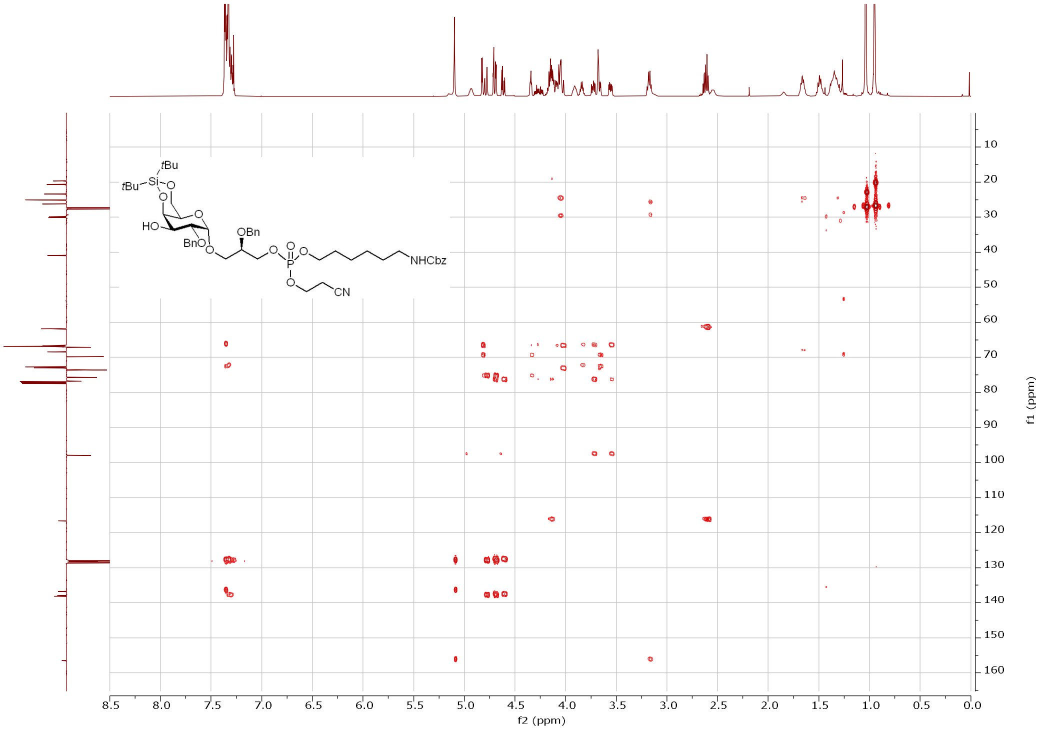

f) ^{31}P NMR (202 MHz, $CDCl_3$)

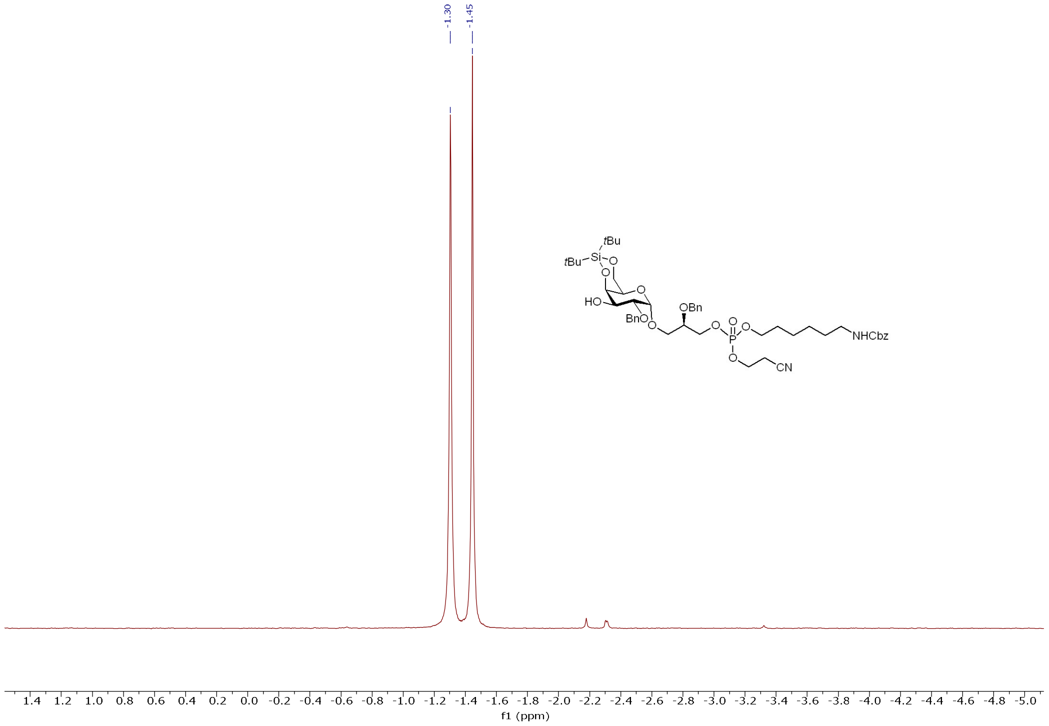

Supplementary Fig. 36 | NMR Spectra of compound 22.

a) ^{1}H NMR (500 MHz, *d*-acetone)

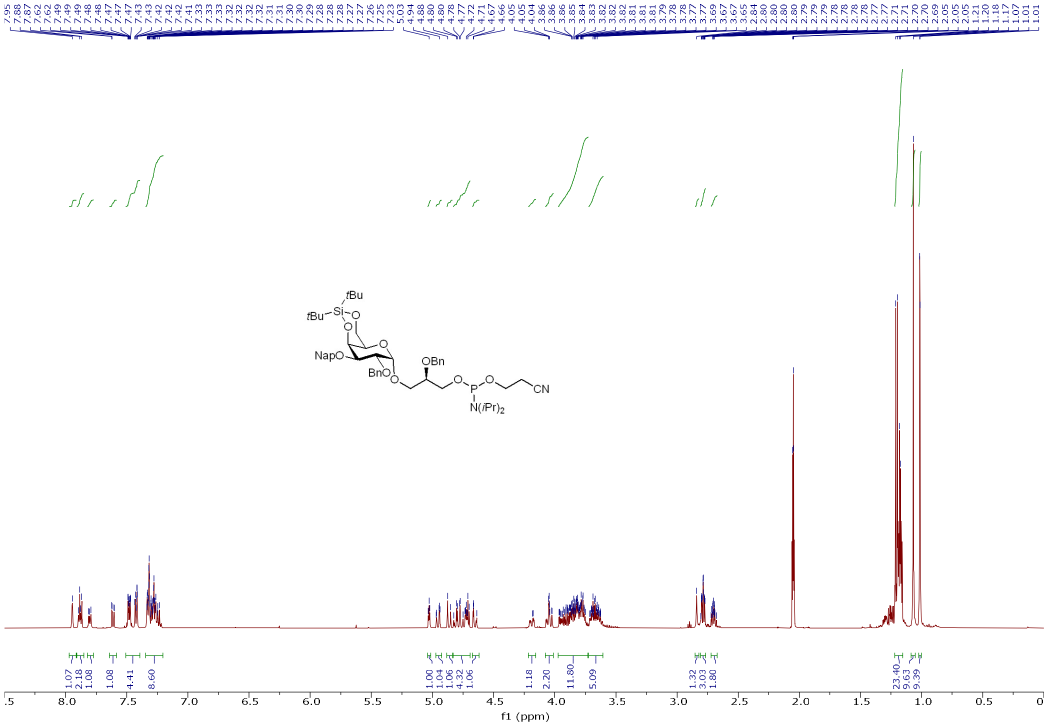

b) H-H COSY

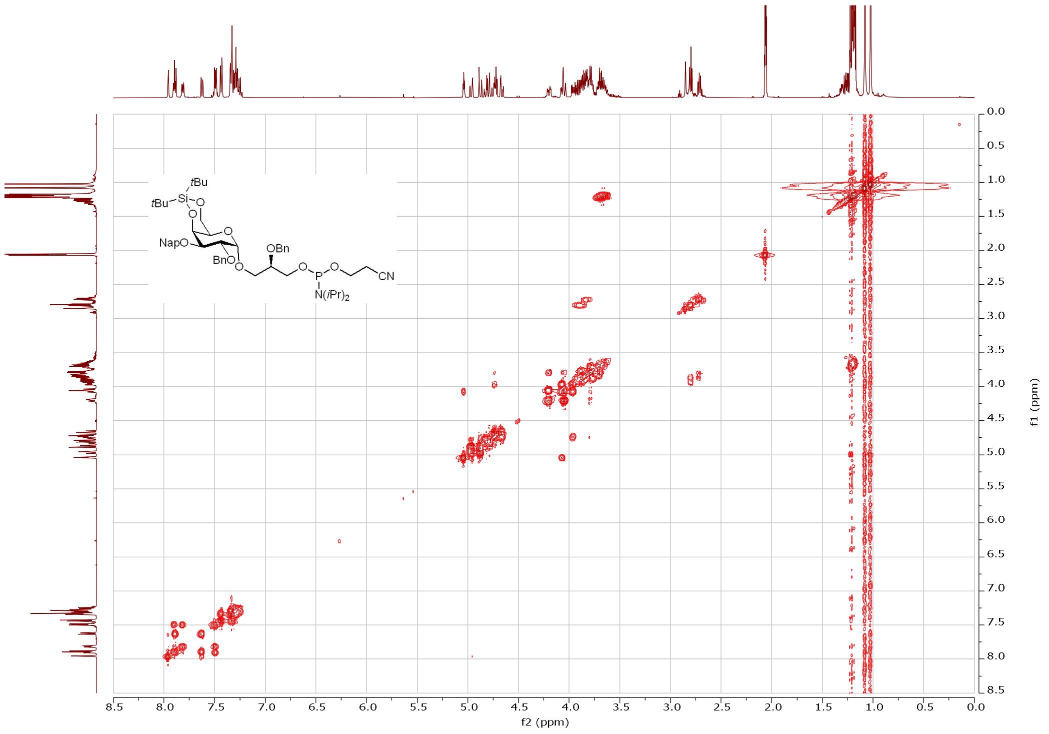

c) HSQC

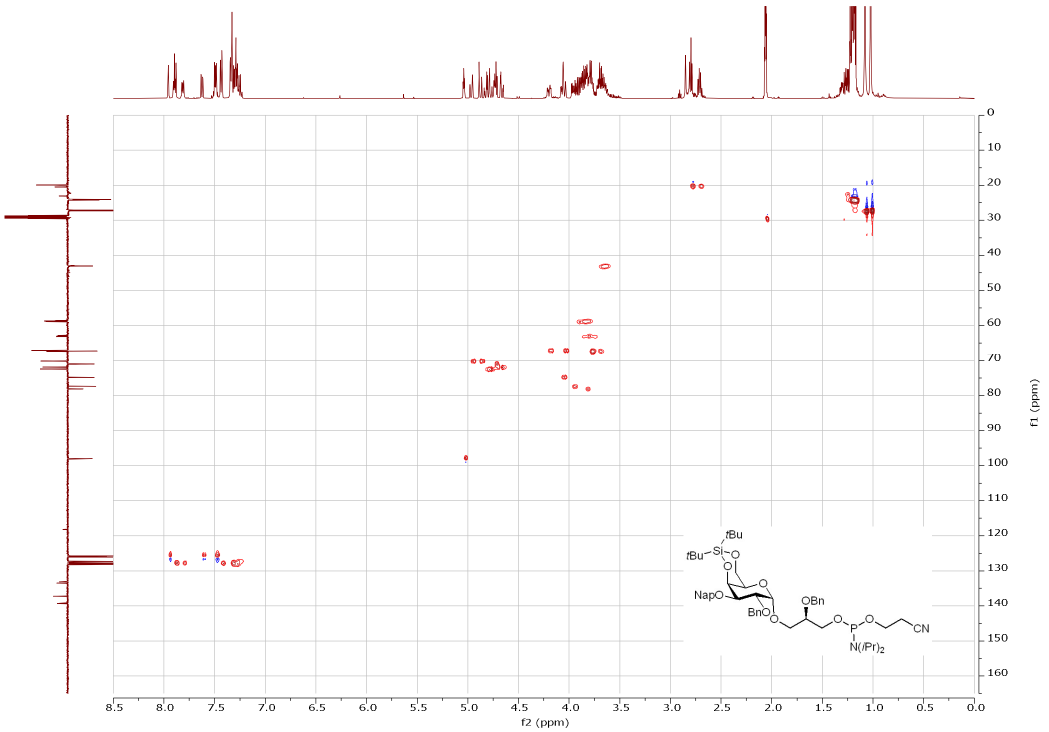

d) ^{13}C NMR (126 MHz, *d*-acetone)

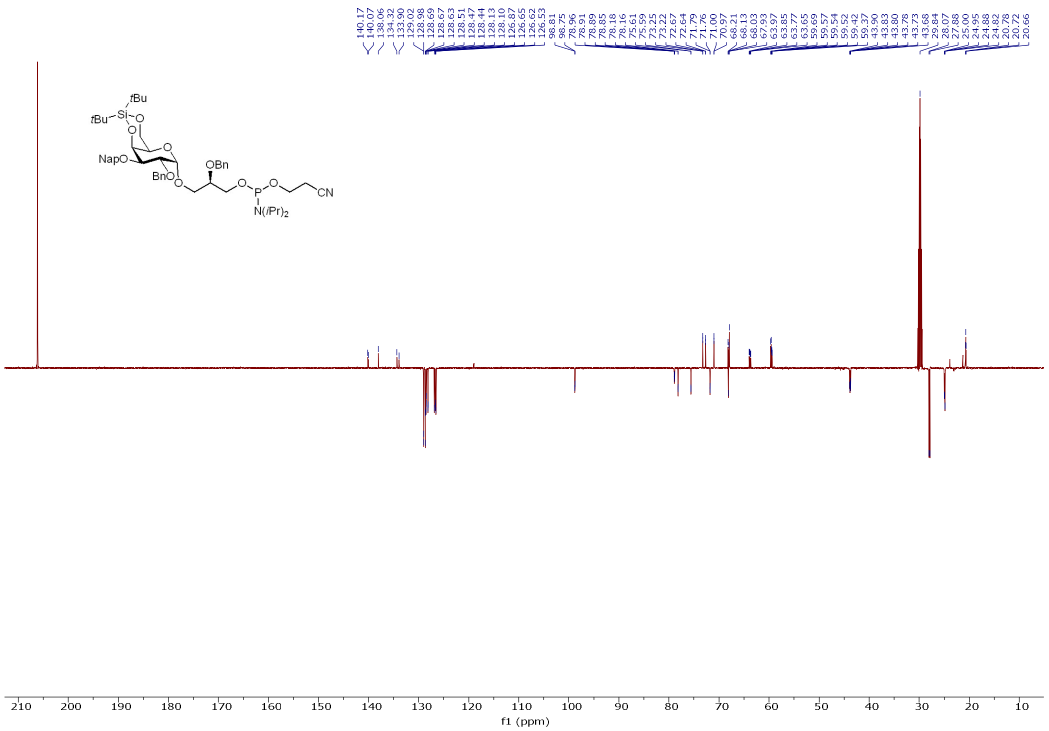

e) HMBC

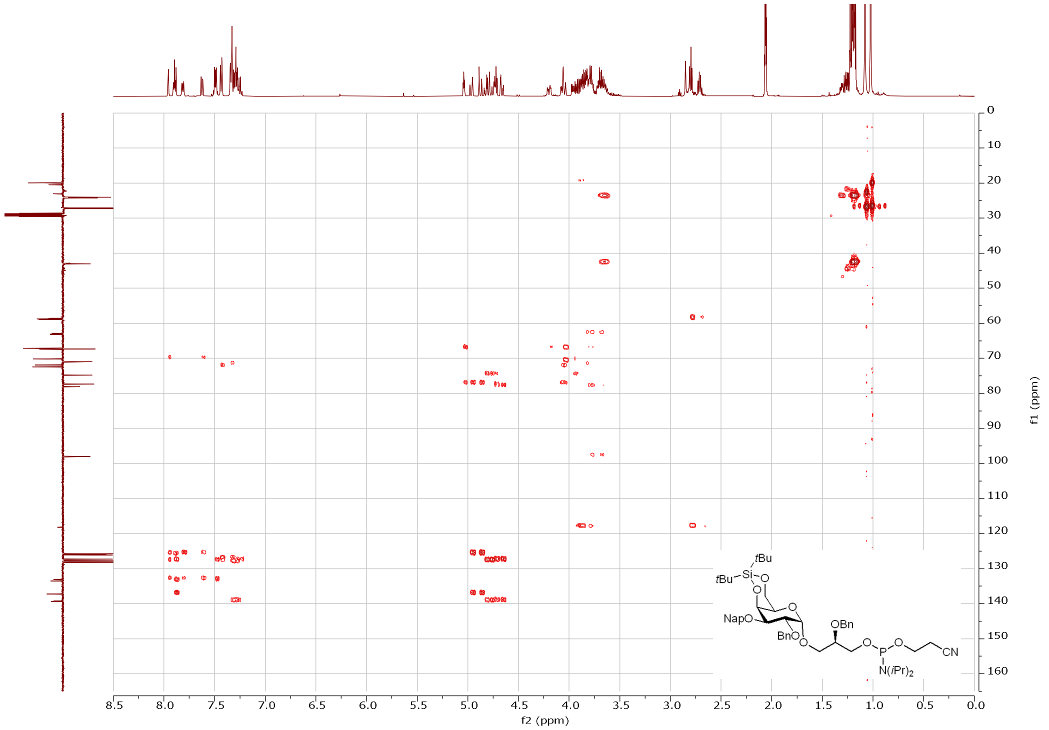

f) ^{31}P NMR (202 MHz, *d*-acetone)

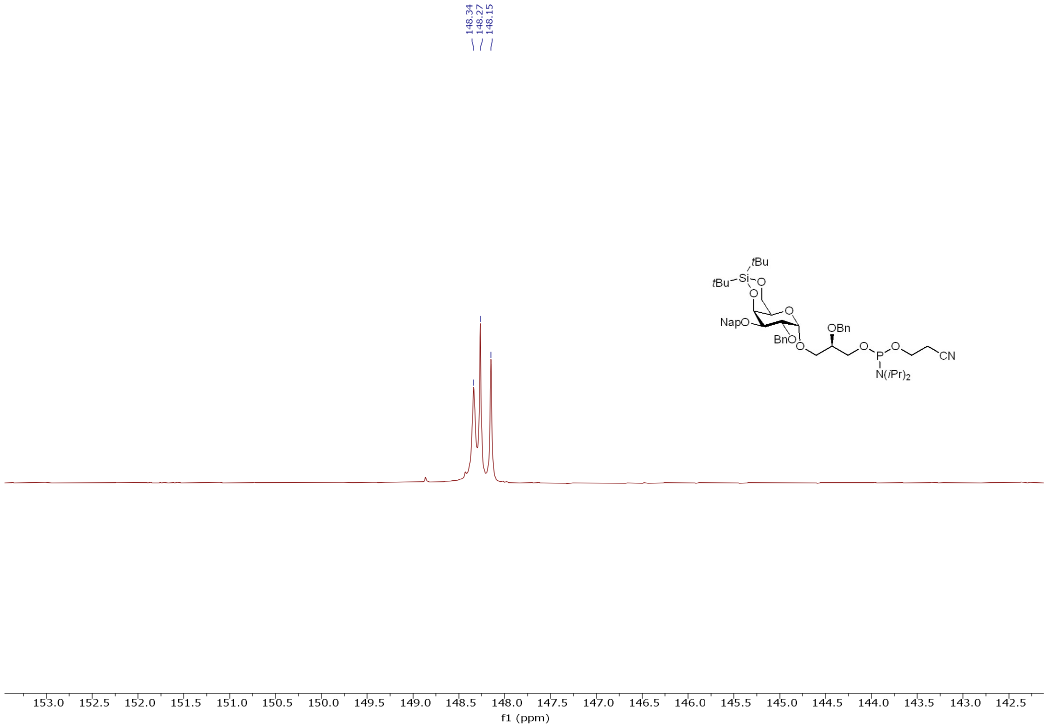

Supplementary Fig. 37 | NMR Spectra of compound 23.

a) ^{1}H NMR (500 MHz, $CDCl_3$)

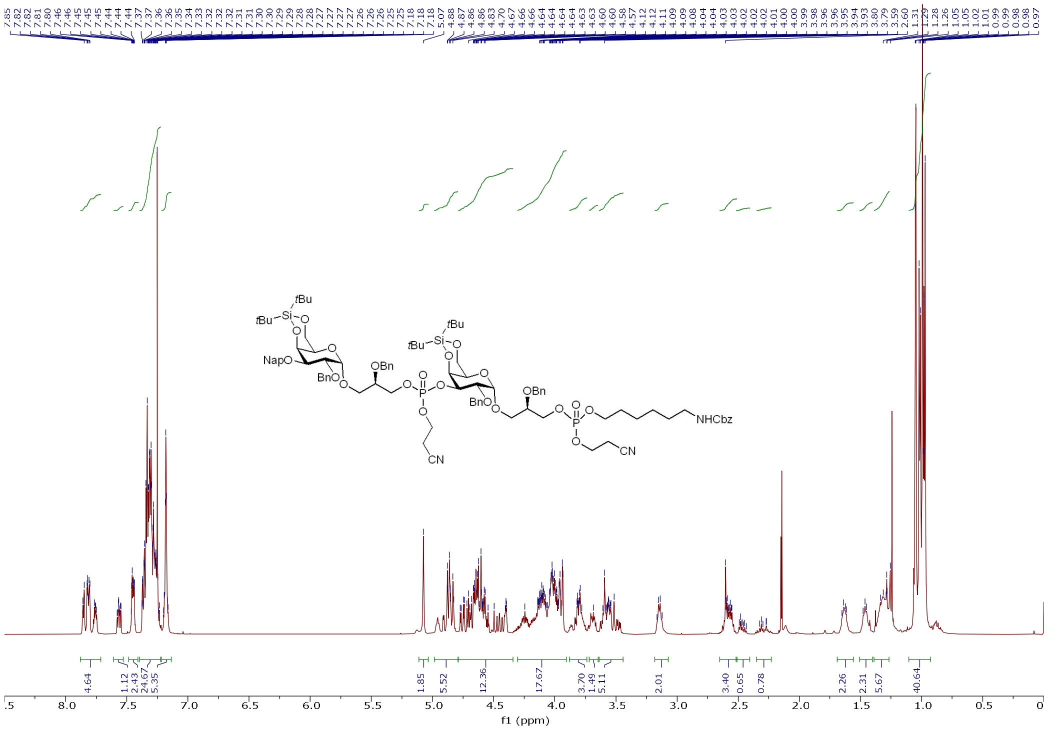

b) H-H COSY

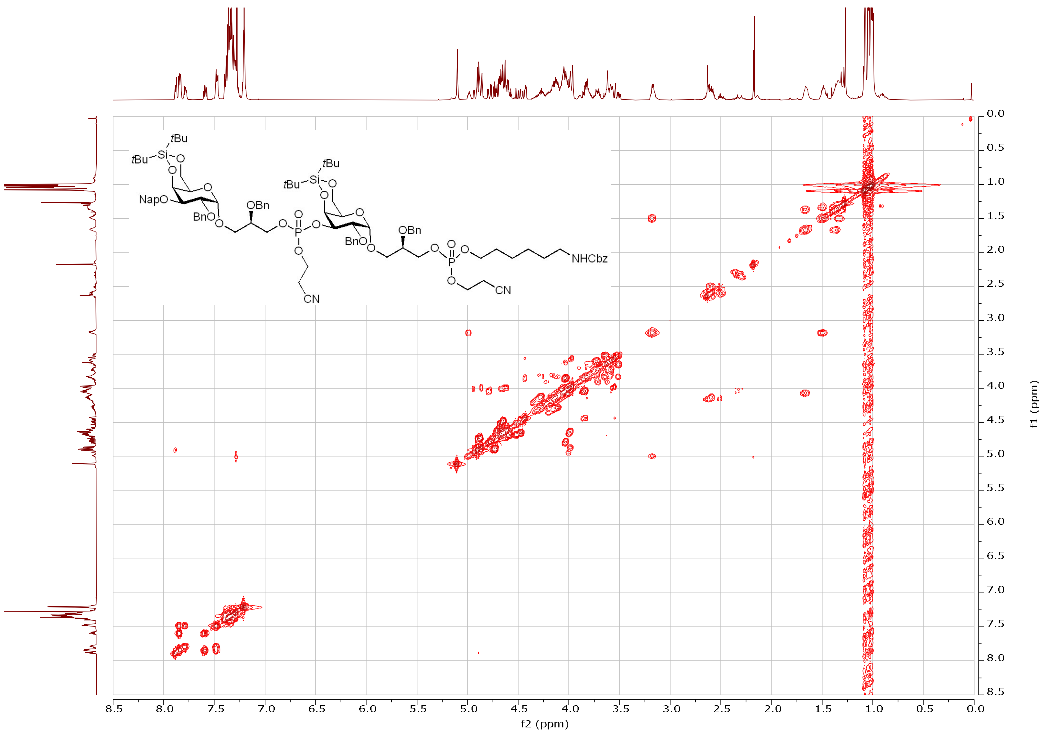

c) HSQC

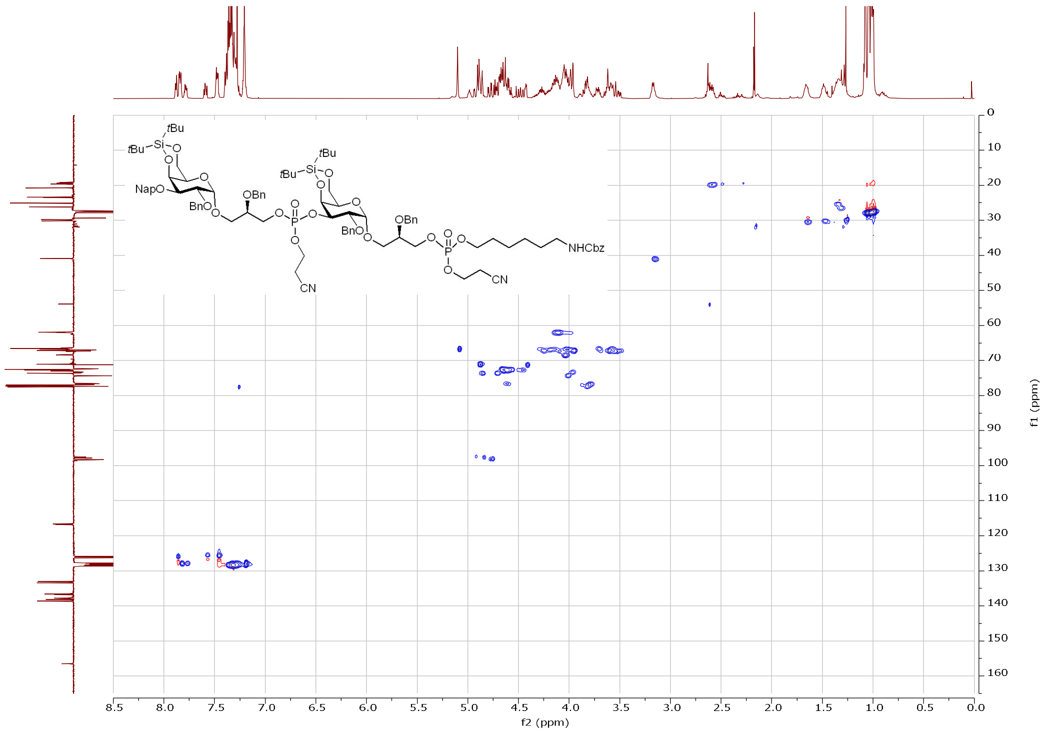

d) ^{13}C NMR (126 MHz, $CDCl_3$)

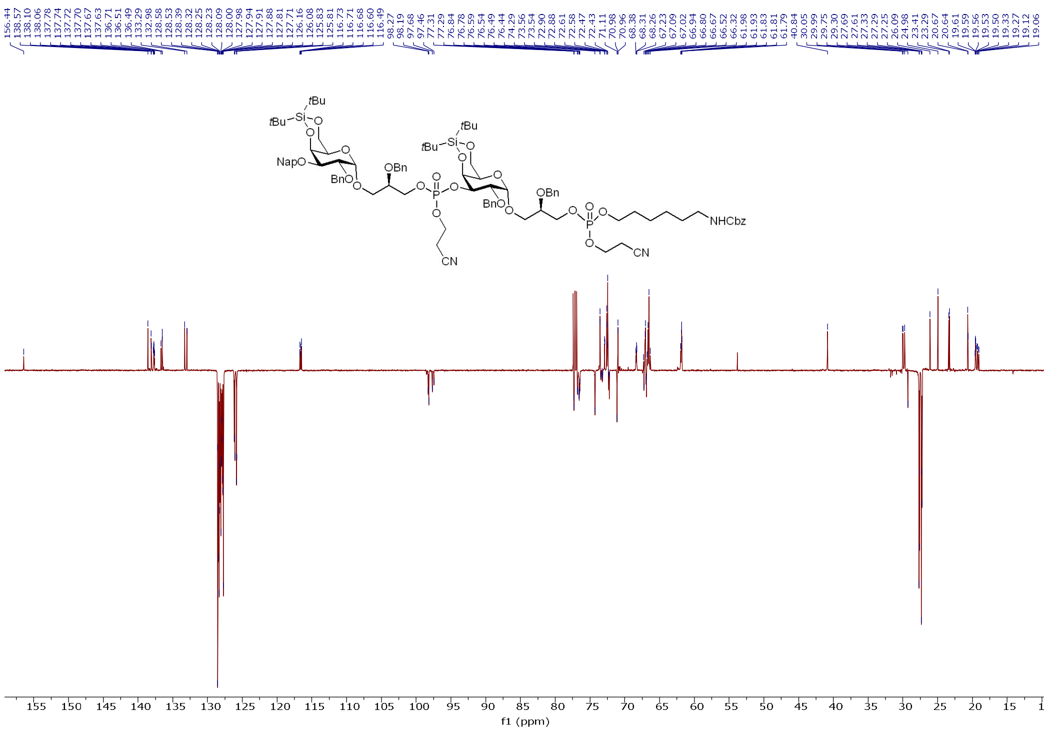

e) HMBC

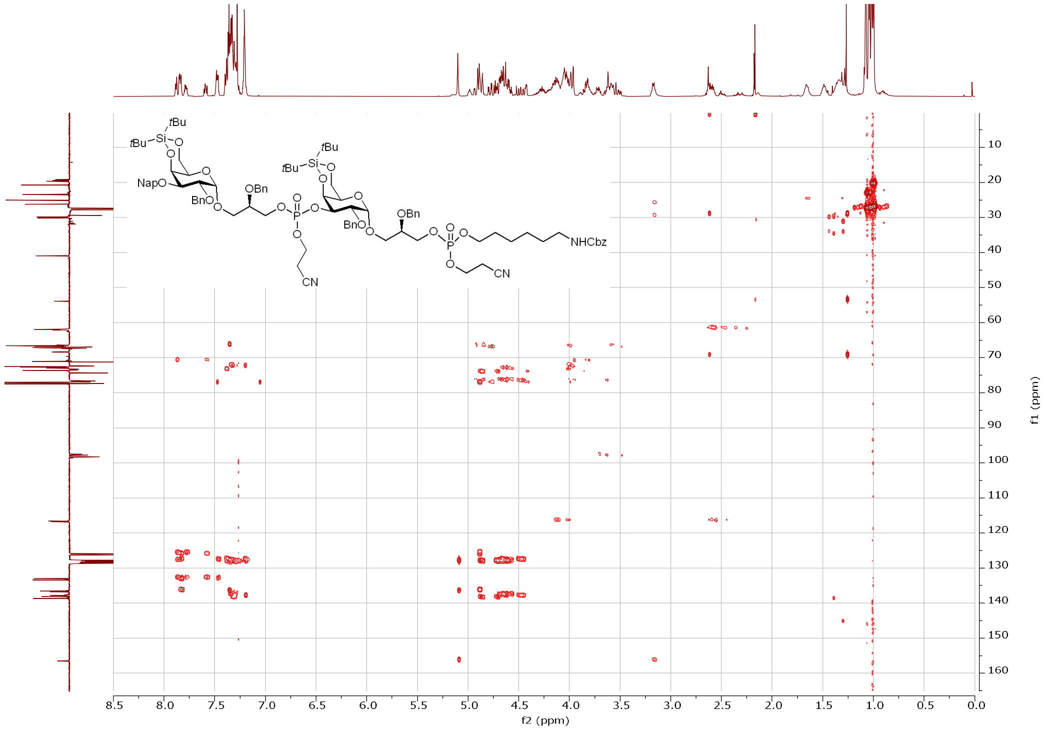

f) ^{31}P NMR (202 MHz, $CDCl_3$)

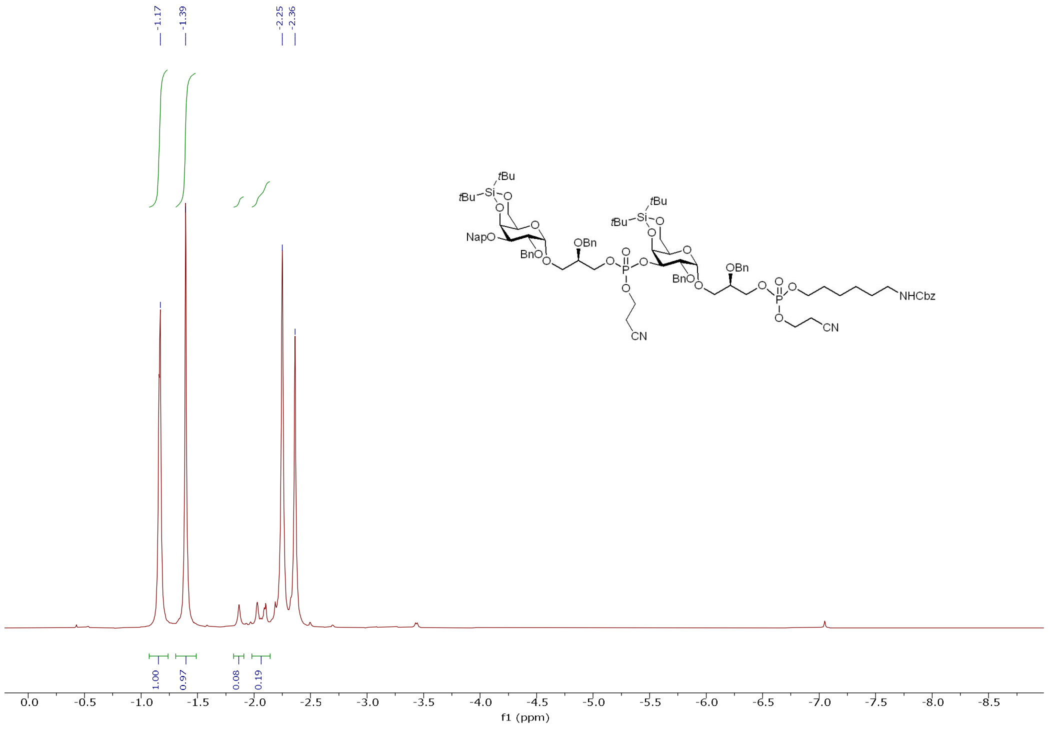

Supplementary Fig. 38 | NMR Spectra of compound 24.

a) ^{1}H NMR (400 MHz, $CDCl_3$)

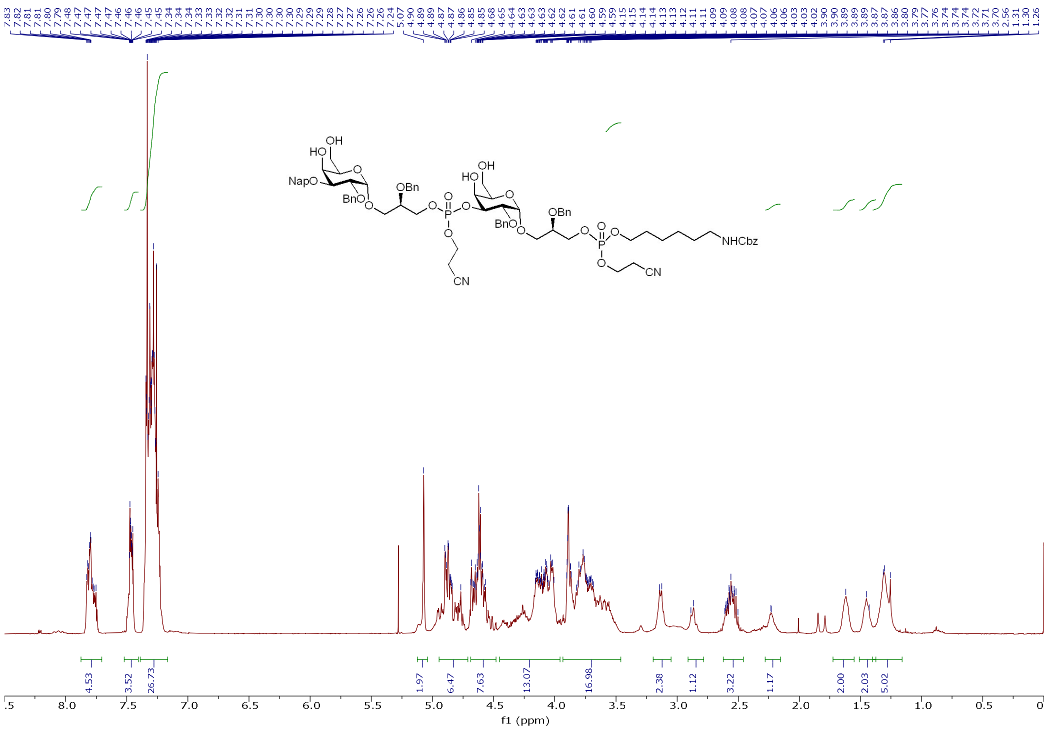

b) ^{13}C NMR (126 MHz, $CDCl_3$)

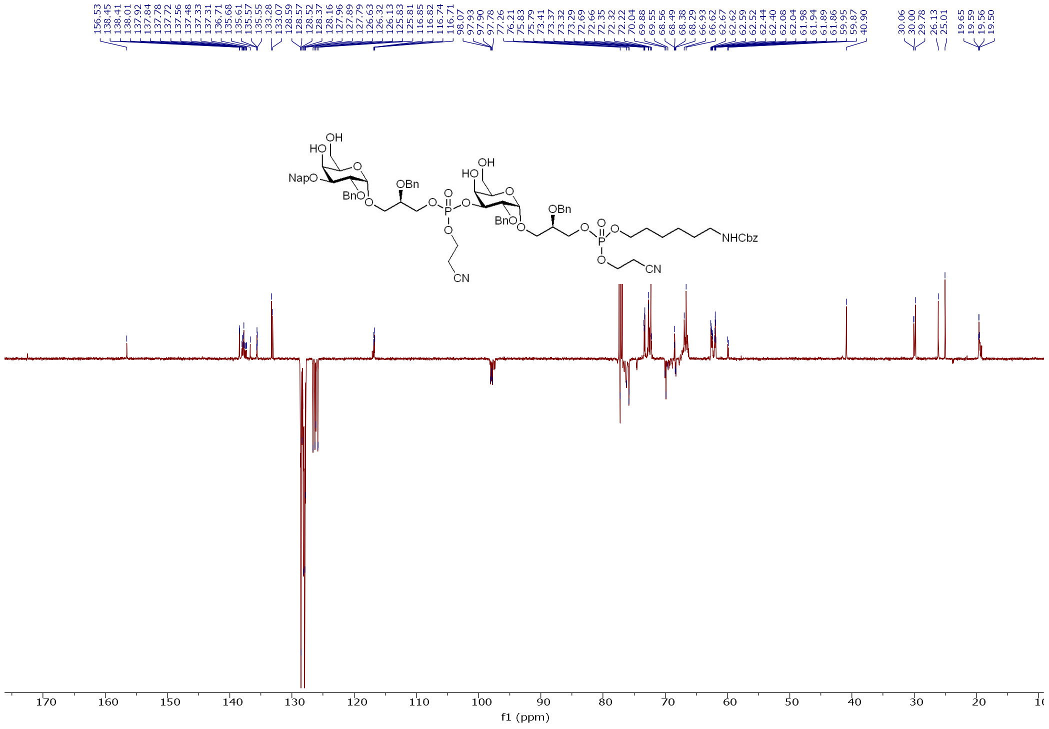

Supplementary Fig. 39 | NMR Spectra of compound 25.

a) ^{1}H NMR (500 MHz, D_2O)

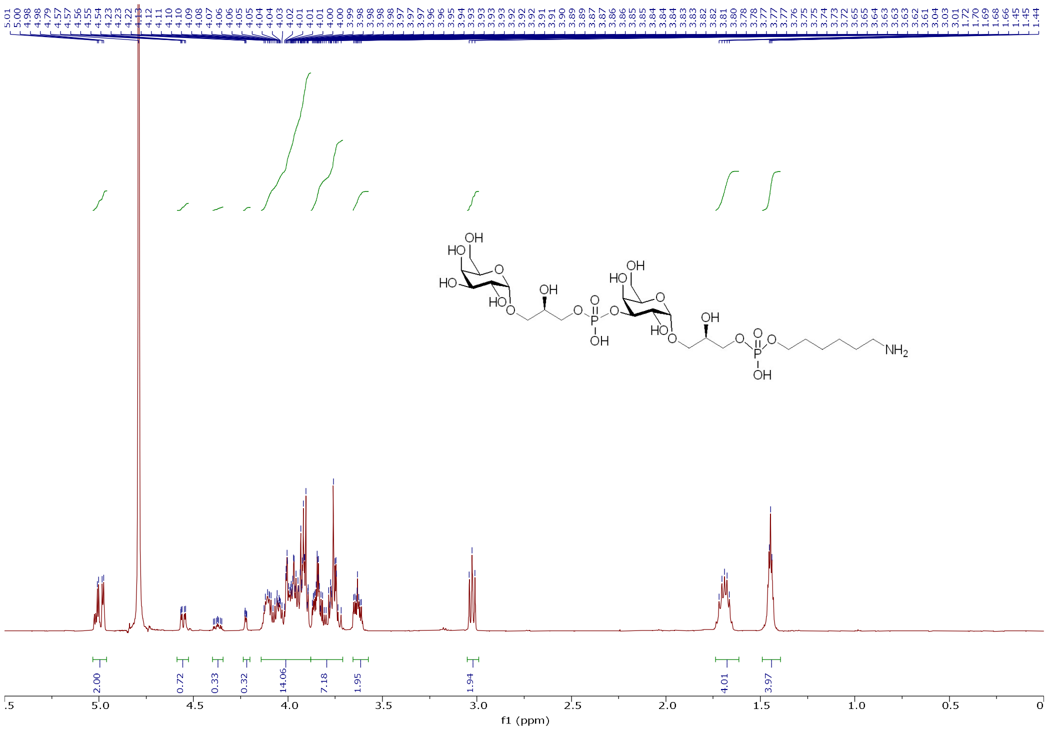

b) H-H COSY

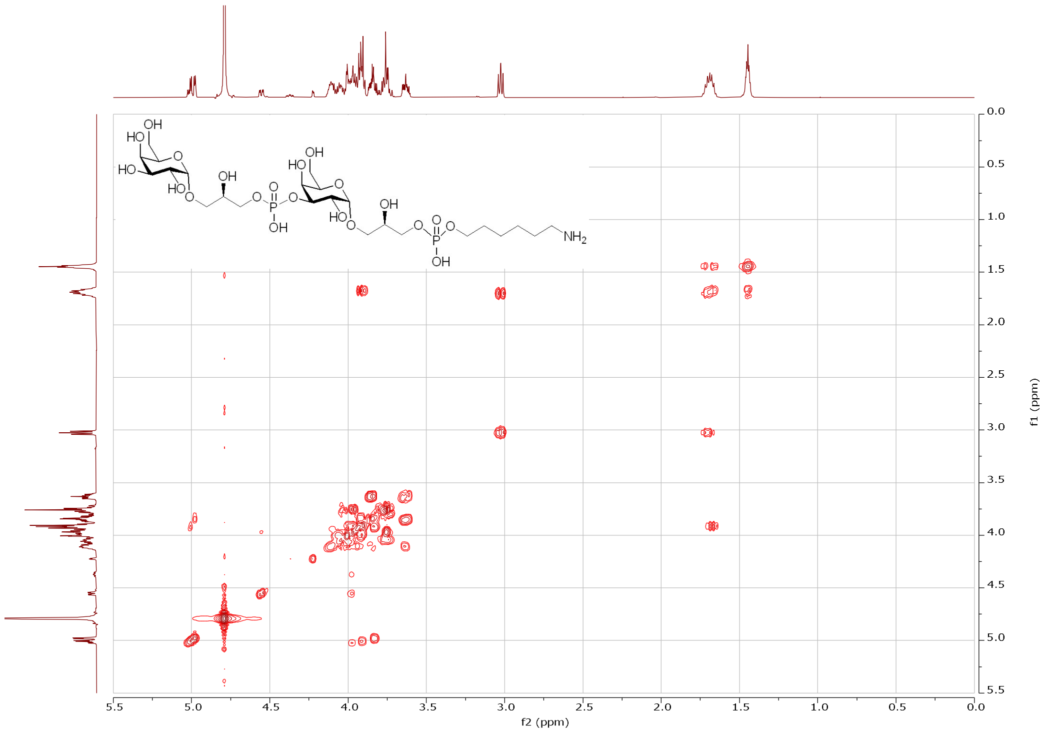

c) HSQC

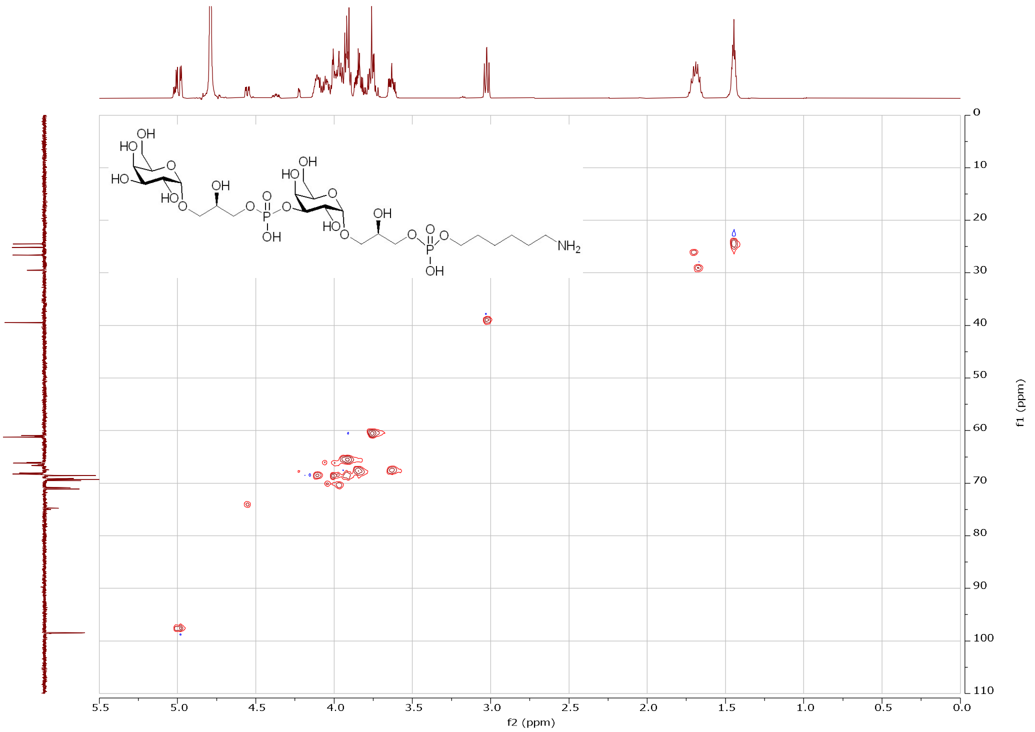

d) ^{13}C NMR (126 MHz, D_2O)

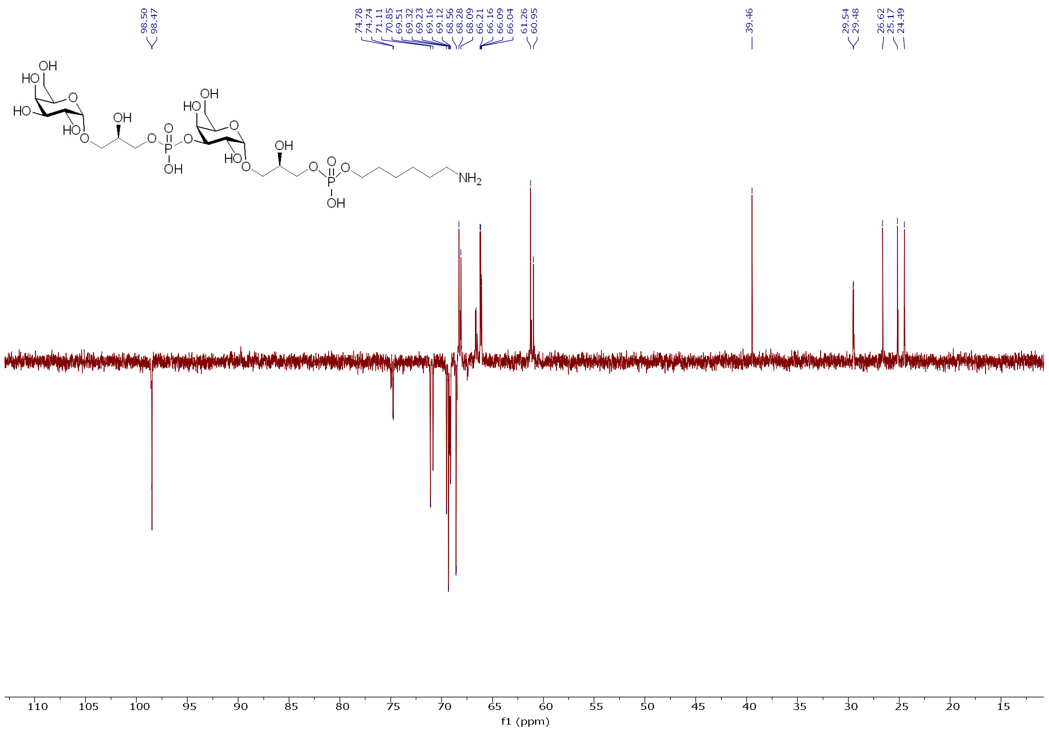

e) ^{31}P NMR (202 MHz, D_2O)

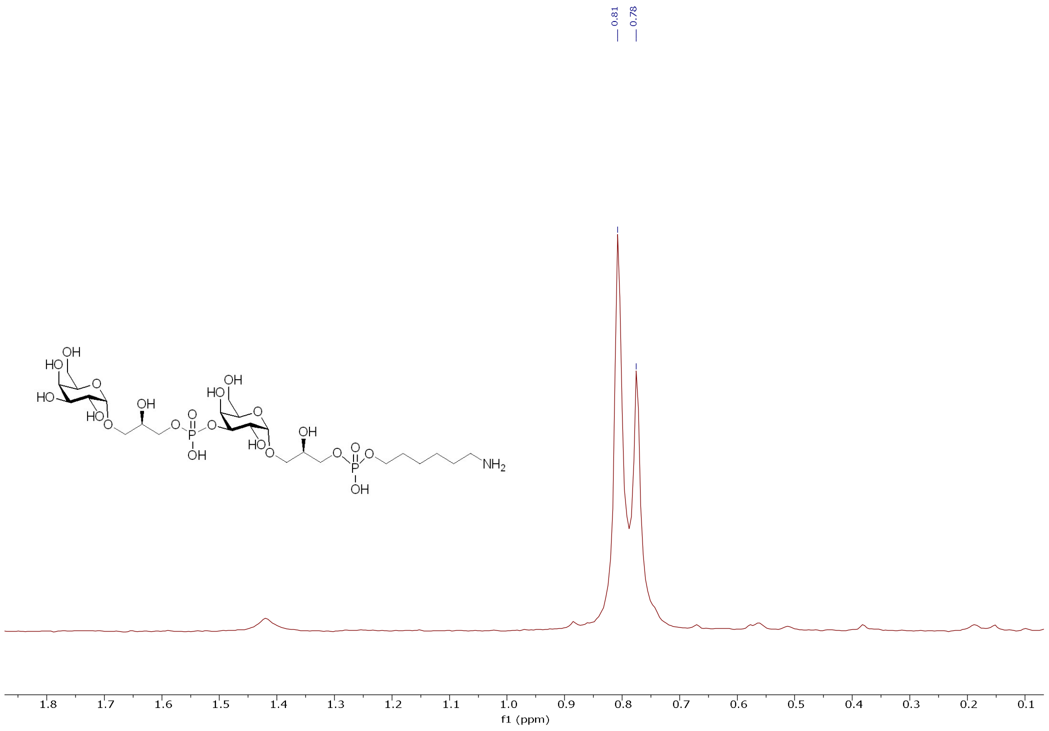

Supplementary Fig. 40 | NMR Spectra of compound 6.

a) ^{1}H NMR (850 MHz, D_2O)

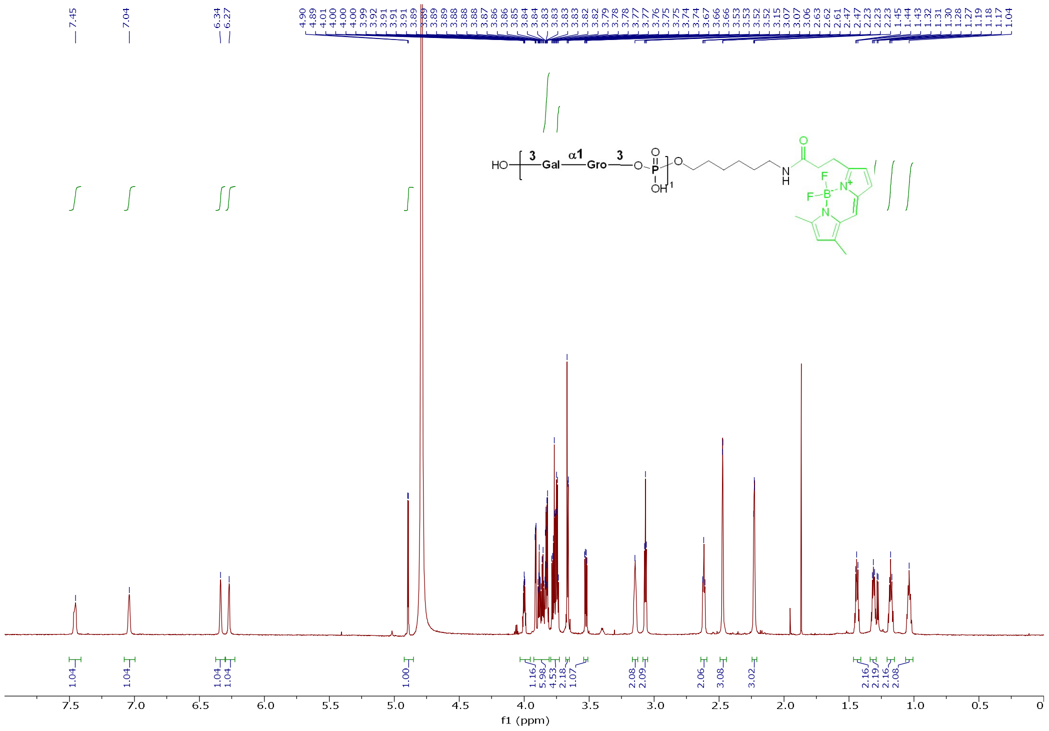

b) H-H COSY

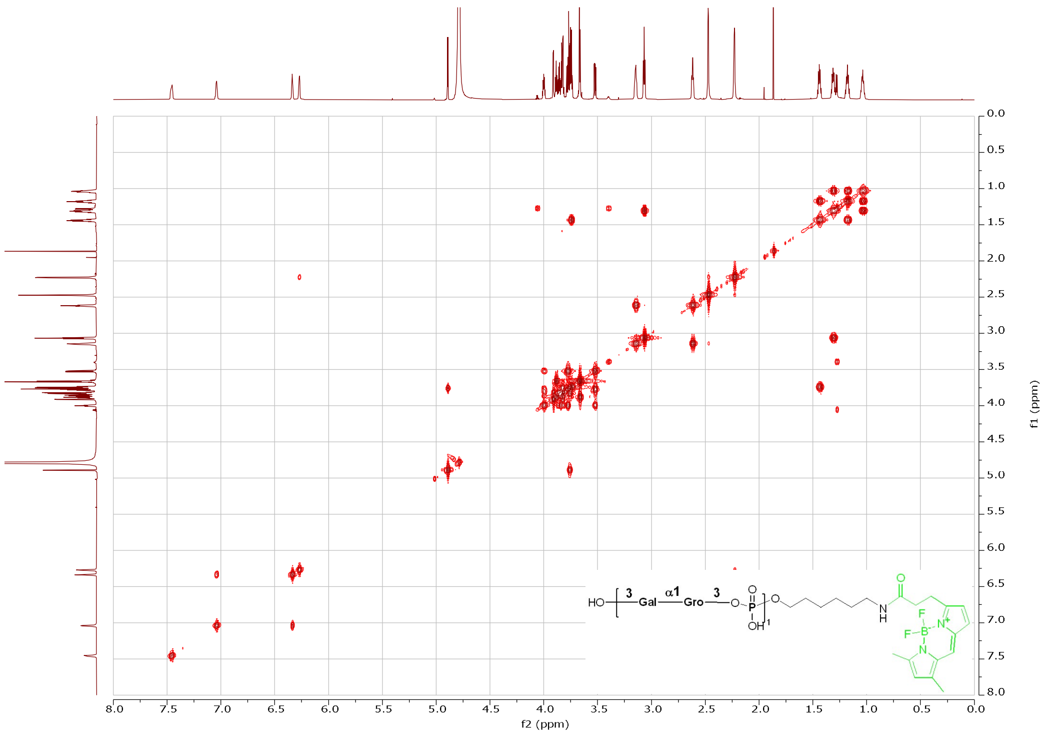

c) HSQC

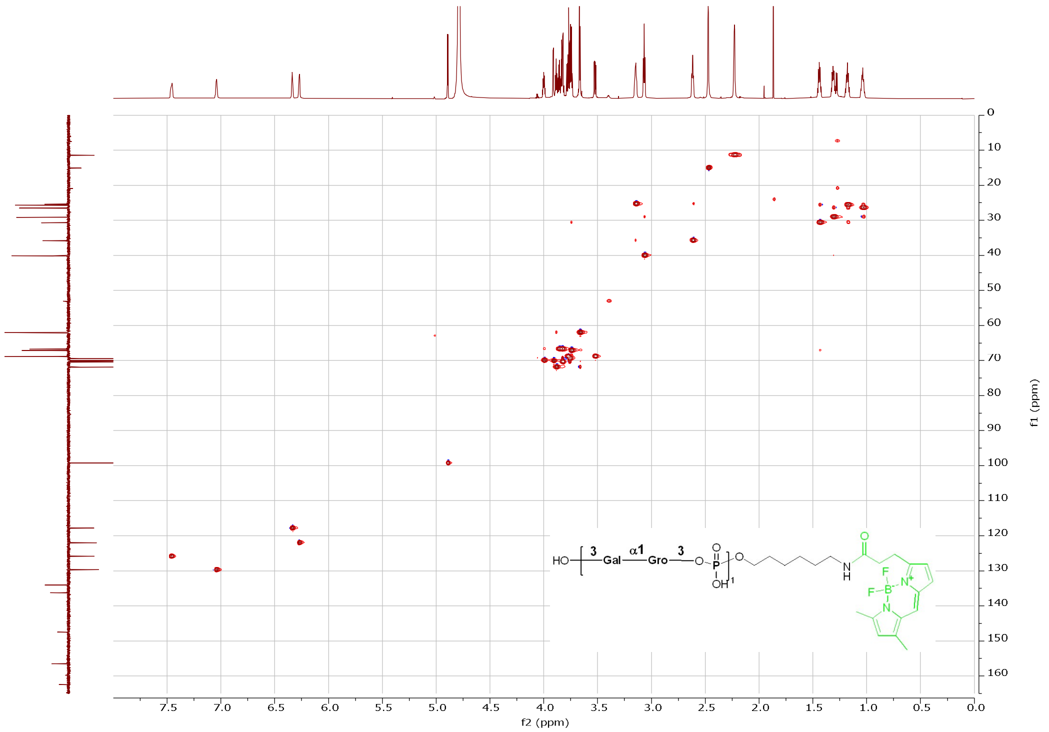

d) ^{13}C NMR (214 MHz, D_2O)

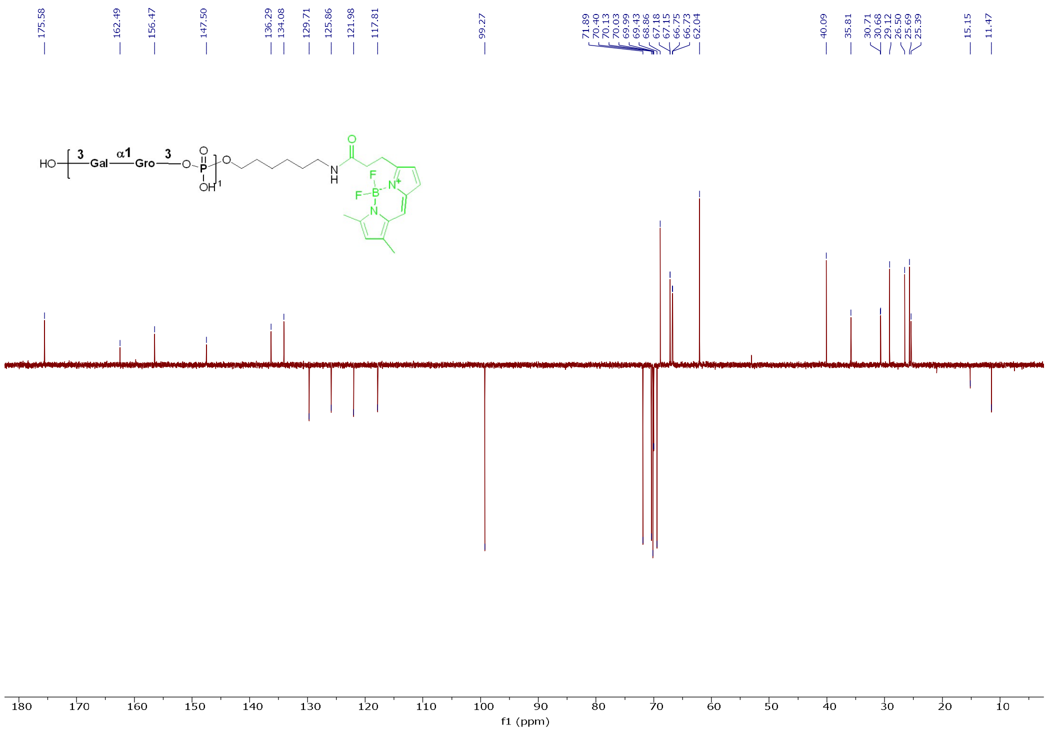

e) HMBC

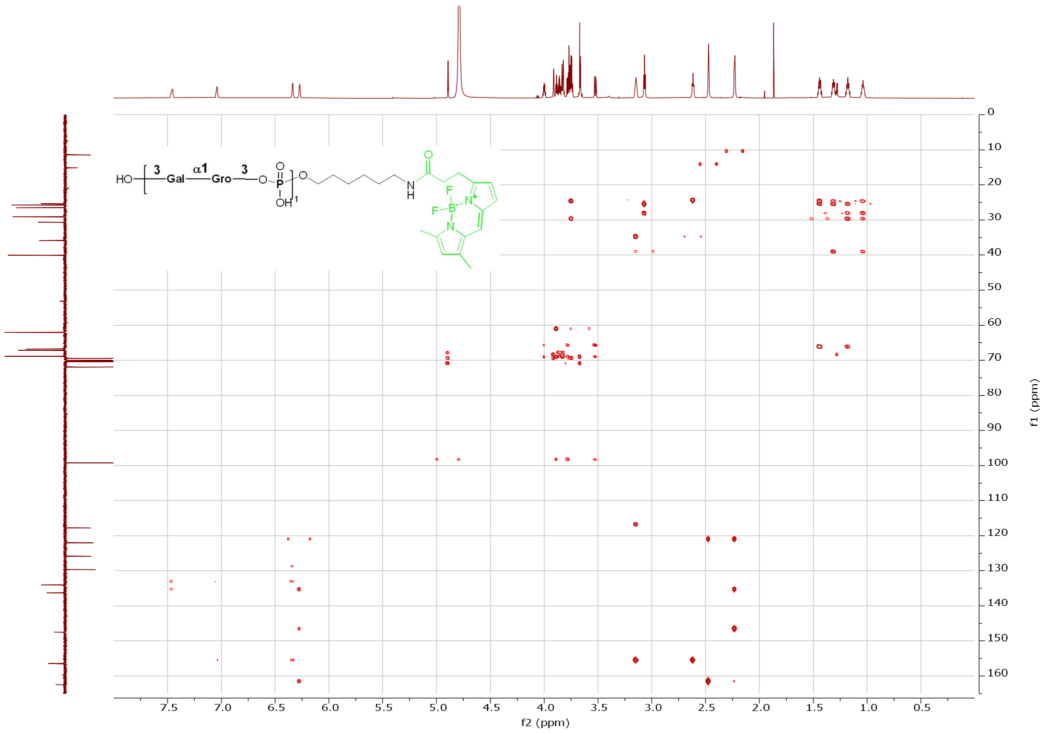

Supplementary Fig. 41 | NMR Spectra of compound 7.

a) ^{1}H NMR (850 MHz, D_2O)

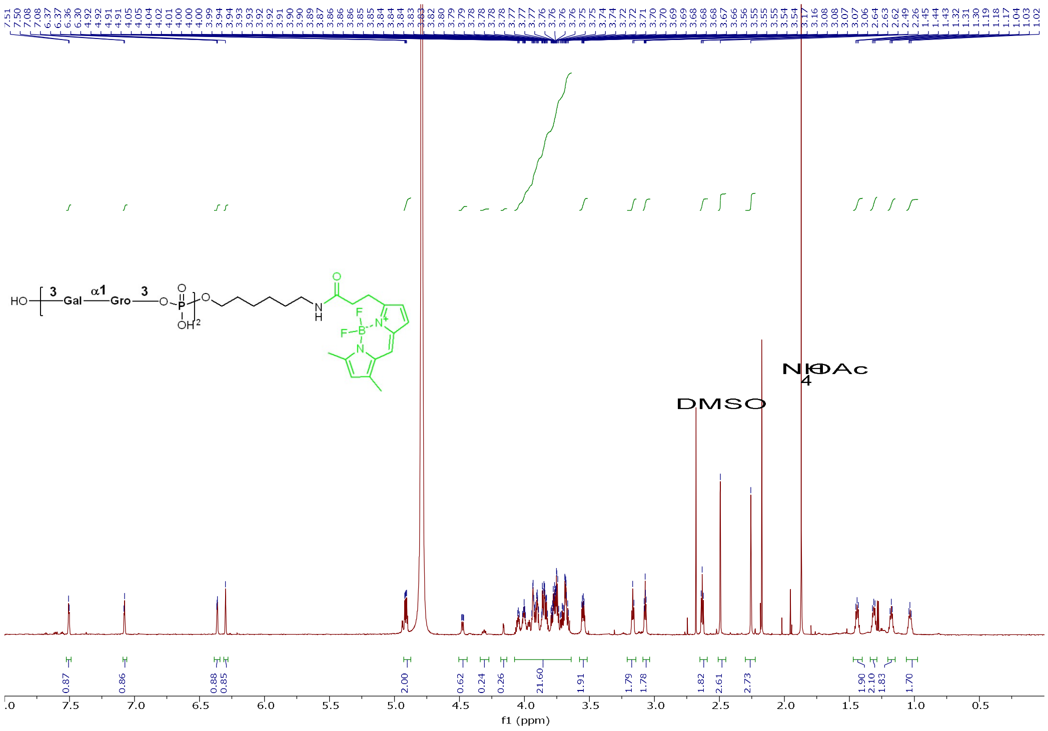

b) H-H COSY

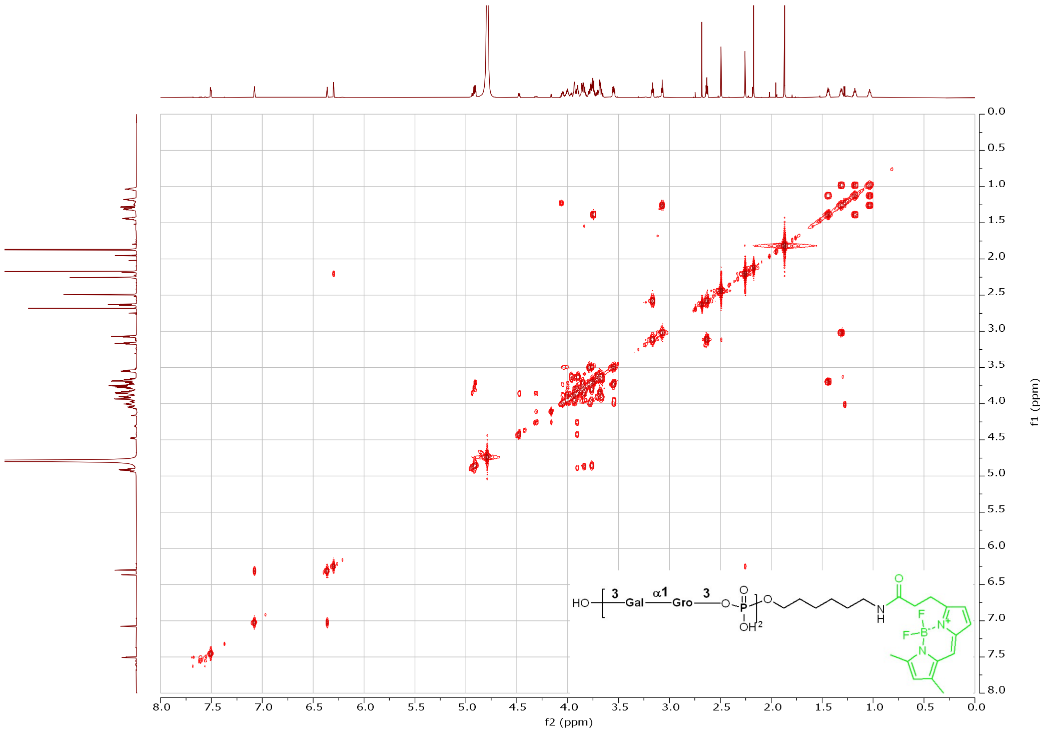

c) HSQC

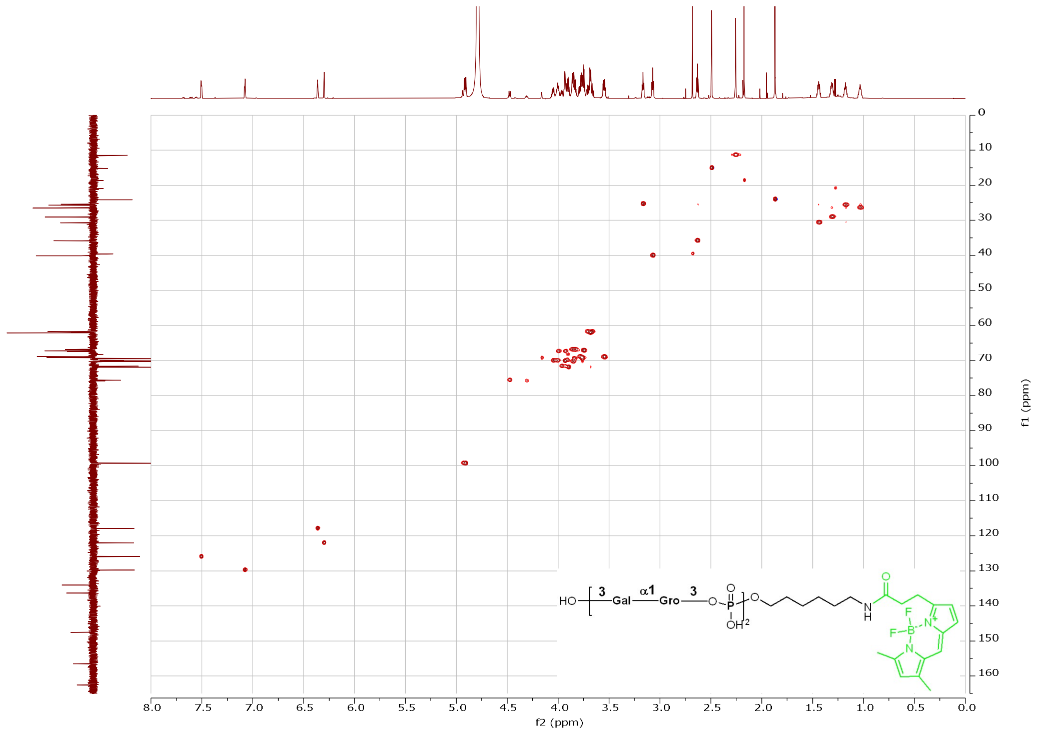

d) ^{13}C NMR (214MHz, $CDCl_3$)

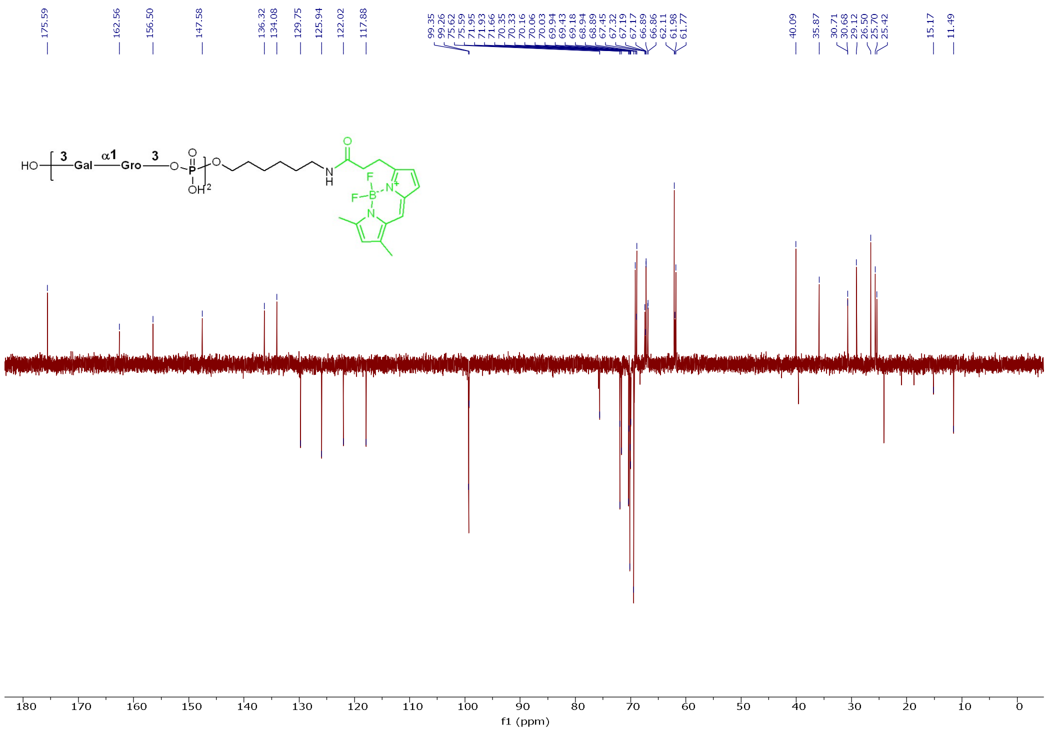

Supplementary References

1. Litschko, C. *et al.* Mix-and-Match System for the Enzymatic Synthesis of Enantiopure Glycerol-3-Phosphate-Containing Capsule Polymer Backbones from Actinobacillus pleuropneumoniae , Neisseria meningitidis , and Bibersteinia trehalosi. *mBio* **12**, e0089721 (2021).

2. Litschko, C. *et al.* A New Family of Capsule Polymerases Generates Teichoic Acid-Like Capsule Polymers in Gram-Negative Pathogens. *mBio* **9**, 16017 (2018).

3. Fiebig, T. *et al.* Molecular cloning and functional characterization of components of the capsule biosynthesis complex of Neisseria meningitidis serogroup A: toward in vitro vaccine production. *J Biol Chem* **289**, 19395–407 (2014).

4. Sulewska, M. *et al.* Extending the enzymatic toolbox for heparosan polymerization, depolymerization, and detection. *Carbohydr Polym* **319**, 121182 (2023).

5. Romanow, A. *et al.* Dissection of hexosyl- and sialyltransferase domains in the bifunctional capsule polymerases from Neisseria meningitidis W and Y defines a new sialyltransferase family. *J Biol Chem* **289**, 33945–57 (2014).

6. Keys, T. G. *et al.* A universal fluorescent acceptor for high-performance liquid chromatography analysis of pro- and eukaryotic polysialyltransferases. *Anal Biochem* **427**, 107–115 (2012).

7. Sievers, F. *et al.* Fast, scalable generation of high-quality protein multiple sequence alignments using Clustal Omega. *Mol Syst Biol* **7**, 539 (2011).

8. Kelley, L. A., Mezulis, S., Yates, C. M., Wass, M. N. & Sternberg, M. J. E. The Phyre2 web portal for protein modeling, prediction and analysis. *Nat Protoc* **10**, 845–58 (2015).

9. Jumper, J. *et al.* Highly accurate protein structure prediction with AlphaFold. *Nature* **596**, 583–589 (2021).

10. Lovering, A. L. *et al.* Structure of the bacterial teichoic acid polymerase TagF provides insights into membrane association and catalysis. *Nat Struct Mol Biol* **17**, 582–9 (2010).

11. Schertzer, J. W., Bhavsar, A. P. & Brown, E. D. Two conserved histidine residues are critical to the function of the TagF-like family of enzymes. *Journal of Biological Chemistry* **280**, 36683–36690 (2005).

12. Cifuente, J. O. *et al.* A multi-enzyme machine polymerizes the Haemophilus influenzae type b capsule. *Nat Chem Biol* **19**, 865–877 (2023).

13. Sobhanifar, S. *et al.* Structure and mechanism of Staphylococcus aureus TarM, the wall teichoic acid α-glycosyltransferase. *Proc Natl Acad Sci U S A* **112**, E576-85 (2015).

14. Guerin, M. E. *et al.* Molecular Recognition and Interfacial Catalysis by the Essential Phosphatidylinositol Mannosyltransferase PimA from Mycobacteria. *Journal of Biological Chemistry* **282**, 20705–20714 (2007).

15. Keys, T. G., Berger, M. & Gerardy-Schahn, R. A high-throughput screen for polysialyltransferase activity. *Anal Biochem* **427**, 60–68 (2012).

16. Fiebig, T. *et al.* Efficient solid-phase synthesis of meningococcal capsular oligosaccharides enables simple and fast chemoenzymatic vaccine production. *J Biol Chem* **293**, 953–962 (2018).

17. Doyle, L. *et al.* Mechanism and linkage specificities of the dual retaining β-Kdo glycosyltransferase modules of KpsC from bacterial capsule biosynthesis. *Journal of Biological Chemistry* **299**, 104609 (2023).

18. van der Es, D. *et al.* Streamlined Synthesis and Evaluation of Teichoic Acid Fragments. *Chemistry - A European Journal* **24**, 4014–4018 (2018).
