# Supplementary material for: Transition transferases prime bacterial capsule polymerization
Source: Nat Chem Biol. 2024 Jul 1;21(1):120–30. doi: 10.1038/s41589-024-01664-8 (PMC11666461; doi:10.1038/s41589-024-01664-8)
Supplement: Supplementary file 2 — Reporting Summary [file 41589_2024_1664_MOESM2_ESM.pdf]

nature portfolio

Corresponding author(s): Timm Fiebig
Marcelo Guerin

Last updated by author(s): Apr 23, 2024

Reporting Summary

Nature Portfolio wishes to improve the reproducibility of the work that we publish. This form provides structure for consistency and transparency in reporting. For further information on Nature Portfolio policies, see our Editorial Policies and the Editorial Policy Checklist.

Statistics

For all statistical analyses, confirm that the following items are present in the figure legend, table legend, main text, or Methods section.

n/a	Confirmed	
☒	☐	The exact sample size (*n*) for each experimental group/condition, given as a discrete number and unit of measurement
☒	☐	A statement on whether measurements were taken from distinct samples or whether the same sample was measured repeatedly
☒	☐	The statistical test(s) used AND whether they are one- or two-sided<br>*Only common tests should be described solely by name; describe more complex techniques in the Methods section.*
☒	☐	A description of all covariates tested
☒	☐	A description of any assumptions or corrections, such as tests of normality and adjustment for multiple comparisons
☒	☐	A full description of the statistical parameters including central tendency (e.g. means) or other basic estimates (e.g. regression coefficient) AND variation (e.g. standard deviation) or associated estimates of uncertainty (e.g. confidence intervals)
☒	☐	For null hypothesis testing, the test statistic (e.g. *F*, *t*, *r*) with confidence intervals, effect sizes, degrees of freedom and *P* value noted<br>*Give P values as exact values whenever suitable.*
☒	☐	For Bayesian analysis, information on the choice of priors and Markov chain Monte Carlo settings
☒	☐	For hierarchical and complex designs, identification of the appropriate level for tests and full reporting of outcomes
☒	☐	Estimates of effect sizes (e.g. Cohen's *d*, Pearson's *r*), indicating how they were calculated

Our web collection on statistics for biologists contains articles on many of the points above.

Software and code

Policy information about availability of computer code

Data collection	We did not generate any new software for our work. Data collection software used was:<br>- NMR: Data were collected with Topspin 3.6.1 (Bruker Biospin, Germany)<br>- HPLC: Data were collected with LCsolution version 1.25 SP4 (Shimadzu)<br>- Gels were scanned using Amersham Imager 680, Software: AI600_66830510 Version2.0.0

Data analysis

We did not generate any new software for our work. Data analysis software comprises:
NMR: Spectra were processed with Topspin 3.6.1 (Bruker Biospin, Germany) and analyzed with Sparky 3.115 (Goddard T.D., Kneller D.G. (2008); University of California, San Francisco, CA).
HPLC: Data analysis was performed with LCsolution version 1.25 SP4 (Shimadzu).
Mass spectrometry: Spectra were processed with Data Explorer Software V4.8
phenix-1.18.2-3874
coot 0.8.9.2
UCSF Chimera 1.14
Data Explorer Software V4.8
Phaser 2.8
CCP4 8.0 suite, AutoDock Vina
MolProbity 4.5.2
Clustal Omega, Online Version, available at https://www.ebi.ac.uk/Tools/msa/clustalo/
DALI server, a network service for comparing protein structures in 3D available at http://ekhidna2.biocenter.helsinki.fi/dali.
Alphafold, web service available at https://colab.research.google.com/github/sokrypton/ColabFold/blob/main/AlphaFold2.ipynb

For manuscripts utilizing custom algorithms or software that are central to the research but not yet described in published literature, software must be made available to editors and reviewers. We strongly encourage code deposition in a community repository (e.g. GitHub). See the Nature Portfolio guidelines for submitting code & software for further information.

Data

Policy information about availability of data

All manuscripts must include a data availability statement. This statement should provide the following information, where applicable:

- Accession codes, unique identifiers, or web links for publicly available datasets
- A description of any restrictions on data availability
- For clinical datasets or third party data, please ensure that the statement adheres to our policy

The atomic coordinates and structure factors have been deposited in the Protein Data Bank, accession codes 8QOY for Cps3D. Data collection and refinement statistics are presented in Supplementary Table 4. PDB IDs used in the analysis of this work include 3L7K, 3L7L, 1A9Z, 4WYI, 8A0C, 3OT5, 6N1X, 3OKA, 6TVP, 1A9Z. Accession codes for sequences used in this study are available in Supplementary Table 1. NMR chemical shifts are presented in Supplementary Tables 3 and 5.

Human research participants

Policy information about studies involving human research participants and Sex and Gender in Research.

Reporting on sex and gender	This is not relevant for this study.
Population characteristics	This is not relevant for this study.
Recruitment	This is not relevant for this study.
Ethics oversight	This is not relevant for this study.

Note that full information on the approval of the study protocol must also be provided in the manuscript.

Field-specific reporting

Please select the one below that is the best fit for your research. If you are not sure, read the appropriate sections before making your selection.

☒ Life sciences ☐ Behavioural & social sciences ☐ Ecological, evolutionary & environmental sciences

For a reference copy of the document with all sections, see nature.com/documents/nr-reporting-summary-flat.pdf

Life sciences study design

All studies must disclose on these points even when the disclosure is negative.

Sample size

No statistical method was used to predetermine sample size. Sample sizes were chosen according to common practices in enzyme research. The chosen sample sizes are standard for investigations of this kind and were chosen as sufficient to represent any variance present in the samples but also to be within the technically practical limits for performing the experiment. There is no quantitative data that would require additional data points for statistical analysis. Recombinant proteins were purified at least once, each purification was documented by SDS PAGE, and separate Coomassie-stained gels displaying all constructs were included in the manuscript (Supplementary Fig. 1a-c). To analyse the elongation of compound 6 and 7, at least three reactions were performed with highly consistent results (Supplementary Fig. 6a). The crystallization construct was purified three times with similar results using the protocol shown in Supplementary Fig. 8. Activity of Cps3D constructs was tested at least two times with very similar results (Supplementary Fig. 11c). Elongation of capsule polymer fragments with inactive truncations of Cps7D and Cps3D was performed at least two times with very similar results (Supplementary Fig. 12c). To analyze the elongation mechanism of CpsD constructs, at least six reactions were performed at different donor to acceptor ratios using the active

truncations of CpsD with highly consistent results (Extended Data Fig. 8). The elongation of poly(Gro3P) followed by PAGE analysis to document the activating effect was repeated five times with very similar results for the App3 biosynthesis system (Supplementary Fig. 13d) and two times with very similar results for the App7 biosynthesis system (Extended Data Fig. 5b). Scaled-up purification of App3 and App7 polymer was performed at least three times, and purified polymer was hydrolysed and fractionated at least two times with highly similar results (Supplementary Fig. 15c, d, f). The elongation of compound 8 was performed at least three times with very similar results (Supplementary Fig. 13c). The elongation of CpsAC products with CpsD and active CpsD truncations was analysed at least three times (Fig. 6b,d, Extended Data Fig. 4d).

Data exclusions	No data were excluded from the analyses.
Replication	see "sample sizes"
Randomization	The experiments were not randomized, because no allocation of samples into experimental groups was required. In our experimental set up, defined enzyme variants were compared under well controlled conditions. Accordingly, the assays performed in this study did not depend on statistical analyses of an unknown relationship, but required a rational approach for activity comparison.
Blinding	The Investigators were not blinded to allocation during experiments and outcome assessment, because results did not require subjective judgment or interpretation.

Reporting for specific materials, systems and methods

We require information from authors about some types of materials, experimental systems and methods used in many studies. Here, indicate whether each material, system or method listed is relevant to your study. If you are not sure if a list item applies to your research, read the appropriate section before selecting a response.

Materials & experimental systems

n/a	Involved in the study
☒	☐ Antibodies
☒	☐ Eukaryotic cell lines
☒	☐ Palaeontology and archaeology
☒	☐ Animals and other organisms
☒	☐ Clinical data
☒	☐ Dual use research of concern

Methods

n/a	Involved in the study
☒	☐ ChIP-seq
☒	☐ Flow cytometry
☒	☐ MRI-based neuroimaging
