# Supplementary figures and images for: Transition transferases prime bacterial capsule polymerization
Source: Nat Chem Biol. 2024 Jul 1;21(1):120–30. doi: 10.1038/s41589-024-01664-8 (PMC11666461; doi:10.1038/s41589-024-01664-8)

Figure 6b

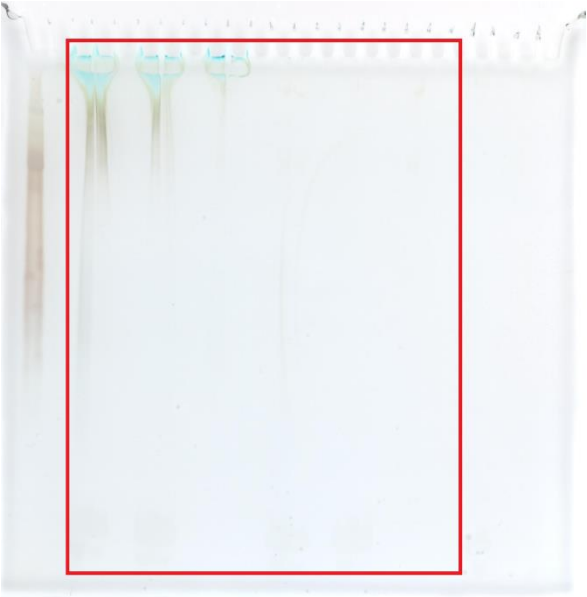

Figure 6d

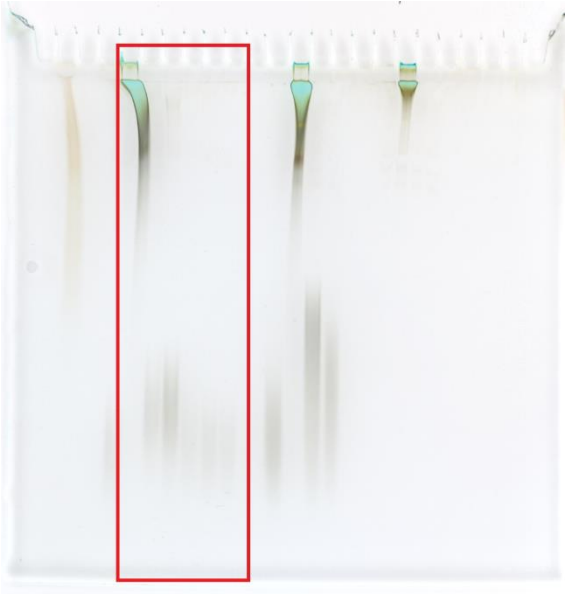

Supplement: Supplementary file 3 — Unprocessed gels. [file 41589_2024_1664_MOESM3_ESM.pdf]

Extended Data Figure 4d

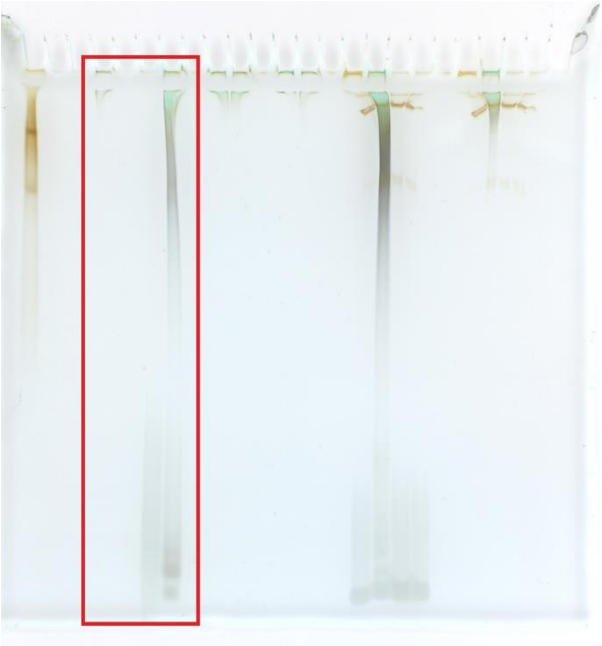

Supplement: Supplementary file 4 — Unprocessed gel. [file 41589_2024_1664_MOESM4_ESM.pdf]

Extended Data Figure 5b

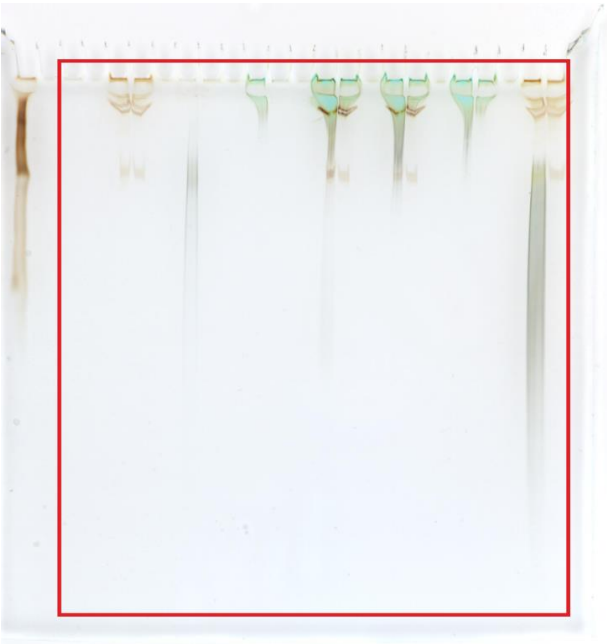

Supplement: Supplementary file 5 — Unprocessed gel. [file 41589_2024_1664_MOESM5_ESM.pdf]
